# Interactive Regulation of Formate Dehydrogenase during CO_2_ Fixation in Gas-Fermenting Bacteria

**DOI:** 10.1128/mBio.00650-20

**Published:** 2020-08-18

**Authors:** Lu Zhang, Yanqiang Liu, Ran Zhao, Can Zhang, Weihong Jiang, Yang Gu

**Affiliations:** a Key Laboratory of Synthetic Biology, CAS Center for Excellence in Molecular Plant Sciences, Shanghai Institute of Plant Physiology and Ecology, Chinese Academy of Sciences, Shanghai, China; b University of Chinese Academy of Sciences, Beijing, China; University of California, Irvine

**Keywords:** CO_2_ fixation, gas-fermenting *C. ljungdahlii*, interactive regulation, lysine acetylation, transcription factor

## Abstract

Protein lysine acetylation, a prevalent posttranslational modification, regulates numerous crucial biological processes in cells. Nevertheless, how lysine acetylation interacts with other types of regulation to coordinate metabolism remains largely unknown owing to the complexity of the process. Here, using a representative gas-fermenting bacterium, Clostridium ljungdahlii, we revealed a novel regulatory mechanism that employs both the lysine acetylation and transcriptional regulation systems to interactively control CO_2_ fixation, a key biological process for utilizing this one-carbon gas. A dominant lysine acetyltransferase/deacetylase system, At2/Dat1, was identified and found to regulate FDH1 (formate dehydrogenase responsible for CO_2_ fixation) activity via a crucial acetylation site (lysine-29). Notably, the global transcription factor CcpA was also shown to be regulated by At2/Dat1; in turn, CcpA could directly control At2 expression, thus indicating an unreported interaction mode between the acetylation system and transcription factors. Moreover, CcpA was observed to negatively regulate FDH1 expression, which, when combined with At2/Dat1, leads to the collaborative regulation of this enzyme. Based on this concept, we reconstructed the regulatory network related to FDH1, realizing significantly increased CO_2_ utilization by C. ljungdahlii.

## Abstract (precis)

Microbial CO_2_ fixation and conversion constitute a potential solution to both utilization of greenhouse gas or industrial waste gases and sustainable production of bulk chemicals and fuels. Autotrophic gas-fermenting bacteria play central roles in this bioprocess. This study provides new insights regarding the metabolic regulatory mechanisms underlying CO_2_ reduction in Clostridium ljungdahlii, a representative gas-fermenting bacterium. A critical formate dehydrogenase (FDH1) responsible for fixing CO_2_ and a dominant reversible lysine acetylation system, At2/Dat1, were identified. Furthermore, FDH1 was found to be interactively regulated by both the At2/Dat1 system and the global transcriptional factor CcpA, and the two regulatory systems are mutually restricted. Reconstruction of this multilevel metabolic regulatory module led to improved CO_2_ metabolism by C. ljungdahlii. These findings not only substantively expand our understanding but also provide a potentially useful metabolic engineering strategy for microbial carbon fixation.

## INTRODUCTION

Protein lysine acetylation (PLA) constitutes a crucial metabolic regulatory mechanism in all domains of life (1). This dynamic and reversible posttranslational modification process was first discovered in eukaryotic cells in the 1960s (2) and subsequently found to regulate numerous eukaryotic cellular processes (3). For prokaryotes, since PLA was first shown to regulate acetyl-coenzyme A (Ac-CoA) synthase activity in Salmonella enterica (4), multiple other PLA-regulated cellular functions have been revealed in bacteria, e.g., virulence (5), stress response (6), essential metabolism, and DNA replication and modification (7), suggesting the importance of PLA in metabolic regulation in prokaryotes. However, knowledge regarding these PLA-affected processes remains confined to a very limited number of bacteria, representing only a small subset of the whole repertoire of biological processes in prokaryotes. Thus, the elucidation of additional PLA-mediated metabolic regulatory mechanisms is required to provide a better understanding of PLA functions in prokaryotes.

In particular, the details regarding PLA interaction or cooperation with other types of metabolic regulation (e.g., transcriptional or translational) remain poorly understood owing to their complexity. Recently, it was reported that some transcription factors (TFs) could regulate the expression of lysine acetyltransferase-coding genes in bacteria, leading to various phenotypic changes (7). These results demonstrate that the regulation of acetylation systems occurs at the transcriptional level and can affect cellular functions. Nevertheless, issues such as whether and how TFs and acetylation systems cooperate in regulation remain to be addressed.

Gas-fermenting *Clostridium* species, an important group of autotrophic acetogenic bacteria, have received considerable attention for their ability to use CO_2_ and/or syngas to produce multiple important bulk chemicals and fuels, showcasing a huge industrial application value (8). These bacteria sequestrate and assimilate CO_2_ via the Wood-Ljungdahl (reductive acetyl-CoA) pathway (WLP), the most efficient among the six different pathways known for microbial carbon fixation (9). Recently, it was observed that the genome-wide transcriptional profile of gas-fermenting Clostridium ljungdahlii differed significantly in the presence of sugars and C_1_ gases (10). Furthermore, our initial acetylproteomic analysis, a methodology used to identify all the acetylated proteins by enriching lysine-acetylated peptides with anti-acetyllysine antibodies and then identifying peptides with mass spectrometric (MS) analysis (11), revealed a large number of peptides with lysine acetylation sites in C. ljungdahlii under the gas fermentation condition, revealing numerous crucial proteins as being extensively acetylated. These findings suggested that both transcriptional regulation and protein lysine acetylation systems play important roles in regulating the gas utilization of C. ljungdahlii and that the underlying mechanisms warrant further exploration.

Here, based on the acetylproteome data, we identified a pair of enzymes, acetyltransferase/NAD^+^-dependent deacetylase (At2/Dat1), which are responsible for the reversible PLA in C. ljungdahlii, a representative gas-fermenting bacterium. Furthermore, we discovered that the At2/Dat1 system could interact with the global TF catabolite control protein A (CcpA), achieving interregulation of each other’s activity. Notably, the key formate dehydrogenase (FDH1) that is responsible for carbon fixation in C. ljungdahlii was found to be interactively regulated by the At2/Dat1 system and CcpA, which subsequently affected CO_2_ assimilation. Based on these findings, we reconstructed the regulatory model, realizing improved CO_2_ utilization in C. ljungdahlii. Our studies highlight a previously unknown mechanism for regulating carbon fixation in autotrophic gas-fermenting bacteria, effectively extending our understanding regarding metabolic regulation by lysine acetylation.

## RESULTS

### Protein lysine acetylation status and the underlying system of C. ljungdahlii grown in the presence of C_1_ gases.

**FIG 1:**
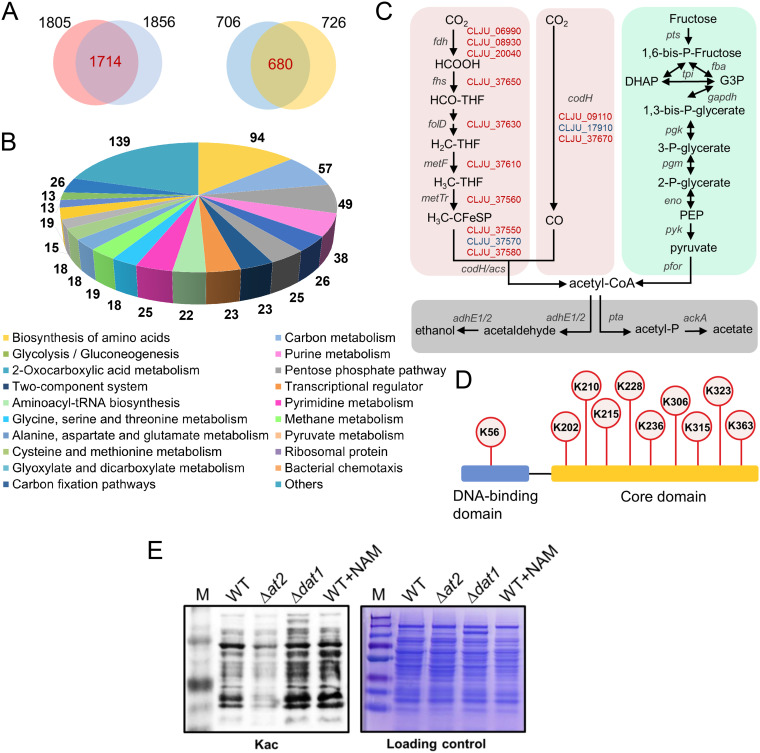
Acetylproteomic analysis of C. ljungdahlii. (A) The number of lysine acetylation sites and acetylated proteins uncovered in C. ljungdahlii. (B) Global protein lysine acetylation in C. ljungdahlii. (C) Acetylated enzymes in the Wood-Ljungdahl pathway (pink), glycolysis (cyan), and product synthesis (gray). The genes encoding the acetylated enzymes of the Wood-Ljungdahl pathway are highlighted in red. *fdh*, formate dehydrogenase; *fhs*, formyl-THF synthetase; *fold*, formyl-THF cyclohydrolase/methylene-THF dehydrogenase; *metF*, methylene-THF reductase; *metTr*, methyltransferase; *codH/acs*, carbon monoxide dehydrogenase/acetyl-CoA synthase; *codH*, carbon monoxide dehydrogenase; *pts*, phosphotransferase; *tpi*, triose phosphofructokinase; *fba*, fructose-1,6-bisphosphate aldolase; *gapdh*, glyceraldehyde-3-phosphate dehydrogenase; *pgk*, phosphoglycerate kinase; *pgm*, phosphoglycerate mutase; *eno*, phosphopyruvate hydratase; *pyk*, pyruvate kinase; *pfor*, pyruvate:ferredoxin oxidoreductase; *adhE1/2*, aldehyde/alcohol dehydrogenase 1 & 2; *pta*, phosphotransacetylase; *ackA*, acetate kinase. (D) CcpA is also highly acetylated at multiple lysine residues. (E) Changes in the global acetylation of C. ljungdahlii after deletion of the deacetylase-coding gene *dat1* (CLJU_c01320). The global acetylation of the wild-type (WT) and *at2*-deleted strain (Δ*at2*) in addition to the WT strain grown in the presence of nicotinamide (NAM), an inhibitor of deacetylase, was used as the control. Kac: lysine (K) acetylation.

To investigate the overall PLA status in C. ljungdahlii during gas fermentation, acetylproteomic analyses (two biological replicates) were performed using cells grown in mixed CO_2_/CO gas. The two sets of acetylproteome data revealed 1,805 and 1,856 acetylation sites that matched 706 and 726 proteins, respectively (Fig. 1A). These acetylated proteins covered approximately 18% of the total proteins and participated in multiple crucial physiological and metabolic processes in C. ljungdahlii (Fig. 1B). Moreover, most of these proteins were acetylated at multiple sites, exhibiting high acetylation levels. Hence, these results demonstrated that PLA is prevalent in C. ljungdahlii cells under the condition of gas fermentation.

Notably, the majority of the enzymes (11/13) in the WLP, which is responsible for carbon fixation and assimilation in C. ljungdahlii, were acetylated including FDH1 to FDH3 (encoded by CLJU_c06990, CLJU_c20040, and CLJU_c08930, respectively) required for the first step (CO_2_ reduction) (Fig. 1C), suggesting that lysine acetylation can directly regulate these processes in C. ljungdahlii. Additionally, the global TF CcpA was also found to be highly acetylated, harboring multiple acetylated lysine residues (Fig. 1D).

Next, we attempted to investigate whether C. ljungdahlii has a major reversible PLA system consisting of acetyltransferase and deacetylase, which may play a key role in protein lysine acetylation and deacetylation. Based on the Kyoto Encyclopedia of Genes and Genomes (KEGG) database (http://www.kegg.jp/) and SMART analysis (http://smart.embl-heidelberg.de/), we predicted 14 acetyltransferases and one NAD^+^-dependent deacetylase in C. ljungdahlii. Notably, these enzymes all exhibit very low amino acid sequence identity (<30%) with the reported prokaryotic acetyltransferases and deacetylases. In a screen of these enzymes using Western blot analysis, we found that the global protein acetylation levels were significantly reduced and elevated in mutants with deletions of *at2* and *dat1*, respectively, compared with the level of the wild-type strain (Fig. 1E; see also Fig. S1 in the supplemental material), whereas no obvious change was observed following deletion of the other 13 potential acetyltransferase-coding genes (Fig. S1). These results suggest that At2 and Dat1 constitute the key acetyltransferase and deacetylase, respectively, in C. ljungdahlii, thereby comprising a major reversible PLA system in this autotrophic gas-fermenting bacterium.

10.1128/mBio.00650-20.1 FIG S1 Identification of the major PLA system in C. ljungdahlii. The Western blot analysis for detecting changes in global acetylation of C. ljungdahlii after separate deletion of 14 acetyltransferase-coding genes was performed. *at1*, CLJU_c06400; *at2*, CLJU_c08980; *at3*, CLJU_c10850; *at4*, CLJU_c21300; *at5*, CLJU_c22490; *at6*, CLJU_c27210; *at7*, CLJU_c29300; *at8*, CLJU_c30550; *at9*, CLJU_c32330; *at10*, CLJU_c35040; *at11*, CLJU_c35080; *at12*, CLJU_c35110; *at13*, CLJU_c38200; *at14*, CLJU_c39010. Kac, lysine (K) acetylation. Download FIG S1, TIF file, 1.7 MB. Copyright © 2020 Zhang et al. 2020 Zhang et al. This content is distributed under the terms of the Creative Commons Attribution 4.0 International license.

### At2/Dat1-mediated reversible acetylation of the CO_2_-reducing enzyme, FDH, markedly influences cellular behaviors in gas fermentation.

**FIG 2:**
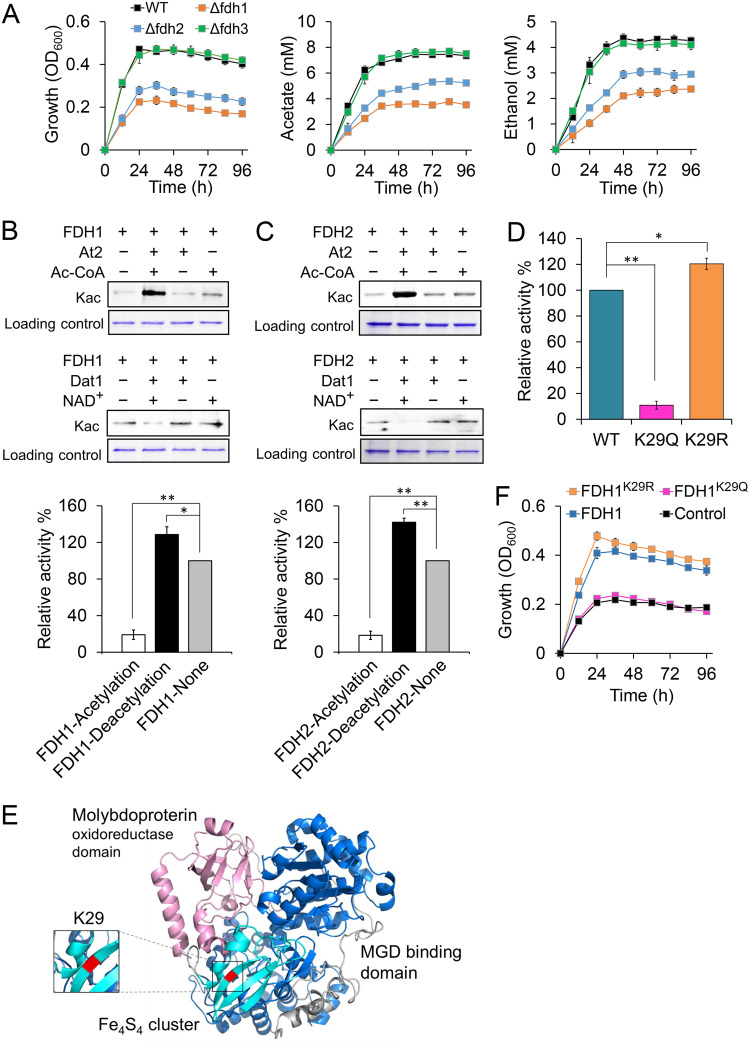
Regulation of FDH1 and FDH2 by At2/Dat1-mediated reversible acetylation. (A) Growth and product synthesis of the wild-type (WT), Δ*fdh1*, Δ*fdh2*, and Δ*fdh3* strains grown on CO_2_. (B and C) Influence of At2 or Dat1 treatment on the acetylation status and activity of FDH1 and FDH2. His-tagged FDH1 and FDH2 were expressed in Escherichia coli and then purified for *in vitro* acetylation by At2 or deacetylation by Dat1. (D) Altered FDH1 activities by an acetylation- or deacetylation-mimicking mutation at lysine residue K29. (E) Location of the K29 residue in the three-dimensional structure of FDH1. The molybdopterin oxidoreductase domain, molybdopterin guanine dinucleotide (MGD) binding domain, and Fe_4_S_4_ cluster are shown in violet, blue, and cyan, respectively. The K29 residue is shown in red. (F) Effects of FDH1 and its variants (FDH1^K29Q^ and FDH1^K29R^) on the growth property of C. ljungdahlii grown on CO_2_. Data are presented as the means ± standard deviations (error bars; *n *= 3). Statistical analysis was performed by a two-tailed Student's *t* test. *, *P < *0.05; **, *P < *0.01.

To confirm which FDH is crucial for the CO_2_ fixation in C. ljungdahlii, we generated mutants with deletion of each *fdh* gene (Fig. S2) and compared their growth properties and those of the wild-type strain grown in the presence of CO_2_. The results showed that the Δ*fdh1* and Δ*fdh2* cells grew much more slowly than the wild-type cells, leading to reduced acetate and ethanol production; in contrast, no obvious change was observed for the Δ*fdh3* mutant (Fig. 2A). These data suggested that FDH1 and FDH2 may play key roles in catalyzing CO_2_ fixation in C. ljungdahlii.

10.1128/mBio.00650-20.2 FIG S2 Confirmation of the deletions of the *fdh1*, *fdh2*, and *fdh3* genes by PCR amplification and electrophoresis. The 2.4-kb and 4.5-kb bands represent the PCR-amplified product from the *fdh*-deleted and wild-type C. ljungdahlii strains, respectively. Download FIG S2, TIF file, 1.3 MB. Copyright © 2020 Zhang et al. 2020 Zhang et al. This content is distributed under the terms of the Creative Commons Attribution 4.0 International license.

Next, we investigated whether lysine acetylation affects the activities of FDH1 and FDH2. The purified FDH1 and FDH2 were separately treated *in vitro* by purified At2 and Dat1 (Fig. S3). As expected, significantly increased and decreased acetylation levels of these two FDH enzymes were observed following At2 and Dat1 treatment (Fig. 2B and C), respectively, demonstrating that At2/Dat1 can catalyze the reversible acetylation of these two FDHs. Additionally, the increased acetylation level led to 80.9% and 81.6% loss in the activities of FDH1 and FDH2, respectively, whereas deacetylation enhanced their activities by 28.7% and 42.1% (Fig. 2B and C).

10.1128/mBio.00650-20.3 FIG S3 Confirmation of the purified Dat1 and At2 by SDS-PAGE. The molecular weights of Dat1 and At2 were 27.7 kDa and 18.5 kDa, respectively. Download FIG S3, TIF file, 1.5 MB. Copyright © 2020 Zhang et al. 2020 Zhang et al. This content is distributed under the terms of the Creative Commons Attribution 4.0 International license.

Because both FDH1 and FDH2 harbor multiple acetylated lysine residues (Fig. S4), we next sought to identify key acetylation lysine residues that determine the activity of these two FDHs. Toward this end, acetylation-mimicking mutations (K→Q) were introduced at these lysine residues, generating different variants for enzymatic activity analysis. As shown in Fig. S5A, among the eight site substitutions of FDH2, the K92Q and K275Q mutations led to relatively significant loss in activity (29% and 27%, respectively); simultaneously, among the 17 acetylated lysine residues of FDH1, a K29Q mutation markedly impaired the activity (86.2% loss) (Fig. S5B), indicating that potential acetylation of this lysine residue would significantly diminish FDH1 activity. Based on this finding, we further generated a deacetylation-mimicking mutation (K→R) at K29; as expected, the activity of the derived FDH1^K29R^ variant was 20.4% higher than that of the original FDH1 (Fig. 2D), further confirming the importance of this lysine residue for FDH1. According to the result of homology modeling (Fig. 2E), the location of the K29 residue is inside the Fe_4_S_4_ cluster of FDH1, which is known to expedite electron transfer and enzymatic reaction (12). Therefore, the higher acetylation level of K29 may impact the electron transfer, thereby decreasing FDH1 activity.

10.1128/mBio.00650-20.4 FIG S4 Acetylated lysine residues in FDH1 and FDH2. (A) Acetylated lysine residues in FDH1: K29, K64, K96, K196, K367, K393, K466, K517, K544, K616, K677, K682, K687, K690, K694, K697, and K704. (B) Acetylated lysine residues in FDH2: K46, K57, K92, K259, K270, K275, K363, and K678. Download FIG S4, TIF file, 2.7 MB. Copyright © 2020 Zhang et al. 2020 Zhang et al. This content is distributed under the terms of the Creative Commons Attribution 4.0 International license.

10.1128/mBio.00650-20.5 FIG S5 Identification of the key lysine acetylation sites of FDH1, FDH2, and CcpA. (A) Effect of each acetylated lysine residue on the activity of FDH2. (B) Effect of each acetylated lysine residue on the activity of FDH1. Data are represented as the means ± standard deviations (error bars; *n *= 3). Statistical analysis was performed by a two-tailed Student’s *t* test. ***, *P < *0.001; **, *P < *0.01; *, *P < *0.05, for results versus those of the wild-type (WT) FDH1 or FDH2. (C) Acetylated lysine residues found in the global transcription factor CcpA by LC-MS/MS. (D) Effects of acetylation-mimicking mutations at acetylated lysine sites on CcpA DNA-binding activity. Binding to the promoter of the CLJU_c20590 gene was determined by EMSA. Download FIG S5, TIF file, 1.6 MB. Copyright © 2020 Zhang et al. 2020 Zhang et al. This content is distributed under the terms of the Creative Commons Attribution 4.0 International license.

Next, we investigated the physiological role of FDH1 acetylation at the K29 residue in C. ljungdahlii by comparing the growth properties of different Δ*fdh1* strains in gas fermentation. As shown in Fig. 2F, expression of either the original *fdh1* or the mutated *fdh1* encoding FDH1^K29R^ could restore the growth ability of the Δ*fdh1* strain in gas fermentation, whereas the mutated *fdh1* encoding FDH1^K29Q^ had no effect, consistent with the results of *in vitro* enzymatic activity assay.

### Acetylation system At2/Dat1 also controls the global transcription factor CcpA.

**FIG 3:**
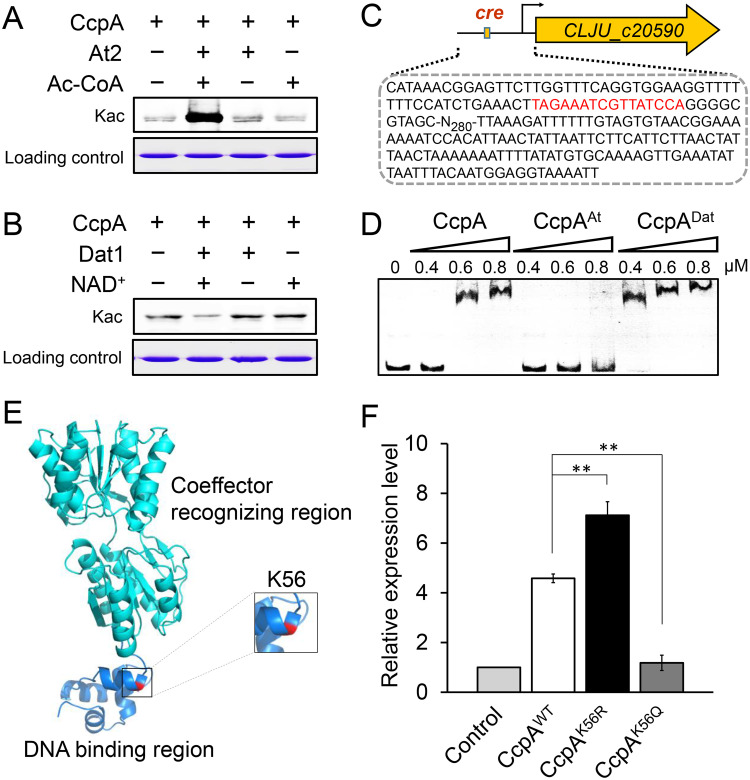
Effect of acetylation on CcpA DNA-binding activity. (A and B) Western blot analysis for uncovering At2-mediated acetylation (A) and Dat1-mediated deacetylation (B) of CcpA. (C and D) Binding of the original CcpA, CcpA^At^ (*in vitro* At2 treatment), and CcpA^Dat^ (*in vitro* Dat1 treatment) to the promoter of the CLJU_c20590 gene. The CcpA-binding *cre* site contained in the promoter of the CLJU_c20590 gene is shown in red in panel C. (E) Location of the K56 lysine residue in the three-dimensional structure of CcpA. (F) Transcriptional differences of the CLJU_c20590 gene in the presence of the WT CcpA and its variants (CcpA^K56Q^ and CcpA^K56R^). The Δ*ccpA* strain containing an empty plasmid was used as the control. Data are presented as the means ± standard deviations (error bars; *n *= 3). Statistical analysis was performed by a two-tailed Student's *t* test. **, *P < *0.01.

As mentioned above, the global TF CcpA was also found to be acetylated. To determine whether acetylation directly affects CcpA function, we obtained CcpA proteins with increased and decreased acetylation levels by *in vitro* treatment with At2 and Dat1 (Fig. 3A and B), respectively, and compared DNA-binding activities of the resultant CcpA^At^ and CcpA^Dat^ with the activity of the untreated CcpA using electrophoretic mobility shift assays (EMSAs). Specifically, a 485-bp DNA fragment covering the promoter region (which contains a CcpA-binding *cre* site) of the CLJU_c20590 gene was obtained by PCR amplification and then used as the DNA probe in EMSA (Fig. 3C). As shown in Fig. 3D, compared to the original CcpA, CcpA^Dat^ yielded a clear mobility shift at a lower protein concentration (0.4 μM), whereas no evident mobility shift was observed for CcpA^At^ even at a protein concentration of 0.8 μM, indicating that the acetylation level could affect the DNA-binding activity of CcpA.

Next, we sought to examine the influence of acetylation at the 10 lysine residues on CcpA by using acetylation-mimicking mutations (K→Q) (Fig. S5C). The results showed that only the K56Q mutation led to obviously weaker CcpA-DNA binding (Fig. S5D), indicating the potential importance of acetylation at this lysine residue for CcpA binding affinity to targets. Furthermore, homology modeling analysis showed that the K56 residue is located in the DNA-binding domain of CcpA (Fig. 3E). To further ascertain whether an acetylation-mimicking mutation at this residue could impair CcpA recognition and binding to target genes *in vivo*, the original wild-type (WT) CcpA protein (CcpA^WT^) and its two mutants (i.e., CcpA^K56Q^ and CcpA^K56R^) were separately introduced into the C. ljungdahlii Δ*ccpA* strain to examine their influence on the expression of the target gene, CLJU_c20590. As expected, compared to the level in the control strain (the Δ*ccpA* strain containing an empty plasmid), the expression of CLJU_c20590 was upregulated by 4.6- and 7.1-fold upon CcpA^WT^ and CcpA^K56R^ overexpression, respectively, whereas no obvious effect was observed for CcpA^K56Q^ (Fig. 3F).

### CcpA directly represses the expression of major acetyltransferase At2 and formate dehydrogenase FDH1.

**FIG 4:**
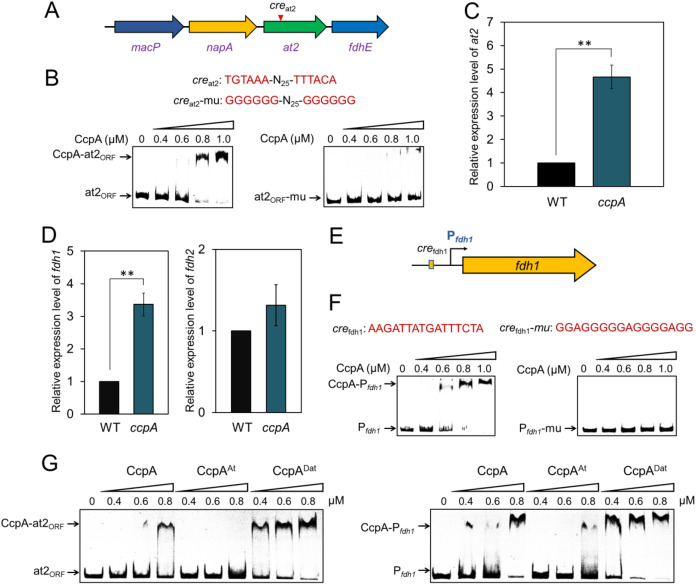
CcpA directly regulates expression of At2 and FDH1 in C. ljungdahlii. (A) Genetic location of *at2* on the chromosome of C. ljungdahlii and its putative CcpA-binding site (inverted triangle). *macP*, methyl-accepting chemotaxis protein; *napA*, Na^+^/H^+^ antiporter; *fdhE*, formylmethanofuran dehydrogenase subunit E. (B) EMSA of CcpA binding to the *cre*_at2_ site. (C and D) qRT-PCR analysis of transcriptional changes of *at2* and *fdh1* and *fdh2*, as indicated, following *ccpA* disruption in C. ljungdahlii. Data are presented as the means ± standard deviations (error bars; *n *= 3). Statistical analysis was performed by a two-tailed Student's *t* test. **, *P < *0.01. (E) Location of the CcpA-binding site, *cre*_fdh1_, in the promoter region of *fdh1*. (F) EMSA of CcpA binding to the *cre*_fdh1_ site. (G) Different binding affinities of the original CcpA, CcpA^At^ (*in vitro* At2 treatment), and CcpA^Dat^ (*in vitro* Dat1 treatment) to the *cre*_at2_ and *cre*_fdh1_ sites. P*_fdh1_*, the DNA probe containing the *cre*_fdh1_ site; at2_ORF_, the DNA probe containing the *cre*_at2_ site.

To evaluate whether CcpA, as a global TF in Gram-positive bacteria (13), can conversely regulate the major acetylation system AT2/Dat1 in C. ljungdahlii, we visually scanned the coding and promoter regions of At2 and Dat1, identifying a palindromic sequence within the At2-coding region (designated *cre*_at2_) (Fig. 4A) that was similar to the recently reported CcpA-binding motif *cre_var_* (14). The results of an EMSA using the purified CcpA and a *cre*_at2_-containing DNA probe (at2_ORF_, where ORF is open reading frame) demonstrated that CcpA could bind to at2_ORF_ (Fig. 4B), whereas mutation of *cre*_at2_ clearly attenuated their binding affinity (Fig. 4B). Furthermore, we investigated the influence of CcpA on *at2* expression *in vivo*. As shown in Fig. 4C, the *at2* transcript level in the *ccpA*-deleted C. ljungdahlii strain was 3.7-fold higher than that in the wild-type strain during gas fermentation using CO_2_ as the sole carbon source. These results, together with the findings shown in Fig. 3, suggest the existence of interplay between CcpA and At2 in C. ljungdahlii.

Interestingly, we also observed a 2.5-fold increase in the transcriptional level of *fdh1* following *ccpA* deletion while no obvious transcriptional change occurred for *fdh2* (Fig. 4D), indicating that the *fdh1* expression is negatively regulated by CcpA in C. ljungdahlii. Visual scanning of the coding and promoter regions of *fdh1* identified a 16-bp palindromic sequence (AAGATTATGATTTCTA) in the *fdh1* promoter region (Fig. 4E), which is similar to the canonical CcpA-binding *cre* site (WTGWAAACGWTWWCAW, in which W represents A or T) (15). Thus, we termed this site *cre*_fdh1_. An EMSA using the purified CcpA and the *fdh1* promoter fragment (P*_fdh1_*) revealed that CcpA could bind to P*_fdh1_* (Fig. 4F) whereas mutation at the *cre*_fdh1_ site abolished this binding (Fig. 4F), suggesting that CcpA could directly inhibit *fdh1* expression in C. ljungdahlii. In addition, compared to the original CcpA, CcpA^Dat^ (Dat1-treated CcpA) showed increased binding affinity to the *cre*_at2_ and *cre*_fdh1_ sites whereas CcpA^At^ (At2-treated CcpA) yielded decreased binding affinities to these two sites (Fig. 4G), further indicating that the acetylation level could affect the DNA-binding activity of CcpA to its targets.

### Interactive regulation of FDH1 by At2/Dat1 and CcpA is critical for carbon sequestration, and the regulatory model can be modified.

**FIG 5:**
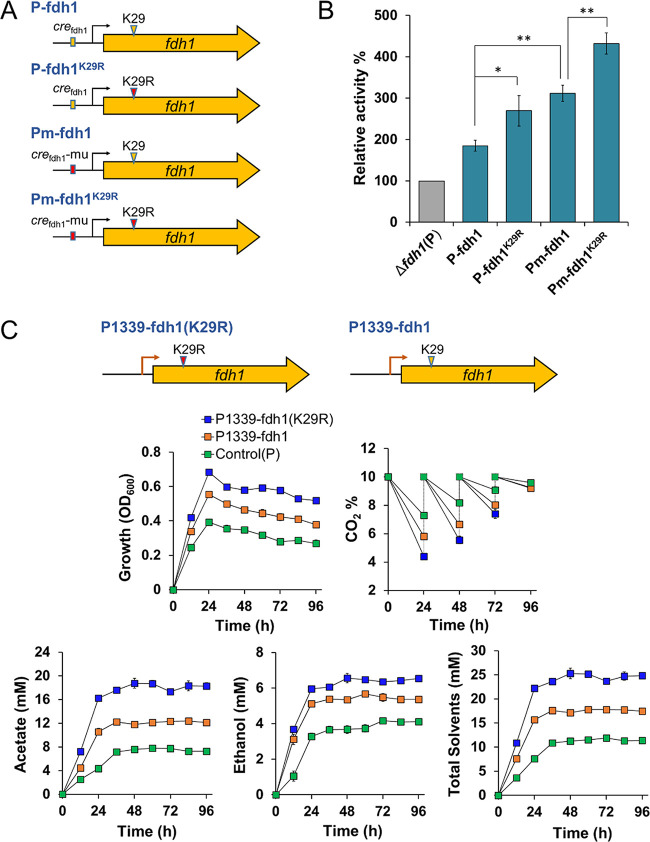
At2/Dat1-CcpA-mediated interactive regulation of FDH1. (A) Four modules for *fdh1* expression. (B) The *in vivo* FDH activities of the Δ*fdh1* strain containing modules P-fdh1, P-fdh1^K29R^, Pm-fdh1, and Pm-fdh1^K29R^. The Δ*fdh1* strain harboring the empty plasmid (P) was used as a control (gray). (C) Growth, CO_2_ consumption, and product production of C. ljungdahlii strains containing the empty plasmid or module P1339-fdh1 and P1339-fdh1(K29R) during gas fermentation. Control (P), the wild-type C. ljungdahlii strain containing an empty plasmid pMTL83151; total solvents, acetate and ethanol. Data are presented as the means ± standard deviations (error bars; *n* = 3). Statistical analysis was performed by a two-tailed Student's *t* test. *, *P* < 0.05; **, *P* < 0.01.

Next, we attempted to elucidate the interaction between the At2/Dat1 and CcpA systems for posttranslational and transcriptional FDH1 regulation. Toward this end, four expression modules were constructed (Fig. 5A): (i) P-fdh1, providing *fdh1* overexpression using its natural promoter; (ii) P-fdh1^K29R^, for overexpression of the *fdh1* mutant encoding FDH1^K29R^ (deacetylation-mimicked mutation) using the natural *fdh1* promoter; (iii) Pm-fdh1, for *fdh1* overexpression using the *cre*_fdh1_-mutated *fdh1* promoter (blocking CcpA regulation); and (iv) Pm-fdh1^K29R^, integrating the mutations described in ii and iii. These four expression modules were separately introduced into the Δ*fdh1* strain, and the *in vivo* FDH activities of the resultant mutant cells grown on CO_2_ were tested. Notably, compared to the activity in the control strain (Δ*fdh1* strain containing an empty plasmid pMTL83151), the *in vivo* FDH activities were increased by construct expression in the order P-fdh1 < P-fdh1^K29R^ < Pm-fdh1 < Pm-fdh1^K29R^ (Fig. 5B). The highest FDH activity was achieved with the module Pm-fdh1^K29R^, reaching 3.0 ± 0.2 U/mg, which was approximately 3.3-fold higher than that of the control strain (Fig. 5B). Together, these results suggested that the *in vivo* activity of FDH1 was negatively affected by either the acetylation-mimicking mutation of K29 or CcpA-mediated transcriptional regulation, with the latter having a greater effect.

To exclude the possibility that the other two FDH-coding genes (*fdh2* and *fdh3*) also contributed to the increased *in vivo* FDH activities (Fig. 5B), we compared the expression levels of *fdh2* and *fdh3* in these mutants. As expected, no obvious transcriptional changes were observed for these two genes (Fig. S6).

10.1128/mBio.00650-20.6 FIG S6 Transcriptional differences of the *fdh1*, *fdh2*, and *fdh3* genes in the C. ljungdahlii Δ*fdh1* strain in the presence of four different expression modules. The four different expression modules are P-fdh1, P-fdh^K29R^, Pm-fdh1, and Pm-fdh1^K29R^. Control, the Δ*fdh1* strain containing an empty plasmid. Data are represented as the mean ± standard deviations (error bars; *n *= 3). Statistical analysis was performed by a two-tailed Student’s *t* test. ***, *P < *0.001. Download FIG S6, TIF file, 2.4 MB. Copyright © 2020 Zhang et al. 2020 Zhang et al. This content is distributed under the terms of the Creative Commons Attribution 4.0 International license.

To potentially improve the CO_2_-utilizing ability of C. ljungdahlii, we attempted to reconstruct the metabolic regulatory network for FDH1 based on the previous findings. Two modules were constructed (Fig. 5C): (i) one for overexpression of *fdh1* under the control of P_1339_, a previously reported strong promoter in C. ljungdahlii (16) containing no CcpA-binding sites, enabling high expression of *fdh1* in the module P1339-fdh1 without CcpA-mediated repression; and (ii) one for overexpression of the *fdh1* mutant encoding FDH1^K29R^, which has much higher activity than the original FDH1, under the control of the promoter P_1339_, yielding the module P1339-fdh1(K29R). The two modules were introduced into the wild-type C. ljungdahlii strain to examine their influence on the growth properties of cells grown on CO_2_. Notably, the cells harboring the P1339-fdh1 module exhibited much faster growth and CO_2_ utilization than the control strain (containing an empty plasmid pMTL83151) (Fig. 5C). Moreover, at the end of fermentation (96 h), the CO_2_ consumption and total solvents (acetate and ethanol) were approximately 76% and 80% higher than those of the control strain, respectively (Fig. 5C). The cells harboring the P1339-fdh1(K29R) module exhibited further improved performance, reaching 129% and 124% higher CO_2_ consumption and total solvent titer, respectively, at the end of fermentation (Fig. 5C), indicating an additive effect of the strategies integrated in this module. These data further supported the concept that the *in vivo* FDH1 activity could be markedly enhanced by eliminating the negative regulation from lysine acetylation and CcpA, leading to improved CO_2_ fixation and assimilation in C. ljungdahlii.

## DISCUSSION

**FIG 6:**
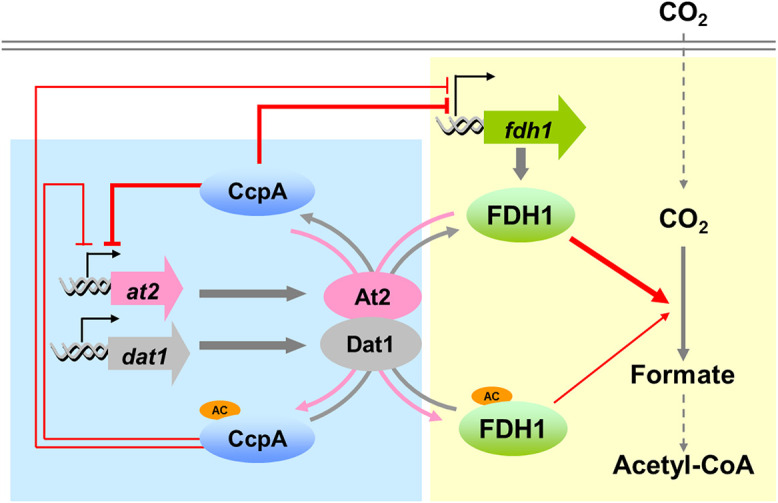
Interactive regulation of carbon fixation by the reversible lysine acetylation system At2/Dat1 and the global transcription factor CcpA in C. ljungdahlii. The thicker red arrow and T-end lines indicate stronger FDH1-based catalytic activity and CcpA-mediated transcriptional repression, respectively. The blue background emphasizes the model of CcpA-At2/Dat1 interactions. The yellow background emphasizes the model of the interactive control of CcpA and At2/Dat1 on FDH1.

Here, we reported the discovery of a complex regulatory mechanism, mediated by both the PLA system and the global TF CcpA, for controlling CO_2_ metabolism in gas-fermenting C. ljungdahlii. We revealed the interplay between the At2/Dat1 system and CcpA together with their interactive regulation of FDH1, the key enzyme catalyzing CO_2_ reduction in C. ljungdahlii (Fig. 6), providing new insights regarding the metabolic regulation of carbon fixation and assimilation in autotrophic gas-fermenting bacteria. Based on these findings, the following reconstruction of the regulatory network achieved improved behavior of C. ljungdahlii with regard to the use of CO_2_.

The overall lysine acetylation status of C. ljungdahlii proteins under the condition of gas fermentation revealed that multiple key enzymes of central metabolism were highly acetylated, e.g., phosphate-3-glyceraldehyde dehydrogenase, pyruvate carboxylase, phosphoglycerate kinase, and aldolase (see Table S1 in the supplemental material). This is consistent with previous findings in some model bacteria such as Escherichia coli, Salmonella enterica, and Bacillus subtilis (17–20), suggesting that the extensive acetylation of enzymes in central metabolism may be conserved from heterotrophic to autotrophic bacteria. In particular, lysine acetylation could significantly change the activities of FDH, which catalyzes the CO_2_ reduction reaction in the WLP. The WLP is present in numerous other important acetogenic bacteria, such as Moorella thermoacetica and Acetobacterium woodii (21–23), as well as in gas-fermenting *Clostridium* strains. Thus, it can be speculated that lysine acetylation of WLP enzymes may be common in these bacteria and may also affect their CO_2_-utilizing ability. In contrast, the status and physiological role of enzyme acetylation in other natural biological carbon fixation pathways remain poorly understood. Further elucidation of these processes will afford new insights regarding the importance of lysine acetylation-mediated metabolic regulation.

10.1128/mBio.00650-20.8 TABLE S1 List of all Clostridium ljungdahlii lysine acetylated sites identified by LC-MS/MS. Download Table S1, PDF file, 0.6 MB. Copyright © 2020 Zhang et al. 2020 Zhang et al. This content is distributed under the terms of the Creative Commons Attribution 4.0 International license.

To date, major acetyltransferases have been identified only in a small number of prokaryotes including S. enterica Pat (4), E. coli Pka (24), B. subtilis AcuA (25), and Mycobacterium tuberculosis PatA (26). These acetyltransferases contain not only a Gcn5-related *N*-acetyltransferase (GNAT) domain that catalyzes the transfer of an acetyl group from Ac-CoA to the ε-amino group of the lysine side chain but also another functional domain capable of recognizing and binding to some specific signal molecules in cells (27). Therefore, the catalytic activities of these acetyltransferases are regulated by different signal molecules, such as Ac-CoA or cAMP. However, the major acetyltransferase, At2, identified in this study exhibits a different structural feature. Structural analysis using the SMART website (http://smart.embl-heidelberg.de/) showed that At2 contained only a single GNAT domain and no signal molecule-binding domain, indicating that it may function independently without regulation by signal molecules. Whether this structural feature confers a special physiological significance to At2, such as maintaining a relatively stable catalytic activity in cells, remains to be explored.

The *in vivo* experiments in the present study demonstrated that FDH1 and FDH2 in C. ljungdahlii are crucial for CO_2_ fixation and assimilation, whereas FDH3 does not have an effect (Fig. 2). This difference may be partly attributed to the various existing forms of these three FDHs in cells. Previous studies have shown that FDH1 and FDH2, but not FDH3, are capable of forming a complex with electron-bifurcating [FeFe] hydrogenase and a small ferredoxin-like protein, respectively (28). Such a multienzyme system is likely more efficient than free enzyme components for catalyzing CO_2_ reduction by transferring electrons provided by H_2_ or ferredoxin/NADPH to FDH. Therefore, deletion of FDH1 or FDH2 would be expected to have a marked impact on CO_2_ utilization by C. ljungdahlii (Fig. 2).

To our knowledge, it remains poorly understood how autotrophic bacteria regulate FDHs, except that some metal ions and varied amounts of H_2_ were found to affect the activity of this enzyme (29, 30). It was reported previously that transcription levels of the three FDHs in C. ljungdahlii cells grown on CO_2_ were much higher than those in the cells grown on fructose (10), indicating that the *in vivo* expression of these FDHs responds to different carbon sources and that some unknown transcription factors may play a role. Here, our finding regarding the direct control by CcpA of FDH1 activity at the transcriptional level confirmed this speculation. Additionally, we also demonstrated that FDH1 activity is directly affected by At2-mediated lysine acetylation at the posttranslational level. Although protein acetylation often has less effect on enzyme activity than transcriptional regulation and thus plays an “auxiliary” role in metabolic regulation (31), it can achieve rapid modification of the target proteins (32). Since CcpA acetylation by At2 stimulates *fdh1* expression on one side but on the other side inhibits the activity of the produced FDH1 (Fig. 6), under which conditions such a scenario would make sense? To explain this question, we investigated the dynamic changes of the CcpA/FDH1 acetylation and *fdh1* transcription levels in gas fermentation. As shown in Fig. S7, the acetylation levels of FDH1 and CcpA were relatively low in the early stage but gradually increased with the extension of fermentation; meanwhile, the *fdh1* transcription also gradually increased, probably due to the decreased CcpA repression. Therefore, it seems that the At2-mediated acetylation of CcpA and FDH1 led to a complementary regulatory effect on FDH1 activity at the transcriptional and posttranslational levels, respectively, thereby maintaining a reasonable FDH activity level in cells. We speculated that such complex regulation of FDH1 by both At2 and CcpA may be more flexible than a single regulatory mechanism for coordinating carbon fixation in C. ljungdahlii.

10.1128/mBio.00650-20.7 FIG S7 The dynamic changes of the CcpA/FDH1 acetylation status and *fdh1* transcription level in gas fermentation. The purified His-tagged FDH1 and CcpA were subjected to Western blotting with the indicated antibodies (anti-AcK and anti-His tag). The Western blotting bands with anti-His tags were used as the protein loading controls. Download FIG S7, TIF file, 2.8 MB. Copyright © 2020 Zhang et al. 2020 Zhang et al. This content is distributed under the terms of the Creative Commons Attribution 4.0 International license.

The interaction model comprising the interplay between the global TF CcpA and the major acetylation system AT2/Dat1 in C. ljungdahlii has not, to our knowledge, been reported previously, although it is known that acetylation or deacetylation could affect the regulatory activities of some TFs (5, 33–36). CcpA is known to be a crucial regulator in Gram-positive bacteria, being involved in regulating multiple different important metabolic processes (13, 37). The importance of CcpA in regulating carbon metabolism has also been reported in the saccharolytic *Clostridium* species (15). Nevertheless, whether and how the *in vivo* level of CcpA is regulated in Gram-positive bacteria remain poorly understood to date, despite the elucidation of a transcriptional autoregulation mechanism for this important TF (38). Therefore, the findings in this study reveal a new regulatory mechanism that controls CcpA function in bacterial cells. We observed that At2-mediated acetylation and Dat1-mediated deacetylation could decrease and increase CcpA binding affinity to the target DNA site (Fig. 3), respectively, thus altering the repression effect of CcpA. However, it should be noted that CcpA is actually a bifunctional TF that combines the activity of transcriptional repression and activation (14). When exerting the activation effect, CcpA relies more on its ability to recruit RNA polymerase (39). Therefore, how lysine acetylation affects the interaction of CcpA with RNA polymerase along with the subsequent transcriptional activation warrants further exploration.

At present, improvement of the transcription and translation efficiencies of target genes represents a widely used metabolic engineering strategy for industrial bacteria. Here, we found that acetylation could effectively regulate FDH activity in a gas-fermenting bacterium and could be applied to alter the cellular behavior with regard to CO_2_ utilization. These results suggested that modulation of the acetylation level of target proteins may also constitute a useful metabolic engineering strategy for further development and application. In summary, this work presents a novel complex metabolic regulatory mechanism that employs both protein acetylation and transcriptional regulation systems for regulating the key carbon-reducing enzyme, FDH, in autotrophic gas-fermenting bacteria. These findings provide new insights regarding the physiological role and application value of protein lysine acetylation in bacteria.

## MATERIALS AND METHODS

### Bacterial strains, plasmids, media, and growth conditions.

E. coli strains DH5α and BL21(DE3) were used as the host for plasmid cloning and protein purification, respectively. Cells were grown in LB medium supplemented with chloramphenicol (12.5 μg/ml) or kanamycin (100 μg/ml) when needed. The wild-type C. ljungdahlii strain DSM 13528 was grown anaerobically at 37°C in yeast extract-tryptone-fructose (YTF) medium (40) and modified ATCC medium 1754 (16) for inoculum preparation and gas fermentation, respectively, and thiamphenicol (5 μg/ml) was added when needed.

All strains and plasmids used in this work are listed in Table S2 in the supplemental material. All media and C. ljungdahlii genetic techniques have been described previously (16). Anti-acetyl lysine antibody (catalog no. ICP0380) and nicotinamide (NAM; catalog no. N0636) were purchased from ImmuneChem Pharmaceuticals, Inc., and Sigma-Aldrich, respectively.

10.1128/mBio.00650-20.9 TABLE S2 Strains and plasmids used in this study. Download Table S2, PDF file, 0.1 MB. Copyright © 2020 Zhang et al. 2020 Zhang et al. This content is distributed under the terms of the Creative Commons Attribution 4.0 International license.

### Proteomic profiling of lysine acetylation.

C. ljungdahlii was grown in modified ATCC medium 1754 (no sugars) in the presence of syngas (CO−CO_2_−H_2_−N_2_, 56%/20%/9%/15%). To prevent the deacetylation reaction from moving forward in cells, NAM (50 mM) was added into the medium to inhibit the activity of the NAD^+^-dependent deacetylase. The C. ljungdahlii cells cultured to exponential growth phase (optical density at 600 nm [OD_600_] of 1.0) were harvested by centrifugation (5, 000 × *g* for 10 min at 4°C). The subsequent procedures (tryptic digestion, affinity purification of lysine-acetylated peptides, and nano-high-performance tandem MS [nano-HPLC-MS/MS]) were performed as previously described (20).

### Plasmid construction.

The primers used in this work are listed in Table S3.

10.1128/mBio.00650-20.10 TABLE S3 Primers used in this study. Download Table S3, PDF file, 0.2 MB. Copyright © 2020 Zhang et al. 2020 Zhang et al. This content is distributed under the terms of the Creative Commons Attribution 4.0 International license.

For *at2* deletion, the CRISPR/Cas9 editing plasmid pMTLcas-*at2* was constructed as follows: the linear plasmid pMTLcas containing the native *cas9* from Streptococcus pyogenes was first obtained by double digestion with SalI and XhoI. Then, the small guide RNA (sgRNA) (which targets the 20-nucleotide [nt] target spacer for the *at2* gene) was obtained by PCR amplification using the primers at2gRNA-for/at2gRNA-rev and pMTLcas as the template. The two homologous arms (HAs) that flank the coding region of *at2* were amplified from C. ljungdahlii genomic DNA using the primers at2UpArm-for/at2UpArm-rev and at2DownArm-for/at2DownArm-rev, and then linked to sgRNA *via* overlapping PCR using the primers at2gRNA-for/at2DownArm-rev, yielding the DNA fragment sgRNA-HA. Finally, the linear pMTLcas vector and sgRNA-HA fragment were assembled using a ClonExpress MultiS One Step cloning kit (Vazyme Biotech Co., Ltd., Nanjing, China), yielding the plasmid for *at2* deletion in C. ljungdahlii.

The other CRISPR/Cas9 editing plasmids (pMTLcas-*dat1*, pMTLcas-*fdh1*, pMTLcas-*fdh2*, pMTLcas-*fdh3*, and pMTLcas-*ccpA*) were constructed in a similar manner, except that the cassettes of the homologous arms were changed accordingly.

The construction of the plasmid pMTL83151-P_1339_-*fdh1* was performed as follows: the promoter P_1339_ was PCR-amplified from the genome of C. ljungdahlii by using the primers P_1339_-for/P_1339_-fdh1-rev and was then assembled with the *fdh1* gene via overlapping PCR by using the primers fdh1-P_1339_-for/fdh1-rev. The resulting DNA fragment and the linear pMTL83151 vector were assembled by using the ClonExpress MultiS One Step Cloning kit, yielding the plasmid for *fdh1* overexpression. Plasmid pMTL83151-P_1339_-*fdh1*-m was constructed in a similar manner, except that the primers (fdh1-m-for/fdh1-m-rev) used for overlapping PCR contained a mutated K29 site.

### Gene expression and protein production.

The CLJU_c08980, CLJU_c01320, CLJU_c06990, CLJU_c20040, and CLJU_c01900 genes, encoding At2, Dat1, FDH1, FDH2, and CcpA, respectively, were obtained by PCR amplification using the genomic DNA of C. ljungdahlii as the template. Notably, in order to efficiently express the *fdh1* and *fdh2* genes in E. coli, the stop codon TGA in *fdh1* and *fdh2*, encoding selenocysteine at positions 139 and 135, respectively, was changed to TGT (encoding cysteine) during cloning. These genes were inserted into the plasmid pET-28a (Invitrogen) and transferred into the E. coli BL21(DE3) strain for expression. The subsequent protein purification procedure was the same as previously described (15).

### EMSA.

Two-step PCR amplification was used to prepare the DNA probes. First, double-stranded DNA fragments were amplified from the genomic DNA of C. ljungdahlii using specific primer pairs containing a universal primer sequence (5′-AGCCAGTGGCGATAAG-3′) at the 5′ terminus. Next, a Cy5 tag was added to the amplified DNA fragment by PCR using the universal primer labeled with Cy5. The PCR products were analyzed by agarose gel electrophoresis and recovered using a PCR purification kit (catalog no. AP-GX-250; Axygen) and then used as probes for EMSAs. EMSAs were carried out according to the protocol as previously described (15).

### Real-time qRT-PCR.

C. ljungdahlii and the derivative mutants were grown anaerobically in modified ATCC medium 1754 using CO_2_/H_2_ at 37°C. Cells were harvested when they reached an optical density (OD_600_) of 1. Total RNA was isolated using a kit (catalog no. cw0581; CWBIO), according to the manufacturer’s instructions, and then treated by DNase I (TaKaRa) to eliminate DNA contained in the extracted samples. The RNA concentration was determined by using a NanoDrop spectrophotometer (Thermo Fisher Scientific, Waltham, MA). cDNA was obtained by reverse transcription using a PrimeScript RT reagent kit (TaKaRa). The procedure of real-time qPCR (qRT-PCR) was the same as described previously (38). The primers used for qRT-PCR are listed in Table S3. Here, the *rho* gene (CLJU_c02220) was used as the internal control (41).

### Western blotting.

The concentrations of the protein samples were first determined using a kit from Sangon Biotech Co., Ltd., China. Next, the protein samples were separated by 12% SDS-PAGE and then transferred to polyvinylidene difluoride (PVDF) membranes for 45 to 60 min at 400 mA. The membrane was blocked at room temperature in 1× Tris-buffered saline with Tween 20 ([TBST] 10 mM Tris-HCl, pH 7.4, 100 mM NaCl and 0.2% Tween 20) containing 5% nonfat dry milk for 30 min. Anti-acetyl-lysine antibody diluted in TBST–0.5% nonfat dry milk (1:15,000) was used as the primary antibody and incubated with the membrane for 1 h; then the blot was washed with TBST three times. Goat horseradish peroxidase-conjugated anti-rabbit antibody diluted in TBST (1:5,000) was used as the secondary antibody and incubated with the membrane for 1 h, followed by three washes with TBST. Finally, an enhanced chemiluminescence (ECL) system (ImageQuant LAS 4000 Mini; GE) was used for signal detection according to the manufacturer’s instructions.

### FDH activity assay.

An FDH activity assay was performed as previously reported (42). In brief, the reaction mixture contained 50 mM Tris-HCl (pH 8.0), 0.1 M NaHCO_3_, 0.2 mM NADH, and 3 μM purified FDH1 or FDH2. The mixture was incubated at 37°C for 1 h, and then the specific activity of FDH1 or FDH2 was assessed by measuring the change in absorbance at 340 nm consequent to the oxidation of NADH on a DU730 spectrophotometer (Beckman Coulter). Protein purification and enzymatic activity measurement were performed under anaerobic conditions.

### *In vitro* acetylation and deacetylation assays.

Acetylation activity assays were performed as previously reported (5). In brief, a 60-μl reaction system containing 50 mM Tris-HCl (pH 8.0), 2 μM target proteins, 6 μM acetyltransferase (At2), 0.2 mM acetyl-CoA, and 5% glycerol was constructed and incubated at 37°C for 3 h. Following the reaction, the samples were divided into two aliquots for Western blot analysis and acetyltransferase activity assay.

Deacetylation activity assays were performed as previously reported (5). Because Dat1 belongs to an NAD^+^-dependent deacetylase, which uses NAD^+^ as a cosubstrate during the deacetylation reaction, NAD^+^ was involved in the assay. In brief, a 60-μl reaction system containing 50 mM Tris-HCl (pH 8.0), 2 μM target proteins, 4 μM deacetylase (Dat1), 1 mM MgCl_2_, 1 mM NAD^+^, and 5% glycerol was constructed and incubated at 37°C for 3 h. Following the reaction, the samples were divided into two aliquots for Western blot analysis and deacetylase activity assay.

### Identification of acetylated lysine residues by mass spectrometry.

The purified FDH proteins were *in vitro* acetylated by the acetyltransferase (At2) in the reaction mixture. Then, the proteins contained in the mixture were separated by 12% SDS-PAGE. The excised bands containing FDH1 were digested with trypsin at 37°C for 20 h. Peptides were separated using a nano-liquid chromatography (LC)-HPLC system (Agilent HPLC-delivered solvents A [0.1% (vol/vol) formic acid in water] and B [0.1% (vol/vol) formic acid in 84% (vol/vol) acetonitrile]) and analyzed with a q-Exactive mass spectrometer. Mass spectrometric data were analyzed using Mascot, version 2.2, software for database search.

### Fermentation.

Inoculum preparation and fermentations of C. ljungdahlii were performed anaerobically in YTF medium and modified ATCC medium 1754, respectively, in which thiamphenicol (5 μg/ml) was added when needed. The detailed manipulations were similar to those previously described (16). Briefly, 100 μl of frozen stock was transferred into 5 ml of liquid YTF medium and then incubated anaerobically at 37°C for 24 h. When the optical density (OD_600_) of grown cells reached 0.8 to 1.0, 1.5 ml of the grown cells was inoculated into 30 ml of modified ATCC medium 1754 with a headspace of CO_2_-H_2_-N_2_ (10%/30%/60%; pressurized to 0.2 MPa) for fermentation. The gases were added into the headspace (again at 0.2 MPa) every 24 h.

### Analytical methods.

Cell growth was monitored based on the absorbance of the culture at *A*_600_ (OD_600_) using a spectrophotometer (DU730; Beckman Coulter). Assays of the fermentation products (acetate and ethanol) were carried out according to previously described methods (16). In brief, samples were taken at appropriate time intervals and then centrifuged at 7,000 × *g* for 10 min at 4°C. The supernatant was then analyzed. The concentrations of acetate and ethanol were determined using a 7890A gas chromatograph (Agilent, Wilmington, DE, USA) equipped with a flame ionization detector (Agilent) and a capillary column (EC-Wax; Alltech).
