# Supplementary figures and images for: Interactive Regulation of Formate Dehydrogenase during CO2 Fixation in Gas-Fermenting Bacteria
Source: mBio. 2020 Aug 18;11(4):e00650-20. doi: 10.1128/mBio.00650-20 (PMC7439476; doi:10.1128/mBio.00650-20)

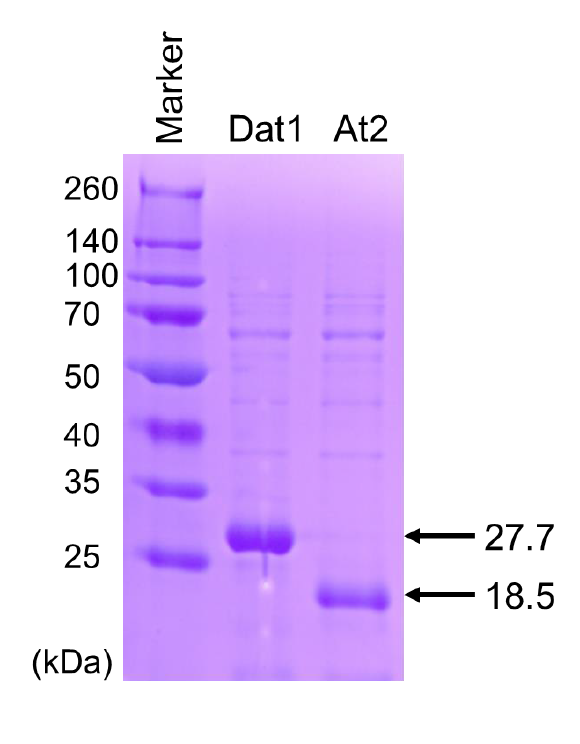

Supplement: FIG S3 [file mBio.00650-20-sf003.tif]

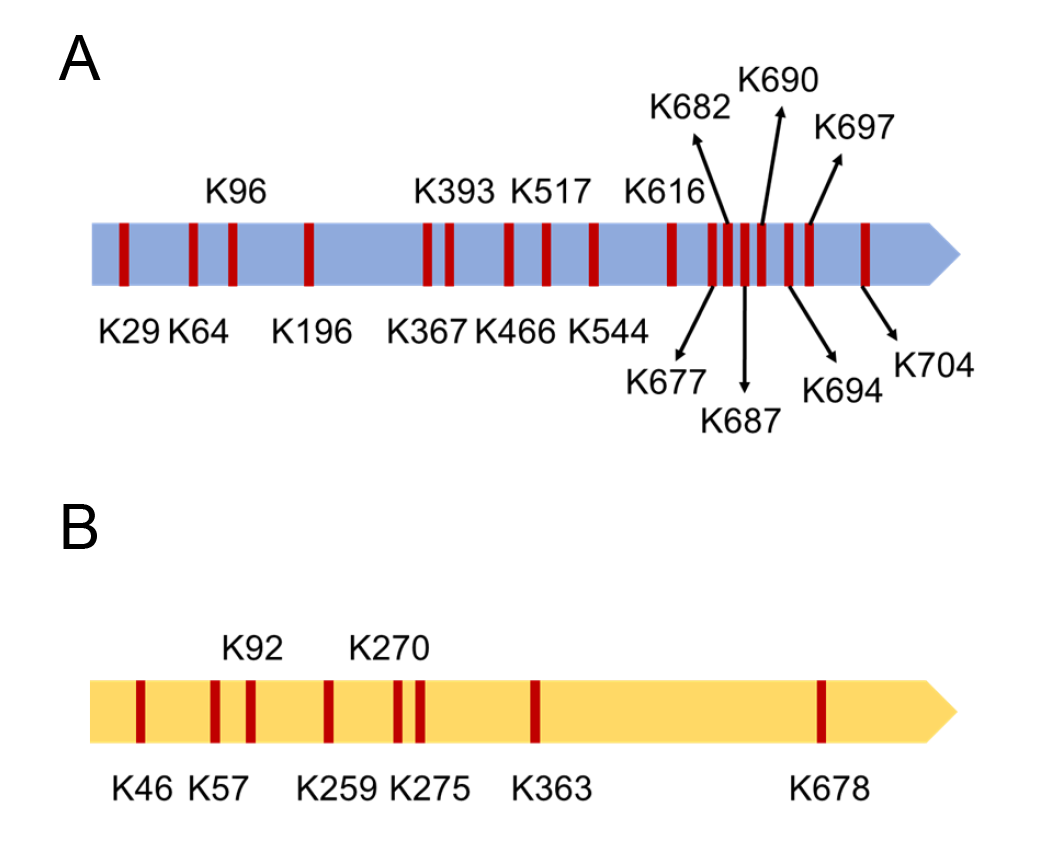

Supplement: FIG S4 [file mBio.00650-20-sf004.tif]

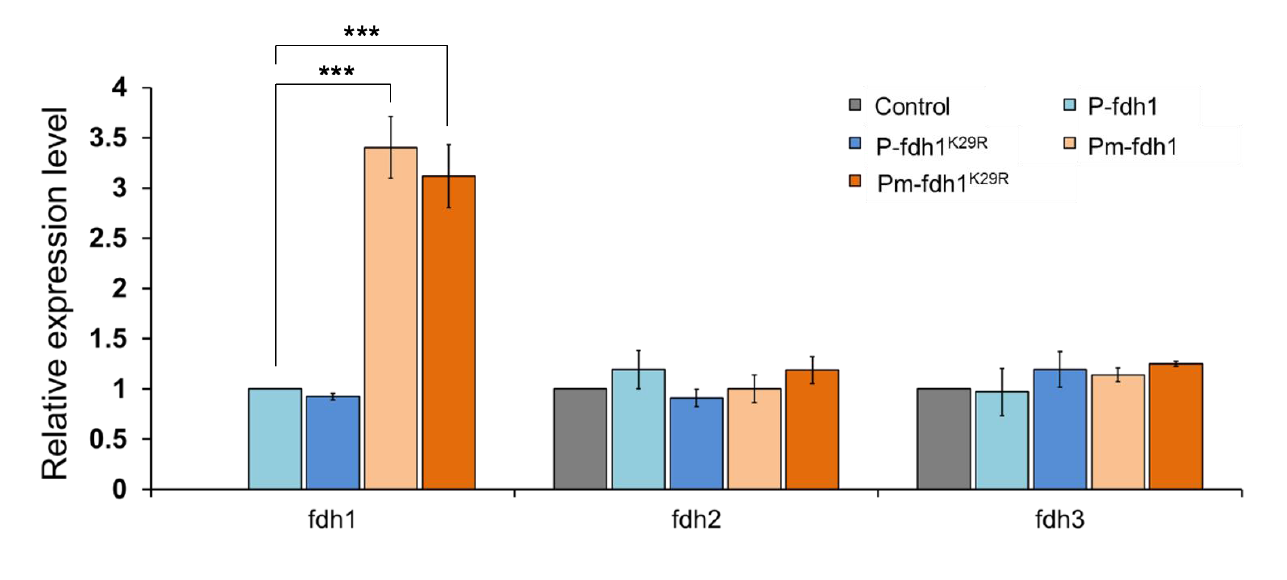

Supplement: FIG S6 [file mBio.00650-20-sf006.tif]

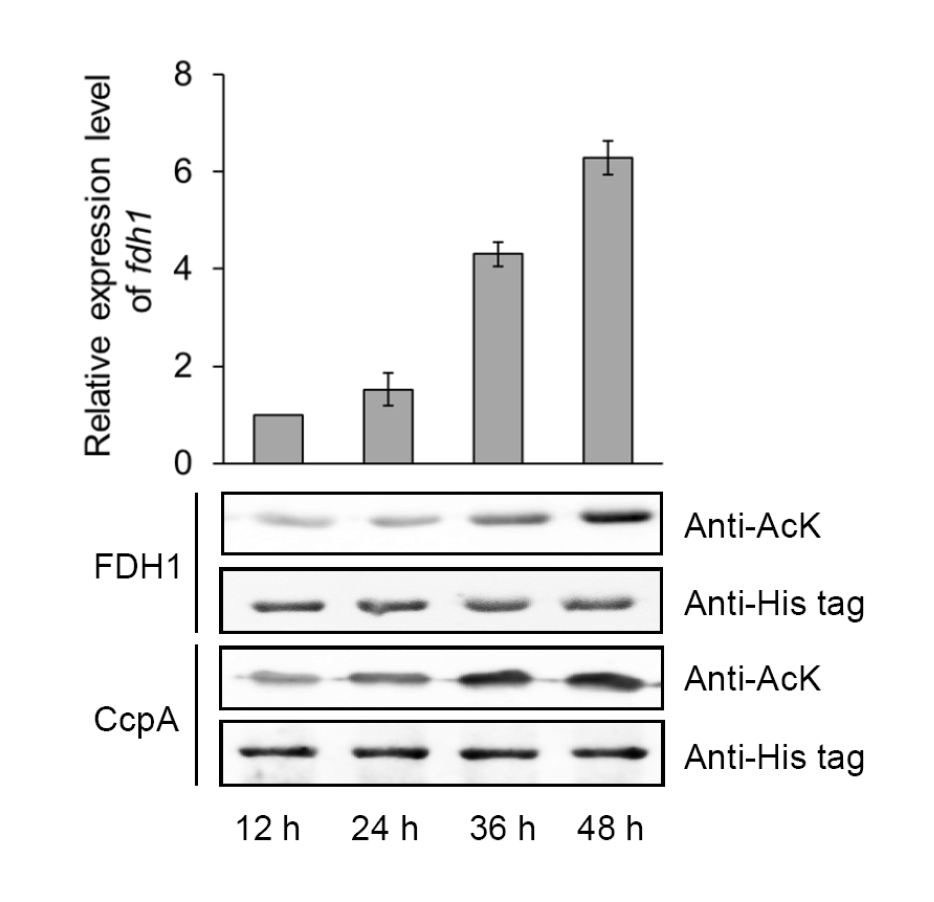

Supplement: FIG S7 [file mBio.00650-20-sf007.tif]
