# Supplementary material for: Interactive Regulation of Formate Dehydrogenase during CO2 Fixation in Gas-Fermenting Bacteria
Source: mBio. 2020 Aug 18;11(4):e00650-20. doi: 10.1128/mBio.00650-20 (PMC7439476; doi:10.1128/mBio.00650-20)
Supplement: TABLE S1 [file mBio.00650-20-st001.pdf]

Table S1. List of all *Clostridium ljungdahlii* lysine acetylated sites identified by LC-MS/MS

Proteins	Amino acid	Sequence window	Modified sequence	Position	Mass error [ppm]	Score	Intensity GAS1	Intensity GAS2
>WP_007061146.1 MULTISPECIES: 30S ribosomal protein S21	K	RAGVLSEVRKREHYEKPSVKRKKKSEAARKR	_EHYEK(ac)PSVK_	5	-0.23081	54.608	3691300	3238600
>WP_011988802.1 MULTISPECIES: 30S ribosomal protein S10	K	HTILDQSSEKIVETAKSTGAKVAGPVPLPTE	_IVETAK(ac)STGAK_	6	0.3928	80.979	26990000	20189000
>WP_011988802.1 MULTISPECIES: 30S ribosomal protein S10	K	RLKAFDHTILDQSSEKIVETAKSTGAKVAGP	_AFDHTILDQSSEK(ac)IVETAK_	13	-1.9913	725.37	5275900	12146000
>WP_013236715.1 MULTISPECIES: chromosomal replication initiator protein DnaA	K	LKQDESLQNAIGDLTKRLNQN	_QDESLQNAIGDLTK(ac)R_	14	-0.30695	38.028	0	4497600
>WP_013236715.1 MULTISPECIES: chromosomal replication initiator protein DnaA	K	YVSSEKFTNELINSIKDDKNVEFRNKYRNID	_FTNELINSIK(ac)DDK_	10	0.24769	65.092	11777000	6199700
>WP_013236715.1 MULTISPECIES: chromosomal replication initiator protein DnaA	K	ILSVPNDLTKGILTSKYKNLIANALKLITSK	_GILTSK(ac)YK_	6	0.0799	83.265	23692000	24284000
>WP_013236721.1 MULTISPECIES: DNA gyrase subunit A	K	RDESDRDGMRIVIELKRDANPNVTLNQLYKH	_IVIELK(ac)R_	6	0.38254	126.54	10387000	12429000
>WP_013236723.1 MULTISPECIES: transcription repressor NadR	K	VIAVSHETNEIEDELKTIVKFGGKVQDVIVE	_AVIAVSHETNEIEDELK(ac)TIVK_	17	1.0842	43.534	10567000	0
>WP_013236723.1 MULTISPECIES: transcription repressor NadR	K	LEKNDKPQKGHILAEKLGVTRQIIVKDIAIL	_GHILAEK(ac)LGVTR_	7	0.48356	107.09	14523000	8291500
>WP_013236725.1 MULTISPECIES: FAD/NAD(P)-binding oxidoreductase	K	IESISKGFKVVTNKNKFTCNMVLNTTPGKNY	_NK(ac)FTCNMVLNTTPGK_	2	0.23496	30.961	6652100	7507300
>WP_013236727.1 MULTISPECIES: serine--tRNA ligase	K	YDELEKLTNDAEDVLKGLKIPYRVVRICKGD	_LTNDAEDVLK(ac)GLK_	10	-0.46458	80.522	12388000	7498900
>WP_013236727.1 MULTISPECIES: serine--tRNA ligase	K	LDEKRRKILVEVEALKNKRNQDSAQIAKMKR	_ILVEVEALK(ac)NKR_	9	0.23611	186.72	19477000	15697000
>WP_013236727.1 MULTISPECIES: serine--tRNA ligase	K	MVKFTKPEESYDELEKLTNDAEDVLKGLKIP	_FTKPEESYDELEK(ac)LTNDAEDVLK_	13	-0.3044	119.06	66047000	49058000
>WP_013236727.1 MULTISPECIES: serine--tRNA ligase	K	YMVNRTSMTGTGQLPKFEEDAFRISNNDYFL	_TSM(ox)TGTGQLPK(ac)FEEDAFR_	11	0.41074	209.67	85265000	95960000
>WP_013236727.1 MULTISPECIES: serine--tRNA ligase	K	EALKNKRNQDSAQIAKMKRNGENADDLVAEM	_RNQDSAQIAK(ac)MK_	10	-0.5293	128.01	150610000	113450000
>WP_013236727.1 MULTISPECIES: serine--tRNA ligase	K	DFDPATIDEVIALDEKRRKILVEVEALKNKR	_GEDFDPATIDEVIALDEK(ac)R_	18	-0.07437	162.15	318930000	213720000
>WP_013236727.1 MULTISPECIES: serine--tRNA ligase	K	EKRRKILVEVEALKNKRNQDSAQIAKMKRNG	_ILVEVEALK(ac)NKR_	11	-5.3457	727.44	12008000	9200400
>WP_013236761.1 MULTISPECIES: toxin Fic	K	TISNKVAKELAEGEYKKFRIRQDREYESDFD	_ELAEGEYK(ac)K_	8	1.0185	55.567	0	2791200
>WP_013236767.1 MULTISPECIES: dihydrodipicolinate synthase family protein	K	TGHDLSPELTLNLLRKHSNIVGYKDTVSEMG	_K(ac)HSNIVGYKDTVSEMGHTR_	1	-0.55185	31.462	11291000	20475000
>WP_013236767.1 MULTISPECIES: dihydrodipicolinate synthase family protein	K	LTLNLLRKHSNIVGYKDTVSEMGHTRKLIQT	_HSNIVGYK(ac)DTVSEMGHTR_	8	-0.27711	142.78	77504000	84429000
>WP_013236772.1 MULTISPECIES: Cof-type HAD-IIB family hydrolase	K	VDVFKVLLISDTEDVKLLKDYFSKYKSLTAV	_VLLISDTEDVK(ac)LLK_	11	0.96015	69.602	0	4614200
>WP_013236772.1 MULTISPECIES: Cof-type HAD-IIB family hydrolase	K	LLDIMASNICKGSALKILSKKFRMDLDKVIV	_GSALK(ac)ILSK_	5	-0.84175	85.807	7961300	17080000
>WP_013236772.1 MULTISPECIES: Cof-type HAD-IIB family hydrolase	K	LVYTNNMVYSSKKNYKVENYESINKSAPPSL	_NYK(ac)VENYESINK_	3	0.10478	135.6	123670000	107890000
>WP_013236773.1 ketose-bisphosphate aldolase	K	NVYNYETIKGVIQAIKNQKTPAVIAFGEKYL	_GVIQAIK(ac)NQK_	7	-0.29547	138.06	38266000	40643000
>WP_013236773.1 ketose-bisphosphate aldolase	K	AIKNQKTPAVIAFGEKYLKNMDVDSVYNLVN	_TPAVIAFGEK(ac)YLK_	10	0.32612	128.54	166760000	115530000
>WP_013236774.1 hydroxyacid dehydrogenase/reductase-like protein	K	DLGENKKEEELKLSVKISDAVTSIVEKLNVR	_LSVK(ac)ISDAVTSIVEK_	4	0.69099	37.092	1778300	10080000
>WP_013236774.1 hydroxyacid dehydrogenase/reductase-like protein	K	SIVEKLNVRPNYIVAKGGITSSDVGTKGLKV	_LNVRPNYIVAK(ac)GGITSSDVGTK_	11	0.44564	102.6	1863100	17375000

>WP_013236774.1 hydroxyacid dehydrogenase/reductase-like protein	K	GGVSDKNLLSRDELIKVDSK NGGLLIVGSHV	_DELIK(ac)VDSK_	5	-0.73974	70.1	4962100	0
>WP_013236774.1 hydroxyacid dehydrogenase/reductase-like protein	K	TFGYTKSHLGEYIEEKTKGTF KAKDTTYISL	_SHLGEYIEEK(ac)TK_	10	0.62301	53.034	6016100	7207200
>WP_013236774.1 hydroxyacid dehydrogenase/reductase-like protein	K	VDSKNGGLLIVGSHVKKTTE QLEELKKCPFI	_NGGLLIVGSHVK(ac)K_	12	0.83861	131.32	20213000	28728000
>WP_013236774.1 hydroxyacid dehydrogenase/reductase-like protein	K	AIDYVDVKIFATALVKAIKSG KNFMYRSAAA	_IFATALVK(ac)AIK_	8	0.52807	94.616	45144000	40758000
>WP_013236774.1 hydroxyacid dehydrogenase/reductase-like protein	K	DSTLRGHYPLETEILKQTVEA NSDVKFDGEV	_GHYPLETEILK(ac)QTVEANSDVK_	11	0.47463	133.93	57603000	37891000
>WP_013236774.1 hydroxyacid dehydrogenase/reductase-like protein	K	FILTNSRGFTAAETEKAHKEI SLNISKVAKK	_GFTAAETEK(ac)AHK_	9	-2.3409	121.68	112930000	97406000
>WP_013236774.1 hydroxyacid dehydrogenase/reductase-like protein	K	KLSVKISDAVTSIVEKLNVRP NYIVAKGGIT	_ISDAVTSIVEK(ac)LNVRPNYIVAK_	11	0.34668	82.586	121280000	46837000
>WP_013236774.1 hydroxyacid dehydrogenase/reductase-like protein	K	RKRIDLGENKKEEELKLSVKI SDAVTSIVEK	_KEEELK(ac)LSVK_	6	-0.1269	193.73	136410000	93522000
>WP_013236774.1 hydroxyacid dehydrogenase/reductase-like protein	K	EKFEAEIQRIIEKTEKLIASGK TVAVYTSRK	_TEK(ac)LIASGK_	3	-0.25702	112.3	209450000	226320000
>WP_013236774.1 hydroxyacid dehydrogenase/reductase-like protein	K	VGSHVKKTTEQLEELKKCPFI EFIEFNCHLV	_TTEQLEELK(ac)K_	9	-0.72987	230.59	1.119E+09	1.064E+09
>WP_013236774.1 hydroxyacid dehydrogenase/reductase-like protein	K	SDSEKFEAEIQRIIEKTEKLIA SGKTVAVYT	_IIEK(ac)TEK(ac)LIASGK_	4	0.76733	117.93	1.154E+09	587650000
>WP_013236774.1 hydroxyacid dehydrogenase/reductase-like protein	K	DSKNGGLLIVGSHVKKTTEQL EELKKCPFIE	_K(ac)TTEQLEELK(ac)K_	1	-0.17729	715.89	13139000	1816600
>WP_013236775.1 2-hydroxy-3-oxopropionate reductase	K	NAIRGGLAGSAVLDQKVPRIL DRNFVPGGKI	_GGLAGSAVLDQK(ac)VPR_	12	0.05344	189.5	113130000	83280000
>WP_013236775.1 2-hydroxy-3-oxopropionate reductase	K	SISPVVSKKIACEVNKKDIEM LDAPVSGGET	_K(ac)IACEVNK(ac)K_	8	0.14013	126.28	149040000	84047000
>WP_013236775.1 2-hydroxy-3-oxopropionate reductase	K	GGIVQFYEKIANVQVKRS	_IANVQVK(ac)R_	7	-0.20102	173.88	613670000	531520000
>WP_013236775.1 2-hydroxy-3-oxopropionate reductase	K	GSVIIDMSSISPVVSKKIACE VNKKDIEMLD	_KGSVIIDMSSISPVVSK(ac)K_	17	-0.79973	245.78	877510000	956660000
>WP_013236775.1 2-hydroxy-3-oxopropionate reductase	K	SVIIDMSSISPVVSKKIACEV NKKDIEMLDA	_K(ac)IACEVNK(ac)K_	1	0.14013	587.32	30824000	13478000
>WP_013236787.1 MULTISPECIES: DNA polymerase III subunit gamma/tau	K	KKIVDTYRESNTEEHKTKNN SSNDEVQTANK	_ESNTEEHK(ac)TK_	8	0.57145	60.434	2537000	4574600
>WP_013236788.1 MULTISPECIES: YbaB/EbfC family nucleoid-associated protein	K	DLILAACNEALKNAEKETAS EMQKVTGGLNI	_NAEK(ac)ETASEMQK_	4	-0.57432	76.522	7218200	6424300
>WP_013236788.1 MULTISPECIES: YbaB/EbfC family nucleoid-associated protein	K	FGGGGNMNNLMKQAQKLQK QMETMQSELQNK	_QAQK(ac)LQK_	4	-0.386	113.85	21624000	24200000
>WP_013236789.1 MULTISPECIES: recombination protein RecR	K	NVEGEATAMYISKILKHLGVK VTRIAHGIPV	_ILK(ac)HLGVK_	3	-0.51309	66.429	3732700	0
>WP_013236793.1 MULTISPECIES: NUDIX domain-containing protein	K	RIVRKVLKELGYTEEKAYRC DNNNCQTDS	_ELGYTEEK(ac)AYR_	8	-0.06852	89.548	4784200	5233100
>WP_013236798.1 MULTISPECIES: alanine--glyoxylate aminotransferase family protein	K	TNIKNTRDIDVAELNKKLGER GFQISNGYGK	_DIDVAELNK(ac)K(ac)LGER_	9	-0.09686	82.261	5533400	0
>WP_013236798.1 MULTISPECIES: alanine--glyoxylate aminotransferase family protein	K	NIKNTRDIDVAELNKKLGERG FQISNGYGKL	_DIDVAELNK(ac)K(ac)LGER_	10	-0.09686	75.462	5533400	0
>WP_013236798.1 MULTISPECIES: alanine--glyoxylate aminotransferase family protein	K	KKLGERGFQISNGYGKLKGK AFRIAHMADCT	_GFQISNGYGK(ac)LK_	10	1.1826	100.72	16029000	43030000
>WP_013236798.1 MULTISPECIES: alanine--glyoxylate aminotransferase family protein	K	LFADERYLSNTLTNIKNTRDID VAELNKKLG	_YLSNTLTNIK(ac)NTR_	10	0.13116	200.44	98223000	83175000
>WP_013236799.1 3-phosphoglycerate dehydrogenase	K	GVFVINCARGGVIDEKVLAKA LNTGKVAGAA	_GGVIDEK(ac)VLAK_	7	-0.21307	37.995	10851000	0
>WP_013236800.1 DUF1015 domain-containing protein	K	SLYIADGHHRTASAVKVGIKR RKENPDYKGD	_TASAVK(ac)VGIK_	6	-0.07023	64.297	7030400	6194000
>WP_013236800.1 DUF1015 domain-containing protein	K	LNGLTRDEYMNKISEKFNVK EYEGKDPYRPS	_ISEK(ac)FNVK_	4	0.95572	123.69	39354000	21027000

>WP_013236800.1 DUF1015 domain-containing protein	K	RIDFVGGIRGLNELEKRVDKEREGVAFSMYP	_GLNELEK(ac)R_	7	0.22728	131.06	60606000	65843000
>WP_013236800.1 DUF1015 domain-containing protein	K	DANTGPIFLTYRYNEKINDIVDKWTNEKKPV	_YNEK(ac)INDIVDK_	4	-0.61327	122.33	100100000	72612000
>WP_013236801.1 MULTISPECIES: N-acetyltransferase	K	ESVLNYYIKNKDYLSKFEPDREKNFYTLDIQ	_DYLSK(ac)FEPDREK_	5	-0.33523	94.191	10310000	18019000
>WP_013236806.1 MULTISPECIES: ABC transporter permease	K	KYKVGDKINLSIAVDKNGKQTESIKKQFTVA	_INLSIAVDK(ac)NGK_	9	-0.05763	58.172	30100000	29274000
>WP_013236809.1 MULTISPECIES: hydrolase	K	LIMHKDIKEMLENCAKRSEINLQYSISTGKS	_EMLENCAK(ac)R_	8	-0.67212	76.064	3804600	3276000
>WP_013236809.1 MULTISPECIES: hydrolase	K	IDDDGMIRVDNIGNFKKEDISHSFIRFDNGT	_VDNIGNFK(ac)K_	8	0.42905	107.57	17216000	20647000
>WP_013236811.1 MULTISPECIES: aminotransferase class V-fold PLP-dependent enzyme	K	EIDVDELKKFLDKDSKFKYATVVHCDTPSGM	_FLDKDSK(ac)FK_	7	0.79125	106.2	13344000	24986000
>WP_013236812.1 MULTISPECIES: peptidylprolyl isomerase	K	FFELAYSYAKDENLEKDDEYLKMLESAKKEI	_DENLEK(ac)DDEYLK_	6	0.01531	57.802	2911600	0
>WP_013236812.1 MULTISPECIES: peptidylprolyl isomerase	K	PERLKAKHILVDSIEKAKKISKEISEGMPFE	_HILVDSIEK(ac)AK_	9	0.14054	120.86	43346000	36112000
>WP_013236814.1 MULTISPECIES: L-arabinose isomerase	K	NKVSQKDIDDTYEEFKKIYIMDVGENDPKFY	_DIDDTYEEFK(ac)K_	10	-0.72103	101.39	17117000	11877000
>WP_013236814.1 MULTISPECIES: L-arabinose isomerase	K	PIVFKSLATSASQITKLMKEVNYNDNVAGVI	_SLATSASQITK(ac)LMK_	11	-0.57611	84.566	18696000	12668000
>WP_013236818.1 MULTISPECIES: alpha-N-arabinofuranosidase	K	YPFSMVSNYGRGTVLKPSVNGASYKCDFGEL	_GTVLK(ac)PSVNGASYK_	5	-3.7124	53.001	3427100	4688800
>WP_013236818.1 MULTISPECIES: alpha-N-arabinofuranosidase	K	KADEDGFRTDAMEVIKDLKLGLVRYPGGNFV	_TDAMEVIK(ac)DLK_	8	0.28344	60.49	3440500	2268900
>WP_013236818.1 MULTISPECIES: alpha-N-arabinofuranosidase	K	LGRAVYSGIYEPGHEKADEDGFRTDAMEVIK	_AVYSGIYEPGHEK(ac)ADEDGFR_	13	0.44431	37.624	15938000	3417300
>WP_013236818.1 MULTISPECIES: alpha-N-arabinofuranosidase	K	EDGFRTDAMEVIKDLKLGLVRYPGGNFVSNY	_DLK(ac)LGLVR_	3	0.04428	127.12	25323000	31484000
>WP_013236819.1 MULTISPECIES: MFS transporter	K	MDMKVQDKDVFKENLKFSEKFGYGCGDLAIN	_ENLK(ac)FSEK_	4	-0.28681	69.812	22039000	23906000
>WP_013236819.1 MULTISPECIES: MFS transporter	K	MDMKVQDKDVFKENLKFSEKFGYGCGD	_VQDKDVFK(ac)ENLK_	8	0.53109	115.57	32433000	39354000
>WP_013236825.1 MULTISPECIES: LD-carboxypeptidase	K	KKGDTIGLVSPASAEKPEKIKGKIEFLKDFG	_KGDTIGLVSPASAEK(ac)PEK_	15	-0.05828	52.814	8418300	2447200
>WP_013236826.1 MULTISPECIES: magnesium transporter CorA family protein	K	STSLKANEMTLEKMLKLELLQKYPEDQDILE	_MLK(ac)LELLQK_	3	-0.21955	70.1	4997000	6348300
>WP_013236829.1 MULTISPECIES: thymidylate kinase	K	MPPEYSKKLMTKRNNKFTGNAEKDIHERDYE	_NNK(ac)FTGNAEK_	3	-0.44809	50.452	12350000	12272000
>WP_013236830.1 MULTISPECIES: hypothetical protein	K	TDLLEILTEGGFRVTKLATTGGFLRAGNTTL	_VTK(ac)LATTGGFLR_	3	0.79805	105.65	22318000	15057000
>WP_013236838.1 MULTISPECIES: 16S rRNA (cytidine(1402)-2'-O)-methyltransferase	K	CHKYNENEKSKLLIQKLKDGKDIALVSDAGT	_LLIQK(ac)LK_	5	0.28034	92.692	22908000	12785000
>WP_013236841.1 MULTISPECIES: AbrB/MazE/SpoVT family DNA-binding domain-containing protein	K	KDALEIYVDGEQIILKKYEPACIFCGDARDV	_DALEIYVDGEQIILK(ac)K_	15	-4.1022	70.281	5771700	5505500
>WP_013236841.1 MULTISPECIES: AbrB/MazE/SpoVT family DNA-binding domain-containing protein	K	NYRGKNICKSCLNELKEGK	_SCLNELK(ac)EGK_	7	-0.28553	69.672	26142000	26817000
>WP_013236844.1 MULTISPECIES: PspA/IM30 family protein	K	EFKKLDEVDLDKELEKYKQN	_LDEVDLDKELEK(ac)YK_	12	1.4427	62.042	4897000	3754300
>WP_013236844.1 MULTISPECIES: PspA/IM30 family protein	K	LDQKLRDMDDSLNKAKLSSAQILGNVHEIEK	_AK(ac)LSSAQILGNVHEIEK(ac)K_	2	-0.21534	58.427	9988500	21564000
>WP_013236844.1 MULTISPECIES: PspA/IM30 family protein	K	KLEADKSYSSLEASYKDACQKAEAVKSKLKE	_SYSSLEASYK(ac)DACQK_	10	0.16792	84.566	13550000	9385100
>WP_013236844.1 MULTISPECIES: PspA/IM30 family protein	K	KKMEESKRVSTEFDEKVKLAMSKGNEELAKK	_VSTEFDEK(ac)VK_	8	-0.23708	76.143	22797000	10189000
>WP_013236844.1 MULTISPECIES: PspA/IM30 family protein	K	LAEGLGDLREEDSLEKEFKKLDEVDLDKELE	_EEDSLEK(ac)EFK_	7	0.19926	111.06	24057000	17033000

>WP_013236844.1 MULTISPECIES: PspA/IM30 family protein	K	YRDEAAARFNNAEASKKVNEILSNVETGSNK	_FNNAEASK(ac)K_	8	-0.00701	87.258	33171000	39831000
>WP_013236844.1 MULTISPECIES: PspA/IM30 family protein	K	EKVKLAMSKGNEELAKKALARKLEADKSYSS	_GNEELAK(ac)K_	7	-0.06457	81.548	52714000	44378000
>WP_013236844.1 MULTISPECIES: PspA/IM30 family protein	K	NTLDDIENPIELLDQKLRDMDDSLNKAKLSS	_VNNTLDDIENPIELLDQK(ac)LR_	18	2.9274	245.95	121250000	136470000
>WP_013236844.1 MULTISPECIES: PspA/IM30 family protein	K	EAVKSKLKELEEEIHKTRVYRDEAAARFNNA	_LKELEEEIHK(ac)TR_	10	0.1508	225.67	225080000	184830000
>WP_013236844.1 MULTISPECIES: PspA/IM30 family protein	K	KLSSAQILGNVHEIEKKMEESKRVSTEFDEK	_LSSAQILGNVHEIEK(ac)K_	15	-0.11476	196.45	589880000	398610000
>WP_013236844.1 MULTISPECIES: PspA/IM30 family protein	K	ELLDQKLRDMDDSLNKAKLSSAQILGNVHEI	_DMDDSLNK(ac)AK_	8	-0.16807	589.77	2046200	13579000
>WP_013236846.1 MULTISPECIES: DUF1836 domain-containing protein	K	FNEQKFQDMVKDMDLKSDDEERIGIFFVVMS	_DMDLK(ac)SDDEER_	5	-0.73304	64.55	1997500	2992200
>WP_013236853.1 MULTISPECIES: hypothetical protein	K	FSSNEVIKVWREAFQKFKTKKGARSSIEALL	_EAFQK(ac)FK_	5	-0.23197	76.847	6764700	0
>WP_013236853.1 MULTISPECIES: hypothetical protein	K	FKTKKGARSSIEALLKRVDKGTHIGTINPLV	_SSIEALLK(ac)R_	8	-1.1859	92.062	7327700	5452300
>WP_013236863.1 MULTISPECIES: methionine--tRNA ligase	K	NKLKEEKKAAQNSNEKSLKKQITIDDFDKID	_AAQNSNEK(ac)SLK_	8	0.34011	50.354	3591500	4073400
>WP_013236863.1 MULTISPECIES: methionine--tRNA ligase	K	QIDDELIKLALETPEKVEKNMNDLRIPEALD	_LALETPEK(ac)VEK_	8	-0.06352	52.391	5431100	1950900
>WP_013236863.1 MULTISPECIES: methionine--tRNA ligase	K	EAYFFKMSKYAPKLIKYIEDHPDFIQPESRK	_LIK(ac)YIEDHPDFIQPESR_	3	0.44396	47.916	9510800	0
>WP_013236863.1 MULTISPECIES: methionine--tRNA ligase	K	LFPRIDVDKKIEELNKLKEEKKAAQNSNEKS	_IEELNK(ac)LK_	6	-0.41089	82.452	23717000	26047000
>WP_013236863.1 MULTISPECIES: methionine--tRNA ligase	K	IPFGSDGLFNNEIFIKRINADLANDLGNLLS	_EIPFGSDGLFNNEIFIK(ac)R_	17	0.85105	119.4	26914000	18639000
>WP_013236871.1 MULTISPECIES: ATP-binding protein	K	ISKSLEYNDLLNNFIKKLK	_SLEYNDLLNNFIK(ac)K_	13	-0.03991	34.179	4587000	0
>WP_013236871.1 MULTISPECIES: ATP-binding protein	K	KCSKEDKGGCKYILPKYGNVKHIIGIMSGKG	_YILPK(ac)YGNVK_	5	0.55886	45.161	7112500	7509400
>WP_013236871.1 MULTISPECIES: ATP-binding protein	K	DKGGCKYILPKYGNVKHIIGIMSGKGGVGKS	_YGNVK(ac)HIIGIMSGK_	5	-0.27889	779.92	6306800	9509300
>WP_013236874.1 anaerobic ribonucleoside-triphosphate reductase	K	AIFVEPTRKEILKNLKQMGVGEEKIKDISEK	_NLK(ac)QMGVGEEK_	3	-1.4751	101.93	33734000	4304000
>WP_013236879.1 isoleucine--tRNA ligase	K	KWPEYDSSAVDDKLEKDMEEAYKIVKLGRSA	_WPEYDSSAVDDKLEK(ac)DMEEAYK_	15	-0.05855	41.115	7579400	6429000
>WP_013236879.1 isoleucine--tRNA ligase	K	EVEKSLGISGKPQIEKYGVEDFIKKCKNSVF	_SLGISGKPQIEK(ac)YGVEDFIK_	12	-1.4778	44.587	7813000	0
>WP_013236879.1 isoleucine--tRNA ligase	K	IDSDKTFVQMEKDVLKLWQDKKVVEKSFKEN	_DVLK(ac)LWQDK_	4	-1.2737	72.2	11260000	9476900
>WP_013236879.1 isoleucine--tRNA ligase	K	SNWYVRRNRSRFWSQKLTDDKIGAYLTLYKV	_FWSQK(ac)LTDDK_	5	-0.08266	132.78	56687000	66180000
>WP_013236880.1 MULTISPECIES: LacI family transcriptional regulator	K	VKAKDGYKGMSEILSKTKVDSVFCTSDEVAM	_GM(ox)SEILSK(ac)TK_	8	3.542	107.83	6363200	6616300
>WP_013236880.1 MULTISPECIES: LacI family transcriptional regulator	K	QPTYDMGSVGMRMLIKLINKQELEKKNYVLS	_MLIK(ac)LINK_	4	-0.93308	87.754	40997000	0
>WP_013236880.1 MULTISPECIES: LacI family transcriptional regulator	K	VRYKGYRKVLEENNLKLDKSKVYFGNVKAKD	_VLEENNLK(ac)LDK_	8	-0.20687	122.51	47310000	45131000
>WP_013236880.1 MULTISPECIES: LacI family transcriptional regulator	K	VVNEETKKKVLTAIEKLGYRPNIVARSLKTQ	_GYRPNIVARSLK(ac)TQ_	12	-0.04353	96.993	77875000	50174000
>WP_013236880.1 MULTISPECIES: LacI family transcriptional regulator	K	INKQELEKKNYVLSHKLIERDSCKK	_NYVLSHK(ac)LIER_	7	0.20308	159.99	79882000	65024000
>WP_013236886.1 MULTISPECIES: TIGR00299 family protein	K	EISKGIFKKVAQAEAKVHSKPINEVHFHEVG	_KVAQAEAK(ac)VHSK_	8	0.71599	57.802	5677400	0
>WP_013236901.1 MULTISPECIES: 4-(cytidine 5'-diphospho)-2-C-methyl-D-erythritol kinase	K	VKAYAKINLSLDVVGKRKDGYHLLKMIMQNI	_INLSLDVVGK(ac)R_	10	0.29255	48.004	5086900	15766000
>WP_013236901.1 MULTISPECIES: 4-(cytidine 5'-diphospho)-2-C-methyl-D-erythritol kinase	K	MLSKNMKNVLENVTLKKYPVLRKIKTDLIDF	_NVLENVTLK(ac)K_	9	-0.04784	91.855	10908000	8313700

>WP_013236910.1 MULTISPECIES: CTP synthase	K	VPLMLHKEGLDELVCKKLKLECSEIDNSEWI	_EGLDELVCK(ac)K_	9	-0.18964	74.162	0	8363700
>WP_013236910.1 MULTISPECIES: CTP synthase	K	YPVIDLMPDQKDIDEKGGTMRLGIYPCKLLK	_DIDEK(ac)GGTMR_	5	0.26232	90.827	16834000	12750000
>WP_013236910.1 MULTISPECIES: CTP synthase	K	LGYDGAHSSEIDPDTKYPVIDLMPDQKDIDE	_NVLGYDGAHSSEIDPDTK(ac)YPVIDLMPDQK_	18	0.62992	52.391	32432000	33982000
>WP_013236910.1 MULTISPECIES: CTP synthase	K	LDLGHYERFIDENLSKNNNVTTGKIYWSVIS	_FIDENLSK(ac)NNNVTTGK_	8	0.12382	150.59	58550000	68519000
>WP_013236916.1 MULTISPECIES: peptide chain release factor 1	K	EETDREMKEMEQEEIKRLTEVIEESENDLRV	_EMEQEEIK(ac)R_	8	0.49215	92.062	5340000	5284600
>WP_013236916.1 MULTISPECIES: peptide chain release factor 1	K	ITHLPTGLVVSCQDEKSQLKNKEKGMKVLKS	_ITHLPTGLVVSCQDEK(ac)SQLK_	16	0.11757	67.995	13280000	4960200
>WP_013236919.1 MULTISPECIES: threonylcarbamoyl-AMP synthase ;>WP_013239343.1 MULTISPECIES: threonylcarbamoyl-AMP synthase	K	MKKPDKNLRPKAPGMKYRHYAPKAPLKIVKG	_APGMK(ac)YR_	5	-0.03025	83.287	11681000	8824600
>WP_013236922.1 MULTISPECIES: uracil phosphoribosyltransferase	K	IPILRAGLGMVGGMIKLIPAAKVGHIGLYRD	_AGLGMVGGMIK(ac)LIPAAK_	11	0.79991	64.394	12177000	0
>WP_013236925.1 MULTISPECIES: UDP-N-acetylglucosamine 2-epimerase (non-hydrolyzing)	K	AGTVRLVGTDEEKIIKLANELIRNPKEYDVM	_IIK(ac)LANELIR_	3	-0.28633	134.68	219630000	281660000
>WP_013236929.1 MULTISPECIES: ATP synthase F0 subunit B	K	PVNNTITNRQQEIDNKIRTADENEKKSKQLV	_QQEIDNK(ac)IR_	7	-0.32615	145.17	25581000	27340000
>WP_013236929.1 MULTISPECIES: ATP synthase F0 subunit B	K	LKNSKQEGKAIVEDYKNKADKVSENIVNDAQ	_AIVEDYK(ac)NK_	7	-0.14324	101.64	65263000	52761000
>WP_013236929.1 MULTISPECIES: ATP synthase F0 subunit B	K	EKKSKQLVTQHQELLKNSKQEGKAIVEDYKN	_QLVTQHQELLK(ac)NSK_	11	-0.35474	218.16	116580000	86767000
>WP_013236929.1 MULTISPECIES: ATP synthase F0 subunit B	K	QQEIDNKIRTADENEKKSKQLVTQHQELLKN	_TADENEK(ac)K_	7	-2.4966	85.666	276220000	45056000
>WP_013236930.1 MULTISPECIES: F0F1 ATP synthase subunit delta	K	LLVLIEKDRINEIDGKLREMENIYLESNNTV	_INEIDGK(ac)LR_	7	-2.2546	74.267	0	4255600
>WP_013236930.1 MULTISPECIES: F0F1 ATP synthase subunit delta	K	EVSTSEKKKMFTEIFKDKVNEDILSFLLVLI	_MFTEIFK(ac)DK_	7	0.23738	62.303	11340000	9917800
>WP_013236930.1 MULTISPECIES: F0F1 ATP synthase subunit delta	K	YVEVNNEVIDGSIRSKLSEMKKIMLKGEQR_	_SK(ac)LSEMK_	2	-0.38627	95.531	39019000	26031000
>WP_013236930.1 MULTISPECIES: F0F1 ATP synthase subunit delta	K	TVIALNDDERNTLIEKLEKKFNKKVLIEEEI	_NTLIEK(ac)LEK_	6	0.07105	98.048	55548000	35750000
>WP_013236930.1 MULTISPECIES: F0F1 ATP synthase subunit delta	K	LEELRQVVAAIKGNSKFLEIMEHPEVSTSEK	_GNSK(ac)FLEIMEHPEVSTSEK_	4	-3.9055	226.57	906700000	870410000
>WP_013236931.1 MULTISPECIES: F0F1 ATP synthase subunit alpha	K	ISVSRVGGNAQIKAMKQVAGTLRLDLAQYRE	_AMK(ac)QVAGTLR_	3	-0.45477	68.893	0	5139800
>WP_013236931.1 MULTISPECIES: F0F1 ATP synthase subunit alpha	K	ASFAQFGSDLDKESMKRLEKGKRLTEILKQP	_ELASFAQFGSDLDKESMK(ac)R_	18	-0.52984	52.784	7178200	0
>WP_013236931.1 MULTISPECIES: F0F1 ATP synthase subunit alpha	K	SDELSDKLGNAIEEFKKIFLAEA	_LGNAIEEFK(ac)K_	9	0.77308	110.39	28306000	19953000
>WP_013236931.1 MULTISPECIES: F0F1 ATP synthase subunit alpha	K	DEIKNKKIISDELSDKLGNAIEEFKKIFLAE	_IISDELSDK(ac)LGNAIEEFK_	9	1.0114	116.82	29933000	16242000
>WP_013236931.1 MULTISPECIES: F0F1 ATP synthase subunit alpha	K	GDVFYLHSRLLERAAKLSDKLGGGSLTALPI	_AAK(ac)LSDK_	3	0.01416	75.243	47989000	47392000
>WP_013236931.1 MULTISPECIES: F0F1 ATP synthase subunit alpha	K	LDYMSTHHKEIGDEIKNKKIISDELSDKLGN	_EIGDEIK(ac)NKK_	7	0.10313	132.01	48416000	8665900
>WP_013236931.1 MULTISPECIES: F0F1 ATP synthase subunit alpha	K	NAGISVSRVGGNAQIKAMKQVAGTLRLDLAQ	_VGGNAQIK(ac)AMK_	8	-0.23613	140.16	96338000	74275000
>WP_013236931.1 MULTISPECIES: F0F1 ATP synthase subunit alpha	K	VLLGSQKGIKEGDTVKRTGRVVEVPVGEAIV	_EGDTVK(ac)R_	6	0.62134	79.116	102910000	65574000
>WP_013236932.1 MULTISPECIES: F0F1 ATP synthase subunit gamma	K	LVTIKRRIRSITSTQKITNAMGLIATSKLRK	_SITSTQK(ac)ITNAMGLIATSK_	7	1.1166	49.528	7966600	9253600
>WP_013236932.1 MULTISPECIES: F0F1 ATP synthase subunit gamma	K	GATKNANDLLDKLNLKYNRERQSAITQEITE	_LNLK(ac)YNR_	4	1.1781	148.28	92786000	63325000
>WP_013236933.1 MULTISPECIES: F0F1 ATP synthase subunit beta	K	QRGGKIGLFGGAGVGKTVLIQELINNIAKEH	_IGLFGGAGVGK(ac)TVLIQELINNIAK_	11	0.33446	136.14	0	4154300

>WP_013236933.1 MULTISPECIES: F0F1 ATP synthase subunit beta	K	EGNDLYYEMQESGVIKKTALVFGQMNEPPGA	_EGNDLYYEMQESGVIK(ac)K_	16	0.34632	84.14	23394000	27356000
>WP_013236934.1 MULTISPECIES: ATP synthase F1 subunit epsilon	K	AKAAKERAEKRLVSQKDGVDVKRAEMALARA	_LVSQK(ac)DGVDVK(ac)R_	5	0.59067	56.225	14036000	10696000
>WP_013236934.1 MULTISPECIES: ATP synthase F1 subunit epsilon	K	RAEKRLVSQKDGVDVKRAEMALARALARINL	_LVSQK(ac)DGVDVK(ac)R_	11	0.59067	75.189	41959000	33380000
>WP_013236940.1 MULTISPECIES: rod shape-determining protein	K	GRGVILKEPSVVAIDKIKNRVLAVGEEAWQM	_GVILKEPSVVAIDK(ac)IK_	14	-2.8587	79.022	8610300	7470800
>WP_013236940.1 MULTISPECIES: rod shape-determining protein	K	LGGIVVRSSIKVAGDKFDEAIMKYVRKEHKL	_VAGDK(ac)FDEAIMK_	5	1.2568	55.261	8659400	6261100
>WP_013236940.1 MULTISPECIES: rod shape-determining protein	K	ETVNSIADTTRAVLEKTPPELSADIADKGIF	_AVLEK(ac)TPPELSADIADK_	5	1.2693	58.159	8954700	14833000
>WP_013236940.1 MULTISPECIES: rod shape-determining protein	K	RPLKDGVISDYDVTEKMLKYFISKACGKNRI	_DGVISDYDVTEK(ac)MLK_	12	0.10291	36.638	21260000	16783000
>WP_013236943.1 MULTISPECIES: methionine adenosyltransferase	K	DIDVPWERLDKVEEIKKYM	_LDKVEEIK(ac)K_	8	-0.27174	143.96	40517000	40781000
>WP_013236943.1 MULTISPECIES: methionine adenosyltransferase	K	NNVIRPVVPREFLDYKTKYFINPTGRFVVGG	_EFLDYK(ac)TK_	6	1.53E-01	87.696	394230000	323320000
>WP_013236947.1 MULTISPECIES: ribosome-associated translation inhibitor RaiA	K	KITVKGKNIVVTDALKNAVEKKLCKLEKYFN	_NIVVTDALK(ac)NAVEK_	9	-0.53994	74.678	10605000	5491700
>WP_013236947.1 MULTISPECIES: ribosome-associated translation inhibitor RaiA	K	NSESLRFQCIPDESEKDEQEQKIVRTKKFAI	_FQCIPDESEK(ac)DEQEQK_	10	0.74773	53.828	11797000	8447500
>WP_013236947.1 MULTISPECIES: ribosome-associated translation inhibitor RaiA	K	FQCIPDESEKDEQEQKIVRTKKFAIKPMSSE	_DEQEQK(ac)IVR_	6	0.772	133.57	65462000	58300000
>WP_013236948.1 MULTISPECIES: preprotein translocase subunit SecA	K	AIESRMVTNAIEGAQKKVEGNNFDIRKTLLQ	_MVTNAIEGAQK(ac)K_	11	0.13575	64.55	2928500	2337500
>WP_013236948.1 MULTISPECIES: preprotein translocase subunit SecA	K	KGKFKAIADEIYETYKKGQPVLVGTVSIEKS	_AIADEIYETYK(ac)K_	11	0.90917	73.848	5519800	2085100
>WP_013236948.1 MULTISPECIES: preprotein translocase subunit SecA	K	LMRIFGSDRLQGIVEKLGLKEDEAIESRMVT	_LQGIVEK(ac)LGLK_	7	0.25134	67.153	8200900	0
>WP_013236948.1 MULTISPECIES: preprotein translocase subunit SecA	K	TGKGVHIVTVNDYLAKRDRDTMAPIYEALGL	_GVHIVTVNDYLAK(ac)R_	13	-0.11467	82.362	13447000	11693000
>WP_013236948.1 MULTISPECIES: preprotein translocase subunit SecA	K	LQYDDVINKQREIIYKQRSQVLEGEDLREYM	_EIIYK(ac)QR_	5	0.19515	100.82	23669000	17938000
>WP_013236948.1 MULTISPECIES: preprotein translocase subunit SecA	K	FREIYGLDVIVIPTHKPIARTDYPDVVYKSA	_EIYGLDVIVIPTHK(ac)PIAR_	14	0.04878	66.965	33246000	30609000
>WP_013236948.1 MULTISPECIES: preprotein translocase subunit SecA	K	FDIRKTLLQYDDVINKQREIIYKQRSQVLEG	_TLLQYDDVINK(ac)QR_	11	0.96056	149.26	37251000	34101000
>WP_013236948.1 MULTISPECIES: preprotein translocase subunit SecA	K	VAKNVVTNQGEETAMKKKPIKKEKTVGRNDP	_NVVTNQGEETAM(ox)K(ac)K_	13	0.18664	180.28	41200000	42372000
>WP_013236948.1 MULTISPECIES: preprotein translocase subunit SecA	K	DMLKKRGVPHQVLNAKFHEMEAEIISHAGEK	_GVPHQVLNAK(ac)FHEMEAEIISHAGEK_	10	-0.31399	87.659	100910000	74441000
>WP_013236951.1 MULTISPECIES: NADP-specific glutamate dehydrogenase	K	RRGRIKEYLKSVPTAKYVPDFREIWNVKCDI	_SVPTAK(ac)YVPDFR_	6	2.24	51.211	5727900	2263200
>WP_013236951.1 MULTISPECIES: NADP-specific glutamate dehydrogenase	K	QNSIRYSWTFEEVNNKIKNIMINIYNNSKNA	_YSWTFEEVNNK(ac)IK_	11	0.5298	42.789	19292000	25822000
>WP_013236951.1 MULTISPECIES: NADP-specific glutamate dehydrogenase	K	MDGKEYVQKVLSTVQKRNLGEEEFLDAVKEV	_VLSTVQK(ac)R_	7	0.30783	154.31	58398000	38319000
>WP_013236953.1 MULTISPECIES: RNA-binding transcriptional accessory protein	K	LGNGTASRESEELIGKLIKEVKEEKGKDLYY	_ESEELIGK(ac)LIK_	8	0.3594	47.971	5108300	5068500
>WP_013236953.1 MULTISPECIES: RNA-binding transcriptional accessory protein	K	NITDTYIEKSVEDSLKRLIYPSIEREIRSQL	_SVEDSLK(ac)R_	7	-0.11516	96.253	6869300	3249400
>WP_013236953.1 MULTISPECIES: RNA-binding transcriptional accessory protein	K	ILAGNFQDDIEKYAEKFINEEKGVESVEDAL	_YAEK(ac)FINEEK_	4	-0.26096	79.693	12216000	4287800
>WP_013236958.1 DUF2225 domain-containing protein	K	LSDYDANIIRENISPKWKSKNYPEVYDIDLA	_ENISPK(ac)WK(ac)SK_	6	-0.10775	67.993	114910000	152710000

>WP_013236960.1 leucine--tRNA ligase	K	MAVPAHDERDFAFATKYKLPIERVIEGGDSL	_DFAFATK(ac)YK_	7	0.64844	64.297	3984600	14827000
>WP_013236960.1 leucine--tRNA ligase	K	SSWYYLRYVDNKNSEKPFDKAKIDKMLPVDM	_NSEK(ac)PFDK_	4	0.50854	85.67	9201300	6189900
>WP_013236960.1 leucine--tRNA ligase	K	MYSIETDKKWQAKWEETSLYKFDDNNLD	_WQAK(ac)WEETSLYK_	4	-0.08696	83.617	13541000	34300000
>WP_013236960.1 leucine--tRNA ligase	K	SEGGAWNDDGIKSISKFVDRIERTISSVKEI	_SISK(ac)FVDR_	4	0.3877	92.439	20802000	20289000
>WP_013236962.1 MULTISPECIES: molecular chaperone DnaJ	K	CSLDPNNFKYQEALNKLRGMNNSYRQPYYDN	_YQEALNK(ac)LR_	7	1.2239	130.75	10968000	6427700
>WP_013236964.1 CYTH domain-containing protein	K	KRIFRSLGLNLLQAIKKYRESYRYKNSLIEI	_SLGLNLLQAIK(ac)K_	11	-0.24662	101.95	3031100	0
>WP_013236965.1 fructose-1,6-bisphosphate aldolase, class II	K	VILQCSAGAIKYAGPKYLKAMVDAAIEETGI	_YAGPK(ac)YLK_	5	0.68806	87.323	30707000	27961000
>WP_013236965.1 fructose-1,6-bisphosphate aldolase, class II	K	FPPNFKPKLRFDILEKVQEKLPNFPIVLHGA	_LRFDILEK(ac)VQEK_	8	0.06682	112.83	77508000	49773000
>WP_013236965.1 fructose-1,6-bisphosphate aldolase, class II	K	LRLAMTAAVRKTFGDKPEVFDPRKYLGAGRD	_TFGDK(ac)PEVFDPR_	5	0.65397	173.68	381470000	233450000
>WP_013236978.1 MULTISPECIES: lipid kinase	K	TANDFAKFIGMPQNIKKACQQIVNSVPKKLD	_FIGMPQNIK(ac)K_	9	-1.39	105.52	11831000	15888000
>WP_013236989.1 MULTISPECIES: transketolase	K	YSTLARKGYFDVDELKSLRKIGSMLQGHPNM	_KGYFDVDELK(ac)SLR_	10	0.022	142.97	226760000	232810000
>WP_013236989.1 MULTISPECIES: transketolase	K	MKKDIEELKKIAVTIRKDIIKMLT	_KDIEELK(ac)K_	7	-0.88518	137.95	123520000	96684000
>WP_013236990.1 MULTISPECIES: transketolase	K	VVVLDADLSKSTKTAKFKDAYPDRFMDMGIA	_TAK(ac)FKDAYPDR_	3	-0.28683	76.655	12902000	15232000
>WP_013236990.1 MULTISPECIES: transketolase	K	IKVSVLNIHTIKPIDKEAVINAARQTGAVIT	_VSVLNIHTIKPIDK(ac)EAVINAAR_	14	-0.18056	65.25	19370000	16599000
>WP_013236990.1 MULTISPECIES: transketolase	K	TGRAFEQVRNSICYPKLNVKICATHAGITVG	_NSICYPK(ac)LNVK_	7	0.17978	116.77	19545000	17266000
>WP_013236990.1 MULTISPECIES: transketolase	K	IKDTFGQSGKPAELLKAYKLTAEDIVKAVKK	_DTFGQSGKPAELLK(ac)AYK_	14	0.00226	117.37	49688000	33768000
>WP_013236990.1 MULTISPECIES: transketolase	K	AELLKAYKLTAEDIVKAVKKCMALK	_LTAEDIVK(ac)AVK_	8	0.5007	88.441	149770000	133500000
>WP_013236990.1 MULTISPECIES: transketolase	K	TFGQSGKPAELLKAYKLTAEDIVKAVKKCMA	_AYK(ac)LTAEDIVK(ac)AVK_	3	0.29844	112.83	472680000	309140000
>WP_013236991.1 MULTISPECIES: cell division ATP-binding protein FtsE	K	VFLVGPSGAGKTTFVKALLKEVQPTSGDIIV	_TTFVK(ac)ALLK_	5	0.90653	68.016	7452600	5421100
>WP_013236991.1 MULTISPECIES: cell division ATP-binding protein FtsE	K	QPTSGDIIVNNLNITKLKRSKIPLYRRKLGV	_EVQPTSGDIIVNNLNITK(ac)LK_	18	0.68443	44.587	7585500	4610400
>WP_013236995.1 MULTISPECIES: excinuclease ABC subunit UvrB	K	QMKTAAKELRFEEAAKLRDAILKLKKQKNK	_FEEAAK(ac)LR_	6	0.36505	60.436	0	3724300
>WP_013236995.1 MULTISPECIES: excinuclease ABC subunit UvrB	K	KPIKGQIDDLYNSIQKTISRGYRILVTTLTK	_GQIDDLYNSIQK(ac)TISR_	12	-0.18171	84.874	6550600	0
>WP_013236995.1 MULTISPECIES: excinuclease ABC subunit UvrB	K	MGVFKIHSKFKPTGDQPKAIDSIA	_IHSK(ac)FKPTGDQPK_	4	0.08126	136.3	44565000	45836000
>WP_013236996.1 excinuclease ABC subunit UvrA	K	KGKKGEHIKVLESIVKNGFVRARIDGEIVDL	_VLESIVK(ac)NGFVR_	7	-2.3622	57.019	2102500	4189800
>WP_013237002.1 MULTISPECIES: UDP-N-acetylenolpyruvoylglucosamine reductase	K	LVKDGGIRGIMIKLIKLNEVKVEDNKIITES	_LIK(ac)LNEVK_	3	0.06902	62.582	0	5776000
>WP_013237002.1 MULTISPECIES: UDP-N-acetylenolpyruvoylglucosamine reductase	K	ATAKDILDLIAIVQHKVKEKFNVDLYTEVRV	_DILDLIAIVQHK(ac)VK_	12	-0.48036	123.75	0	343160000
>WP_013237007.1 MULTISPECIES: 6-phosphofructokinase	K	KGYDEQELCRSILEAKMKGKMHNLILLAEGI	_SILEAK(ac)MK_	6	-0.13022	77.058	28626000	22803000
>WP_013237007.1 MULTISPECIES: 6-phosphofructokinase	K	VVRTALDKNLNVMGIKRGYSGLMNGEIVPMQ	_NLNVMGIK(ac)R_	8	0.21603	143.01	36051000	30314000
>WP_013237007.1 MULTISPECIES: 6-phosphofructokinase	K	EFKTEEGRERAVNILKTFRIDGLVVIGGDGS	_AVNILK(ac)TFR_	6	0.43032	101.46	37955000	4025700
>WP_013237007.1 MULTISPECIES: 6-phosphofructokinase	K	AEKVENITGIETRATKLGHIQRGGSPTCSDR	_ATK(ac)LGHIQR_	3	-0.15311	155.07	235210000	172840000

>WP_013237007.1 MULTISPECIES: 6-phosphofructokinase	K	QRGGTILKTARSKEFKTEEGRERAVNILKTF	_SK(ac)EFK(ac)TEEGR_	5	-1.4658	136.96	552520000	508630000
>WP_013237007.1 MULTISPECIES: 6-phosphofructokinase	K	RSSVADIIQRGGTILKTARSKEFKTEEGRER	_GGTILK(ac)TAR_	6	0.04609	141.02	31542000	25970000
>WP_013237008.1 MULTISPECIES: pyruvate kinase	K	IMVARGDMGVEIPIEKVPMIQKFIIEKCNKA	_GDMGVEIPIEK(ac)VPMIQK_	11	-0.06092	57.499	30120000	43681000
>WP_013237008.1 MULTISPECIES: pyruvate kinase	K	AKRAEEQINYDSLLEKKREAHIQNVPNAISL	_RAEEQINYDSLLEK(ac)K_	14	0.68184	132.05	46625000	38765000
>WP_013237008.1 MULTISPECIES: pyruvate kinase	K	EVVEKDDILVVKDLDKGYINILDRVAGIISE	_DLDK(ac)GYINILDR_	4	0.26299	131.06	50981000	48682000
>WP_013237008.1 MULTISPECIES: pyruvate kinase	K	IMLDTKGPEIRTGNFKEDKAELKEGQQFTVY	_TGNFK(ac)EDK_	5	-0.12327	98.033	81778000	75194000
>WP_013237008.1 MULTISPECIES: pyruvate kinase	K	VSRHNFSHGDHEEHEKRINMVKKLREKYNKP	_HNFSHGDHEEHEK(ac)R_	13	0.30416	215.87	123360000	377380000
>WP_013237009.1 23S rRNA (uracil(1939)-C(5))-methyltransferase RlmD	K	DFKKKRVKDCLERIGKFEVEEQDRIFKDNVL	_IGK(ac)FEVEEQDR_	3	-0.34455	76.655	0	4508200
>WP_013237039.1 hypothetical protein	K	DLISEKDGNRYAISVKTRNFKKGSNESRMYS	_YAISVK(ac)TR_	6	0.18766	126.52	3444300	3092200
>WP_013237050.1 hypothetical protein	K	YVVYSGNIKNPANTLKIGINEYKVNSISMAD	_NPANTLK(ac)IGINEYK_	7	-0.23785	32.275	3170400	57539000
>WP_013237050.1 hypothetical protein	K	KGKDEGKKEKTIEIVKRAIKKGMDNETIKEL	_TIEIVK(ac)R_	6	-0.2586	119.89	29240000	20664000
>WP_013237101.1 MULTISPECIES: chemotaxis protein	K	STSLTAIREKRVLSQKVPRSAYGMRLSITSI	_VLSQK(ac)VPR_	5	-0.01127	81.191	3580700	3636500
>WP_013237107.1 phosphoglycolate phosphatase	K	GTRCTKGEVIKHVLEKYRINSDEAVMIGDRK	_HVLEK(ac)YR_	5	0.1392	108.24	97237000	78460000
>WP_013237118.1 MULTISPECIES: transcriptional regulator	K	IPENNRRIVKLSISEKFKKSNDIVNIRQVFI	_LSISEK(ac)FK_	6	0.02662	69.954	5663700	4829700
>WP_013237122.1 carbamoyl-phosphate synthase small subunit	K	VSEVSTKGKYAIGEGKRNVAIMDFGIKKTMV	_YAIGEGK(ac)R_	7	-0.6781	87.181	22806000	14048000
>WP_013237123.1 carbamoyl-phosphate synthase large subunit	K	SLDYVVVKIPKWPFDKFHNADRELGTKMMAT	_WPFDK(ac)FHNADR_	5	-0.42799	46.592	0	3469200
>WP_013237124.1 MULTISPECIES: amidohydrolase	K	VGNPEKGIIYPQHHPKYDIDERAMAYGVEVM	_GIIYPQHHPK(ac)YDIDER_	10	0.0694	89.541	10625000	9326600
>WP_013237124.1 MULTISPECIES: amidohydrolase	K	MSDIKELTEKYYPEIVELRRYFHKY	_ELTEK(ac)YYPEIVELR_	5	-0.1285	55.841	21808000	12435000
>WP_013237131.1 MULTISPECIES: glutamate-1-semialdehyde-2,1-aminomutase	K	PVRAFKDVPLNPPVIKRGKGAYIYDEDGNKY	_DVPLNPPVIK(ac)R_	10	0.38757	92.538	27245000	34832000
>WP_013237131.1 MULTISPECIES: glutamate-1-semialdehyde-2,1-aminomutase	K	LKKLHDNPDYYEHLEKLGAKLEQGIKDIAKK	_LHDNPDYYEHLEK(ac)LGAK_	13	0.64301	140.81	161140000	198720000
>WP_013237142.1 MULTISPECIES: methionine ABC transporter ATP-binding protein	K	EGEIKTSGNVEEIFLKPGKNLKKLLGNDSLL	_TSGNVEEIFLK(ac)PGK_	11	-0.69625	89.507	27805000	20214000
>WP_013237144.1 DUF2088 domain-containing protein	K	VKIGTLPSGGELIVNKLAVKADLLIAEGFIE	_IGTLPSGGELIVNK(ac)LAVK_	14	0.2457	66.335	19450000	9728000
>WP_013237147.1 MULTISPECIES: pyridoxal phosphate-dependent aminotransferase	K	MISNKMKEHVSKSSIIRAMFEEGKRLA	_EHVSK(ac)SSIIR_	5	0.76546	51.092	1316300	2819400
>WP_013237147.1 MULTISPECIES: pyridoxal phosphate-dependent aminotransferase	K	SGYEDVRAKIAENINKKYNLKLTENNLVMTC	_IAENINK(ac)K_	7	0.77167	77.923	15175000	12074000
>WP_013237166.1 cell wall-binding repeat-containing protein	K	SKSVSFDYDGNDLTVKFKYIGTNVDGSTYTD	_SVSFDYDGNDLTVK(ac)FK_	14	0.01587	44.294	6841800	9900200
>WP_013237166.1 cell wall-binding repeat-containing protein	K	FDTNLAVLKAFSSDLKADKLYIANASAADPD	_AFSSDLK(ac)ADK_	7	-0.16145	69.979	20819000	9581400
>WP_013237166.1 cell wall-binding repeat-containing protein	K	ATLQDDNKTVLITLAKSKKQGDNLDLTVKKG	_TVLITLAK(ac)SK_	8	-0.3298	91.549	41672000	17872000
>WP_013237166.1 cell wall-binding repeat-containing protein	K	HMANKLSDSKYSITIKNIRDIASTPNTMPDY	_YSITIK(ac)NIR_	6	0.34774	154.49	62772000	44678000
>WP_013237166.1 cell wall-binding repeat-containing protein	K	LAKSKKQGDNLDLTVKKGILSSDKSTTIPEC	_QGDNLDLTVK(ac)K_	10	-0.24623	171.29	160980000	125680000

>WP_013237166.1 cell wall-binding repeat-containing protein	K	ILSSDKSTTIPECTQKVSFNDTTAPTISSVQ	_STTIPECTQK(ac)VSFNDTTAPTISSVQAR_	10	-0.32104	75.896	179410000	179170000
>WP_013237169.1 metallophosphoesterase	K	HGNLEALKAALCDIEKKSIDTIICLGDLVGY	_AALCDIEK(ac)K_	8	-1.169	59.931	7209700	388860000
>WP_013237181.1 MULTISPECIES: cell wall-binding repeat-containing protein	K	TLDSITAIGGNKLRVKFSEPIRMDSSNLSSF	_VK(ac)FSEPIR_	2	-0.00615	103.56	9978200	14569000
>WP_013237192.1 MULTISPECIES: SLAP domain-containing protein	K	QPITINKMPMTVLDAKKRAVKSESFELKDFT	_MPMTVLDAK(ac)K_	9	-1.5734	107.24	34948000	26775000
>WP_013237193.1 MULTISPECIES: cell wall-binding repeat-containing protein	K	TDLPKYNSASEYEAFKDDLTTKISNYFKNDS	_YNSASEYEAFK(ac)DDLTTK_	11	-0.47255	100.35	16446000	11327000
>WP_013237198.1 tyrosine protein kinase	K	TTNEVGLSDVLVGKVKFEEAVKETGIENLNL	_VK(ac)FEEAVK_	2	-0.31909	82.426	54017000	49506000
>WP_013237250.1 hypothetical protein	K	ATMNRYRNDFYNPIFKRNEIIGVTHSAKTDA	_YRNDFYNPIFK(ac)R_	11	-0.10698	109.29	3701000	0
>WP_013237253.1 hypothetical protein	K	MVSVPRNAKKILEGIKEYKKNSNKGNAINIK	_ILEGIK(ac)EYK_	6	0.39085	65.347	2424300	9325200
>WP_013237253.1 hypothetical protein	K	KKNDYIEAKSEEYLLKSEEQRIMRMADFYSS	_SEEYLLK(ac)SEEQR_	7	2.2494	63.727	3704000	0
>WP_013237253.1 hypothetical protein	K	HLQNRKPLTGYKNREKFNYDNVIKKQSVNID	_EK(ac)FNYDNVIK_	2	0.49721	47.559	7046900	7944800
>WP_013237253.1 hypothetical protein	K	NVDSFLRALYTETIIKKGLLYVYNGSEDSKV	_ALYTETIIK(ac)K_	9	-0.96725	79.974	12153000	8284200
>WP_013237253.1 hypothetical protein	K	GEILKLVKDMVNQIFKDPKKGPFYASRVLFS	_DMVNQIFK(ac)DPK_	8	1.2797	56.225	12904000	7508300
>WP_013237253.1 hypothetical protein	K	AKIDLIRTPELTELVKKEVEKYEKIIDKKKE	_TPELTELVK(ac)K_	9	-0.27241	164.97	19412000	21424000
>WP_013237253.1 hypothetical protein	K	TGYKNREKFNYDNVIKKQSVNIDEEMKEYLR	_FNYDNVIK(ac)K_	8	-0.20594	91.937	26386000	30465000
>WP_013237253.1 hypothetical protein	K	VEILKLKVSSIIEEVKSSVGELDTRKYQMVE	_VSSIIEEVK(ac)SSVGELDTR_	9	0.39421	148.56	29653000	19478000
>WP_013237253.1 hypothetical protein	K	YNMDMKGDKINIGEVKRAIGDMNKLIQDKLT	_INIGEVK(ac)R_	7	-0.4628	131.06	35566000	22647000
>WP_013237256.1 hypothetical protein	K	KTDNLDDLSLNDAELKSAKLYTMEEYKEFNG	_TDNLDDLSLNDAELK(ac)SAK_	15	-0.76854	71.742	4305100	10174000
>WP_013237260.1 hypothetical protein	K	DPTPKQHVEPLIENLKFKEDKIPPLTLIFYD	_QHVEPLIENLK(ac)FK_	11	-0.22636	127.56	30273000	29813000
>WP_013237264.1 ABC transporter substrate-binding protein	K	EKLVEKNPDILICSNKFNSKNGIKSTKGYKD	_NPDILICSNK(ac)FNSK_	10	-0.00157	31.389	3289800	0
>WP_013237274.1 malate dehydrogenase	K	KYYSEIKSGLININSKIKIVKETPVSATIDG	_SGLININSK(ac)IK_	9	-0.56128	68.44	14247000	13492000
>WP_013237282.1 glutamate-5-semialdehyde dehydrogenase	K	KNAAENFLPHLGETLKSLGVEIRGCSETKKL	_NAAENFLPHLGETLK(ac)SLGVEIR_	15	0.26693	59.814	0	24278000
>WP_013237282.1 glutamate-5-semialdehyde dehydrogenase	K	LIKVASLPDPIGEVSKMWKRPNGLRIGKVRV	_VASLPDPIGEVSK(ac)MWK_	13	0.82256	128.9	40791000	28719000
>WP_013237282.1 glutamate-5-semialdehyde dehydrogenase	K	SGKGKAFIDRLELNQKRIDSMAGGLIKVASL	_LELNQK(ac)R_	6	0.02124	164.63	109730000	97268000
>WP_013237299.1 MULTISPECIES: ATP-dependent chaperone ClpB	K	IPVSKLVEGERQKLVKLEDELAKRVIGQKEA	_LVK(ac)LEDELAK_	3	0.42711	55.314	3961500	6596400
>WP_013237299.1 MULTISPECIES: ATP-dependent chaperone ClpB	K	LKYGVIPKLESTIDEKEQSIKENNEAAMLKE	_LESTIDEK(ac)EQSIK_	8	1.0011	47.189	5213700	2723500
>WP_013237299.1 MULTISPECIES: ATP-dependent chaperone ClpB	K	YEALAKYGRNLVEEAKKHKLDPVIGRDEEIR	_NLVEEAK(ac)K_	7	-0.00321	84.476	6979700	6102300
>WP_013237299.1 MULTISPECIES: ATP-dependent chaperone ClpB	K	FIFLGPTGVGKTELAKTLARTLFDSEENIIR	_TELAK(ac)TLAR_	5	-0.21446	64.297	7174100	7687700
>WP_013237299.1 MULTISPECIES: ATP-dependent chaperone ClpB	K	EKEQSIKENNEAAMLKEEVTEQEISQIVSKW	_ENNEAAMLK(ac)EEVTEQEISQIVSK_	9	0.09287	38.237	9343600	5834500
>WP_013237299.1 MULTISPECIES: ATP-dependent chaperone ClpB	K	PLKRYIENILETSIAKKIINGDIYTGCKVRV	_YIENILETSIAK(ac)K_	12	1.294	70.412	15680000	16881000
>WP_013237299.1 MULTISPECIES: ATP-dependent chaperone ClpB	K	VYATRRFEDVFLQAEKIAQKFKDSYISVEHV	_RFEDVFLQAEK(ac)IAQK_	11	0.61748	43.791	16973000	11431000

>WP_013237299.1 MULTISPECIES: ATP-dependent chaperone ClpB	K	EALAKYGRNLVEEAKKHKLDPVIGRDEEIRR	_K(ac)HKLDPVIGR_	1	0.99997	70.056	17035000	16844000
>WP_013237299.1 MULTISPECIES: ATP-dependent chaperone ClpB	K	KEKDTASMERLKAVEKELSNLKDRDNEMTAK	_AVEK(ac)ELSNLK_	4	0.32897	114.97	19554000	17488000
>WP_013237299.1 MULTISPECIES: ATP-dependent chaperone ClpB	K	IDKDVRDKVMSDMKFKFKPEFLNRLDDIIMF	_FK(ac)FKPEFLNR_	2	-0.2379	143.89	27526000	16421000
>WP_013237299.1 MULTISPECIES: ATP-dependent chaperone ClpB	K	EGERQKLVKLEDELAKRVIGQKEAVTAVSNA	_LEDELAK(ac)R_	7	0.21089	144.38	43402000	36187000
>WP_013237299.1 MULTISPECIES: ATP-dependent chaperone ClpB	K	DGLIPNIFGKMSVNLKSLVKETKDVLDKMPK	_MSVNLK(ac)SLVK_	6	-0.85612	99.283	69104000	80071000
>WP_013237299.1 MULTISPECIES: ATP-dependent chaperone ClpB	K	YDLNKIAELKYGVIPKLESTIDEKEQSIKEN	_YGVIPK(ac)LESTIDEK_	6	-0.05241	94.023	95016000	63955000
>WP_013237299.1 MULTISPECIES: ATP-dependent chaperone ClpB	K	DIYTGCKVRVDYENDKFKIEKL	_VRVDYENDK(ac)FK_	9	-1.1069	132.79	95976000	86969000
>WP_013237299.1 MULTISPECIES: ATP-dependent chaperone ClpB	K	LAKYGRNLVEEAKKHKLDPVIGRDEEIRRVV	_HK(ac)LDPVIGR_	2	-0.12502	119.75	216450000	221720000
>WP_013237303.1 phosphoenolpyruvate carboxykinase (ATP)	K	GVPDEILNPRNTWEDKEAYDETARKLALKFS	_NTWEDK(ac)EAYDETAR_	6	0.12706	124.45	21341000	17527000
>WP_013237303.1 phosphoenolpyruvate carboxykinase (ATP)	K	EDKEAYDETARKLALKFSKNFEKFKDVSEDI	_LALK(ac)FSK_	4	0.1521	94.662	44501000	25524000
>WP_013237310.1 MULTISPECIES: dihydropteroate synthase	K	SFSDGGKFNNIELAIKHAKQMEEEGADIIDV	_FNNIELAIK(ac)HAK_	9	0.07756	84.289	20469000	16581000
>WP_013237310.1 MULTISPECIES: dihydropteroate synthase	K	VSEQEELERVIPIIGKLSRCVDIPISIDTYK	_VIPIIGK(ac)LSR_	7	1.6382	83.862	38236000	48639000
>WP_013237310.1 MULTISPECIES: dihydropteroate synthase	K	MDKVITIGKKDFQIGKRTYIMGILNVTPDSF	_KDFQIGK(ac)R_	7	0.9691	112.75	281800000	190300000
>WP_013237313.1 2-amino-4-hydroxy-6-hydroxymethyldihydropteridine diphosphokinase	K	FLLEVEKKLKRERIIKWGPRTIDLDVILYDN	_IIK(ac)WGPR_	3	-0.23908	129.96	8771800	8482600
>WP_013237313.1 2-amino-4-hydroxy-6-hydroxymethyldihydropteridine diphosphokinase	K	PVLNDRIINLTHELEKTQEL	_IINLTHELEK(ac)TQEL_	10	-0.15492	32.734	9325200	7649200
>WP_013237330.1 MULTISPECIES: 1-acyl-sn-glycerol-3-phosphate acyltransferase	K	RSKGPRIGEFKKGSLKLALKSKVPIVPIAID	_KGSLK(ac)LALK_	5	-0.25605	112.71	6547400	33385000
>WP_013237332.1 aconitate hydratase	K	IEDTLKQIKNDVVTVKNKNRNEEYKMVFNVT	_NDVVTVK(ac)NK_	7	0.87387	75.294	3177900	2574400
>WP_013237332.1 aconitate hydratase	K	TNSSFVDMMKVAHILKGKTVNENVSLTISPG	_VAHILK(ac)GK_	6	0.1117	63.21	8980600	6385600
>WP_013237332.1 aconitate hydratase	K	VGDNITTDHIMPSNAKLLPYRSNIPHLAEYC	_VGDNITTDHIM(ox)PSNAK(ac)LLPYR_	16	0.1362	168.62	113320000	119000000
>WP_013237333.1 isocitrate/isopropylmalate dehydrogenase family protein	K	PDNIIESIKKNKVALKAPVTTPVGKGFKSVN	_VALK(ac)APVTTPVGK_	4	0.31207	80.522	8092600	6241200
>WP_013237333.1 isocitrate/isopropylmalate dehydrogenase family protein	K	DKYGTPIPDNIIESIKKNKVALKAPVTTPVG	_YGTPIPDNIIESIK(ac)K_	14	-1.0334	30.543	19527000	16266000
>WP_013237333.1 isocitrate/isopropylmalate dehydrogenase family protein	K	NGRKKVTAVHKANIMKLSDGLFLKTAEKVAE	_ANIMK(ac)LSDGLFLK_	5	-0.22411	80.522	21451000	12953000
>WP_013237334.1 MULTISPECIES: O-acetylhomoserine aminocarboxypropyltransferase/cysteine synthase	K	HPASTTHAELNEEQQKAAGVTPDLIRLSIGV	_SLVIHPASTTHAELNEEQQK(ac)AAGVTPDLIR_	20	-0.08036	88.001	23743000	22194000
>WP_013237334.1 MULTISPECIES: O-acetylhomoserine aminocarboxypropyltransferase/cysteine synthase	K	KVAWVNYPELDGSPYKELAKKYLPKGAGSIF	_VAWVNYPELDGSPYK(ac)ELAK_	15	0.35491	38.017	35091000	26306000
>WP_013237334.1 MULTISPECIES: O-acetylhomoserine aminocarboxypropyltransferase/cysteine synthase	K	GGHGTTLGGIIVDGGKFDWAASGKFPDFTTP	_FIGGHGTTLGGIIVDGGK(ac)FDWAASGK_	18	0.79803	63.122	36108000	45634000

>WP_013237334.1 MULTISPECIES: O-acetylhomoserine aminocarboxypropyltransferase/cysteine synthase	K	ERHVENTRKVVEFLSKHPKVAWVNYPELDGS	_VVEFLSK(ac)HPK_	7	-0.35941	119.16	506240000	500880000
>WP_013237337.1 MULTISPECIES: amino acid ABC transporter substrate-binding protein	K	LDELKKDGTMSKISTKWFGEDIYK	_ISTK(ac)WFGEDIYK_	4	0.35376	42.789	6436200	5015800
>WP_013237340.1 MULTISPECIES: argininosuccinate synthase	K	KAIKTGASKVYIEDLKEEFVKDYLFKAVKAN	_VYIEDLK(ac)EEFVK_	7	0.31682	62.14	4443800	3355000
>WP_013237340.1 MULTISPECIES: argininosuccinate synthase	K	VGQDDDMEEVEKKAIKTGASKVYIEDLKEEF	_AIK(ac)TGASK_	3	-0.50079	94.363	33385000	5749300
>WP_013237340.1 MULTISPECIES: argininosuccinate synthase	K	DPTIKIIAPWRIWDIKSREDAIDYANSKGVD	_IWDIK(ac)SR_	5	0.27074	102.71	49206000	49783000
>WP_013237341.1 MULTISPECIES: argininosuccinate lyase	K	EERDKAIEDVSVDELKKFSSLFSDDVYEFIN	_AIEDVSVDELK(ac)K_	11	0.39117	54.7	10423000	2526300
>WP_013237341.1 MULTISPECIES: argininosuccinate lyase	K	LYCKKSALEVIKNIEKLQDVIKNLGENNNIM	_NIEK(ac)LQDVIK_	4	0.40278	123.79	194550000	151960000
>WP_013237344.1 hypothetical protein	K	LNITGPFERHTTQELKAKYGDDFEAIASQKA	_HTTQELK(ac)AK_	7	-0.0956	67.113	11712000	11919000
>WP_013237347.1 xanthine dehydrogenase	K	TIGGGRLEADAIGLAKKSLSLKQTVMQSFDL	_LEADAIGLAK(ac)K_	10	0.15619	69.979	7295000	2501700
>WP_013237347.1 xanthine dehydrogenase	K	MEKIYKEVVKLLQKEESFVIATIFD	_EVVK(ac)LLQK_	4	0.47634	65.252	10735000	9247100
>WP_013237354.1 MULTISPECIES: ribonucleoside-diphosphate reductase, adenosylcobalamin-dependent	K	SYIGKTLKDCWINALKSGIKFGYRNAQVSVI	_DCWINALK(ac)SGIK_	8	-0.00846	71.241	3789600	0
>WP_013237362.1 NAD(+) synthase	K	IQTSALAKRVSHTNLKRAVIGISGGLDSTLA	_VSHTNLK(ac)R_	7	-0.07201	96.143	21349000	45489000
>WP_013237364.1 MULTISPECIES: SPFH/Band 7/PHB domain protein	K	GTDEKVIALKQVEALKEMAKNPANKLILPNE	_QVEALK(ac)EMAK_	6	-0.96583	64.73	0	7000300
>WP_013237364.1 MULTISPECIES: SPFH/Band 7/PHB domain protein	K	RSIIDSGTDEKVIALKQVEALKEMAKNPANK	_VIALK(ac)QVEALK_	5	0.04239	90.685	11306000	9258300
>WP_013237369.1 formate dehydrogenase H subunit alpha, selenocysteine-containing	K	LADEPGYVQVNPMDAKAKGIIDGELMRISSR	_ALQQLADEPGYVQVNPMDAK(ac)AK_	20	-1.1933	75.358	12880000	5432600
>WP_013237369.1 formate dehydrogenase H subunit alpha, selenocysteine-containing	K	AGCNLYLHVKNGKIIKAEPANGRTNEGSLCL	_LHVKNGKIIK(ac)AEPANGR_	10	-1.3385	37.283	19536000	0
>WP_013237369.1 formate dehydrogenase H subunit alpha, selenocysteine-containing	K	KTPELKYCAVKIEAIKDQKEAEKFIKDQYDL	_IEAIK(ac)DQK_	5	-0.12264	99.139	26812000	18397000
>WP_013237369.1 formate dehydrogenase H subunit alpha, selenocysteine-containing	K	CAVKIEAIKDQKEAEKFIKDQYDLLKKKMNV	_EAEK(ac)FIK_	4	-0.94146	79.597	27718000	17150000
>WP_013237369.1 formate dehydrogenase H subunit alpha, selenocysteine-containing	K	PSENHPGTSYLYKGNKFNTPTGKANLFAAEW	_GNK(ac)FNTPTGK_	3	-0.22101	134.68	688630000	517120000
>WP_013237373.1 MULTISPECIES: NADH-quinone oxidoreductase subunit NuoE	K	RKYKYIPREALENLAKYLDTPVSRLYGMATF	_EALENLAK(ac)YLDTPVSR_	8	-1.1345	58.671	6907100	2702500
>WP_013237373.1 MULTISPECIES: NADH-quinone oxidoreductase subunit NuoE	K	TKQQNEDLSGQDVIEKYPKEQRFTLAILQDI	_QQNEDLSGQDVIEK(ac)YPK_	14	0.33931	74.786	18286000	16938000
>WP_013237374.1 MULTISPECIES: NADH dehydrogenase	K	KGSMEVYEEMKAQIAKLGANAEVNVKLKATG	_AQIAK(ac)LGANAEVNVK_	5	-1.524	55.213	1731600	0
>WP_013237374.1 MULTISPECIES: NADH dehydrogenase	K	ATKQRLRNHKESEFCKRQYKIALRNVGEIDP	_ESEFCK(ac)R_	6	0.1369	98.133	4560700	0
>WP_013237374.1 MULTISPECIES: NADH dehydrogenase	K	IASIRYFRDEFEEHIKNKRCRAGVCKKLTTF	_DEFEEHIK(ac)NKR_	8	0.36209	104.79	9446200	9683700
>WP_013237374.1 MULTISPECIES: NADH dehydrogenase	K	TGCLAKGSMEVYEEMKAQIAKLGANAEVNVK	_GSMEVYEEMK(ac)AQIAK_	10	-1.9088	83.729	22698000	11249000
>WP_013237374.1 MULTISPECIES: NADH dehydrogenase	K	ERGGYKALKKAISSMKPEDVLEEITKSGLRG	_AISSMK(ac)PEDVLEEITK_	6	0.32237	78.285	32915000	24354000

>WP_013237374.1 MULTISPECIES: NADH dehydrogenase	K	KAQIAKLGANAEVNVKLKATGCDGLCEKGPV	_LGANAEVNVK(ac)LK_	10	-0.89276	88.187	83166000	91198000
>WP_013237374.1 MULTISPECIES: NADH dehydrogenase	K	IEKGGDWYHAIGTMEKSKGTKVFSLVGKVKN	_GGDWYHAIGTMEK(ac)SK_	13	-0.18541	169.37	85298000	46367000
>WP_013237374.1 MULTISPECIES: NADH dehydrogenase	K	NGEIIEKLLYFETATKQRLRNHKESEFCKRQ	_LLYFETATK(ac)QR_	9	3.0566	192.1	100370000	94073000
>WP_013237374.1 MULTISPECIES: NADH dehydrogenase	K	IAYFKVKVEDVEDVVKKTLMNGEIIEKLLYF	_VKVEDVEDVVK(ac)K_	11	0.12275	143.86	164040000	152090000
>WP_013237375.1 NADH dehydrogenase	K	PVSKEVEVITNDDEIKRMRKNIVMLLKVRCP	_EVEVITNDDEIK(ac)R_	12	-5.9867	206.69	101510000	105510000
>WP_013237375.1 NADH dehydrogenase	K	NKEVNELAKAFGVEEKRVKRFKLDPEQNCVL	_AFGVEEK(ac)R_	7	0.15368	143.12	158700000	120420000
>WP_013237377.1 MULTISPECIES: 4Fe-4S dicluster domain-containing protein	K	DANLTYRKSHENPEIKKIYDEFLDKPLGAKS	_KSHENPEIK(ac)K_	9	0.3583	61.48	2810000	5691400
>WP_013237377.1 MULTISPECIES: 4Fe-4S dicluster domain-containing protein	K	QMAGAIFKTYGAEINKVNPAKIYNVSVMPCT	_TYGAEINK(ac)VNPAK_	8	-0.02304	51.875	4487100	8485200
>WP_013237377.1 MULTISPECIES: 4Fe-4S dicluster domain-containing protein	K	VVSGLKNVKDVMESVKAGKCDLHFIEVMTCP	_DVMESVK(ac)AGK_	7	-0.3327	43.168	5234700	5304700
>WP_013237377.1 MULTISPECIES: 4Fe-4S dicluster domain-containing protein	K	KGYQSVYDDVPTPVSKRLFDRGLLKEVDEPL	_GYQSVYDDVPTPVSK(ac)R_	15	0.40604	96.414	12040000	14213000
>WP_013237377.1 MULTISPECIES: 4Fe-4S dicluster domain-containing protein	K	HENPEIKKIYDEFLDKPLGAKSHELLHTKYI	_IYDEFLDK(ac)PLGAK_	8	0.11214	78.763	88455000	33855000
>WP_013237378.1 MULTISPECIES: 4Fe-4S dicluster domain-containing protein	K	ASQYRDGEVVDQKGLKMSEEGNPTVNGKGRV	_GLK(ac)MSEEGNPTVNGK_	3	-0.39489	50.155	4850800	0
>WP_013237378.1 MULTISPECIES: 4Fe-4S dicluster domain-containing protein	K	SLKLVTYDDNNNIVEKKDDDEREVS	_LVTYDDNNNIVEK(ac)K_	13	0.36618	104.17	28831000	16502000
>WP_013237381.1 chemotaxis protein	K	ALEIGVDPNEYIEALKKVKVVEKDRVEAIAK	_ALEIGVDPNEYIEALK(ac)K_	16	-0.35867	50.608	0	6246400
>WP_013237382.1 MULTISPECIES: APC family permease	K	LSIDEKPKEINFAEQKRYVIVPIDTLNKSFL	_EINFAEQK(ac)R_	8	0.07793	91.087	4719200	2654000
>WP_013237384.1 MULTISPECIES: hypothetical protein	K	GITSHKVTIGLILAHKTGSLSNILDKIAQRR	_VTIGLILAHK(ac)TGSLSNILDK_	10	0.76952	49.34	8258800	1889100
>WP_013237385.1 MULTISPECIES: homoserine dehydrogenase	K	IVENKREKTITFSSEKASINKSWNLKSKWLL	_TITFSSEK(ac)ASINK_	8	-0.02811	98.531	0	9888400
>WP_013237385.1 MULTISPECIES: homoserine dehydrogenase	K	KDLMAERGYELLQLAKKNGVTIHFEASVGGG	_GYELLQLAK(ac)K_	9	0.39294	99.653	24090000	25488000
>WP_013237386.1 homoserine kinase	K	GEDIKHSKVNVPDKLKFVAMIPSFKVNTALS	_LK(ac)FVAMIPSFK_	2	0.21783	36.417	0	9041900
>WP_013237386.1 homoserine kinase	K	NEQGYSYNGFKINVLKCDIPMSRGLGSSSAC	_INVLK(ac)CDIPMSR_	5	-1.9208	43.991	1816600	11705000
>WP_013237386.1 homoserine kinase	K	KVGEDIKHSKVNVPDKLKFVAMIPSFKVNTA	_VNVPDK(ac)LK_	6	0.05261	79.906	8390000	7156500
>WP_013237386.1 homoserine kinase	K	NVPDKLKFVAMIPSFKVNTALSREVLPKSYL	_FVAMIPSFK(ac)VNTALSR_	9	-0.05045	117.37	86911000	86895000
>WP_013237386.1 homoserine kinase	K	QPYRKSLIRNFDDVFKKSKDLGSIGEFISGS	_NFDDVFK(ac)K_	7	-0.2158	73.665	195300000	211680000
>WP_013237386.1 homoserine kinase	K	GSGSTLMAVVDKNAEKFVSSMKNFLSELEDT	_NAEK(ac)FVSSMK_	4	-0.29481	113.63	383140000	340410000
>WP_013237392.1 MULTISPECIES: rod shape-determining protein	K	IVLREPSVVAINNDVKKVMAIGNEAKDMIGR	_EPSVVAINNDVK(ac)K_	12	-0.14282	32.624	6605900	15623000
>WP_013237392.1 MULTISPECIES: rod shape-determining protein	K	DCVALGAGKALDTIDKIVASRK	_ALDTIDK(ac)IVASR_	7	-0.27818	90.926	9888600	8241100
>WP_013237392.1 MULTISPECIES: rod shape-determining protein	K	SGVTEVEKRAIEEATKHAGARDVLLMEEPMA	_AIEEATK(ac)HAGAR_	7	-0.11236	63.727	10524000	14345000
>WP_013237392.1 MULTISPECIES: rod shape-determining protein	K	EPVAAIIDAIKTTLEKTPPELASDIMDKGIM	_TTLEK(ac)TPPELASDIMDK_	5	-0.15298	97.502	45894000	40023000

>WP_013237396.1 MULTISPECIES: septum site-determining protein MinC	K	GCTLKITTELKSLNEKQFNKLKNILFEKFLI	_SLNEK(ac)QFNK_	5	0.44155	93.551	7424500	5115500
>WP_013237397.1 MULTISPECIES: septum site-determining protein MinD	K	NPEVTSVRDSDRVIGKLDAKGIEKHQLIVNR	_VIGK(ac)LDAK_	4	0.30949	80.438	112800000	101780000
>WP_013237400.1 MULTISPECIES: methylglyoxal synthase	K	MRIAFIAHDKKKDDIVDFVKRYKDV	_IAFIAHDK(ac)K_	8	-0.65185	65.347	7811100	5633400
>WP_013237400.1 MULTISPECIES: methylglyoxal synthase	K	FIAHDKKKDDIVDFVKRYKDVFEGHEIFATG	_KDDIVDFVK(ac)R_	9	0.26823	157.38	23607000	17096000
>WP_013237412.1 RidA family protein	K	KYFKKDMPARSCVEVKLPKDALVEVEVIALV	_SCVEVK(ac)LPK_	6	-0.26591	64.711	5623200	3763900
>WP_013237417.1 L-seryl-tRNA(Sec) selenium transferase ;>WP_013239415.1 L-seryl-tRNA(Sec) selenium transferase	K	KGQRGSRYSHVEELIKRVTGAEAAMVVNNNA;KGTRGSRYSHVEELIKRITGAEASLVVNNNA	_YSHVEELIK(ac)R_	9	-0.41204	100.48	5399400	5898100
>WP_013237428.1 AcrB/AcrD/AcrF family protein	K	ANEKFDDIKSTSKLVKNIEGVLKQQKEVTAY	_LVK(ac)NIEGVLK_	3	-0.10777	42.718	0	77233000
>WP_013237439.1 MULTISPECIES: hydroxylamine reductase	K	GKQPDVANKQDKLTCKLIELAYASEGKNRDK	_LTCK(ac)LIELAYASEGK_	4	0.75045	75.376	18014000	12977000
>WP_013237439.1 MULTISPECIES: hydroxylamine reductase	K	VGACGKQPDVANKQDKLTCKLIELAYASEGK	_QDK(ac)LTCK_	3	0.0112	101.64	40044000	9471700
>WP_013237439.1 MULTISPECIES: hydroxylamine reductase	K	SRIDEIINAVEVQSEKFKSDSKFHFDVLWDG	_IDEIINAVEVQSEK(ac)FK_	14	-1.9316	151.8	422600000	448410000
>WP_013237463.1 nucleotide exchange factor GrpE	K	EDDKYDKNSIVEVLQKGYKREDKIIRYSMVK	_NSIVEVLQK(ac)GYK_	9	0.19927	96.103	6033300	0
>WP_013237463.1 nucleotide exchange factor GrpE	K	NLERAVNVEGNAEDLKKGIEMTMKQFNNALS	_AVNVEGNAEDLK(ac)K_	12	0.14518	110.29	25835000	22342000
>WP_013237463.1 nucleotide exchange factor GrpE	K	KEDKEDKEISLLGELKKENSKLKDENKKAIN	_EISLLGELK(ac)K_	9	0.59903	91.549	88390000	8219300
>WP_013237464.1 MULTISPECIES: molecular chaperone DnaK	K	LGDKVSAEDKKTVEEKIEALKKIKDGEDLEA	_TVEEK(ac)IEALK_	5	0.39162	70.977	4427000	1306600
>WP_013237464.1 MULTISPECIES: molecular chaperone DnaK	K	IDIKNNADQSVYQTEKTLKDLGDKVSAEDKK	_NNADQSVYQTEK(ac)TLK_	12	0.33053	53.981	8070500	3263400
>WP_013237464.1 MULTISPECIES: molecular chaperone DnaK	K	NLSDDEINKAVDEAKKFEEQDKKRKESIDIK	_K(ac)FEEQDKK_	1	-0.00165	130.75	35594000	22894000
>WP_013237464.1 MULTISPECIES: molecular chaperone DnaK	K	INKAVDEAKKFEEQDKKRKESIDIKNNADQS	_KFEEQDK(ac)K_	7	-0.53491	146.23	38325000	23042000
>WP_013237464.1 MULTISPECIES: molecular chaperone DnaK	K	RNDKMALQRLKEAAEKAKIELSASTQTNINL	_LKEAAEK(ac)AK_	7	0.0431	103.7	52995000	49056000
>WP_013237464.1 MULTISPECIES: molecular chaperone DnaK	K	EALKKIKDGEDLEAIKKATEDLTQTFYGITS	_IKDGEDLEAIK(ac)K_	11	-0.1139	140.08	103600000	78670000
>WP_013237464.1 MULTISPECIES: molecular chaperone DnaK	K	VSFQANGERLVGQVAKRQAITNPDKTIMSIK	_LVGQVAK(ac)R_	7	-0.02138	154.68	105110000	82099000
>WP_013237464.1 MULTISPECIES: molecular chaperone DnaK	K	DDFDQKVMDYIAEDFKAKNGIDLRNDKMALQ	_VMDYIAEDFK(ac)AK_	10	0.38746	116.01	241390000	182390000
>WP_013237464.1 MULTISPECIES: molecular chaperone DnaK	K	GASEKKDDNVVDADYKVDDDK	_KDDNVVDADYK(ac)VDDDK_	11	-0.22733	212.9	360490000	387590000
>WP_013237464.1 MULTISPECIES: molecular chaperone DnaK	K	AEDFKAKNGIDLRNDKMALQRLKEAAEKAKI	_NDK(ac)M(ox)ALQR_	3	0.15406	148.38	570890000	401540000
>WP_013237464.1 MULTISPECIES: molecular chaperone DnaK	K	DATGPKHIDMNLTRAKFNELTQDLVERTIEP	_AK(ac)FNELTQDLVER_	2	-3.7344	195.88	2.408E+09	2.04E+09
>WP_013237465.1 molecular chaperone DnaJ	K	QYVNVIVDVPKDLNEKQREAIIMLMEASGEV	_DLNEK(ac)QR_	5	-0.18022	91.906	19380000	1566400
>WP_013237465.1 molecular chaperone DnaJ	K	ASDGDIKKAFRKLALKYHPDRNPNDKKAEEK	_LALK(ac)YHPDRNPNDK_	4	0.26154	144.68	118100000	156250000
>WP_013237465.1 molecular chaperone DnaJ	K	QSGDLYVNIRVSPHSKFKRKGFDIYTDTHIS	_VSPHSK(ac)FK_	6	-0.27679	96.711	199980000	126050000
>WP_013237467.1 MULTISPECIES: 16S rRNA (uracil(1498)-N(3))-methyltransferase	K	VVTSRVVVKNDSGEFKKVDRWNKIAKEACKQ	_NDSGEFK(ac)K_	7	-0.10103	66.621	5490400	3603800
>WP_013237469.1 MULTISPECIES: histidine triad nucleotide-binding protein	K	KIIAHIYLVAKQIAAKLGIDEKGYRIVTNCG	_QIAAK(ac)LGIDEK_	5	1.3455	38.58	2610100	5900600

>WP_013237469.1 MULTISPECIES: histidine triad nucleotide-binding protein	K	YLVAKQIAAKLGIDEKGYRIVTNCGEEAGQT	_LGIDEK(ac)GYR_	6	-0.51127	153.08	83381000	52915000
>WP_013237470.1 MULTISPECIES: GatB/YqeY domain-containing protein	K	SILEFEKGKRQDLVEKAKSEIDILLEYLPQQ	_RQDLVEK(ac)AK_	7	0.23252	76.228	7528400	46871000
>WP_013237470.1 MULTISPECIES: GatB/YqeY domain-containing protein	K	DGKKLDDEKIIDIIAKEVKERRESILEFEKG	_IIDIIAK(ac)EVK_	7	0.06196	56.225	13723000	7991200
>WP_013237470.1 MULTISPECIES: GatB/YqeY domain-containing protein	K	EIIRNAVNEVGAESIKDMKKVMAIVMPKTRG	_NAVNEVGAESIK(ac)DMK_	12	-0.38001	162.87	27215000	16047000
>WP_013237475.1 MULTISPECIES: GTPase Era	K	QLVFVDTPGIHKPKHKLGDYMVKAAEDSIKD	_HK(ac)LGDYMVK_	2	-1.1367	62.303	2294500	4207300
>WP_013237479.1 MULTISPECIES: pyruvate, phosphate dikinase	K	FDEDRIPEMREMIVSKTEEQRRKALDKLLPR	_EMIVSK(ac)TEEQR_	6	1.5435	49.5	0	1950900
>WP_013237479.1 MULTISPECIES: pyruvate, phosphate dikinase	K	GEDVVAGIRTPQPITKLKEDLPECYSQFMSI	_TPQPITK(ac)LK_	7	-0.57156	91.087	0	139270000
>WP_013237479.1 MULTISPECIES: pyruvate, phosphate dikinase	K	MGIEKREFEDVLDDVKNAKGVKYDTDLDESD	_EFEDVLDDVK(ac)NAK_	10	-0.37774	78.763	6361800	3934100
>WP_013237479.1 MULTISPECIES: pyruvate, phosphate dikinase	K	PGAACGKIYFTADDAKKHHDQGEKVILVRLE	_IYFTADDAK(ac)K_	9	0.1839	87.298	12135000	17209000
>WP_013237479.1 MULTISPECIES: pyruvate, phosphate dikinase	K	AKGVKYDTDLDESDLKNIIQRFKDIYKKEVK	_YDTDLDESDLK(ac)NIIQR_	11	-0.19397	63.337	16059000	16020000
>WP_013237479.1 MULTISPECIES: pyruvate, phosphate dikinase	K	QIYDALKNVEETTGKKFGSIENPLLVSVRSG	_K(ac)FGSIENPLLVSVR_	1	-0.58933	125.22	27693000	41562000
>WP_013237479.1 MULTISPECIES: pyruvate, phosphate dikinase	K	RLDQVGVGKLVETAVKLGKKTRPDIHLGICG	_LVETAVK(ac)LGK_	7	0.45392	90.108	177360000	160400000
>WP_013237479.1 MULTISPECIES: pyruvate, phosphate dikinase	K	QLDTLLHPNFDSDELKRAVVIANGLPASPGA	_QLDTLLHPNFDSDELK(ac)R_	16	0.25903	199.15	522290000	472750000
>WP_013237479.1 MULTISPECIES: pyruvate, phosphate dikinase	K	EFEDVLDDVKNAKGVKYDTDLDESDLKNIIQ	_GVK(ac)YDTDLDESDLK(ac)NIIQR_	3	0.63561	104.17	857350000	796160000
>WP_013237489.1 MULTISPECIES: SAM-dependent methyltransferase	K	SDINKGPVEKARFNIKLHGLENKIDVRLGSG	_FNIK(ac)LHGLENK_	4	-0.40118	172.17	7019700	13315000
>WP_013237490.1 MULTISPECIES: Nif3-like dinuclear metal center hexameric protein	K	MYLRVQDIHDILEKRAPSILKESYDNVGL	_VQDIHDILEK(ac)R_	10	0.29006	73.848	5865500	0
>WP_013237492.1 MULTISPECIES: pyridoxal-5'-phosphate-dependent protein subunit beta	K	MHKLIDLTINEEQLKKTAASAKEKNIIIPT	_LIDLTINEEQLK(ac)K_	12	-0.35179	144.57	28279000	33330000
>WP_013237492.1 MULTISPECIES: pyridoxal-5'-phosphate-dependent protein subunit beta	K	PEISGVKARIFAMAGKFFPTGAHKVGASFAC	_IFAMAGK(ac)FFPTGAHK_	7	-1.798	75.669	28587000	21843000
>WP_013237519.1 MULTISPECIES: 5-(carboxyamino)imidazole ribonucleotide mutase	K	EIKKKLIEYRKNMKEKFINENGKGVEL	_EK(ac)FINENGK_	2	0.76248	67.726	3206300	79379000
>WP_013237519.1 MULTISPECIES: 5-(carboxyamino)imidazole ribonucleotide mutase	K	IQYEAYILSAHRVPEKLLETVKKLEEEGCEC	_VPEK(ac)LLETVK_	4	-0.24735	118.28	30965000	15451000
>WP_013237520.1 phosphoribosylaminoimidazolesuccinocarboxamide synthase	K	FELLEKHGIETHFEQKLNDREQLCKKVEIVP	_HGIETHFEQK(ac)LNDR_	10	0.18302	68.83	13783000	9393800
>WP_013237520.1 phosphoribosylaminoimidazolesuccinocarboxamide synthase	K	LRQGIKLIDFKLEFGKFNEKILADEISPDT	_LEFGK(ac)FNEK_	5	0.59147	129.85	19658000	21660000
>WP_013237520.1 phosphoribosylaminoimidazolesuccinocarboxamide synthase	K	PLEVIVRNVAAGSMAKRLGLEEGSALKTTVY	_NVAAGSMAK(ac)R_	9	-0.51491	121.73	25033000	24078000
>WP_013237520.1 phosphoribosylaminoimidazolesuccinocarboxamide synthase	K	SINNILKEFFLRQGIKLIDFKLEFGKFNEKI	_QGIK(ac)LIDFK_	4	-0.10336	76.282	63880000	3068700
>WP_013237520.1 phosphoribosylaminoimidazolesuccinocarboxamide synthase	K	ISPDTCRLWDAKTNEKLDKDRFRRDMGNVKE	_TNEK(ac)LDKDR_	4	-0.21145	128.91	79379000	105230000
>WP_013237521.1 MULTISPECIES: amidophosphoribosyltransferase	K	VVIVDDSIVRGTTSGKLVEILRKAGATEVHF	_GTTSGK(ac)LVEILR_	6	0.77098	71.241	4244700	6654700

>WP_013237521.1 MULTISPECIES: amidophosphoribosyltransferase	K	FIAPSQELRSKAVAVKLNPLKVNVEGKRVVI	_AVAVK(ac)LNPLK_	5	-0.15386	110.87	66864000	2868400
>WP_013237522.1 MULTISPECIES: phosphoribosylformylglycinamidine cyclo-ligase	K	CVDNKDAKNVVEELNKMGEKAYIIGSVKKGE	_NVVEELNK(ac)MGEK_	8	-0.12056	63.727	3329400	0
>WP_013237522.1 MULTISPECIES: phosphoribosylformylglycinamidine cyclo-ligase	K	ELNKMGEKAYIIGSVKKGEKAVCLK	_AYIIGSVK(ac)K_	8	0.61947	78.934	4938300	4814100
>WP_013237522.1 MULTISPECIES: phosphoribosylformylglycinamidine cyclo-ligase	K	HITGGGFFENIPRMFKDDFTAVIDKKSFEVP	_MFK(ac)DDFTAVIDKK_	3	-0.25546	49.929	8096800	6800000
>WP_013237522.1 MULTISPECIES: phosphoribosylformylglycinamidine cyclo-ligase	K	KVLLTPTKIYVKPVLKLLDKFKIKAMAHITG	_IYVKPVLK(ac)LLDK_	8	-0.00904	89.959	39608000	60025000
>WP_013237524.1 MULTISPECIES: bifunctional phosphoribosylaminoimidazolecarboxamide formyltransferase/IMP cyclohydrolase PurH	K	RVKTLHPVIHGGILAKRDNKEHMDTIKSKNI	_TLHPVIHGGILAK(ac)R_	13	-0.44751	34.685	0	2638300
>WP_013237524.1 MULTISPECIES: bifunctional phosphoribosylaminoimidazolecarboxamide formyltransferase/IMP cyclohydrolase PurH	K	IVIAPDFDKDAVEILKAKKNLRIIKCDVKPT	_DAVEILK(ac)AK_	7	-1.3142	66.435	6988800	5881500
>WP_013237524.1 MULTISPECIES: bifunctional phosphoribosylaminoimidazolecarboxamide formyltransferase/IMP cyclohydrolase PurH	K	GILAKRDNKEHMDTIKSKNIKPIDMVVVNLY	_EHMDTIK(ac)SK_	7	0.35732	72.607	20571000	20297000
>WP_013237525.1 phosphoribosylamine--glycine ligase	K	ESAIYRKLDKFNLEWKKGASCCVVAASKGYP	_FNLEWK(ac)K_	6	-0.683	82.671	10207000	8519500
>WP_013237525.1 phosphoribosylamine--glycine ligase	K	TVVGPEGPLTEGIVDKFKSKGLKIFGPSLKA	_ENIDLTVVGPEGPLTEGIVDK(ac)FK_	21	-1.2586	37.896	20661000	5815800
>WP_013237541.1 hypothetical protein	K	KQFLEELKNKNLEEEKKLEELKNKKLEEQKK	_NLEEEK(ac)K_	6	0.4192	89.296	9833900	8597000
>WP_013237558.1 formate dehydrogenase subunit alpha	K	YTPEYVEKIVNVPAEKIKKAMEMYASAKNPM	_IVNVPAEK(ac)IK_	8	-0.13528	88.681	0	7993800
>WP_013237558.1 formate dehydrogenase subunit alpha	K	GYHLTEVPKAVLEEHKLKAYYIMGEDCVQSD	_AVLEEHK(ac)LK_	7	-0.55459	55.567	1978900	2065400
>WP_013237558.1 formate dehydrogenase subunit alpha	K	NNTKEIWDEMRSLSPKFAGATYEKMETLDGV	_SLSPK(ac)FAGATYEK_	5	-0.30872	101.32	55824000	66407000
>WP_013237565.1 formylmethanofuran dehydrogenase subunit E	K	EKLFYYKKPGFSVPEKARSFNNIVCENCGED	_KPGFSVPEK(ac)AR_	9	0.60109	68.132	6628700	5841000
>WP_013237565.1 formylmethanofuran dehydrogenase subunit E	K	SFFNRKNGDALRVVLKNWPRSGDRQKDIDYL	_VVLK(ac)NWPR_	4	-0.39547	146.94	105230000	8972200
>WP_013237567.1 MULTISPECIES: cytochrome c biogenesis protein	K	EECKVEFCCKSDIVEKAISTIRKVHPYEEPV	_SDIVEK(ac)AISTIR_	6	-0.36769	39.016	2677900	0
>WP_013237569.1 (Fe-S)-binding protein	K	EDDIYSIKDLSTLIEKKFNVNGEELVRKMYK	_DLSTLIEK(ac)K_	8	1.4838	76.465	2868400	4463100
>WP_013237573.1 3-isopropylmalate dehydrogenase	K	RITRKAFEIARKRNNKLTLVDKANVLESSKL	_NNK(ac)LTLVDK_	3	0.15843	58.699	5140600	5282400
>WP_013237573.1 3-isopropylmalate dehydrogenase	K	LLGAVGGPKWDSLPSKLRPEAGLLGIRKALG	_WDSLPSK(ac)LRPEAGLLGIR_	7	0.72473	56.407	22065000	11504000
>WP_013237575.1 nitrate reductase subunit beta	K	HNENGKSFYDLDLSNKFLESRNHISKDDSGN	_SFYDLDLSNK(ac)FLESR_	10	0.38167	118.33	32935000	26961000
>WP_013237576.1 carbon-monoxide dehydrogenase catalytic subunit	K	EKISNHVIDTAIESYKERRSKVTMNIPKNHG	_ISNHVIDTAIESYK(ac)ER_	14	-0.1641	62.377	0	8568600
>WP_013237576.1 carbon-monoxide dehydrogenase catalytic subunit	K	LGGSWKPLVDLIAAGKIKGVAGIVGCSNLTA	_SFLGGSWKPLVDLIAAGK(ac)IK_	18	-4.1166	200.27	0	157470000
>WP_013237576.1 carbon-monoxide dehydrogenase catalytic subunit	K	KDLYKPKFEKMEVINKLAYAPRLENWNKLNI	_MEVINK(ac)LAYAPR_	6	1.388	56.225	2549200	0
>WP_013237576.1 carbon-monoxide dehydrogenase catalytic subunit	K	SDPHKKAVLVADAVLKDLYKPKFEKMEVINK	_AVLVADAVLK(ac)DLYKPK_	10	0.82117	81.311	15212000	5699900

>WP_013237576.1 carbon-monoxide dehydrogenase catalytic subunit	K	NTLAEKLGITESDPHKKAVLVADAVLKDLYK	_LGITESDPHK(ac)K_	10	0.23604	48.741	19552000	13131000
>WP_013237576.1 carbon-monoxide dehydrogenase catalytic subunit	K	GGEIKGMNALNTLAEKLGITESDPHKKAVLV	_GMNALNTLAEK(ac)LGITESDPHK_	11	-0.55908	105.32	170830000	87805000
>WP_013237576.1 carbon-monoxide dehydrogenase catalytic subunit	K	MSNNKICKSADKVLEKFIGSLDGVETSHHRV	_VLEK(ac)FIGSLDGVETSHHR_	4	0.04586	148.44	271580000	397840000
>WP_013237586.1 MULTISPECIES: lipoate--protein ligase A	K	EENPIKEMEDLFIDKKFNIEDIKNIIEKNDV	_K(ac)FNIEDIK_	1	-0.18324	58.981	5235000	6889000
>WP_013237586.1 MULTISPECIES: lipoate--protein ligase A	K	VTNISEHIDKGLSLIKFKDMLIQCFFESRKE	_GLSLIK(ac)FK_	6	-0.13949	119.89	23676000	18750000
>WP_013237586.1 MULTISPECIES: lipoate--protein ligase A	K	NNKYGTWEWNFGKSPKFNYNKEMRFDAGSIC	_SPK(ac)FNYNK_	3	-0.02854	157.68	56136000	47164000
>WP_013237593.1 MULTISPECIES: ornithine carbamoyltransferase	K	EKVIFEHCLPAFHDLKTKVGKQIHDKFGLNE	_VIFEHCLPAFHDLK(ac)TK_	14	0.19038	93.51	25995000	16787000
>WP_013237597.1 MULTISPECIES: TrmB family transcriptional regulator	K	MELEKELIAKLVKIGFNKYEAMIYL	_ELIAK(ac)LVK_	5	0.11417	68.915	11489000	14680000
>WP_013237597.1 MULTISPECIES: TrmB family transcriptional regulator	K	WSKDYNNLYDELLDAKKRNIKIVSVLYGKVD	_DYNNLYDELLDAK(ac)K_	13	-0.1298	77.42	11535000	25559000
>WP_013237599.1 pyruvate:ferredoxin (flavodoxin) oxidoreductase	K	IELLEYEELAKLVDQKALNDFRRRALNPDHP	_LVDQK(ac)ALNDFR_	5	-1.1345	39.917	1372300	9384600
>WP_013237599.1 pyruvate:ferredoxin (flavodoxin) oxidoreductase	K	FVNFFDGFRTSHEIQKIELLEYEELAKLVDQ	_TSHEIQK(ac)IELLEYEELAK_	7	-0.04143	55.33	7860300	13612000
>WP_013237599.1 pyruvate:ferredoxin (flavodoxin) oxidoreductase	K	TRERADKITALLEKEKGSNDLLNDIYENRDF	_EK(ac)GSNDLLNDIYENR_	2	-2.9688	85.619	9337800	0
>WP_013237599.1 pyruvate:ferredoxin (flavodoxin) oxidoreductase	K	IKNFAEGEGTRERADKITALLEKEKGSNDLL	_ERADK(ac)ITALLEK_	5	0.47337	110.38	15925000	12470000
>WP_013237599.1 pyruvate:ferredoxin (flavodoxin) oxidoreductase	K	SLAKQFPDQADALFEKTKKDALQRIAGYKKL	_QFPDQADALFEK(ac)TK_	12	-0.30244	92.063	16032000	10734000
>WP_013237599.1 pyruvate:ferredoxin (flavodoxin) oxidoreductase	K	EDEAAATKELPKFIEKIVNPMNRQEGDKLPV	_FIEK(ac)IVNPM(ox)NR_	4	0.02235	109.42	25131000	30010000
>WP_013237599.1 pyruvate:ferredoxin (flavodoxin) oxidoreductase	K	HAAIRPVLTTEEELAKAPQGFEAKDANGAKG	_PVLTTEEELAK(ac)APQGFEAK_	11	-1.5298	88.427	25227000	0
>WP_013237599.1 pyruvate:ferredoxin (flavodoxin) oxidoreductase	K	YWALYRYNPELKEEGKKSFSLDSKEPTTDFK	_YNPELKEEGK(ac)K_	10	-0.0136	77.64	25255000	14066000
>WP_013237599.1 pyruvate:ferredoxin (flavodoxin) oxidoreductase	K	CKFWGLGSDGTVGANKSAIKIIGDHTDMYAQ	_FWGLGSDGTVGANK(ac)SAIK_	14	0.09419	107.75	38658000	87107000
>WP_013237599.1 pyruvate:ferredoxin (flavodoxin) oxidoreductase	K	DMNNAAIDKGVNAVVKIDVPASWKDTEDEAA	_GVNAVVK(ac)IDVPASWK_	7	0.71109	141.85	41636000	53804000
>WP_013237599.1 pyruvate:ferredoxin (flavodoxin) oxidoreductase	K	NCEDVCPAKEKALVMKPVDTQLSKTEAWDYA	_ALVMK(ac)PVDTQLSK_	5	-0.56104	93.348	45240000	45844000
>WP_013237599.1 pyruvate:ferredoxin (flavodoxin) oxidoreductase	K	GSLSAELKAAFEDWIKNFAEGEGTRERADKI	_AAFEDWIK(ac)NFAEGEGTR_	8	-0.78444	113.29	47597000	32173000
>WP_013237599.1 pyruvate:ferredoxin (flavodoxin) oxidoreductase	K	KGVNAVVKIDVPASWKDTEDEAAATKELPKF	_IDVPASWK(ac)DTEDEAAATK_	8	-0.21068	136.2	58279000	48063000
>WP_013237599.1 pyruvate:ferredoxin (flavodoxin) oxidoreductase	K	VPASWKDTEDEAAATKELPKFIEKIVNPMNR	_DTEDEAAATK(ac)ELPK(ac)FIEK_	10	-0.02705	193.64	62454000	77189000
>WP_013237599.1 pyruvate:ferredoxin (flavodoxin) oxidoreductase	K	VDCGYWALYRYNPELKEEGKKSFSLDSKEPT	_YNPELK(ac)EEGK_	6	-0.09857	110.84	80736000	70696000
>WP_013237599.1 pyruvate:ferredoxin (flavodoxin) oxidoreductase	K	HNQSYVHKYDVLAGLKKGGNFLLNTVWTQEE	_YDVLAGLK(ac)K_	8	0.50914	92.062	82396000	88776000
>WP_013237599.1 pyruvate:ferredoxin (flavodoxin) oxidoreductase	K	GMFLGVKQTRERLQDKIEEALKGSLSAELKA	_LQDK(ac)IEEALK_	4	0.39192	138.22	112750000	105530000
>WP_013237599.1 pyruvate:ferredoxin (flavodoxin) oxidoreductase	K	LTVHLYRPFSIKHFMKYIPKTVKKIAVLDRT	_HFMK(ac)YIPK_	4	-0.24882	91.9	167740000	165260000
>WP_013237599.1 pyruvate:ferredoxin (flavodoxin) oxidoreductase	K	EGTRERADKITALLEKEKGSNDLLNDIYENR	_ITALLEK(ac)EK_	7	0.11608	135.58	267840000	185660000
>WP_013237599.1 pyruvate:ferredoxin (flavodoxin) oxidoreductase	K	HEIQKIELLEYEELAKLVDQKALNDFRRRAL	_IELLEYEELAK(ac)LVDQK_	11	1.5454	139.34	1.327E+09	282970000
>WP_013237599.1 pyruvate:ferredoxin (flavodoxin) oxidoreductase	K	WKDTEDEAAATKELPKFIEKIVNPMNRQEGD	_ELPK(ac)FIEK(ac)IVNPMNR_	4	-2.594	193.64	4.713E+09	5.448E+09
>WP_013237602.1 MULTISPECIES: hypothetical protein	K	VENVKFDSSNNKVLLKIIKAYDKEVRDDIKN	_VLLK(ac)IIK_	4	0.1508	72.234	8723200	6531100

>WP_013237602.1 MULTISPECIES: hypothetical protein	K	KNSAKITNLRLWLNEKDIKNSILKLIKANEI	_LWLNEK(ac)DIK_	6	0.54114	82.749	9903300	0
>WP_013237602.1 MULTISPECIES: hypothetical protein	K	KFFLYNNELKNKEQFKEAEFKHGKSIYEVIR	_NKEQFK(ac)EAEFK_	6	-0.19218	119.74	19734000	27948000
>WP_013237602.1 MULTISPECIES: hypothetical protein	K	GTSPKVLPIKKVENVKFDSSNNKVLLKIIKA	_VENVK(ac)FDSSNNK_	5	0.01324	257.8	58123000	126760000
>WP_013237603.1 DUF342 domain-containing protein	K	KSKKDGEIIKILLESKFKKLIKLGINVIADL	_ILLESK(ac)FK_	6	0.19706	98.392	16238000	11763000
>WP_013237606.1 MULTISPECIES: chemotaxis response regulator protein-glutamate methylesterase	K	VDLVLPIDKIANEIVKIIKFGGR	_IANEIVK(ac)IIK_	7	-0.33384	70.942	4804600	5191200
>WP_013237606.1 MULTISPECIES: chemotaxis response regulator protein-glutamate methylesterase	K	VLPIDKIANEIVKIIKFGGR	_IIK(ac)FGGR_	3	0.0246	74.717	7662000	2247900
>WP_013237607.1 MULTISPECIES: protein-glutamate O-methyltransferase CheR	K	NVSEFFRNPEIFEELKVRLKEELLPRNSPLK	_NPEIFEELK(ac)VR_	9	-0.33523	101.39	15150000	15831000
>WP_013237608.1 MULTISPECIES: chemotaxis protein CheA	K	SASNKNYIVIVKVGEKTVGLMVDSLLGQREI	_VGEK(ac)TVGLMVDSLLGQR_	4	-0.49143	42.813	7248000	2259300
>WP_013237608.1 MULTISPECIES: chemotaxis protein CheA	K	IIYNGNVIPLIRLYEKLGVEKSASNKNYIVI	_LYEK(ac)LGVEK_	4	-0.17524	76.282	21560000	16644000
>WP_013237608.1 MULTISPECIES: chemotaxis protein CheA	K	EVTDISGRGVGMDVVKTKISSLGGTVELVSE	_GVGMDVVK(ac)TK_	8	-0.15288	69.979	22031000	25068000
>WP_013237608.1 MULTISPECIES: chemotaxis protein CheA	K	AVIKVEDDGSGIPVEKIREKAENAGINTEGM	_VEDDGSGIPVEK(ac)IR_	12	-0.17159	159.02	58821000	46788000
>WP_013237613.1 MULTISPECIES: flagellar motor switch phosphatase FliY	K	APAQSQMEQRTPVYEKPIETNVNKQPVEVSR	_TPVYEK(ac)PIETNVNK_	6	-0.28633	67.275	22993000	22832000
>WP_013237614.1 MULTISPECIES: flagellar biosynthesis anti-sigma factor FlgM	K	VANGTYKVDSKLVAQKIIDNIKGKV	_LVAQK(ac)IIDNIK_	5	0.20667	143.5	9622000	0
>WP_013237620.1 flagellar protein	K	DVKKAVDKLNKLLEDKATHLKYEVCGKFNDI	_LLEDK(ac)ATHLK_	5	-0.08431	44.309	2914400	3410300
>WP_013237636.1 flagellin ;>WP_013237628.1 MULTISPECIES: flagellin protein	K	RHMNSNTNAASKSMQKLSSGLRINSAADDAA	_SMQK(ac)LSSGLR_	4	1.0877	65.5	9184500	8606400
>WP_013237640.1 MULTISPECIES: glycosyltransferase family 1 protein	K	GTPSKRREKIIQILEKKFNIVYTQAFGEEMT	_IIQILEK(ac)K_	7	0.4438	69.825	1220200	0
>WP_013237676.1 MULTISPECIES: DUF3867 domain-containing protein	K	MEKNNISQEKLFNIQKKLMERYGFNMEDVEK	_LFNIQK(ac)K_	6	0.27967	113.25	11541000	16855000
>WP_013237678.1 MULTISPECIES: hypothetical protein	K	TTHLEQIKKSVCIIEKLYKNIEVIGLWINEN	_SVCIIEK(ac)LYK_	7	0.52285	66.595	9594000	0
>WP_013237678.1 MULTISPECIES: hypothetical protein	K	CAANKVDKTTHLEQIKKSVCIIEKLYKNIEV	_TTHLEQIK(ac)K_	8	-0.32934	68.626	14800000	8955100
>WP_013237681.1 methyl-accepting chemotaxis protein	K	ENLNSTSKNLINVVNKFKVE	_NLINVVNK(ac)FK_	8	0.67835	99.653	6508900	0
>WP_013237682.1 MULTISPECIES: hypothetical protein	K	MSEHEKVFIPKESIDKLVSGLKSIRITTREK	_ESIDK(ac)LVSGLK_	5	-0.27199	38.96	4975400	0
>WP_013237682.1 MULTISPECIES: hypothetical protein	K	VFIPKESIDKLVSGLKSIRITTREKATRKEV	_LVSGLK(ac)SIR_	6	-0.87656	70.908	8527000	6653000
>WP_013237692.1 flagellar hook-length control protein FliK	K	LKSQIYTELLQKLNPKLADNLNSGKNVSTNN	_LNPK(ac)LADNLNSGK_	4	-0.08906	63.76	4713100	4394400
>WP_013237696.1 MULTISPECIES: flagellar protein FlbD	K	GKKYLVKESNEEIVEKVIKYKRKIYTGNFD	_ESNEEIVEK(ac)VIK_	9	-1.1525	88.021	15264000	9424700
>WP_013237705.1 MULTISPECIES: flagellar protein	K	IPVNNHKYMPLSKGEKVHVIYYDGKNVYKFS	_GEK(ac)VHVIYYDGK_	3	-0.56428	94.728	27262000	8832300
>WP_013237712.1 MULTISPECIES: manganese-dependent inorganic pyrophosphatase	K	KVGVGGQISTMDTEGFKSIRKDIIKVMESKCK	_VGVGGQISTMDTEGFK(ac)SIR_	15	-0.25204	81.317	23828000	23425000
>WP_013237717.1 MULTISPECIES: redox-regulated ATPase YchF	K	AGLVKGASKGEGLGNKFLSHIREVEAIVHVV	_GEGLGNK(ac)FLSHIR_	7	0.4515	165.7	44922000	45457000
>WP_013237723.1 MULTISPECIES: UDP-N-acetylmuramoyl-tripeptide--D-alanyl-D-alanine ligase	K	IVEGDTVLIKASRVMKFETIAEELKKNSNN	_VMK(ac)FETIAEELK_	3	-0.45987	37.426	1997400	5820900

>WP_013237730.1 YggS family pyridoxal phosphate-dependent enzyme	K	ELKDKNVTLVAVSKTKPIEDIQKAYDAGIRD	_NVTLVAVSK(ac)TKPIEDIQK_	11	-0.03437	92.977	0	13291000
>WP_013237730.1 YggS family pyridoxal phosphate-dependent enzyme	K	LIHSLDSIRLLNELEKHYKDQKLIANTLIQI	_LLNELEK(ac)HYK_	7	-0.64188	94.297	12585000	8849200
>WP_013237730.1 YggS family pyridoxal phosphate-dependent enzyme	K	VIPKGDEESCKNYFQKLKSIFDGLKKREFNN	_NYFQK(ac)LK_	5	0.24847	116.35	37943000	1123600
>WP_013237730.1 YggS family pyridoxal phosphate-dependent enzyme	K	EEELKDKNVTLVAVSKTKPIEDIQKAYDAGI	_NVTLVAVSK(ac)TKPIEDIQK_	9	0.87603	208.2	51393000	42021000
>WP_013237734.1 MULTISPECIES: DivIVA domain-containing protein	K	NKEDSNHTEKEIFEDKDELEEIKNFFIKG	_EIFEDK(ac)DELEEIK_	6	-0.40834	76.262	25104000	27825000
>WP_013237734.1 MULTISPECIES: DivIVA domain-containing protein	K	IQITDEFENVKQEFSKFRTKFRNFMKTQMEM	_QEFSK(ac)FR_	5	-0.10256	148.07	100210000	86985000
>WP_013237734.1 MULTISPECIES: DivIVA domain-containing protein	K	DEFLDKIAEDYESIYKENSLLKEKMQSLDEK	_IAEDYESIYK(ac)ENSLLK_	10	0.51412	126.16	102160000	185110000
>WP_013237735.1 MULTISPECIES: 5'-methylthioadenosine/adenosylhomocysteine nucleosidase	K	VGRIVTGDQFISSTEKVRYLNKEFGALACEM	_IVTGDQFISSTEK(ac)VR_	13	0.55082	45.864	8234300	7071100
>WP_013237735.1 MULTISPECIES: 5'-methylthioadenosine/adenosylhomocysteine nucleosidase	K	RSISDNANNGASMDYKKFAPIAIKNSTQIIK	_SISDNANNGASMDYK(ac)K_	15	-0.14708	61.097	18693000	15541000
>WP_013237739.1 MULTISPECIES: bifunctional pyr operon transcriptional regulator/uracil phosphoribosyltransferase PyrR	K	LEKNRGTEDIILVGIKRRGYPLAKRIAENIY	_GTEDIILVGIK(ac)R_	11	0.95349	78.692	6607200	9370400
>WP_013237739.1 MULTISPECIES: bifunctional pyr operon transcriptional regulator/uracil phosphoribosyltransferase PyrR	K	AMNRTLKRISHEILEKNRGTEDIILVGIKRR	_ISHEILEK(ac)NR_	8	0.52292	100.48	13401000	9894300
>WP_013237739.1 MULTISPECIES: bifunctional pyr operon transcriptional regulator/uracil phosphoribosyltransferase PyrR	K	DIILVGIKRRGYPLAKRIAENIYNIEGIKLK	_RGYPLAK(ac)R_	7	1.0205	160.54	14699000	24608000
>WP_013237739.1 MULTISPECIES: bifunctional pyr operon transcriptional regulator/uracil phosphoribosyltransferase PyrR	K	MKLKALILDEKAMNRTLKRISHEILE	_ALILDEK(ac)AMNR_	7	-3.3821	104.52	22870000	8076000
>WP_013237765.1 MULTISPECIES: aminopeptidase P family protein	K	DKAFFITDSRFTEQAKQQVKDYEVLEYGKSG	_FTEQAK(ac)QQVK_	6	-0.03033	52.49	4800300	2907600
>WP_013237765.1 MULTISPECIES: aminopeptidase P family protein	K	KEEINSIKNAAKIADKAFDHMVKFIKSGMTE	_IADK(ac)AFDHMVK_	4	-0.79206	142.1	59025000	47108000
>WP_013237765.1 MULTISPECIES: aminopeptidase P family protein	K	GKSGSFVKFLSDLIGKLKVHRLGFEENIVSY	_FLSDLIGK(ac)LK_	8	0.31079	95.573	183900000	186300000
>WP_013237776.1 MULTISPECIES: Asp23/Gls24 family envelope stress response protein	K	GVRIPDVALKVQENVKRAVQSMTGLDVSAIN	_VQENVK(ac)R_	6	-0.27512	139.92	33350000	28449000
>WP_013237776.1 MULTISPECIES: Asp23/Gls24 family envelope stress response protein	K	YVVVEYGVRIPDVALKVQENVKRAVQSMTGL	_IPDVALK(ac)VQENVK_	7	0.25858	161.21	95813000	90589000
>WP_013237777.1 MULTISPECIES: transcription antitermination factor NusB	K	KETDGAEEVYSENIDKLKNVDMEYVKRVVKG	_ETDGAEEVYSENIDK(ac)LK_	15	-0.02497	84.14	20674000	11092000
>WP_013237778.1 MULTISPECIES: exodeoxyribonuclease VII large subunit	K	NKKDKIKVIMKDGTSKFKLIHY	_DGTSK(ac)FK_	5	0.34932	73.324	16425000	17564000
>WP_013237781.1 MULTISPECIES: 1-deoxy-D-xylulose-5-phosphate synthase	K	GSSNTYSKAFGNEMVKLAEKDDRIVAITAAM	_AFGNEMVK(ac)LAEK_	8	1.7755	52.555	6501900	3513800
>WP_013237781.1 MULTISPECIES: 1-deoxy-D-xylulose-5-phosphate synthase	K	SAKDIQGPVIIHIITKKGKGYEFAEKNPGKF	_DIQGPVIIHIITK(ac)K_	13	-0.13334	77.746	24414000	29855000
>WP_013237781.1 MULTISPECIES: 1-deoxy-D-xylulose-5-phosphate synthase	K	TAAMRDGTGLKSFSQKFPERFFDVGIAEQHA	_SFSQK(ac)FPER_	5	0.19746	126.07	51299000	38303000
>WP_013237783.1 MULTISPECIES: NAD(+) kinase	K	NTIEVRRSKNKRKFIKFDDNNYFDTLKDKIN	_FIK(ac)FDDNNYFDTLK_	3	-0.86896	64.394	8853900	5514800
>WP_013237784.1 MULTISPECIES: arginine repressor	K	KELKLIKVLSDSGKYKYATISHTKNFLSNKL	_YK(ac)YATISHTK_	2	-0.07384	88.596	17315000	5467300

>WP_013237784.1 MULTISPECIES: arginine repressor	K	FVMTRDGESAHIITQKMKKMISQ	_DGESAHIITQK(ac)MK_	11	-0.40672	92.19	41956000	26756000
>WP_013237789.1 MULTISPECIES: NUDIX hydrolase	K	NIYKGKIIDVNVHTVKLPNGRESKREIVNHC	_IIDVNVHTVK(ac)LPNGR_	10	0.21738	102.5	14123000	22213000
>WP_013237801.1 MULTISPECIES: electron transport complex subunit RsxC	K	IGTTFKDAIDFCGGFKEEPVKIISGGPMMGF	_DAIDFCGGFK(ac)EEPVK_	10	0.26878	46.964	2400000	1875000
>WP_013237801.1 MULTISPECIES: electron transport complex subunit RsxC	K	KAFEGTKVQVVGLPTKYPQGAEKMLINVLTG	_VQVVGLPTK(ac)YPQGAEK_	9	-4.8684	32.815	9248200	6167500
>WP_013237801.1 MULTISPECIES: electron transport complex subunit RsxC	K	_MLKSFRGGVHPDDSKKYTANKPIEIAPIPD	_GGVHPDDSK(ac)K_	9	-0.07407	61.353	10641000	11986000
>WP_013237801.1 MULTISPECIES: electron transport complex subunit RsxC	K	KYTANKPIEIAPIPDKVFIPVRQHIGAPTSP	_YTANKPIEIAPIPDK(ac)VFIPVR_	15	0.7112	94.632	76047000	59336000
>WP_013237801.1 MULTISPECIES: electron transport complex subunit RsxC	K	AGIVGMGGATFPVHVKLAPPPDKKVDVFILN	_EAGIVGMGGATFPVHVK(ac)LAPPPDK_	17	-0.90157	102.07	76378000	79834000
>WP_013237801.1 MULTISPECIES: electron transport complex subunit RsxC	K	VVAGVQIIMKILNVEKAFVGIEDNKPKAIEA	_ILNVEK(ac)AFVGIEDNKPK_	6	0.23975	138.39	553450000	444040000
>WP_013237802.1 MULTISPECIES: NADH:ubiquinone oxidoreductase	K	MAEAQIKKNIFTISSSPHVRCD	_(ac)AEAQIK(ac)K_	6	0.44295	66.258	10475000	8618500
>WP_013237806.1 MULTISPECIES: 4Fe-4S dicluster domain-containing protein	K	NLCVPGKAAVAEQVAKLTGKSAPPIEPRVAH	_AAVAEQVAK(ac)LTGK_	9	-0.31391	120.84	100940000	102580000
>WP_013237815.1 serine hydroxymethyltransferase	K	KTEYKEYIDQVVKNAKILGEELTKYGFRLVS	_NAK(ac)ILGEELTK_	3	0.0559	46.955	17764000	13836000
>WP_013237815.1 serine hydroxymethyltransferase	K	MGSFLTNKYAEGYPGKRYYGGCFVVDKVENL	_YAEGYPGK(ac)R_	8	0.21687	114.89	34513000	34503000
>WP_013237815.1 serine hydroxymethyltransferase	K	EALKTEYKEYIDQVVKNAKILGEELTKYGFR	_EYIDQVVK(ac)NAK_	8	0.2662	117.71	36072000	31010000
>WP_013237819.1 phosphoribosylformylglycinamidine synthase	K	VSHGEGRFIASDSVIKNLKDKGQIATQYVDF	_FIASDSVIK(ac)NLK_	9	-0.01262	55.452	10929000	8749600
>WP_013237819.1 phosphoribosylformylglycinamidine synthase	K	CEAVSKMSFGNRIGMKFTEPMKGEEIFAPDY	_IGMK(ac)FTEPMK_	4	-0.51416	71.221	17842000	15347000
>WP_013237821.1 MULTISPECIES: alcohol dehydrogenase	K	AIYKGIGKIELEEIAKPKVEKGTVLIKVKAC	_IELEEIAK(ac)PK_	8	0.00217	57.348	4318900	7828300
>WP_013237821.1 MULTISPECIES: alcohol dehydrogenase	K	IGKIELEEIAKPKVEKGTVLIKVKACAICGG	_VEK(ac)GTVLIK_	3	-0.07578	54.608	6885600	7599900
>WP_013237821.1 MULTISPECIES: alcohol dehydrogenase	K	HEIAGEIVETASDIEKYKVGDRVIVAPAIGC	_SIKPPIVLGHEIAGEIVETASDIEK(ac)YK_	25	0.56808	137.9	57322000	85532000
>WP_013237823.1 3-phosphoglycerate dehydrogenase	K	VHARLSEETKGLIGEKELSLMKSTAYLINTA	_GLIGEK(ac)ELSLMK_	6	0.83924	53.252	5579000	7512200
>WP_013237823.1 3-phosphoglycerate dehydrogenase	K	SYIGVCRGGVENVDVKAAKAKGIKVMRTMGR	_GGVENVDVK(ac)AAK_	9	-0.06888	96.103	27896000	26745000
>WP_013237823.1 3-phosphoglycerate dehydrogenase	K	PASDDEFWYLRSQVEKLGPSAGKPPKELDEA	_SQVEK(ac)LGPSAGKPPK_	5	-0.57329	78.285	28012000	15542000
>WP_013237827.1 MULTISPECIES: 2-hydroxyacid dehydrogenase	K	MNFASRNNVQYVTIEKILKEADFISIHLPLT	_NNVQYVTIEK(ac)ILK_	10	1.3331	79.633	7161600	5080300
>WP_013237831.1 MULTISPECIES: ATP phosphoribosyltransferase regulatory subunit	K	GMEVIERASHLMEDNKRALKALESIKKVYEI	_ASHLMEDNK(ac)R_	9	-0.20726	82.749	46141000	30320000
>WP_013237833.1 MULTISPECIES: histidinol dehydrogenase	K	LERQVQSLERKDIILKSLKDRGAIIIVDSIE	_DIILK(ac)SLK_	5	0.34421	68.224	8073800	6946200
>WP_013237833.1 MULTISPECIES: histidinol dehydrogenase	K	KEVTKSNIKVTQEEIKKAYEFVEDEFIDALK	_VTQEEIK(ac)K_	7	0.7666	119.27	10951000	9049800
>WP_013237835.1 MULTISPECIES: imidazole glycerol phosphate synthase subunit HisH	K	EAIILPGVGAFPEAMKNLKKRNLDKVLKKEA	_SEAIILPGVGAFPEAMK(ac)NLK_	17	0.20554	38.017	4627800	4310500

>WP_013237835.1 MULTISPECIES: imidazole glycerol phosphate synthase subunit HisH	K	IEECEGLGLIPGKITKLYGDIKIPHMGWNNL	_ITK(ac)LYGDIK_	3	-0.03271	107.21	20169000	18157000
>WP_013237835.1 MULTISPECIES: imidazole glycerol phosphate synthase subunit HisH	K	VDYGMGNLKSVQKALKYIGTDSKITSDQREI	_ALK(ac)YIGTDSK_	3	0.63565	93.058	25073000	22516000
>WP_013237836.1 MULTISPECIES: 1-(5-phosphoribosyl)-5-[(5-phosphoribosylamino)methylideneamino]imidazole-4-carboxamide isomerase	K	SEIVGEDPVKVALSFKKSGAEYIHMVDLDGA	_VALSFK(ac)K_	6	0.13048	105.39	0	13365000
>WP_013237836.1 MULTISPECIES: 1-(5-phosphoribosyl)-5-[(5-phosphoribosylamino)methylideneamino]imidazole-4-carboxamide isomerase	K	ALKKYGEKIAVGIDAKNKKVAVNGWLNVSSV	_IAVGIDAK(ac)NKK_	8	-0.12932	91.855	4872400	11071000
>WP_013237836.1 MULTISPECIES: 1-(5-phosphoribosyl)-5-[(5-phosphoribosylamino)methylideneamino]imidazole-4-carboxamide isomerase	K	KIKNIEVISKVIDAIKIPVELGGGIRDIKTI	_VIDAIK(ac)IPVELGGGIR_	6	-0.12037	71.692	15745000	13993000
>WP_013237836.1 MULTISPECIES: 1-(5-phosphoribosyl)-5-[(5-phosphoribosylamino)methylideneamino]imidazole-4-carboxamide isomerase	K	AIDLRNGKCVRLYQGKFEKSEIVGEDPVKVA	_LYQGK(ac)FEK_	5	-0.0335	69.812	15803000	18913000
>WP_013237836.1 MULTISPECIES: 1-(5-phosphoribosyl)-5-[(5-phosphoribosylamino)methylideneamino]imidazole-4-carboxamide isomerase	K	IEIGISRVILGTAALKNPDFAKEALKKYGEK	_VILGTAALK(ac)NPDFAK_	9	0.16892	54.598	32476000	7727500
>WP_013237837.1 MULTISPECIES: histidinol-phosphate transaminase	K	NIIPSKSNFIFISHAKYPAKELFIKLREKGV	_SNFIFISHAK(ac)YPAK_	10	-0.12901	68.044	10036000	10555000
>WP_013237837.1 MULTISPECIES: histidinol-phosphate transaminase	K	KCVSKVINTRETTVNKLEKMDFNIIPSKSNF	_ETTVNK(ac)LEK_	6	0.54563	54.539	13338000	9201500
>WP_013237837.1 MULTISPECIES: histidinol-phosphate transaminase	K	EPYVCGEQPKDKKYVKLNTNESPYPPSPKVI	_YVK(ac)LNTNESPYPPSPK_	3	-1.7036	79.771	17637000	25876000
>WP_013237837.1 MULTISPECIES: histidinol-phosphate transaminase	K	YPVYANLYNLNYKLAKLNEDFSIPFKEFLTE	_LAK(ac)LNEDFSIPFK_	3	0.03472	61.212	19212000	17656000
>WP_013237842.1 MULTISPECIES: DNA-binding response regulator	K	INTKNHQVKVGNKDVKLTPREFEILELLSSN	_DVK(ac)LTPR_	3	-0.34982	76.899	11914000	10266000
>WP_013237844.1 MULTISPECIES: hypothetical protein	K	MPSTSEISETISNMEKKCETEVLNGKVVFKN	_KQDMPSTSEISETISNMEK(ac)K_	19	-1.6745	66.874	14039000	0
>WP_013237844.1 MULTISPECIES: hypothetical protein	K	DNNYFLELKADILSIKRLWREELDTLHSWGK	_ADILSIK(ac)R_	7	-0.57715	117.04	20619000	20592000
>WP_013237846.1 PHP domain-containing protein	K	FIDSLGHIDYIARYAKFEDKELYYSDFPDII	_YAK(ac)FEDK_	3	0.39065	77.282	27285000	6681800
>WP_013237848.1 glycyl radical protein	K	DMKGLTPETILKVIEKLLSEEKKTSLEGLEP	_VIEK(ac)LLSEEK_	4	-0.05658	49.5	7723400	5435300
>WP_013237851.1 MULTISPECIES: sensor histidine kinase	K	IDLNWISKNYELPLEKLKEAAERIEIVESDY	_NYELPLEK(ac)LK_	8	1.2168	57.348	3649300	3847000
>WP_013237875.1 MULTISPECIES: Rrf2 family transcriptional regulator	K	YLEQLFSNLRKSNLVKSIRGAQGGYILNREP	_SNLVK(ac)SIR_	5	-0.30912	115.78	18227000	32691000
>WP_013237876.1 MULTISPECIES: cysteine desulfurase NifS	K	IITTKIEHHAILHACKFLEKNGFEVTYLPVD	_IEHHAILHACK(ac)FLEK_	11	-0.25411	99.481	7610400	32129000
>WP_013237876.1 MULTISPECIES: cysteine desulfurase NifS	K	GKAIELATSEMKTESKRLSKLRDKLTKGLVE	_AIELATSEMK(ac)TESK(ac)R_	14	0.58622	108.71	12695000	15440000
>WP_013237876.1 MULTISPECIES: cysteine desulfurase NifS	K	VEEIPYAKVNGPSGDKRLPGNVNLSFIGIEG	_VNGPSGDK(ac)R_	8	0.21632	63.426	23371000	13597000
>WP_013237876.1 MULTISPECIES: cysteine desulfurase NifS	K	VARRRAMSPLWEDFLKTQNKGEKVEQL	_RAMSPLWEDFLK(ac)TQNK_	12	-1.5216	83.633	91710000	75191000
>WP_013237876.1 MULTISPECIES: cysteine desulfurase NifS	K	IVGIGKAIELATSEMKTESKRLSKLRDKLTK	_AIELATSEMK(ac)TESK(ac)R_	10	0.58622	164.81	352270000	252020000
>WP_013237880.1 MULTISPECIES: alanine--tRNA ligase	K	EKEIVHKLDLQSSELKELEKEIEELKLKVAG	_LDLQSSELK(ac)ELEK_	9	0.35133	39.509	9371800	0
>WP_013237880.1 MULTISPECIES: alanine--tRNA ligase	K	EISSKLKCSEKEIVHKLDLQSSELKELEKEI	_EIVHK(ac)LDLQSSELK_	5	0.33774	40.025	11114000	7753200

>WP_013237880.1 MULTISPECIES: alanine--tRNA ligase	K	YPELKEKEEYIKKIIKLEEERFDETIDSGMQ	_IIK(ac)LEEER_	3	0.53241	100.02	68657000	3953000
>WP_013237881.1 MULTISPECIES: IreB family regulatory phosphoprotein	K	ALTKEILTEVYDSLIKKGYNPVNQLVGYLIS	_EILTEVYDSLIK(ac)K_	12	0.60913	79.633	39704000	18774000
>WP_013237883.1 MULTISPECIES: DUF1292 domain-containing protein	K	NAADLILEDENGKKVKFQVVTKFDIKEEEYI	_VK(ac)FQVVTK_	2	0.12771	116.05	35051000	20779000
>WP_013237885.1 MULTISPECIES: ribonuclease J	K	VPVYGTKLTLGIVESKLREHNLNDVVKLKCV	_LTLGIVESK(ac)LR_	9	-0.06321	122.51	0	22934000
>WP_013237885.1 MULTISPECIES: ribonuclease J	K	ERTVGTTFQKFFSLAKSRIIVATFASNIHRI	_FFSLAK(ac)SR_	6	-0.23358	95.099	6395100	7909800
>WP_013237885.1 MULTISPECIES: ribonuclease J	K	GHACQEELKLIHTLVKPKFFMPVHGEYRHLK	_LIHTLVK(ac)PKFFMPVHGEYR_	7	-0.24494	40.349	17342000	8106400
>WP_013237885.1 MULTISPECIES: ribonuclease J	K	ACQEELKLIHTLVKPKFFMPVHGEYRHLKQH	_LIHTLVKPK(ac)FFMPVHGEYR_	9	0.79547	93.754	17342000	8529900
>WP_013237885.1 MULTISPECIES: ribonuclease J	K	GEPMSALSRMAASEHKKVNIIPGDTIIISAN	_MAASEHK(ac)K_	7	-0.17696	106.36	41700000	30592000
>WP_013237885.1 MULTISPECIES: ribonuclease J	K	IVESKLREHNLNDVVKLKCVKAKDVIKLDSM	_LREHNLNDVVK(ac)LK_	11	0.27248	208.72	70113000	68824000
>WP_013237886.1 MULTISPECIES: YlbF family regulator	K	CEEVTALREASKEIEKSEANIKMLDDFRKIQ	_EIEK(ac)SEANIK_	4	-0.62584	56.548	3802700	19367000
>WP_013237886.1 MULTISPECIES: YlbF family regulator	K	LREASKEIEKSEANIKMLDDFRKIQMEAYSE	_SEANIK(ac)MLDDFR_	6	-1.1224	91.914	10122000	6755200
>WP_013237887.1 MULTISPECIES: translational GTPase TypA	K	AGECSRAEDIEVNVCKKKHLTNTRTSSADDA	_AEDIEVNVCK(ac)K_	10	4.7724	49.5	4385600	10727000
>WP_013237887.1 MULTISPECIES: translational GTPase TypA	K	KKALELKLKPIVVINKIDKSDARPSEVLDEI	_LKPIVVINK(ac)IDK_	9	0.16266	44.829	16471000	12198000
>WP_013237889.1 MULTISPECIES: peptidase U32	K	IDHIPELVKSGINSLKIEGRMKSSYYVATVV	_SGINSLK(ac)IEGR_	7	-0.31892	63.727	13284000	12175000
>WP_013237895.1 MULTISPECIES: cell division protein FtsZ	K	TLVTIPNERLLSVVDKKTTLMESFRLADDVL	_LLSVVDK(ac)K_	7	-0.72657	62.582	4679500	6079600
>WP_013237895.1 MULTISPECIES: cell division protein FtsZ	K	KLTKGLGAGANPEIGKKAAEENKDEISQAIK	_GLGAGANPEIGK(ac)K_	12	-0.52887	62.14	6113800	6681200
>WP_013237895.1 MULTISPECIES: cell division protein FtsZ	K	PFEGRKRMLHAEMGIKDLKDKVDTLVTIPNE	_MLHAEMGIK(ac)DLK_	9	0.16257	123.84	16658000	10821000
>WP_013237901.1 peptidoglycan editing factor PgeF	K	GWKGTLACILSKTIEKLERNFNSESKDIIVC	_TIEK(ac)LER_	4	-0.07609	89.142	21984000	18317000
>WP_013237909.1 MULTISPECIES: DUF47 domain-containing protein	K	EENLKKLKEVEHEGDKKQHEILEQLNKTFIT	_EVEHEGDK(ac)K_	8	0.20417	131.62	79126000	44061000
>WP_013237912.1 MULTISPECIES: ribosome biogenesis GTPase Der	K	ISKAVMMKEPPVILGKRLKIYYVTQIGNKPP	_EPPVILGK(ac)R_	8	-0.06952	53.775	3551100	12050000
>WP_013237912.1 MULTISPECIES: ribosome biogenesis GTPase Der	K	AFIGKPNVGKSSLINKLLGEERVIVSDIPGT	_SSLINK(ac)LLGEER_	6	-0.24166	51.875	5377000	5505700
>WP_013237912.1 MULTISPECIES: ribosome biogenesis GTPase Der	K	ALGLGDMLDKVVENFKDDEDDDIDSEYIKVA	_VVENFK(ac)DDEDDDIDSEYIK_	6	0.72602	70.335	22143000	16446000
>WP_013237913.1 MULTISPECIES: NAD(P)H-dependent glycerol-3-phosphate dehydrogenase	K	NMEYAKKIQDLFMTNKFRIYTNEDIIGVEIG	_IQDLFMTNK(ac)FR_	9	-1.7412	66.262	14669000	9212600
>WP_013237915.1 MULTISPECIES: YicC family protein	K	VEIKSVNHRYCDLNIKMPKSLMPLEDRIRKI	_YCDLNIK(ac)MPK_	7	1.3697	42.743	0	4810300
>WP_013237915.1 MULTISPECIES: YicC family protein	K	SLMPLEDRIRKIVQQKINRGKVDVFITQMNF	_IVQQK(ac)INR_	5	-0.16641	60.682	6437500	6023300
>WP_013237915.1 MULTISPECIES: YicC family protein	K	RLNSHIVQFKETLTLKEPVGRKLDFILQEMN	_ETLTLK(ac)EPVGR_	6	0.59342	59.198	10768000	9484100
>WP_013237916.1 MULTISPECIES: DUF370 domain-containing protein	K	ANRLVAIVSPESAPIKRIIQEARDRGMLIDA	_LVAIVSPESAPIK(ac)R_	13	0.45788	147.87	50532000	36638000
>WP_013237919.1 MULTISPECIES: bifunctional phosphopantothenoylcysteine decarboxylase/phosphopantothenate--cysteine ligase CoaBC	K	FEEPRAFEIQHISLAKKADLVAIVPATANII	_AFEIQHISLAK(ac)K_	11	0.74191	64.121	6889600	5761600
>WP_013237926.1 MULTISPECIES: serine/threonine-protein phosphatase	K	YMKSNETSNMEEDLLKAVSVANDKIYELSIS	_SNETSNMEEDLLK(ac)AVSVANDK_	13	0.3408	35.389	4329000	6954300

>WP_013237927.1 MULTISPECIES: serine/threonine-protein kinase	K	PIRRYQTMEDMLVDLKKIENNQKINLSDDDL	_YQTMEDMLVDLK(ac)K_	12	-0.36994	97.551	62015000	42275000
>WP_013237934.1 DAK2 domain-containing protein	K	PGWILSEAIKLGELSKIKVDNMREQHRHLLS	_LGELSK(ac)IK_	6	1.0348	79.906	2659600	5794000
>WP_013237934.1 DAK2 domain-containing protein	K	TEFFIHSKGTCINEFKKKLETIGDSMVVVDD	_GTCINEFK(ac)K_	8	0.16535	61.65	6085700	4813300
>WP_013237934.1 DAK2 domain-containing protein	K	VTYAVRDTEIDDISIKKGDILGLIDGKIEQV	_DTEIDDISIK(ac)K_	10	-0.07982	82.171	9695600	10357000
>WP_013237934.1 DAK2 domain-containing protein	K	DCEEEKVKHFVEEIEKKYTEMDVQCYSGKQP	_HFVEEIEK(ac)K_	8	-0.08796	125.48	13711000	10818000
>WP_013237934.1 DAK2 domain-containing protein	K	LEVSNSKDSSIESISKQLARGALMGARGNSG	_DSSIESISK(ac)QLAR_	9	0.24274	74.987	14164000	10038000
>WP_013237934.1 DAK2 domain-containing protein	K	ELLQDVCSYSRTILNKTPDMLPVLKKAKVVD	_TILNK(ac)TPDMLPVLK_	5	0.65188	71.279	22985000	17903000
>WP_013237936.1 MULTISPECIES: 16S rRNA (guanine(966)-N(2))-methyltransferase RsmD	K	FLDKNVENLGFSNICKTINKDSYEALQELSR	_NVENLGFSNICK(ac)TINK_	12	2.0863	35.757	2463700	14707000
>WP_013237938.1 hypothetical protein	K	TKEANMRAEEIISSAKKNAKAIRLGARDYAD	_AEEIISSAK(ac)K_	9	0.69215	50.284	1897300	0
>WP_013237938.1 hypothetical protein	K	LDVLQKIVDYLPSELKKAQWIVNEKERILSE	_IVDYLPSELK(ac)K_	10	0.75336	89.548	7736600	4512200
>WP_013237940.1 MULTISPECIES: nucleotidyltransferase	K	CMVPYLRYKSFLYENKIKNLPDISEGIENRI	_SFLYENK(ac)IK_	7	0.36484	55.567	1568300	1906800
>WP_013237942.1 MULTISPECIES: phosphate acetyltransferase	K	VLAEGEEERTLQACEKIIKEGIANLILVGNE	_TLQACEK(ac)IIK_	7	-0.58514	98.943	14956000	17673000
>WP_013237942.1 MULTISPECIES: phosphate acetyltransferase	K	KHELVDKVRNAVEIAKKAKPDLSLDGELQLD	_NAVEIAK(ac)K_	7	0.7243	86.011	20549000	0
>WP_013237942.1 MULTISPECIES: phosphate acetyltransferase	K	IIKEGIANLILVGNEKVIKEKASKLGVSLNG	_EGIANLILVGNEK(ac)VIK_	13	0.2327	145.55	32658000	19985000
>WP_013237942.1 MULTISPECIES: phosphate acetyltransferase	K	LILVGNEKVIKEKASKLGVSLNGAEIVDPET	_ASK(ac)LGVSLNGAEIVDPETSDKLK_	3	-0.55397	91.128	89643000	74457000
>WP_013237942.1 MULTISPECIES: phosphate acetyltransferase	K	ELQLDASIVEKVASLKAPGSEVAGKANVLVF	_VASLK(ac)APGSEVAGK_	5	0.09402	111.12	92640000	81592000
>WP_013237942.1 MULTISPECIES: phosphate acetyltransferase	K	DAFYELRKKKGITPEKADKIVRDPIYFATMM	_KGITPEK(ac)ADK_	7	-0.03979	161.99	655920000	363410000
>WP_013237943.1 acetate kinase	K	LKYQLIDMQDESVVAKGLVERIGMDGSILTH	_YQLIDMQDESVVAK(ac)GLVER_	14	-2.9531	60.564	25327000	17542000
>WP_013237943.1 acetate kinase	K	ICNGLSYFGIKIDEEKNKKRGEALEISTPDS	_IDEEK(ac)NKK_	5	0.2295	159.04	30448000	27543000
>WP_013237943.1 acetate kinase	K	NKKRGEALEISTPDSKIKVLVIPTNEELMIA	_RGEALEISTPDSK(ac)IK_	13	0.18434	106.19	38896000	42134000
>WP_013237943.1 acetate kinase	K	KRTGMSVDEVDTLMNKKSGILGVSGVSSDFR	_TGMSVDEVDTLMNK(ac)K_	14	-0.19443	108.71	56904000	50266000
>WP_013237943.1 acetate kinase	K	IGMDGSILTHKVNGEKFVTEQTMEDHKVAIQ	_VNGEK(ac)FVTEQTMEDHK_	5	-0.54028	185.67	1.34E+09	1.099E+09
>WP_013237947.1 MULTISPECIES: acyl carrier protein	K	EAEKVKTVGDVVEYIKAHTEE	_TVGDVVEYIK(ac)AHTEE_	10	0.14273	41.644	4127500	0
>WP_013237953.1 MULTISPECIES: signal recognition particle protein	K	MAFEGLASKLQETLKKLRGKGKLSEKDIKD	_LQETLK(ac)K_	6	0.28624	91.041	13092000	16023000
>WP_013237953.1 MULTISPECIES: signal recognition particle protein	K	QETLKKLRGKGKLSEKDIKDAMREVKLALLE	_LSEK(ac)DIK_	4	0.14505	85.212	15144000	12756000
>WP_013237954.1 MULTISPECIES: 30S ribosomal protein S16	K	AVKWIKDGAQPTDVIKKLFDKAGLKDKLDK	_DGAQPTDVIK(ac)K_	10	0.1223	62.408	45918000	28803000
>WP_013237954.1 MULTISPECIES: 30S ribosomal protein S16	K	EEIGYYNPTTDPTTVKFDEEKAVKWIKDGAQ	_FIEEIGYYNPTTDPTTVK(ac)FDEEK_	18	-0.13446	76.186	117290000	78371000
>WP_013237958.1 MULTISPECIES: 50S ribosomal protein L19	K	QIRTDLPKFNVGDTVKVHVKIKEGNRERIQV	_FNVGDTVK(ac)VHVK_	8	0.48192	47.712	7135600	4027800
>WP_013237958.1 MULTISPECIES: 50S ribosomal protein L19	K	VGVERTFPVNAPIIDKIDVIRRGKVRRAKLY	_TFPVNAPIIDK(ac)IDVIR_	11	0.29663	113.69	52041000	20496000
>WP_013237958.1 MULTISPECIES: 50S ribosomal protein L19	K	IIKQIENEQIRTDLPKFNVGDTVKVHVKIKE	_TDLPK(ac)FNVGDTVK_	5	-0.22586	135.02	2.164E+09	1.698E+09

>WP_013237966.1 MULTISPECIES: GTP-sensing pleiotropic transcriptional regulator CodY	K	LDGNEGLLVASKIADKVGITRSVIVNALRKF	_IADK(ac)VGITR_	4	0.51667	61.161	14051000	11776000
>WP_013237966.1 MULTISPECIES: GTP-sensing pleiotropic transcriptional regulator CodY	K	GTHIKILNEKLMDELKKIK	_LMDELK(ac)K_	6	-0.01446	78.496	16956000	14158000
>WP_013237966.1 MULTISPECIES: GTP-sensing pleiotropic transcriptional regulator CodY	K	RSLGMKGTHIKILNEKLMDELKKIK	_ILNEK(ac)LMDELK_	5	-0.39683	90.614	33915000	31362000
>WP_013237967.1 30S ribosomal protein S2	K	FEVLPKKEVIKLNNEKEKLEKNLGGIKTMNA	_LNNEK(ac)EK_	5	-0.45515	80.229	12654000	7739200
>WP_013237967.1 30S ribosomal protein S2	K	YIFTERNGIYIIDLQKTVKKVEEAYNFIREI	_NGIYIIDLQK(ac)TVK_	10	0.63467	88.087	15585000	13322000
>WP_013237967.1 30S ribosomal protein S2	K	IGTKKQAQEAIKEEAKRSNMHYVNNRWLGGM	_QAQEAIK(ac)EEAK(ac)R_	11	0.34693	247.13	104300000	59034000
>WP_013237967.1 30S ribosomal protein S2	K	GGMLTNFATIKTRIAKLEELEKMEEDGTFEV	_IAK(ac)LEELEK_	3	0.02865	136.63	841410000	603170000
>WP_013237968.1 elongation factor Ts	K	LVNQVWVKDSDFTIDKYLKDKSKEVGAEIKI	_DSDFTIDK(ac)YLK_	8	0.49195	45.879	3910700	0
>WP_013237968.1 elongation factor Ts	K	MNCKKALNEANGDTEKAIEILREKGLSAAAK	_ALNEANGDTEK(ac)AIEILR_	11	-2.8006	91.457	5184500	8678000
>WP_013237968.1 elongation factor Ts	K	EVGAEIKISDFVRFEKGEGIEKKEENFAEEV	_FEK(ac)GEGIEK_	3	-0.25218	50.978	8020100	6549400
>WP_013237968.1 elongation factor Ts	K	VKELRERTGAGMMNCKKALNEANGDTEKAIE	_TGAGMMNCK(ac)K_	9	-0.30239	53.683	9289900	5583600
>WP_013237968.1 elongation factor Ts	K	PLFLDKNTVDTDALEKEKEIYKVQALNEGKP	_NTVDTDALEK(ac)EK_	10	0.61656	110.29	12776000	9118400
>WP_013237970.1 MULTISPECIES: ribosome recycling factor	K	MGKTIDALKSELASMKAGRASAALLDRIEAE	_SELASMK(ac)AGR_	7	-0.27105	60.434	6562400	0
>WP_013237970.1 MULTISPECIES: ribosome recycling factor	K	LCIQPWDKTALKAIEKAILKSDLGINPSNDG	_TALK(ac)AIEK(ac)AILK_	8	-0.10219	89.355	16474000	10993000
>WP_013237970.1 MULTISPECIES: ribosome recycling factor	K	QKKTDDFIKKIDEIIKKKENEIMSL	_KIDEIIK(ac)K_	7	0.12683	121.73	29571000	23636000
>WP_013237970.1 MULTISPECIES: ribosome recycling factor	K	IKALKKENDISEDEIKKAEADVQKKTDDFIK	_ENDISEDEIK(ac)K_	10	-0.38196	114.86	37827000	26973000
>WP_013237970.1 MULTISPECIES: ribosome recycling factor	K	DISEDEIKKAEADVQKKTDDFIKKIDEIIKK	_KAEADVQK(ac)K_	8	0.35803	123.96	97096000	75549000
>WP_013237973.1 MULTISPECIES: 1-deoxy-D-xylulose-5-phosphate reductoisomerase	K	HIIDEFNPKYAVLTEKESYLKIKDIFSNKKS	_YAVLTEK(ac)ESYLK_	7	0.1528	72.958	18154000	11297000
>WP_013237976.1 MULTISPECIES: ribosome maturation factor RimP	K	SSPGVERKLYTEEHLKRYIGYSVQVNISGLL	_LYTEEHLK(ac)R_	8	-1.0118	154.49	22915000	21060000
>WP_013237977.1 transcription termination/antitermination protein NusA	K	KYNFNDKLKLYIIEVKNTTKGAQIVISRTHP	_LYIIEVK(ac)NTTK_	7	-0.4895	66.262	3739000	8531600
>WP_013237977.1 transcription termination/antitermination protein NusA	K	VGPKGVRVQNIVNELKNEKIDIIKWSKLPEE	_VQNIVNELK(ac)NEK_	9	0.22337	42.789	4239200	9681800
>WP_013237977.1 transcription termination/antitermination protein NusA	K	GPNEQIPGEKYNFNDKLKLYIIEVKNTTKGA	_YNFNDK(ac)LK_	6	-0.14224	69.954	4645600	4290700
>WP_013237977.1 transcription termination/antitermination protein NusA	K	SLAIGKEGQNVRLAAKLTGWKIDIKSKSQVE	_LAAK(ac)LTGWK_	4	0.65899	101.11	15141000	12924000
>WP_013237977.1 transcription termination/antitermination protein NusA	K	KGAQIVISRTHPGLIKRLFELEVPEIFDGTV	_THPGLIK(ac)R_	7	0.1477	90.777	36027000	27670000
>WP_013237980.1 translation initiation factor IF-2	K	INQEIDFNTAEKLGEKFDVTVLRKEEDINKE	_LGEK(ac)FDVTVLR_	4	-0.38634	88.338	2244700	4618200
>WP_013237980.1 translation initiation factor IF-2	K	NIIVKADVQGSIEALKQSLQKLSNEEVKVRV	_ADVQGSIEALK(ac)QSLQK_	11	1.5089	34.909	2376000	9426500
>WP_013237980.1 translation initiation factor IF-2	K	EALKQSLQKLSNEEVKVRVLHGAVGAITETD	_LSNEEVK(ac)VR_	7	-1.1476	73.985	4167000	0
>WP_013237980.1 translation initiation factor IF-2	K	QSAHKVSLEDLYNQIKEGKVKELNIIVKADV	_VSLEDLYNQIK(ac)EGK_	11	0.79789	39.005	11576000	10979000
>WP_013237981.1 MULTISPECIES: 30S ribosome-binding factor RbfA	K	MTNYRSGRINEEMKKEISNIIRSDIKDPR	_INEEMK(ac)K_	6	-1.0498	91.041	9339100	0

>WP_013237984.1 MULTISPECIES: bifunctional riboflavin kinase/FAD synthetase	K	SSSIIRTIISDEGDIKKANKLLTRPFMLQGE	_TIISDEGDIK(ac)K_	10	0.68432	39.917	1375700	6888900
>WP_013237985.1 MULTISPECIES: 30S ribosomal protein S15	K	IALLTERINHLNDHLKIHKKDHHSRRGLLMM	_INHLNDHLK(ac)IHK_	9	-0.01107	69.276	11347000	11002000
>WP_013237985.1 MULTISPECIES: 30S ribosomal protein S15	K	MEKALKEEIMKKYARHEGDTGSPEVQ	_ALKEEIMK(ac)K_	8	-0.28392	147.95	117260000	64693000
>WP_013237991.1 30S ribosomal protein S12 methylthiotransferase RimO	K	GELMVLQKQVMTSINKSKIGSTYKVIVEGKK	_QVMTSINK(ac)SK_	8	0.87443	47.288	4385800	0
>WP_013237993.1 MULTISPECIES: recombinase RecA	K	FYSSVRMDVRRIDSIKQGDTIVGNRTRVKVI	_RIDSIK(ac)QGDTIVGNR_	6	0.55814	69.716	7613900	11900000
>WP_013237993.1 MULTISPECIES: recombinase RecA	K	RLMSQALRKLTSSINKSKCVTVFINQLREKV	_LTSSINK(ac)SK_	7	-0.03114	99.815	13160000	12542000
>WP_013237993.1 MULTISPECIES: recombinase RecA	K	QFLKENNDILVEIESKIREKYKLPSLKKNSL	_ENNDILVEIESK(ac)IR_	12	-0.07177	124.45	37407000	23848000
>WP_013237994.1 MULTISPECIES: ribonuclease Y	K	LSSEEAKNILLEQVNKEIKHESAMMIKEIET	_NILLEQVNK(ac)EIK_	9	0.41598	83.314	11224000	0
>WP_013237994.1 MULTISPECIES: ribonuclease Y	K	IDGRIHPARIEEMVQKAEKEVENNVKEEGEQ	_IEEMVQK(ac)AEK_	7	0.61749	96.015	23169000	0
>WP_013237994.1 MULTISPECIES: ribonuclease Y	K	TQKKEAILEAKEEIHKLRNDFDKESRDRRNE	_EEIHK(ac)LR_	5	-0.17856	126.24	29392000	29182000
>WP_013237994.1 MULTISPECIES: ribonuclease Y	K	ISVEELYKKQREKLEKLSGLSSEEAKNILLE	_LEK(ac)LSGLSSEEAK_	3	-0.21247	163.8	40842000	24961000
>WP_013237999.1 pyridoxal phosphate-dependent aminotransferase	K	TKEEDGFKYTLETLEKAYTKNTKMILLNSPN	_YTLETLEK(ac)AYTK_	8	-0.25812	59.185	10335000	11064000
>WP_013237999.1 pyridoxal phosphate-dependent aminotransferase	K	QYAAVAALNGKDTQIKSMVSEFKDRRDCMVS	_DTQIK(ac)SMVSEFK_	5	0.81578	61.375	28999000	27265000
>WP_013237999.1 pyridoxal phosphate-dependent aminotransferase	K	LNGKDTQIKSMVSEFKDRRDCMVSKIDKMKN	_SM(ox)VSEFK(ac)DR_	7	-0.0386	141.88	77101000	45527000
>WP_013237999.1 pyridoxal phosphate-dependent aminotransferase	K	ATSMENIQNGMDRIDKFISQLK	_IDK(ac)FISQLK_	3	0.35517	112.36	737510000	723610000
>WP_013238000.1 MULTISPECIES: HPr family phosphocarrier protein	K	MISKEVIVKSSTGLHARPATLLVK	_EVIVK(ac)SSTGLHAR_	5	0.65138	51.875	6207200	3985400
>WP_013238000.1 MULTISPECIES: HPr family phosphocarrier protein	K	KASSFKSDLSIEFDGKKANIKSLIGVLSLGV	_SDLSIEFDGK(ac)K_	10	0.37769	137.17	20965000	25364000
>WP_013238000.1 MULTISPECIES: HPr family phosphocarrier protein	K	KSSTGLHARPATLLVKKASSFKSDLSIEFDG	_PATLLVK(ac)K_	7	0.20889	145.34	34314000	14018000
>WP_013238002.1 MULTISPECIES: adenylosuccinate lyase	K	QVVDFIQQQIKPLLDKSKDILGEEAEIDV	_APGQVVDFIQQQIKPLLDK(ac)SK_	19	-0.94136	226.51	0	27073000
>WP_013238002.1 MULTISPECIES: adenylosuccinate lyase	K	FFKMSEEEILSLVDSKKFIGRAPGQVVDFIQ	_MSEEEILSLVDSK(ac)K_	13	0.13136	47.532	9020500	12810000
>WP_013238002.1 MULTISPECIES: adenylosuccinate lyase	K	ELKANMENINYEDAKKREKEVRHDVMSHVYA	_ANMENINYEDAKK(ac)R_	13	0.27727	59.185	20688000	14389000
>WP_013238002.1 MULTISPECIES: adenylosuccinate lyase	K	DNTDLIIMREALHIIKRKVINVISYLTKFAL	_EALHIIK(ac)R_	7	0.05767	154.31	65903000	56451000
>WP_013238002.1 MULTISPECIES: adenylosuccinate lyase	K	DELKANMENINYEDAKKREKEVRHDVMSHVY	_ANM(ox)ENINYEDAK(ac)K_	12	0.03057	194.7	86151000	89384000
>WP_013238004.1 glutamate--tRNA ligase	K	LSKRNGDASFEDLIEKGYLKEAVLNYIALLG	_NGDASFEDLIEK(ac)GYLK_	12	1.5616	60.474	4783000	0
>WP_013238004.1 glutamate--tRNA ligase	K	ITHVVRGSEYLSSAPKYNRLYEAFGWEIPIY	_GSEYLSSAPK(ac)YNR_	10	-0.06919	95.417	16985000	14863000
>WP_013238005.1 MULTISPECIES: type I glyceraldehyde-3-phosphate dehydrogenase	K	RAAGESIIPTTTGAAKAVAKVLPNLEGKLSG	_AAGESIIPTTTGAAK(ac)AVAK_	15	0.11078	48.314	8848200	8854800
>WP_013238005.1 MULTISPECIES: type I glyceraldehyde-3-phosphate dehydrogenase	K	SCTTNCLAPFAKVLDKNFGIIRGLMTTVHSY	_VLDK(ac)NFGIIR_	4	-1.0071	101.65	30991000	10185000
>WP_013238005.1 MULTISPECIES: type I glyceraldehyde-3-phosphate dehydrogenase	K	DLANYIAGEMEKASEKPIDMVAEGV	_ASEK(ac)PIDMVAEGV_	4	0.34298	57.802	61976000	44816000
>WP_013238005.1 MULTISPECIES: type I glyceraldehyde-3-phosphate dehydrogenase	K	PWGKLDVDIVIESTGKFKDRESLYKHIKAGA	_LDVDIVIESTGK(ac)FK_	12	-0.13579	173.25	65666000	87902000

>WP_013238005.1 MULTISPECIES: type I glyceraldehyde-3-phosphate dehydrogenase	K	ELSKSVTVEEVNEAFKRASEGSMKGILGYSE	_SVTVEEVNEAFK(ac)R_	12	-2.5108	218.01	121480000	89130000
>WP_013238010.1 MULTISPECIES: response regulator	K	DDSLSVRQFIRKILEKANYKVYEAGDGEEGI	_ILEK(ac)ANYK_	4	-0.23802	92.47	37769000	25426000
>WP_013238011.1 MULTISPECIES: methyl-accepting chemotaxis protein	K	YLAVDQTHKIFKVILKSIESMIAKVDEVKTS	_VILK(ac)SIESMIAK_	4	-0.31205	36.284	2682000	1539500
>WP_013238011.1 MULTISPECIES: methyl-accepting chemotaxis protein	K	KDLAESFNLMMKNISKLMNNVLKSSETVLET	_NISK(ac)LMNNVLK_	4	-0.41566	49.5	6395300	4303000
>WP_013238039.1 MULTISPECIES: ArsC family transcriptional regulator	K	VGIDNLINNRVKEYDKLNIGLIRSGDIKEEI	_EYDK(ac)LNIGLIR_	4	1.8081	40.589	1544700	0
>WP_013238044.1 MULTISPECIES: ABC transporter ATP-binding protein	K	NFKDFKAVKGIDLSIKKGRVYGFLGPNGAGK	_GIDLSIK(ac)K_	7	0.99385	119.17	80556000	2177000
>WP_013238048.1 methyl-accepting chemotaxis protein	K	QKVNISSDVVTELSEKSKTIANVTTTITDIA	_VNISSDVVTELSEK(ac)SK_	14	-0.51124	94.544	14065000	13936000
>WP_013238048.1 methyl-accepting chemotaxis protein	K	TTFNKMINNLKNIIIKIRNVSEVVAESSNSY	_NIIIK(ac)IR_	5	0.19741	114.02	34904000	32570000
>WP_013238048.1 methyl-accepting chemotaxis protein	K	SDGSKNLHTTVELLNKMEQKVNISSDVVTEL	_NLHTTVELLNK(ac)MEQK_	11	0.25088	209.6	209580000	205260000
>WP_013238048.1 methyl-accepting chemotaxis protein	K	YKEVSLEVVQEGKNIKQSIDNLTSGSKNIAE	_NIK(ac)QSIDNLTSGSK_	3	-1.0316	166.38	300140000	308560000
>WP_013238050.1 MULTISPECIES: pyridoxamine 5'-phosphate oxidase	K	KANPKMQISATSPEGKWLRLNGKAVFNTSKE	_MQISATSPEGK(ac)WLR_	11	-0.26454	56.482	4392000	4179900
>WP_013238050.1 MULTISPECIES: pyridoxamine 5'-phosphate oxidase	K	TANTKNVYKQLKANPKMQISATSPEGKWLRL	_ANPK(ac)MQISATSPEGK_	4	0.01459	177.88	18541000	383020000
>WP_013238051.1 MULTISPECIES: methyl-accepting chemotaxis protein	K	MDMCSKKVFALMDEIKNIKLN	_VFALMDEIK(ac)NIK_	9	-1.4295	66.262	4071600	6866500
>WP_013238051.1 MULTISPECIES: methyl-accepting chemotaxis protein	K	KGCRLGKWYYNIDSDKFKNSKAFVELEKSHI	_WYYNIDSDK(ac)FK_	9	0.71311	38.021	6735200	2643400
>WP_013238051.1 MULTISPECIES: methyl-accepting chemotaxis protein	K	DSNCKGIGKFIYDLSKRIDSMRIDMLKNKSC	_FIYDLSK(ac)R_	7	0.70208	148.38	9610600	6940100
>WP_013238054.1 electron transfer flavoprotein subunit alpha/FixB family protein	K	AGRGIKKAEDLHMLHKLASLLGGEIGASRAL	_KAEDLHMLHK(ac)LASLLGGEIGASR_	10	-0.88619	39.647	2654600	10085000
>WP_013238063.1 MULTISPECIES: ferrous iron transport protein B	K	KKNTVVKETVSDKIDKVITNKWLGIPIFALI	_IDK(ac)VITNK_	3	0.64684	63.565	6988200	5677900
>WP_013238097.1 MULTISPECIES: (deoxy)nucleoside triphosphate pyrophosphohydrolase	K	NLSSLKWAPADIPTAKKLISERV	_WAPADIPTAK(ac)K_	10	1.1784	36.847	5215800	0
>WP_013238116.1 DNA phosphorothioation-dependent restriction protein DptH	K	RKSRPYQKVIEEYLTKKGIENAKEHSSSIIN	_VIEEYLTK(ac)K_	8	1.0432	53.683	8137200	8608500
>WP_013238116.1 DNA phosphorothioation-dependent restriction protein DptH	K	LKRENKSDFRLIGDNKLQHGTKEFFTRDEFR	_LIGDNK(ac)LQHGTK_	6	-0.55252	38.021	66154000	1693000
>WP_013238118.1 hypothetical protein	K	DNFKNYIRYIEHYEEKRKYLPNKLNNILQRT	_YIEHYEEK(ac)R_	8	-0.36208	55.567	3068500	3638100
>WP_013238119.1 cysteine desulfurase	K	EIAKKDMNKYIKKISKLEIETKKMLTEKYPG	_ISK(ac)LEIETK_	3	0.04077	77.379	0	3132100
>WP_013238121.1 DNA sulfur modification protein DndD	K	KSLILDEEHLDNLLIKRNQVEDNFKNSGGLL	_SLILDEEHLDNLLIK(ac)R_	15	0.72979	68.944	6696000	9692700
>WP_013238170.1 threonine ammonia-lyase	K	_MKVNLTLKDVLEASKRIEGICVKTKLIYSS	_DVLEASK(ac)R_	7	-0.26615	80.239	0	9726700
>WP_013238170.1 threonine ammonia-lyase	K	ITGAFKLRGASNKIVKLSDEQKAKGLIASSA	_IVK(ac)LSDEQK_	3	-0.96993	101.9	0	29810000
>WP_013238170.1 threonine ammonia-lyase	K	IKVQATKNYGADVVLKGKVYDEAYEEAKRLE	_NYGADVVLK(ac)GK_	9	0.44108	72.652	12769000	6365900
>WP_013238170.1 threonine ammonia-lyase	K	AEKGANVIKLDHNQFKAVDRLKHVVLEVTVE	_LDHNQFK(ac)AVDR_	7	0.21601	126.76	21783000	25839000
>WP_013238170.1 threonine ammonia-lyase	K	DTPGQLLKISQILAEKGANVIKLDHNQFKAV	_ISQILAEK(ac)GANVIK_	8	0.40366	57.136	27989000	11820000
>WP_013238170.1 threonine ammonia-lyase	K	VVLKGKVYDEAYEEAKRLEKEHGYTFVHPFN	_VYDEAYEEAK(ac)R_	10	-0.44731	133.89	32689000	28703000

>WP_013238170.1 threonine ammonia-lyase	K	LKISQILAEKGANVIKLDHNQFKAVDRLKHV	_GANVIK(ac)LDHNQFK_	6	-0.13709	210.15	56828000	58824000
>WP_013238173.1 xanthine phosphoribosyltransferase	K	KKILEEGHALSESILKVDSFLNHKVDPSLMY	_ILEEGHALSESILK(ac)VDSFLNHK_	14	-0.17636	192.16	76561000	12274000
>WP_013238198.1 MULTISPECIES: nitroreductase	K	IGSCWINQLRTICDEKEVREVLSSFGIPENH	_TICDEK(ac)EVR_	6	-0.0598	51.445	6945900	4214100
>WP_013238198.1 MULTISPECIES: nitroreductase	K	QFTVVQSREKMELLAKVVRKALGRDAGYNFY	_MELLAK(ac)VVR_	6	0.40341	65.305	12114000	7727900
>WP_013238224.1 hypothetical protein	K	VNKAMALSKELHDIFKDKHKCLCCRVLTKDM	_ELHDIFK(ac)DK_	7	0.3559	109.66	9287900	14694000
>WP_013238229.1 VanZ family protein	K	SSGTKISVWGKYDKGKFIANVISILDESREQ	_GK(ac)FIANVISILDESR_	2	0.26119	44.457	31760000	20293000
>WP_013238237.1 aminopeptidase	K	IECAEFTRLISEVAYKEGARDVIINWSDELS	_LISEVAYK(ac)EGAR_	8	-0.38356	62.924	7961600	6014300
>WP_013238237.1 aminopeptidase	K	YLNGKDEIFDEFPNWKKELYTSYAKSGAAFL	_DEIFDEFPNWK(ac)K_	11	-0.26847	66.262	10200000	10065000
>WP_013238312.1 bifunctional acetaldehyde-CoA/alcohol dehydrogenase	K	MKVTNVEELMKRLEEIKDAQKKFATY	_VTNVEELM(ox)K(ac)R_	9	1.1419	150.61	0	2398100
>WP_013238312.1 bifunctional acetaldehyde-CoA/alcohol dehydrogenase	K	PAFKIAQLAGVDVPEKAKVLIGEVESVELEE	_IAQLAGVDVPEK(ac)AK_	12	-0.42894	47.532	0	7535700
>WP_013238312.1 bifunctional acetaldehyde-CoA/alcohol dehydrogenase	K	ILSKDETDKVGKIILKNGALNAGIVGQPAFK	_IILK(ac)NGALNAGIVGQPAFK_	4	-0.12079	32.926	8923000	14600000
>WP_013238312.1 bifunctional acetaldehyde-CoA/alcohol dehydrogenase	K	KVFTDVEPDPTLATAKKGAAELLSYEPDTII	_VFTDVEPDPTLATAK(ac)K_	15	0.0322	86.641	13303000	17695000
>WP_013238312.1 bifunctional acetaldehyde-CoA/alcohol dehydrogenase	K	PEKVYFKYGSLGVALKELKVMNKKKVFIVTD	_YGSLGVALK(ac)ELK_	9	0.47519	72.958	29266000	27184000
>WP_013238312.1 bifunctional acetaldehyde-CoA/alcohol dehydrogenase	K	FEYLPKAYTEGTTNVKAREKMVHASCIAGMA	_AYTEGTTNVK(ac)AR_	10	-1.0346	109.24	7346700	8949100
>WP_013238319.1 XRE family transcriptional regulator	K	LEAIYKAFKQLDKNIKFTDIINIVDNEEKAK	_NIK(ac)FTDIINIVDNEEK_	3	-0.13447	52.823	3754300	1959000
>WP_013238319.1 XRE family transcriptional regulator	K	MIKIYISDLLGKYKKNQAWLAQETGIR	_IYISDLLGK(ac)YK_	9	-0.00695	57.836	3913200	8618600
>WP_013238319.1 XRE family transcriptional regulator	K	KFTDIINIVDNEEKAKD	_FTDIINIVDNEEK(ac)AKD_	15	-0.05687	37.555	4225900	5323000
>WP_013238319.1 XRE family transcriptional regulator	K	NIKFTDIINIVDNEEKAKD	_FTDIINIVDNEEK(ac)AKD_	13	-0.05687	37.555	4225900	6496200
>WP_013238330.1 hypothetical protein	K	EQSKIVEPTALEGKIKFHALNKEILNKVSAA	_IK(ac)FHALNK_	2	-0.02063	87.696	3902800	4040100
>WP_013238393.1 MULTISPECIES: cell division protein FtsA	K	DTIRSLELTAVKSAEKQVTKNTKGRLYCVGY	_SAEK(ac)QVTK_	4	-0.03216	56.432	3032500	0
>WP_013238394.1 MULTISPECIES: amidohydrolase	K	IIPDEVTISGIIRTMKTEHREYVKKRLCEIT	_TMK(ac)TEHR_	3	0.3129	79.469	3492800	3830600
>WP_013238394.1 MULTISPECIES: amidohydrolase	K	MQRNFLKMAESMKQELIETRRD	_NFLK(ac)MAESMK_	4	0.1915	61.815	10322000	8666400
>WP_013238395.1 MULTISPECIES: RluA family pseudouridine synthase	K	TNNKSSVSNTPEKDSKKISMRVKNVNTCGIY	_SSVSNTPEKDSK(ac)K_	12	-0.14702	33.204	3572500	0
>WP_013238395.1 MULTISPECIES: RluA family pseudouridine synthase	K	YMENKTITEGLPPILKKIKKDIFNKF	_TITEGLPPILK(ac)K_	11	-0.29562	85.288	30754000	20871000
>WP_013238399.1 dipeptidase PepV	K	ATQEVVRIKSTLDEEKPGMPFGEGPAKALEK	_STLDEEK(ac)PGMPFGEGPAK_	7	0.25529	66.965	23328000	11511000
>WP_013238399.1 dipeptidase PepV	K	YPVTKKGEEIMDTLSKKFAECGAKIERVEAD	_KGEEIMDTLSK(ac)K_	11	-0.02211	178.67	38587000	24863000
>WP_013238399.1 dipeptidase PepV	K	ATMAALFGLKAIKDLKMPLSKKVRVIFGTNE	_DLK(ac)MPLSK_	3	-0.10263	94.85	61411000	51254000
>WP_013238415.1 MULTISPECIES: flavodoxin	K	TSGQGKNKYNNIIEQKLKEKGFEVVGNFACK	_YNNIIEQK(ac)LK_	8	-0.63651	42.001	2820000	7787900
>WP_013238426.1 MULTISPECIES: asparagine synthase B	K	DSSLVCAIAQKMNPEKPIKTFAIGMTEDAID	_MNPEK(ac)PIK_	5	0.00656	69.423	10275000	6027100

>WP_013238426.1 MULTISPECIES: asparagine synthase B	K	MCTIMCYTGTDMKHEKFAEALQRTESRGPDM	_HEK(ac)FAEALQR_	3	-0.20055	178.81	139120000	129700000
>WP_013238437.1 MULTISPECIES: aldo/keto reductase	K	TAAFYKNEDGVGTGIKESKVPREKIFLTSKV	_NEDGVGTGIK(ac)ESK_	10	1.988	35.35	11607000	6157700
>WP_013238437.1 MULTISPECIES: aldo/keto reductase	K	DEGYEKTLKAFETSIKKLKTDYLDLYLIHWP	_AFETSIK(ac)K_	7	0.02777	84.476	14102000	8028500
>WP_013238445.1 MULTISPECIES: amino acid permease	K	CGRMLYTLSKNGQAPKFLGKVTKDGVPANAI	_NGQAPK(ac)FLGK_	6	-0.22038	52.725	15377000	15658000
>WP_013238450.1 MULTISPECIES: molybdopterin-binding protein	K	IEQEKMDKAVEVAGDKPLIKIMPFKNKKVGI	_AVEVAGDK(ac)PLIK_	8	-0.5931	36.847	4717900	2880300
>WP_013238468.1 MULTISPECIES: deoxyribose-phosphate aldolase	K	DHTLLKPETTKSQIIKLTEEAKKYDFASVCV	_SQIIK(ac)LTEEAK_	5	0.17304	63.727	0	4106900
>WP_013238470.1 phosphopentomutase	K	MDYIKNAGLDSIAIGKIRDIFDGEGVTKAIR	_NAGLDSIAIGK(ac)IR_	11	-0.16538	70.412	7275900	1499000
>WP_013238470.1 phosphopentomutase	K	DIGATIADNFKVKMPKYGESFLQKLI	_MPK(ac)YGESFLQK_	3	-0.06176	88.338	20662000	13643000
>WP_013238471.1 pyrimidine-nucleoside phosphorylase	K	KVAVMGQTGNLTPADKKLYALRDVTGTVNSI	_VAVMGQTGNLTPADK(ac)K_	15	-0.47235	69.729	17973000	9525500
>WP_013238477.1 MULTISPECIES: hypothetical protein	K	CDYHLDEDYNAFRNHKLNHKVLEKVTGKYGV	_NHK(ac)LNHK_	3	0.26339	114.19	58216000	0
>WP_013238483.1 MULTISPECIES: ROK family transcriptional regulator	K	GFSLPGTVNEKELLLKNVPNLGFKNVSFKKF	_ELLLK(ac)NVPNLGFK_	5	0.19108	73.848	7492500	6450400
>WP_013238488.1 thioredoxin	K	KFNRHEYIEDPGQKPKWMARQHHDYQREGNP	_HEYIEDPGQKPK(ac)WMAR_	12	-0.09289	188.4	61131000	91995000
>WP_013238493.1 MULTISPECIES: hypothetical protein	K	IEIEDERGLSHCINLKKCFSVVVEREGSLGY	_GLSHCINLK(ac)K_	9	-0.97491	73.887	9028300	6646400
>WP_013238501.1 MULTISPECIES: NADPH-dependent butanol dehydrogenase	K	MIKDMNKSMDIPLTLKDYGVDEKEFKDSEDF	_SMDIPLTLK(ac)DYGVDEK_	9	0.12311	35.757	2323600	0
>WP_013238501.1 MULTISPECIES: NADPH-dependent butanol dehydrogenase	K	LPRDIYFGENSLETLKDLDGKKAVIVVGGGS	_DIYFGENSLETLK(ac)DLDGK_	13	1.5558	106.94	31699000	20960000
>WP_013238501.1 MULTISPECIES: NADPH-dependent butanol dehydrogenase	K	LDGKKAVIVVGGGSMKRFGFLDKVVNYLKEA	_AVIVVGGGSM(ox)K(ac)R_	11	0.08144	135.99	48244000	41551000
>WP_013238501.1 MULTISPECIES: NADPH-dependent butanol dehydrogenase	K	IVMVNQYLIKSYNEDKEARNQMHLAQCLAGM	_SYNEDK(ac)EAR_	6	0.28862	74.162	77027000	2328700
>WP_013238501.1 MULTISPECIES: NADPH-dependent butanol dehydrogenase	K	SMDIPLTLKDYGVDEKEFKDSEDFIAHNAVL	_DYGVDEK(ac)EFK_	7	-0.08981	115.78	202650000	149510000
>WP_013238501.1 MULTISPECIES: NADPH-dependent butanol dehydrogenase	K	KKACAPRYAEIARSLKLPGNTDDELVDSLTN	_SLK(ac)LPGNTDDELVDSLTNMIK_	3	-0.72274	166.61	224520000	13716000
>WP_013238501.1 MULTISPECIES: NADPH-dependent butanol dehydrogenase	K	ACTGSNPRSINDTEMKKLLEYIYYGKKVDF	_SINDTEMK(ac)K_	8	-0.61578	114.84	292540000	238400000
>WP_013238504.1 MULTISPECIES: chemotaxis protein	K	RKLSEQSRSTVPKIAKLTDNIKEKVESSSKM	_IAK(ac)LTDNIK_	3	-0.2374	131.5	54105000	52901000
>WP_013238521.1 HAMP domain-containing protein	K	EGIAEAIDSIKTVVNKLNTSANKMTENKDKI	_TVVNK(ac)LNTSANK_	5	0.83456	53.252	2290200	0
>WP_013238524.1 MULTISPECIES: hypothetical protein	K	KWIMNEQRRRNILGEKEFFKSKLTNLMDMSY	_NILGEK(ac)EFFK_	6	-0.04209	48.004	16545000	15042000
>WP_013238539.1 two-component system response regulator	K	ESFMDVFNKLMKTVPKFGEESNSDVELVSRG	_TVPK(ac)FGEESNSDVELVSR_	4	-1.1336	127.22	127820000	187200000
>WP_013238539.1 two-component system response regulator	K	LTLLINRIMADDELFKDLDEVYYKVYKESFM	_IM(ox)ADDELFK(ac)DLDEVYYK_	9	1.0911	68.257	192950000	123410000
>WP_013238540.1 MULTISPECIES: chemotaxis protein CheX	K	MIIDGLSFRVNVAIEKCK	_VNVAIEK(ac)CK_	7	-0.27956	65.347	3546500	2444400
>WP_013238544.1 MULTISPECIES: ABC transporter ATP-binding protein	K	ENLSGMMVIRAFNTQKFEEERFDKANKDVTK	_AFNTQK(ac)FEEER_	6	0.37198	89.266	10704000	12568000
>WP_013238564.1 uroporphyrinogen decarboxylase	K	MHKNDKMTPNERLAAFMTGKP	_NDK(ac)MTPNER_	3	0.24032	72.848	10594000	20921000

>WP_013238577.1 MULTISPECIES: cytosine deaminase	K	TWALRKKMLTEEDVIKRATTALKWQIANGIQ	_MLTEEDVIK(ac)R_	9	-0.13572	120.68	5932200	3518600
>WP_013238613.1 MULTISPECIES: PLP-dependent aminotransferase family protein	K	TAKYMEMYDIEEHIQKIKEVYRHRRELMIQC	_YMEMYDIEEHIQK(ac)IK_	13	-0.19928	67.964	12070000	0
>WP_013238613.1 MULTISPECIES: PLP-dependent aminotransferase family protein	K	EVYRHRRELMIQCIEKYFPKDIKHTLPDGGL	_ELMIQCIEK(ac)YFPK_	9	-0.95853	82.543	33108000	32172000
>WP_013238613.1 MULTISPECIES: PLP-dependent aminotransferase family protein	K	DDRIIEGIKRLGEVLKK	_LGEVLK(ac)K_	6	-0.03214	94.262	53722000	40715000
>WP_013238613.1 MULTISPECIES: PLP-dependent aminotransferase family protein	K	GLRLGWICADKSIIQKYIPFKQSADLHTDIF	_SIIQK(ac)YIPFK_	5	0.48071	100.48	69798000	75071000
>WP_013238614.1 MULTISPECIES: PLP-dependent aminotransferase family protein	K	YSGMLPSNFLLPSERKLAEELNVSRSTVISA	_K(ac)LAEELNVSR_	1	-0.12288	42.743	8421300	1513700
>WP_013238616.1 MULTISPECIES: hypothetical protein	K	DKEILEILKAMQQDMKSMKQDINSVEQDISS	_AMQQDMK(ac)SMK_	7	-1.3041	98.943	8504500	5018700
>WP_013238631.1 MULTISPECIES: molecular chaperone HtpG	K	EYKIRSLIKKYSDFIKYPIKMMVKKSKLKEG	_YSDFIK(ac)YPIK_	6	0.0469	51.445	2222500	0
>WP_013238631.1 MULTISPECIES: molecular chaperone HtpG	K	QNVKADKILEINTNHKMFKSIKDAFENDKDK	_ILEINTNHK(ac)MFK_	9	5.7554	74.611	7955700	0
>WP_013238631.1 MULTISPECIES: molecular chaperone HtpG	K	GTEITLKIKESTDDEKYDEFLDEYKIRSLIK	_ESTDDEK(ac)YDEFLDEYK_	7	0.53231	51.695	9718400	13873000
>WP_013238631.1 MULTISPECIES: molecular chaperone HtpG	K	EDYNQFYMDKHFGYEKPLKVIHSSVEGVVSY	_HFGYEK(ac)PLK_	6	0.26141	97.635	141050000	119420000
>WP_013238649.1 MULTISPECIES: DUF2225 domain-containing protein	K	LKWFSQIITTHGVPSKLKDRSREQRDLIKEA	_WFSQIITTHGVPSK(ac)LK_	14	-0.55884	59.862	4731800	0
>WP_013238658.1 formate dehydrogenase H subunit alpha, selenocysteine-containing	K	EVVMKFTPKYTSSITKVPADKIIEAARIIAK	_YTSSITK(ac)VPADK_	7	-0.18958	42.095	5559900	10441000
>WP_013238658.1 formate dehydrogenase H subunit alpha, selenocysteine-containing	K	DKAFIAERTENFEDFKEVVMKFTPKYTSSIT	_TENFEDFK(ac)EVVMK_	8	-0.12312	68.809	15243000	14160000
>WP_013238658.1 formate dehydrogenase H subunit alpha, selenocysteine-containing	K	GTPILHVGKFSRGLGKFSPIEYREPAELPDK	_GLGK(ac)FSPIEYR_	4	0.7475	141.88	54924000	65685000
>WP_013238658.1 formate dehydrogenase H subunit alpha, selenocysteine-containing	K	ENFEDFKEVVMKFTPKYTSSITKVPADKIIE	_FTPK(ac)YTSSITK_	4	0.37121	95.477	187440000	199640000
>WP_013238687.1 MULTISPECIES: electron transfer flavoprotein subunit beta/FixA family protein	K	EVCSEIWEGTYLKSVKLPALITVTKDINEPR	_SVK(ac)LPALITVTK_	3	0.24592	38.279	13715000	15529000
>WP_013238689.1 FAD-binding oxidoreductase	K	GKLPTNYEQLKEEIYKTAIKYGGTITAEHGT	_EEIYK(ac)TAIK_	5	-1.1827	53.751	7747000	2131900
>WP_013238693.1 MULTISPECIES: ketol-acid reductoisomerase	K	NQTGRPGFTARRRMEKDAPIEKVGKELRSMM	_RMEK(ac)DAPIEK_	4	-1.1873	103.08	13290000	8404700
>WP_013238693.1 MULTISPECIES: ketol-acid reductoisomerase	K	AVKQADIITVLLPDEKQKQIYDESIKDNLSE	_QADIITVLLPDEK(ac)QK_	13	0.28468	101.42	67014000	32010000
>WP_013238693.1 MULTISPECIES: ketol-acid reductoisomerase	K	KLKVYYDEDADLNLLKGKKIAILGFGSQGHA	_VYYDEDADLNLLK(ac)GK_	13	-0.25327	126.63	77241000	76855000
>WP_013238694.1 MULTISPECIES: dihydroxy-acid dehydratase	K	CSDGKVTEEELEEEAKRACPGCGSCSGLFTA	_VTEEELEEEAK(ac)R_	11	0.08696	72.958	1992200	2681900
>WP_013238694.1 MULTISPECIES: dihydroxy-acid dehydratase	K	LTVTGKTVGETIKDFKVLDYEVIRSVDNAYS	_DFK(ac)VLDYEVIR_	3	1.9553	46.592	2024200	0
>WP_013238694.1 MULTISPECIES: dihydroxy-acid dehydratase	K	NIPAVVVSGGPMRAGKLNNKALDFSTCIEKV	_AGK(ac)LNNK_	3	-0.30438	137.44	69783000	54346000
>WP_013238695.1 acetolactate synthase, large subunit, biosynthetic type	K	VLSDLNKEIVAPQTEKWMEEIKNWKKDLYIE	_EIVAPQTEK(ac)WMEEIK_	9	0.22318	64.394	12124000	13533000
>WP_013238701.1 MULTISPECIES: allophanate hydrolase	K	GFFWPMDPRCKMIVPKYNPPRIWTPEGAVGI	_MIVPK(ac)YNPPR_	5	-0.08391	80.239	3828100	1889000

>WP_013238715.1 MULTISPECIES: methyltetrahydrofolate cobalamin methyltransferase	K	EAVEKKDGNYIQEIAKKQEEAGATYIDVNCG	_DGNYIQEIAK(ac)K_	10	1.1684	66.262	4240400	2145200
>WP_013238717.1 MULTISPECIES: cobalamin biosynthesis protein	K	TTGLAYPSKILSTLNKYGKGIESIRIVSVVD	_ILSTLNK(ac)YGK_	7	0.41977	52.79	4569100	5868600
>WP_013238717.1 MULTISPECIES: cobalamin biosynthesis protein	K	LYKVSANKDINDSIWKEVAKSNE	_DINDSIWK(ac)EVAK_	8	-0.9166	43.382	13255000	5098400
>WP_013238719.1 hypothetical protein	K	SFDWRGMAASKDVHEKIFRNIVSSIKDKYEF	_DVHEK(ac)IFR_	5	0.066	63.48	2741700	4901800
>WP_013238719.1 hypothetical protein	K	ELDASYAAVIDTSKIKFHEDYRKACEKNICG	_IK(ac)FHEDYRK_	2	-1.3894	84.213	180430000	176680000
>WP_013238724.1 DUF4445 domain-containing protein	K	KNLKATEKELKFLEEKELKEGIHLACETKVY	_FLEEK(ac)ELK_	5	0.4481	61.582	21781000	21410000
>WP_013238724.1 DUF4445 domain-containing protein	K	IKILDKNLKATEKELKFLEEKELKEGIHLAC	_ELK(ac)FLEEK_	3	0.12862	133.23	55336000	58726000
>WP_013238724.1 DUF4445 domain-containing protein	K	LLDIVAGLVRLGIINKRGKLLSPDNFINPNA	_LGIINK(ac)R_	6	0.62475	133.99	65192000	61583000
>WP_013238727.1 MULTISPECIES: purine-nucleoside phosphorylase	K	INLSGQNPLIGQNLEKFGPRFPDMSDAYDSH	_DHINLSGQNPLIGQNLEK(ac)FGPR_	18	0.00013	104.52	18375000	15994000
>WP_013238728.1 MULTISPECIES: translation initiation factor IF-3	K	VCKIMNYSKFLYEQSKKAKQAKKNQKIVELK	_FLYEQSK(ac)K_	7	0.15558	83.005	4291900	5147800
>WP_013238728.1 MULTISPECIES: translation initiation factor IF-3	K	LNEEIRADKIRLVGEKESEIVNTKDALNMAR	_LVGEK(ac)ESEIVNTK_	5	0.0518	151.78	41834000	29293000
>WP_013238728.1 MULTISPECIES: translation initiation factor IF-3	K	KKAKQAKKNQKIVELKEVRLSATIEENDIEI	_IVELK(ac)EVR_	5	0.0587	109.21	62235000	57662000
>WP_013238729.1 MULTISPECIES: transcriptional regulator	K	ISEKKLGQSSSSIDIKKEIKASEQRVRRAVL	_LGQSSSSIDIK(ac)K_	11	0.21912	96.591	41092000	34958000
>WP_013238729.1 MULTISPECIES: transcriptional regulator	K	LKNNTPLSQPIHINVKKFIKVLYIKSKNYKS	_NNTPLSQPIHINVK(ac)K_	14	-0.10879	117.92	44748000	56252000
>WP_013238756.1 MULTISPECIES: methylcobamide--CoM methyltransferase	K	LEVHSGRAQVVLDTIKLLKGKNKNIPIIANL	_AQVVLDTIK(ac)LLK_	9	-1.1929	72.958	2662000	0
>WP_013238760.1 2-hydroxyacyl-CoA dehydratase	K	KEKLEKEFNTTITDEKLKNSIKLRNRERKAL	_EFNTTITDEK(ac)LK_	10	1.2315	51.211	4167500	4088800
>WP_013238765.1 MULTISPECIES: YbaK/EbsC family protein	K	FGLKTQMDTYLDESLKKFEYVYPAAGSRNSA	_TQMDTYLDESLK(ac)K_	12	-1.1871	49.31	6653500	7375800
>WP_013238770.1 MULTISPECIES: hypothetical protein	K	TDKRSDIIQSIKFNDKEKLINFCRGIQKGSP	_SDIIQSIK(ac)FNDKEK_	12	-0.0872	48.704	5858400	2613400
>WP_013238770.1 MULTISPECIES: hypothetical protein	K	LPKYTDKRSDIIQSIKFNDKEKLINFCRGIQ	_SDIIQSIK(ac)FNDKEK_	8	1.0605	48.704	5858400	5226700
>WP_013238775.1 tRNA (N6-isopentenyl adenosine(37)-C2)-methylthiotransferase MiaB	K	CQMNEEDSEKLSGMLKRLGYKRTEDRSSADL	_LSGMLK(ac)R_	6	0.15646	110.61	11440000	9396200
>WP_013238777.1 MULTISPECIES: PLP-dependent aminotransferase family protein	K	MDEHGIIIEKLEEELKKNKKVKMIYTIPDFQ	_LEEELK(ac)K_	6	-0.46844	130.77	67437000	38700000
>WP_013238779.1 pyridoxal phosphate-dependent aminotransferase	K	AEDGFHLPPKEVIESKITDKTRALMISNPGN	_EVIESK(ac)ITDK_	6	1.5611	70.977	0	5436900
>WP_013238779.1 pyridoxal phosphate-dependent aminotransferase	K	RKEYQKRRDILVDGLKKIPGVVCQKPTGAFY	_RDILVDGLK(ac)K_	9	-0.94056	95.502	17322000	19252000
>WP_013238779.1 pyridoxal phosphate-dependent aminotransferase	K	LKDIEDRVIIVDSISKRYSACGARIGLVASK	_VIIVDSISK(ac)R_	9	0.43904	115.78	30217000	23111000
>WP_013238782.1 MULTISPECIES: 30S ribosomal protein S1	K	EPGVDALVHISQISHKRIDKPEDVLKIGQEI	_FANFGAFVELEPGVDALVHISQISHK(ac)R_	26	0.26271	227.66	0	129630000
>WP_013238782.1 MULTISPECIES: 30S ribosomal protein S1	K	KVIRRQNEDGYVVLSKIELERENAYKELKEA	_RQNEDGYVVLSK(ac)IELER_	12	0.66115	72.184	2915200	3038400
>WP_013238782.1 MULTISPECIES: 30S ribosomal protein S1	K	ILDIDREKKKLSLSLKKLMEDPWINVDIKYP	_LSLSLK(ac)K_	6	0.21071	94.662	8747700	9122900
>WP_013238782.1 MULTISPECIES: 30S ribosomal protein S1	K	DALVHISQISHKRIDKPEDVLKIGQEIKAKI	_RIDK(ac)PEDVLK_	4	0.14595	120.06	8986300	10987000
>WP_013238782.1 MULTISPECIES: 30S ribosomal protein S1	K	INVDIKYPVGNVVLGKVVRFANFGAFVELEP	_YPVGNVVLGK(ac)VVR_	10	-0.17312	166.14	28987000	32089000

>WP_013238782.1 MULTISPECIES: 30S ribosomal protein S1	K	LSYMDKNDTQIILGEKIKGTVISVNQKEVFL	_NDTQIILGEK(ac)IK_	10	-0.67433	140.01	41318000	37728000
>WP_013238782.1 MULTISPECIES: 30S ribosomal protein S1	K	YVVLSKIELERENAYKELKEANGNSQVLKVI	_ENAYK(ac)ELK_	5	0.13615	56.432	104340000	75068000
>WP_013238782.1 MULTISPECIES: 30S ribosomal protein S1	K	SELSWGRVGKPSDVLKIGDKIKVYILDIDRE	_VGKPSDVLK(ac)IGDK_	9	0.00151	111.83	206170000	92707000
>WP_013238782.1 MULTISPECIES: 30S ribosomal protein S1	K	SQISHKRIDKPEDVLKIGQEIKAKILEVNKD	_IDKPEDVLK(ac)IGQEIK_	9	0.1591	90.385	362490000	430140000
>WP_013238783.1 MULTISPECIES: (d)CMP kinase	K	SYTDVNRVCEVTKSLKMHFEGEKLIVNGEDL	_SLK(ac)MHFEGEK_	3	-1.0315	76.655	5027500	6121500
>WP_013238788.1 MULTISPECIES: sulfurtransferase-like selenium metabolism protein YedF	K	ATVVVDNEVSKNNVCKFAKNNSFTAKVEQKE	_NNVCK(ac)FAK_	5	0.19069	111.82	16251000	7111700
>WP_013238791.1 MULTISPECIES: molybdenum cofactor guanylyltransferase	K	SGRLSIRKIIEGTNIKYIPETIKEKYDNSLS	_IIEGTNIK(ac)YIPETIK_	8	-0.44125	30.543	0	7530000
>WP_013238792.1 MULTISPECIES: formimidoylglutamase	K	IQNHSNTVDLFKTAHKFGVKYVLAKDVTNDT	_TAHK(ac)FGVK_	4	-0.10162	99.136	136110000	90480000
>WP_013238797.1 MULTISPECIES: imidazolonepropionase	K	ELSLKLKNVKYIDILKAGGGILSTVNSVRNT	_YIDILK(ac)AGGGILSTVNSVR_	6	-0.02442	38.417	6191700	3836800
>WP_013238797.1 MULTISPECIES: imidazolonepropionase	K	GILSTVNSVRNTSLEKIQSETKKRLDLMLLH	_NTSLEK(ac)IQSETK_	6	0.33051	160.56	24864000	25375000
>WP_013238797.1 MULTISPECIES: imidazolonepropionase	K	AGSREFELSLKLKNVKYIDILKAGGGILSTV	_NVK(ac)YIDILK_	3	-0.06764	70.908	50589000	73707000
>WP_013238798.1 MULTISPECIES: urocanate hydratase	K	FRPEGRIHGKPIDEYKGKCTEGKAFQVMIEN	_IHGKPIDEYK(ac)GK_	10	-0.5013	49.423	0	7384100
>WP_013238798.1 MULTISPECIES: urocanate hydratase	K	RLLAEDREKFDKLVDKSLRRHFELIKALVDK	_LVDK(ac)SLR_	4	0.00999	105.39	9200400	6782300
>WP_013238798.1 MULTISPECIES: urocanate hydratase	K	DKLVDKSLRRHFELIKALVDKGTYFFDYGNS	_RHFELIK(ac)ALVDK_	7	1.289	111.79	12558000	16591000
>WP_013238798.1 MULTISPECIES: urocanate hydratase	K	DRGFNLTQSQTEIALKNALRYIPPKYHEQLI	_GFNLTQSQTEIALK(ac)NALR_	14	-0.22814	82.259	17205000	13066000
>WP_013238798.1 MULTISPECIES: urocanate hydratase	K	QTEIALKNALRYIPPKYHEQLIPEFLEELTT	_YIPPK(ac)YHEQLIPEFLEELTTR_	5	-1.5861	88.396	117360000	12330000
>WP_013238798.1 MULTISPECIES: urocanate hydratase	K	EAMTVKLDDELPEMPKFVEGIRRAPDRGFNL	_LDDELPEMPK(ac)FVEGIR_	10	-0.46547	92.773	323590000	168790000
>WP_013238799.1 MULTISPECIES: serine/threonine protein kinase	K	EVLNDNNISLKFTHQKFREFIYSNQSMARKK	_FTHQK(ac)FR_	5	-0.19158	94.407	12345000	17076000
>WP_013238803.1 MULTISPECIES: bifunctional glutamate N-acetyltransferase/amino-acid acetyltransferase ArgJ	K	VYTKNIVKGAPLIVTKKHLENKKAQAVIANS	_GAPLIVTK(ac)K_	8	0.70804	72.928	0	6382400
>WP_013238803.1 MULTISPECIES: bifunctional glutamate N-acetyltransferase/amino-acid acetyltransferase ArgJ	K	KLNIEAIKNAVPDLVKKLDKDGYKDASKAIM	_NAVPDLVK(ac)K_	8	0.06272	67.563	5464900	5108300
>WP_013238803.1 MULTISPECIES: bifunctional glutamate N-acetyltransferase/amino-acid acetyltransferase ArgJ	K	INEENEDYKLFLQALKKLNIEVAKMIAKDGE	_LFLQALK(ac)K_	7	0.75067	91.065	11325000	8408300
>WP_013238804.1 MULTISPECIES: N-acetyl-gamma-glutamyl-phosphate reductase	K	YKKWYKKDFEIMDLHKKSVYGLPELNRKYIK	_DFEIMDLHK(ac)K_	9	0.16862	61.353	1365300	1540900
>WP_013238814.1 MULTISPECIES: HAD-IB family hydrolase	K	IDGTLYREGLITEVFKKLVKYEVIPGERWYK	_EGLITEVFK(ac)K_	9	0.29769	61.353	3198700	0
>WP_013238818.1 MULTISPECIES: CBS domain-containing protein	K	DVVTAKSDEDILAVAKRLREKNIVSIPVIDD	_SDEDILAVAK(ac)R_	10	-0.2687	174.98	108180000	62591000
>WP_013238820.1 MULTISPECIES: arginine--tRNA ligase	K	LLNRAIERTLEIINEKNPELENKEEVAKKIG	_TLEIINEK(ac)NPELENK_	8	1.5514	39.005	1545700	12038000
>WP_013238820.1 MULTISPECIES: arginine--tRNA ligase	K	NFFVDKAAFTKDTLEKVLKEGENYGKSELGK	_DTLEK(ac)VLK_	5	-0.88801	60.478	7413700	9566100
>WP_013238820.1 MULTISPECIES: arginine--tRNA ligase	K	NAEDEGVKAARIQLVKASCQVIKNGLNLLGI	_IQLVK(ac)ASCQVIK_	5	-0.71295	80.69	8781700	10178000

>WP_013238820.1 MULTISPECIES: arginine--tRNA ligase	K	KELLRIYVKFHEEAEKDPSLDEEGRMYFKKL	_FHEEAEK(ac)DPSLDEEGR_	7	1.925	76.82	8943100	8537100
>WP_013238820.1 MULTISPECIES: arginine--tRNA ligase	K	KAPNMIAEELKSKLDKEYFEKIENLGPYLNF	_LDK(ac)EYFEK_	3	-0.06776	111.63	12337000	12477000
>WP_013238820.1 MULTISPECIES: arginine--tRNA ligase	K	SLHKDPIKELLRIYVKFHEEAEKDPSLDEEG	_IYVK(ac)FHEEAEKDPSLDEEGR_	4	0.1143	169.19	41428000	50463000
>WP_013238821.1 MULTISPECIES: UDP-glucose 4-epimerase GalE	K	SVKEVIEVSRKVTGEKIEAEIAPRRQGDPAV	_VTGEK(ac)IEAEIAPR_	5	5.244	52.265	5722500	7143000
>WP_013238821.1 MULTISPECIES: UDP-glucose 4-epimerase GalE	K	NGKGFSVKEVIEVSRKVTGEKIEAEIAPRRQ	_K(ac)VTGEKIEAEIAPR_	1	-0.42937	52.265	5722500	9454700
>WP_013238821.1 MULTISPECIES: UDP-glucose 4-epimerase GalE	K	TNPYGESKLTVEKILKWSDRAYGIKYAALRY	_ILK(ac)WSDR_	3	0.21437	143	68182000	63883000
>WP_013238833.1 hemerythrin	K	ILGMDLKIKDYFDELKLSRLKNSK	_IKDYFDELK(ac)LSR_	9	-0.65979	77.078	19365000	24703000
>WP_013238833.1 hemerythrin	K	MYDYPGYSEQKRVHDKFIEKIESLDLDEMDE	_VHDK(ac)FIEK_	4	-0.0686	128.27	540910000	474500000
>WP_013238837.1 MULTISPECIES: PHP domain-containing protein	K	VHILGYFKDISHISPKFKDFLKEMNLYRINR	_DISHISPK(ac)FK_	8	-0.61203	93.258	23750000	22351000
>WP_013238838.1 MULTISPECIES: aminopeptidase	K	AEKNGYKNIEDIMNAKSPLKPGDKVYANNKD	_NIEDIMNAK(ac)SPLKPGDK_	9	-0.45435	58.09	8558800	9952900
>WP_013238838.1 MULTISPECIES: aminopeptidase	K	KEYKYAWDKYSKKDFKILFKISDDYEKFMSK	_KDFK(ac)ILFK_	4	-0.0263	149.72	15225000	13602000
>WP_013238840.1 5-formyltetrahydrofolate cyclo-ligase	K	ELKSGAYGILEPEDTKFKVKESSIDLCYIPG	_SGAYGILEPEDTK(ac)FK_	13	-0.3859	68.944	5840100	0
>WP_013238857.1 MULTISPECIES: bifunctional 3,4-dihydroxy-2-butanone-4-phosphate synthase/GTP cyclohydrolase II	K	LLYMSQEGRGIGILNKIKAYKLQENGMDTVE	_GIGILNK(ac)IK_	7	-0.41745	105.57	4876300	3107900
>WP_013238857.1 MULTISPECIES: bifunctional 3,4-dihydroxy-2-butanone-4-phosphate synthase/GTP cyclohydrolase II	K	NGDEHVCLVKGDNFEKEPVLVRVHSECLTGD	_GDNFEK(ac)EPVLVR_	6	-2.8248	47.189	9481500	2290600
>WP_013238863.1 MULTISPECIES: response regulator	K	FEVIGEAKNGVSAITKYKQYNPDIVTMDITM	_NGVSAITK(ac)YK_	8	-1.0321	72.006	5098700	20796000
>WP_013238871.1 5-aminoimidazole-4-carboxamide ribonucleotide formyltransferase	K	GIIAPSYSKEALELLKTKKKGNYCIIQIDPN	_EALELLK(ac)TK_	7	0.86047	100.57	14734000	10833000
>WP_013238871.1 5-aminoimidazole-4-carboxamide ribonucleotide formyltransferase	K	AAISDEVDLSAAKILKASVSDGIIAPSYSKE	_ILK(ac)ASVSDGIIAPSYSK_	3	0.36547	101.15	30893000	17029000
>WP_013238871.1 5-aminoimidazole-4-carboxamide ribonucleotide formyltransferase	K	FEQKRDDAVITDDLLKNIVTDNKNMPESAKR	_RDDAVITDDLLK(ac)NIVTDNK_	12	-0.14944	130.36	46581000	40449000
>WP_013238871.1 5-aminoimidazole-4-carboxamide ribonucleotide formyltransferase	K	SPAGAAVAVPLNDVLKKAYAVSDMELSPAAI	_HVSPAGAAVAVPLNDVLK(ac)K_	18	0.17309	82.007	68283000	54433000
>WP_013238901.1 MULTISPECIES: 4Fe-4S dicluster domain-containing protein	K	CVEACPKGIIVKVEDKPNPSKCMACGICVKA	_VEDK(ac)PNPSK_	4	0.25599	109.66	108380000	16914000
>WP_013238911.1 hypothetical protein	K	LKDCVSDLDKEEKTLKILLDHWVEHNRSHEG	_TLK(ac)ILLDHWVEHNR_	3	0.54665	104.17	3221300	4009100
>WP_013238911.1 hypothetical protein	K	LDHWVEHNRSHEGEFKNWAEKSKAMDKVKTA	_SHEGEFK(ac)NWAEK_	7	-0.76687	47.429	3802900	0
>WP_013238952.1 hypothetical protein	K	KKEYADEKSAALNALKEAKKVIGGSKISIDY	_SAALNALK(ac)EAK_	8	-0.45942	89.403	9193600	8938800
>WP_013238953.1 nitrogenase iron protein	K	IGCDPKADSTVSLHEKRNVNTVLELVREKKN	_ADSTVSLHEK(ac)R_	10	0.89784	105.46	14713000	9249500
>WP_013238954.1 hydrogenase expression/formation protein HypE	K	CAFVIEEGFPMDKLEKIAAAMEKTAKEAGVK	_LEK(ac)IAAAMEK_	3	-1.149	77.062	8272500	6017300
>WP_013238954.1 hydrogenase expression/formation protein HypE	K	CEGRLVVFAPKEIAPKLVDTLHKGKYSKDAA	_EIAPK(ac)LVDTLHK_	5	-0.61701	52.555	10122000	1870600
>WP_013238954.1 hydrogenase expression/formation protein HypE	K	GFPMDKLEKIAAAMEKTAKEAGVKIAAGDTK	_IAAAMEK(ac)TAK_	7	-0.48632	57.785	16999000	13341000

>WP_013238959.1 MULTISPECIES: oxidoreductase	K	LVLNPEARFAADDIMKRLNIPSAELVRLYQI	_FAADDIMK(ac)R_	8	0.31442	104.17	7263500	1073500
>WP_013238967.1 MULTISPECIES: FAD-binding oxidoreductase	K	MDKIIAQNAAQQLGTKWPANKGSVDLIFMID	_IIAQNAAQQLGTK(ac)WPANK_	13	0.90322	51.695	0	4889900
>WP_013238970.1 MULTISPECIES: butanediol dehydrogenase	K	EDIVKEGFETLTGPEKKKHVKIIVTPDKSLL	_EGFETLTGPEK(ac)K_	11	-0.61506	137.4	48624000	52884000
>WP_013238973.1 MULTISPECIES: ABC transporter ATP-binding protein	K	ADSGSVRINNIDINEKPIEAKYQFGFVPDSP	_INNIDINEK(ac)PIEAK_	9	0.41935	40.025	7749100	7527300
>WP_013238980.1 MULTISPECIES: hypothetical protein	K	EACTVCGKKCVWCRGKYELNFKESVSEVIEL	_GK(ac)YELNFK_	2	0.04139	82.279	8140300	15983000
>WP_013238980.1 MULTISPECIES: hypothetical protein	K	ENEFYTKTDSRFAGAKFIPHIKNYKEACTVC	_FAGAK(ac)FIPHIK_	5	-0.08231	85.807	25389000	18113000
>WP_013238980.1 MULTISPECIES: hypothetical protein	K	IYELFEDNPLKYLPQKFKKHDAAVIIGVHED	_YLPQK(ac)FK_	5	0.04913	77.374	115960000	135500000
>WP_013238990.1 MULTISPECIES: acetolactate synthase, large subunit, biosynthetic type	K	KDITASITKKSCIVEKVEDLADTVREAFQIA	_SCIVEK(ac)VEDLADTVR_	6	-0.4687	79.771	6226500	3008700
>WP_013238990.1 MULTISPECIES: acetolactate synthase, large subunit, biosynthetic type	K	DGSFKMNSTELATVAKYKLPIVQLLLNNRAL	_MNSTELATVAK(ac)YK_	11	-0.50873	51.211	6364000	2829000
>WP_013238990.1 MULTISPECIES: acetolactate synthase, large subunit, biosynthetic type	K	DIDVAIKGDVKEVLQKINCKLEKADHRDWME	_EVLQK(ac)INCK_	5	-0.50041	105.14	25425000	10419000
>WP_013238990.1 MULTISPECIES: acetolactate synthase, large subunit, biosynthetic type	K	KDVQSAEVEYEPFRSKLSEIKEKKYFNLNEY	_SK(ac)LSEIK_	2	0.19822	87.696	52063000	52593000
>WP_013238990.1 MULTISPECIES: acetolactate synthase, large subunit, biosynthetic type	K	AIKGDVKEVLQKINCKLEKADHRDWMEKIKQ	_INCK(ac)LEK_	4	0.12248	126.28	74249000	2636900
>WP_013238994.1 MULTISPECIES: NADH-dependent alcohol dehydrogenase	K	EAALRNVINNAPIVLKDPKNYDARAEIMWTG	_NVINNAPIVLK(ac)DPK_	11	0.14596	86.291	8863700	7793200
>WP_013238994.1 MULTISPECIES: NADH-dependent alcohol dehydrogenase	K	TDNGTKTVGQFVKLNKDDIVKILNLAK	_LNK(ac)DDIVK_	3	0.30535	60.682	13555000	12752000
>WP_013238995.1 MULTISPECIES: DNA topoisomerase III	K	DAGREGELVARWIIEKARVNKPIKRLWISSQ	_WIIEK(ac)AR_	5	-0.43374	88.029	9580700	6431300
>WP_013238996.1 MULTISPECIES: S-ribosylhomocysteine lyase	K	NYLDMNLSMAKYLANKFLKEVLTNITDKNLN	_YLANK(ac)FLK_	5	0.64454	79.116	30918000	23485000
>WP_013239000.1 nitrogenase molybdenum-iron protein subunit alpha	K	KDSLKFLVDNYDDVPKFSVSNRQQYQFYGLL	_FLVDNYDDVPK(ac)FSVSNR_	11	0.41746	76.85	0	12444000
>WP_013239001.1 MULTISPECIES: nitrogenase iron protein	K	IEKNKNFVIPKPMSQKKLEEIFLAHGLVEQV	_NFVIPKPMSQK(ac)K_	11	0.29519	100.93	8116900	9184000
>WP_013239001.1 MULTISPECIES: nitrogenase iron protein	K	SNQAREYRSLAEAIEKNKNFVIPKPMSQKKL	_SLAEAIEK(ac)NK_	8	-0.82233	107.24	10823000	12099000
>WP_013239002.1 nitrogenase cofactor biosynthesis protein NifB	K	QNSENNLEDKNVIQLKSHKYRFAVASKSGVS	_NVIQLK(ac)SHK_	6	-1.2162	80.688	4448700	22764000
>WP_013239019.1 nitrate reductase subunit beta	K	FRENGVNLKLGVKAEKLLVDEANNPKELLLN	_AEK(ac)LLVDEANNPK_	3	1.6147	69.602	5710300	0
>WP_013239019.1 nitrate reductase subunit beta	K	AGVRANTAFLQGSSVKFDKFGLIIDERGKTN	_ANTAFLQGSSVK(ac)FDK_	12	0.9386	156.17	21184000	25108000
>WP_013239023.1 MULTISPECIES: sulfite reductase subunit C	K	EHIGYIVDRTGYQEFKKWALKDVHLDPKAEV	_TGYQEFK(ac)K_	7	-0.12214	72.038	19211000	22930000
>WP_013239023.1 MULTISPECIES: sulfite reductase subunit C	K	KACVKNCKLRSADALKFENFKVLRDDKKCIG	_SADALK(ac)FENFK_	6	0.78133	160.36	33685000	24592000
>WP_013239024.1 anaerobic sulfite reductase subunit B	K	FNVLMGFKSPEDILFKKNIEEWRKNIGVTLT	_SPEDILFK(ac)K_	8	0.19129	113.22	20498000	8909700
>WP_013239025.1 MULTISPECIES: anaerobic sulfite reductase subunit A	K	VDEIYLRNGVEDPYYKKVRDKVKFALMECEH	_NGVEDPYYK(ac)K_	9	1.1812	43.308	2194900	4095400
>WP_013239025.1 MULTISPECIES: anaerobic sulfite reductase subunit A	K	NYDLFVKHKDGKVSVKCKDESFKNYFDGMTE	_VSVK(ac)CKDESFK_	4	0.10935	62.338	7537900	13215000
>WP_013239027.1 MULTISPECIES: dihydropyrimidinase	K	VLHAFLRGNKVISSGKFIEDKPLGKYLYRKT	_VISSGK(ac)FIEDKPLGK_	6	0.02347	125.28	51181000	66082000

>WP_013239029.1 MULTISPECIES: YgeY family selenium metabolism-linked hydrolase	K	FKMAPILNELKDLNEKLINDEFLGKGTLTVS	_DLNEK(ac)LINDEFLGK_	5	0.90636	99.481	0	6901900
>WP_013239031.1 MULTISPECIES: putative selenate reductase subunit YgfK	K	FNMSVGYDLDGIKSPKMNNYIEGMRNASNTK	_SPK(ac)MNNYIEGMR_	3	1.811	42.209	0	3086400
>WP_013239031.1 MULTISPECIES: putative selenate reductase subunit YgfK	K	RKGILKDPKSCEEEYKRCLSCSNICELCVDV	_SCEEEYK(ac)R_	7	0.09718	133.12	11497000	7900100
>WP_013239031.1 MULTISPECIES: putative selenate reductase subunit YgfK	K	RITQMAQKVERKLKGKFSGIDTDMLSKLAEE	_GK(ac)FSGIDTDMLSK_	2	0.5962	96.591	13447000	12205000
>WP_013239032.1 MULTISPECIES: knotted carbamoyltransferase YgeW	K	KVKDPVKTLTEIIDSKPPRFLAK	_TLTEIIDSK(ac)PPR_	9	-0.05546	56.951	1932600	0
>WP_013239032.1 MULTISPECIES: knotted carbamoyltransferase YgeW	K	EVENVARRNAEISGGKFIKTNSMKEAFENAD	_NAEISGGK(ac)FIK_	8	0.41192	57.019	9391800	0
>WP_013239032.1 MULTISPECIES: knotted carbamoyltransferase YgeW	K	MEKLKELIQNLKKLDYKNMYSNDFFLT	_ELIQNLK(ac)K_	7	0.0587	104.45	62235000	57662000
>WP_013239037.1 (2Fe-2S)-binding protein	K	MIKFILNEKEVTSTARGSERLLDV	_FILNEK(ac)EVTSTAR_	6	0.78134	89.959	10071000	5191500
>WP_013239038.1 MULTISPECIES: aldehyde oxidase	K	TSGKYHFHVSLPEMIKRADELADYRKKRHMY	_YHFHVSLPEMIK(ac)R_	12	0.05433	128.85	25386000	27011000
>WP_013239039.1 MULTISPECIES: selenium-dependent xanthine dehydrogenase	K	AKYFRDNLQIPDPVGKLKMNQKFKRVDVDEK	_DNLQIPDPVGK(ac)LK_	11	0.6141	33.204	6412900	9718500
>WP_013239047.1 MULTISPECIES: HD domain-containing protein	K	KYRSEIMKSDGEGGLKRILQKFDSDSIRISV	_SDGEGGLK(ac)R_	8	-1.1955	85.67	12298000	14699000
>WP_013239047.1 MULTISPECIES: HD domain-containing protein	K	LKNKYMECGKGIIYPKFNEKSGHPPFIDCKY	_GIIYPK(ac)FNEK_	6	-0.25027	75.387	17365000	19959000
>WP_013239048.1 molybdenum dehydrogenase accessory protein	K	VYESLLDEGYAEEDLKRVHSPIGVSIYAQTP	_FVYESLLDEGYAEEDLK(ac)R_	17	0.67715	48.547	14306000	11444000
>WP_013239049.1 phenylacetate--CoA ligase family protein	K	KEYLKNIEEDDIKSFKYFEKIPFTLPEHIKQ	_SFK(ac)YFEK_	3	0.5035	103.74	19371000	14312000
>WP_013239060.1 FAD-dependent oxidoreductase	K	MDVKTFIDAIEQDDFKKAYKVMTKKMPFTRI	_TFIDAIEQDDFK(ac)K_	12	-0.51606	101.32	14049000	25688000
>WP_013239060.1 FAD-dependent oxidoreductase	K	IEEELAVMNKKDIKIKFNEFVDEKKLEEIVN	_IK(ac)FNEFVDEK_	2	-0.23413	110.39	150540000	202630000
>WP_013239061.1 MULTISPECIES: aldehyde oxidoreductase	K	GNATRVACENLLAAMKKDDGTYMTYDEMVAA	_VACENLLAAMK(ac)K_	11	0.09091	56.225	4432700	5938900
>WP_013239061.1 MULTISPECIES: aldehyde oxidoreductase	K	VICLPTMIEKLRPLYKEAKERVKNKNVNSGD	_LRPLYK(ac)EAK_	6	0.21787	115.78	12366000	20051000
>WP_013239061.1 MULTISPECIES: aldehyde oxidoreductase	K	DACGVQITKLPARPEKVLAALKELEK	_LPARPEK(ac)VLAALK_	7	0.20752	80.96	22211000	24187000
>WP_013239061.1 MULTISPECIES: aldehyde oxidoreductase	K	CSVILNGKVVRSCITKMKRVPDEAEIITIEG	_SCITK(ac)MK_	5	-0.72496	73.616	57167000	52184000
>WP_013239061.1 MULTISPECIES: aldehyde oxidoreductase	K	HNSNPTREDVRAWFQKHRNVCRCTGYKPLVD	_AWFQK(ac)HR_	5	0.0568	150.82	240480000	252420000
>WP_013239061.1 MULTISPECIES: aldehyde oxidoreductase	K	PTNKGDGLERPILNDKKIFQFGDAIAMVLAD	_GDGLERPILNDK(ac)K_	12	0.15428	106.13	10809000	13031000
>WP_013239063.1 MULTISPECIES: glycine dehydrogenase subunit 2	K	ENPDIVKSAPNTTIIKRLDEVKAARKPVVKW	_SAPNTTIIK(ac)R_	9	-0.27019	49.5	168140000	0
>WP_013239064.1 MULTISPECIES: aminomethyl-transferring glycine dehydrogenase	K	QMAANVTRRKNIVVSKTLNPEVRKVLKTYLR	_NIVVSK(ac)TLNPEVR_	6	-1.8218	80.522	0	5079500
>WP_013239064.1 MULTISPECIES: aminomethyl-transferring glycine dehydrogenase	K	KKLLENKILGGYDLEKQYPSYKNSMLLCVTE	_ILGGYDLEK(ac)QYPSYK_	9	0.41183	81.632	11509000	0
>WP_013239066.1 MULTISPECIES: glycine cleavage system protein T	K	SIKVRNKLLNAEVVSKKFYNKNYNK	_LLNAEVVSK(ac)K_	9	0.47111	118.4	94111000	89863000
>WP_013239067.1 MULTISPECIES: dihydropyrimidine dehydrogenase subunit A	K	DEEHAGADNIEIDYAKLDGVKFLYKMAPVEI	_SDEEHAGADNIEIDYAK(ac)LDGVK_	17	-0.46302	96.131	24272000	69974000

>WP_013239067.1 MULTISPECIES: dihydropyrimidine dehydrogenase subunit A	K	IKVVPTEVSFDENTGKKKVTLKDEETKLFNA	_VVPTEVSFDENTGK(ac)K_	14	0.10815	126.71	31127000	28486000
>WP_013239067.1 MULTISPECIES: dihydropyrimidine dehydrogenase subunit A	K	VEVSKHLVNAMEEYIKSMDKKFESSDKN	_HLVNAMEEYIK(ac)SMDK_	11	0.28017	136.48	34723000	4724300
>WP_013239067.1 MULTISPECIES: dihydropyrimidine dehydrogenase subunit A	K	CKKGCPVSTPINEVIKLFKENKIKEAGKLLF	_KGCPVSTPINEVIK(ac)LFK_	14	-0.78668	144.33	156430000	109190000
>WP_013239072.1 MULTISPECIES: NADH oxidase	K	LVLKNRLIMPPMATEKSEDDGKVSSKILEHY	_LIMPPMATEK(ac)SEDDGK_	10	-0.3706	92.575	21362000	13875000
>WP_013239072.1 MULTISPECIES: NADH oxidase	K	VDVKADLIGVGRAMYKDSEWAKRAIESF	_AMYK(ac)DSEWAK_	4	0.08426	85.533	47306000	35930000
>WP_013239073.1 MULTISPECIES: APC family permease	K	MSKENSKGINALKKNQLNFVEVIALSVA	_GINALK(ac)K_	6	0.12095	90.986	34250000	43151000
>WP_013239074.1 MULTISPECIES: cyclase	K	PPTGAIIFSIVPKAQKAPGFPVRSFAILP	_AQK(ac)APGFPVR_	3	-0.79969	95.502	6830600	8463200
>WP_013239074.1 MULTISPECIES: cyclase	K	DDYVLTVDDILEWEKKYGKIPEGSFVAFRSD	_K(ac)YGKIPEGSFVAFR_	1	-0.05063	99.011	10954000	8490700
>WP_013239074.1 MULTISPECIES: cyclase	K	FASFNPAKFETIFTHKDGFFVKQYTFAGQYG	_FETIFTHK(ac)DGFFVK_	8	-0.07732	105.03	14448000	16842000
>WP_013239074.1 MULTISPECIES: cyclase	K	EFGPDTPRFASFNPAKFETIFTHKDGFFVKQ	_FASFNPAK(ac)FETIFTHK_	8	0.56856	88.73	20479000	16332000
>WP_013239081.1 MULTISPECIES: homoserine O-succinyltransferase	K	PIEIPKNYFPHNDTAKQPRATWKSHANLLFL	_NYFPHNDTAK(ac)QPR_	10	0.13929	134.99	81829000	102610000
>WP_013239081.1 MULTISPECIES: homoserine O-succinyltransferase	K	GRKFDGMIITGAPVEKLKFEDVDYWDELTEI	_KFDGMIITGAPVEK(ac)LK_	14	-0.43657	128.26	284290000	232290000
>WP_013239083.1 MULTISPECIES: hypothetical protein	K	PKYNGVGTYMRGEKDKFLTEVLWPQGIKSVL	_DK(ac)FLTEVLWPQGIK_	2	0.58277	57.804	7350300	6404700
>WP_013239083.1 MULTISPECIES: hypothetical protein	K	KTGASLAGIANVEDLKKAPAFTVVPKMPKYN	_TGASLAGIANVEDLK(ac)K_	15	0.65981	51.888	12856000	21456000
>WP_013239084.1 MULTISPECIES: amino acid permease	K	MDTNSKANEQGQLKRTLKARHMNMIAIGG	_ANEQGQLK(ac)R_	8	-0.32806	80.239	16122000	14718000
>WP_013239084.1 MULTISPECIES: amino acid permease	K	ASRMLYSLSKEGKAPKFLSKVNKHGVPTTAL	_APK(ac)FLSK_	3	0.15202	113.5	26742000	22145000
>WP_013239097.1 MULTISPECIES: dihydrofolate reductase	K	IYKQLLPYCNKLYLTKIHSHEKGDTYFPKFD	_LYLTK(ac)IHSHEK_	5	0.13774	66.262	5377200	5028100
>WP_013239121.1 MULTISPECIES: ATP-dependent Clp protease ATP-binding subunit ClpA	K	DNILKEKIFGQDEAVKAVVRAIKRSRAGFNE	_IFGQDEAVK(ac)AVVR_	9	1.5193	62.14	0	1786900
>WP_013239136.1 MULTISPECIES: NADPH-dependent butanol dehydrogenase	K	ACTGSNPRAITEEEMKKLLQYMYNGQKVNF_	_AITEEEMK(ac)K_	8	-0.76998	105.78	22241000	8568100
>WP_013239136.1 MULTISPECIES: NADPH-dependent butanol dehydrogenase	K	LDGNKAVVVVGGGSMKRFGFLAKVEKYLKET;LKGKKAVVVVGGGSMKRFGFLDKVEEYLKEA	_AVVVVGGGSMK(ac)R_	11	0.12717	131.43	12579000	17400000
>WP_013239137.1 MULTISPECIES: copper chaperone	K	DLDSKTALVESSGEIKDADIKSAIEDVGYEV	_TALVESSGEIK(ac)DADIK_	11	0.5686	68.257	5811600	1508000
>WP_013239152.1 MULTISPECIES: MOSC domain-containing protein	K	MAKVISVNISEKKGEIKKPIGRGFFKK	_VISVNISEK(ac)K_	9	0.39301	133.81	0	30605000
>WP_013239153.1 MULTISPECIES: cyclic pyranopterin monophosphate synthase MoaC	K	HINDQGRAKMVDVSEKGNTERKAVAAASIYM	_MVDVSEK(ac)GNTER_	7	-0.66675	103.75	7867900	4834000
>WP_013239154.1 MULTISPECIES: molybdenum cofactor biosynthesis protein	K	KVEYYKVIPDEIEEIKKQLCYICDDLKLNLI	_VIPDEIEEIK(ac)K_	10	1.1561	78.342	11981000	5003600
>WP_013239154.1 MULTISPECIES: molybdenum cofactor biosynthesis protein	K	KTGPAIENMLDKSQYKVEYYKVIPDEIEEIK	_SQYK(ac)VEYYK_	4	-0.37454	57.347	21689000	15475000
>WP_013239158.1 MULTISPECIES: DNA-binding response regulator	K	GIDYYGDLRTVDTHVKRLREKLGVKSYLIAT	_TVDTHVK(ac)R_	7	0.76603	86.833	5899900	4495500
>WP_013239161.1 MULTISPECIES: GntR family transcriptional regulator	K	ELGFSSDEIIKIVEEKVQGGK	_IVEEK(ac)VQGGK_	5	1.163	74.162	3128400	4612500

>WP_013239161.1 MULTISPECIES: GntR family transcriptional regulator	K	IQRAYQELERNDIAYKQRGTGTFIKEDISMV	_NDIAYK(ac)QR_	6	0.50855	125.96	21862000	16664000
>WP_013239168.1 aldo/keto reductase	K	MRLPIINNDPSKINEKEALKIIRHAIDNGVN	_INEK(ac)EALK_	4	-0.30391	84.743	27423000	15708000
>WP_013239169.1 carbamoyl-phosphate synthase (glutamine-hydrolyzing) large subunit	K	ASVNDIDKKTSLNVIKKYGELGFSISASSGT	_TSLNVIK(ac)K_	7	0.03859	60.973	4209400	4133300
>WP_013239169.1 carbamoyl-phosphate synthase (glutamine-hydrolyzing) large subunit	K	IMGFTDEYICKLIGMKLEDLKKLREVNGIKS	_LIGMK(ac)LEDLK_	5	-0.08631	51.927	4749400	5175400
>WP_013239169.1 carbamoyl-phosphate synthase (glutamine-hydrolyzing) large subunit	K	RSSALASKAAGYPIAKIAAKIALGYTLDELK	_AAGYPIAK(ac)IAAK_	8	1.0314	37.426	9017400	9479600
>WP_013239169.1 carbamoyl-phosphate synthase (glutamine-hydrolyzing) large subunit	K	SIAVYPASDLPENVLKKIEDYTVKIAKKLNV	_TGVHSGDSIAVYPASDLPENVLK(ac)K_	23	0.16998	92.95	25254000	19267000
>WP_013239171.1 ornithine carbamoyltransferase	K	DLAKKLKEEKYSGTEKKRLQGKNIALIFEKD	_YSGTEK(ac)K_	6	0.36204	92.439	92203000	60887000
>WP_013239171.1 ornithine carbamoyltransferase	K	PAFHNTETKVGEEIYKKFGYEALEVTEEVFE	_VGEEIYK(ac)K_	7	-0.06157	98.629	130860000	102070000
>WP_013239201.1 MULTISPECIES: hypothetical protein	K	DSDIDDKLRLLLSQGKKIRAIKEAKHDLHLN	_LLLSQGK(ac)K_	7	-0.26452	72.547	6407300	5188500
>WP_013239208.1 antibiotic biosynthesis monooxygenase	K	LKVVAKACVKVGETEKFKKLSSELIRESLKE	_VGETEK(ac)FK_	6	-0.16841	128.36	82077000	41800000
>WP_013239243.1 MULTISPECIES: rubrerythrin	K	SMAHMRYLIWGDHAEKAGYPNIARLFRGIAY	_YLIWGDHAEK(ac)AGYPNIAR_	10	0.11394	76.151	20387000	5165000
>WP_013239265.1 aminotransferase class V-fold PLP-dependent enzyme	K	KLGNKYSFTIWQKSDKYNSVIRLVTSWATKE	_SDK(ac)YNSVIR_	3	0.05906	123.86	18377000	24243000
>WP_013239273.1 DUF4003 domain-containing protein	K	MVENYSELKSNFKWDTSLLKHFCAMMHA	_SNFK(ac)WDTSLLK_	4	0.24526	53.967	14441000	18994000
>WP_013239283.1 MULTISPECIES: flavodoxin family protein	K	SYGKCARVDDLSPVLKRLDTADAVVLGSPIY	_VDDLSPVLK(ac)R_	9	0.06008	92.538	13478000	24880000
>WP_013239287.1 MULTISPECIES: transcription elongation factor GreA	K	MKNILTQENIDKLREELDYRMTVKRAE	_NILTQENIDK(ac)LR_	10	-0.03142	93.959	2904800	1869500
>WP_013239287.1 MULTISPECIES: transcription elongation factor GreA	K	IDVKSADKSVIGVNSKFKIKFVEDEDEEIVS	_SVIGVNSK(ac)FK_	8	-0.06522	110.53	35084000	32127000
>WP_013239305.1 MULTISPECIES: glycerol dehydrogenase	K	GECSKNEIDRLCKKLKENKNNVVIGIGGGKI	_LK(ac)ENKNNVVIGIGGGK_	2	0.98547	45.864	5678200	0
>WP_013239305.1 MULTISPECIES: glycerol dehydrogenase	K	SKNEIDRLCKKLKENKNNVVIGIGGGKIFDT	_LKENK(ac)NNVVIGIGGGK_	5	-0.24062	85.493	38304000	10591000
>WP_013239305.1 MULTISPECIES: glycerol dehydrogenase	K	ENGIKRTRDAIEESFKGKDSLLAFEAFNGEC	_DAIEESFK(ac)GK_	8	-0.01621	111.06	60892000	54141000
>WP_013239305.1 MULTISPECIES: glycerol dehydrogenase	K	TLLEDGLKAKMAVINKVPTKAVENIIEANTY	_MAVINK(ac)VPTK_	6	0.12446	104.52	103910000	84868000
>WP_013239305.1 MULTISPECIES: glycerol dehydrogenase	K	PKNPDLVLVDSSIVAKAPVRLLVSGMGDALA	_NPDLVLVDSSIVAK(ac)APVR_	14	0.06321	205.42	400960000	246430000
>WP_013239316.1 MULTISPECIES: ferredoxin	K	YYEDKLTEIIEKNDLKSITVVRMEVPCCLGI	_NDLK(ac)SITVVR_	4	0.33121	61.344	10434000	10214000
>WP_013239316.1 MULTISPECIES: ferredoxin	K	DDIKYYEDKLTEIIEKNDLKSITVVRMEVPC	_LTEIIEK(ac)NDLK_	7	-2.347	127.87	11017000	10652000
>WP_013239316.1 MULTISPECIES: ferredoxin	K	IIERESKDYDEELVAKKAKKKEKKEVMPCGC	_DYDEELVAK(ac)K_	9	-0.18643	147.95	113820000	159540000
>WP_013239317.1 MULTISPECIES: methyl-accepting chemotaxis protein	K	NEINSTLTEIEKSVAKISEGLASTKDTFNNQ	_SVAK(ac)ISEGLASTK_	4	-0.05397	47.037	4498500	11276000
>WP_013239317.1 MULTISPECIES: methyl-accepting chemotaxis protein	K	HEIDSSINNLSELVDKLRDLSE	_IHEIDSSINNLSELVDK(ac)LR_	17	1.4656	99.891	25261000	20977000
>WP_013239317.1 MULTISPECIES: methyl-accepting chemotaxis protein	K	IIGSIVLVKSLERHNKFVEMSKVLSSAIETL	_HNK(ac)FVEMSK_	3	0.69858	169.76	110630000	82981000
>WP_013239319.1 MULTISPECIES: TIGR00268 family protein	K	CLASRFPYGNEINSQKLKMVEDAEQFLMDLG	_FPYGNEINSQK(ac)LK_	11	-0.21475	61.375	9148600	6207400
>WP_013239322.1 MULTISPECIES: glutamine--tRNA ligase/YqeY domain fusion protein	K	DTNPSKEDTEYVESIKEDVKWLGYDWDELHF	_FDDTNPSKEDTEYVESIK(ac)EDVK_	18	0.58314	96.79	10454000	9232700

>WP_013239322.1 MULTISPECIES: glutamine--tRNA ligase/YqeY domain fusion protein	K	EENLELFERMKNGEFKDGEKVLRAKIDVTSP	_NGEFK(ac)DGEK_	5	0.09748	103.7	19850000	17200000
>WP_013239326.1 DegT/DnrJ/EryC1/StrS aminotransferase family protein	K	FKLESLHGQSKDALSKMQAGAWKYDIVTDGG	_DALSK(ac)MQAGAWK_	5	-1.1511	55.452	7115200	2445000
>WP_013239326.1 DegT/DnrJ/EryC1/StrS aminotransferase family protein	K	EDAEYVVKELISAVEKVMK	_ELISAVEK(ac)VMK_	8	0.40071	66.262	8427800	7026500
>WP_013239326.1 DegT/DnrJ/EryC1/StrS aminotransferase family protein	K	AAIGLAQFPRYEEMLKKRKAIFDLYTKFLSE	_YEEMLK(ac)K_	6	-0.44323	78.496	23916000	16395000
>WP_013239326.1 DegT/DnrJ/EryC1/StrS aminotransferase family protein	K	LRIKDFSEDQRNEVIKSLAAKDIATNVHYTP	_NEVIK(ac)SLAAK_	5	-0.12756	99.53	37190000	22375000
>WP_013239327.1 DegT/DnrJ/EryC1/StrS family aminotransferase	K	VDEDTFNLDANKIEEKITSKTASILPIHLFS	_IEEK(ac)ITSK_	4	0.97731	68.016	3996300	741570
>WP_013239327.1 DegT/DnrJ/EryC1/StrS family aminotransferase	K	AAVLLVKLKYIDNALKQRQKVADWYKERLAD	_YIDNALK(ac)QR_	7	0.1204	116.52	71496000	53973000
>WP_013239338.1 MULTISPECIES: (Fe-S)-cluster assembly protein ;>WP_013240397.1 hypothetical protein	K	ASDQLIKLMSLGLSEKEAEDTILKGFLK	_LMSLGLSEK(ac)EAEDTILK_	9	0.61297	50.079	12072000	12395000
>WP_013239338.1 MULTISPECIES: (Fe-S)-cluster assembly protein ;>WP_013240397.1 hypothetical protein	K	VQIKGVSSTKRDTEIKLHKNARAVVSERLLT	_DTEIK(ac)LHK_	5	0.11065	101.99	17006000	10444000
>WP_013239338.1 MULTISPECIES: (Fe-S)-cluster assembly protein ;>WP_013240397.1 hypothetical protein	K	MSLGLSEKEAEDTILKGFLK	_EAEDTILK(ac)GFLK_	8	-0.05103	78.692	19047000	14580000
>WP_013239344.1 MULTISPECIES: DUF362 domain-containing protein	K	MKKSKVYFTDFRTKLGEGLPTKLKKLIKK	_TK(ac)LGEGLPTK_	2	0.04941	92.866	13380000	0
>WP_013239351.1 MULTISPECIES: radical SAM protein	K	YNNDKSKHEFIQDILKNTDTKCLKKFFTNFF	_HEFIQDILK(ac)NTDTK_	9	0.8181	93.823	2564900	19183000
>WP_013239351.1 MULTISPECIES: radical SAM protein	K	EDFVPYAEKTWDEVFKKKGNDNFAKG	_TWDEVFK(ac)K_	7	-0.08139	86.011	20394000	22075000
>WP_013239351.1 MULTISPECIES: radical SAM protein	K	MKIKNALEKNTKQAIVNKIVNYLDKNP	_NTK(ac)QAIVNK_	3	0.1722	110.76	238320000	156000000
>WP_013239374.1 MULTISPECIES: hydroxylamine reductase	K	DTVILTAGCAKYKYNKLNLGDIGGIPRILDA	_YNK(ac)LNLGDIGGIPR_	3	-1.0637	76.262	0	4026500
>WP_013239376.1 glycine/betaine reductase C ;>WP_013239421.1 glycine/betaine reductase C	K	AAGLKEIIGKILEKEKPAAAVEEVKIPPKKV	_EK(ac)PAAAVEEVK_	2	0.67084	43.991	6460000	4734300
>WP_013239376.1 glycine/betaine reductase C ;>WP_013239421.1 glycine/betaine reductase C	K	GDFEIVEVSDAEEGHKKMVELLDNKVIDGCV	_VEGDFEIVEVSDAEEGHK(ac)K_	18	-0.64691	70.942	11652000	14372000
>WP_013239376.1 glycine/betaine reductase C ;>WP_013239421.1 glycine/betaine reductase C	K	ANAAGLKEIIGKILEKEKPAAAVEEVKIPPK	_ILEK(ac)EKPAAAVEEVK_	4	-0.29577	126.77	38863000	39954000
>WP_013239377.1 glycine/betaine reductase C	K	AVSKEEIKNMIAEELKKFASKMMEE	_NMIAEELK(ac)K_	8	-1.766	54.608	1123600	1581200
>WP_013239377.1 glycine/betaine reductase C	K	FTEAVREKFKSHAVLKIKADELKEGIDLSEI	_SHAVLK(ac)IK_	6	0.34566	90.37	1971300	18460000
>WP_013239378.1 MULTISPECIES: glycine/sarcosine/betaine reductase complex component A2	K	GDRDGIPGPAIEECVKSAKGEVVFASTECFV	_DGIPGPAIEECVK(ac)SAK_	13	-0.11469	48.547	0	7146700
>WP_013239380.1 thioredoxin-disulfide reductase ;>WP_013239425.1 thioredoxin-disulfide reductase	K	VHRRDELRAAKSIQEKAFKNPKIEIKWNSVI	_SIQEK(ac)AFK_	5	0.7088	73.931	12556000	8454300
>WP_013239389.1 MULTISPECIES: GNAT family N-acetyltransferase	K	ELPCVFNNYAVFYKGKFETVNLLDIGSLKRI	_GK(ac)FETVNLLDIGSLK_	2	-1.3857	44.807	2010000	4946500
>WP_013239422.1 MULTISPECIES: glycine/betaine reductase C	K	CAGPSHSIVNAAALVKAGVYKNVIVFAGGST	_GFCAGPSHSIVNAAALVK(ac)AGVYK_	18	-1.3376	56.529	20040000	17887000

>WP_013239422.1 MULTISPECIES: glycine/betaine reductase C	K	TETVREKFKARPILSKKADELKEGVCLADIE	_ARPILSK(ac)K_	7	0.03726	128.12	33778000	32522000
>WP_013239422.1 MULTISPECIES: glycine/betaine reductase C	K	TVSKEEIKSMIAEEFKKFAAQMMEE	_SM(ox)IAEEFK(ac)K_	8	-0.55743	82.287	34068000	25174000
>WP_013239424.1 MULTISPECIES: thiol reductase thioredoxin	K	MVILDKNTFKDEVLETKGYVLVDYF	_NTFK(ac)DEVLETK_	4	-0.31233	119.03	56353000	38402000
>WP_013239424.1 MULTISPECIES: thiol reductase thioredoxin	K	KQYGNQVKFCKLNTTKARRLAISQRVLGLPT	_LNTTK(ac)AR_	5	-0.19603	107.59	8965500	0
>WP_013239424.1 MULTISPECIES: thiol reductase thioredoxin	K	VEELSKQYGNQVKFCKLNTTKARRLAISQRV	_FCK(ac)LNTTK(ac)AR_	3	0.28017	124.98	362750000	213140000
>WP_013239427.1 MULTISPECIES: glycine/betaine/sarcosine/D-proline family reductase selenoprotein B	K	THTIICGDNFMGSNEKEAVSRILGFLEGKEF	_DAEVTHTIICGDNFMGSNEK(ac)EAVSR_	20	-0.28085	107.08	20664000	13999000
>WP_013239427.1 MULTISPECIES: glycine/betaine/sarcosine/D-proline family reductase selenoprotein B	K	GYYGRGKRHQIWLESKEPAAKRVVKMLIKKL	_HQIWLESK(ac)EPAAK_	8	0.42625	130.24	82354000	94991000
>WP_013239427.1 MULTISPECIES: glycine/betaine/sarcosine/D-proline family reductase selenoprotein B	K	DPNVIVPLDALRAYEKEGKIGKIHEYFYSTV	_AYEK(ac)EGK_	4	0.07805	79.116	9519300	8404000
>WP_013239428.1 MULTISPECIES: glycine reductase complex component B subunit alpha	K	FGETTNYKEGILTINKKEALDYVLEDEHITE	_EGILTINK(ac)K_	8	0.23863	90.64	23992000	19219000
>WP_013239433.1 DUF1177 domain-containing protein	K	AFGKNNCSFYDEDEFKKLNEIYGSMTKFQK	_NNCSFYDEDEFK(ac)K_	12	0.37841	44.616	2046200	21371000
>WP_013239435.1 DUF917 domain-containing protein	K	QVKETSIKGIISTTEKVGRAIRIAKSNNTNP	_GIISTTEK(ac)VGR_	8	1.5694	79.659	4600200	4965600
>WP_013239453.1 2-hydroxy-3-oxopropionate reductase	K	DMSSIAPLVSREIAKKLEEKGIDMLDAPVSG	_K(ac)LEEKGIDMLDAPVSGGEPK_	1	-0.2205	79.568	0	22367000
>WP_013239453.1 2-hydroxy-3-oxopropionate reductase	K	AKAPMVLDRNFKPGFKIDLHIKDLKNAVETA	_NFKPGFK(ac)IDLHIK_	7	-0.01279	44.426	4281300	5470200
>WP_013239453.1 2-hydroxy-3-oxopropionate reductase	K	LHLDGEGQSDHSALVKFYEKLGKVKVTK	_NLHLDGEGQSDHSALVK(ac)FYEK_	17	0.0226	57.989	13832000	6979500
>WP_013239453.1 2-hydroxy-3-oxopropionate reductase	K	IAPLVSREIAKKLEEKGIDMLDAPVSGGEPK	_LEEK(ac)GIDMLDAPVSGGEPK_	4	-2.5249	97.488	22529000	22367000
>WP_013239453.1 2-hydroxy-3-oxopropionate reductase	K	LVVYNRSKASEEELEKAGAERGASPKDVAAK	_ASEEELEK(ac)AGAER_	8	0.18195	180.09	24535000	19125000
>WP_013239457.1 glucarate dehydratase	K	TIEDSKSLVVGQSIGKYKSIIRNVKETFSSR	_SLVVGQSIGK(ac)YK_	10	-0.21093	75.479	7996700	5198200
>WP_013239469.1 MULTISPECIES: adenosine deaminase	K	HAGETGIVENIMKSIKLLHAERIGHGTYAYK	_SIK(ac)LLHAER_	3	0.24261	52.79	5890800	1758900
>WP_013239473.1 MULTISPECIES: hypothetical protein	K	ELGIALKQRMQLQQAKKRKISSAFFTFVEQT	_MQLQQAK(ac)K_	7	-0.61385	86.953	20782000	10483000
>WP_013239475.1 MULTISPECIES: HesB-like protein	K	MDFLKISDIAYQEFKKFLDENNATSGAIRI	_ISDIAYQEFK(ac)K_	10	-0.04832	57.019	11217000	13471000
>WP_013239475.1 MULTISPECIES: HesB-like protein	K	FNISVDEKKDHDLIQKVKDITFIVDSDLFVQ	_KDHDLIQK(ac)VK_	8	0.09248	204.17	33887000	29590000
>WP_013239483.1 MULTISPECIES: class I SAM-dependent methyltransferase	K	NERIEENNLQGKVEVKLADYRELADKKQKFD	_VEVK(ac)LADYR_	4	0.19199	54.608	4655900	27467000
>WP_013239513.1 MULTISPECIES: shikimate dehydrogenase	K	VRGANVSMPNKTVVHKYLDKLSEAAELCGSV	_TVVHK(ac)YLDK_	5	0.65641	91.658	21837000	22802000
>WP_013239514.1 MULTISPECIES: type I 3-dehydroquinate dehydratase	K	MKSIVKVKNVKLGEGIPKIAVPFMGR	_NVK(ac)LGEGIPK_	3	-0.12033	51.927	21879000	17996000
>WP_013239529.1 MULTISPECIES: hypothetical protein	K	SKDEGRHARILEGILKKYNK	_ILEGILK(ac)K_	7	0.09516	87.616	4159600	4471100
>WP_013239529.1 MULTISPECIES: hypothetical protein	K	ANNAKHEDALKAEEQKIDAAKEFFNSASKDE	_AEEQK(ac)IDAAK_	5	0.26738	50.284	9831700	10213000
>WP_013239539.1 MULTISPECIES: CBS domain-containing protein	K	GDLLWKLKNTPDLNFKNTSKVLLKQISRRMH	_NTPDLNFK(ac)NTSK_	8	0.03187	95.417	17927000	19493000
>WP_013239539.1 MULTISPECIES: CBS domain-containing protein	K	NTPDLNFKNTSKVLLKQISRRMHNKPVKINS	_VLLK(ac)QISR_	4	-0.31846	103.56	27448000	13323000
>WP_013239542.1 MULTISPECIES: cardiolipin synthase	K	NSESPLFTNNSIKIFKSGVEKFKYLKRELLN	_IFK(ac)SGVEK_	3	-0.70245	66.073	0	21764000

>WP_013239543.1 MULTISPECIES: fructose-bisphosphatase class III	K	GKVRMTTFERYFIDDKIAHYE KKNYYYQYRD	_YFIDDK(ac)IAHYEK_	6	-0.10441	43.991	3181100	3325400
>WP_013239543.1 MULTISPECIES: fructose-bisphosphatase class III	K	QFKLEAEIIKRHPEFKMDDRL LLDKIDIEKW	_RHPEFK(ac)MDDR_	6	1.1917	86.624	13879000	24671000
>WP_013239543.1 MULTISPECIES: fructose-bisphosphatase class III	K	DKDILSSTVILEHVTKRKRVE DTDIGKDLTK	_NAIEEDKDILSSTVIL EHVTK(ac)R_	21	0.23402	189.79	282050000	263340000
>WP_013239544.1 MULTISPECIES: phosphoglucomutase	K	MAKLHKQGKSIEDLIKSLKIS PESLEFRFDI	_SIEDLIK(ac)SLK_	7	0.05251	73.499	5564000	17120000
>WP_013239544.1 MULTISPECIES: phosphoglucomutase	K	EDIKYILDSAVEKDLKYPSYK GEISKVDFID	_DLK(ac)YPSYK_	3	-0.26453	81.594	7416000	8055700
>WP_013239544.1 MULTISPECIES: phosphoglucomutase	K	RKGINSNEDYEMPLSKFKIIV DAGNGAGGFF	_GINSNEDYEMPLSK(ac)FK_	14	0.00369	44.807	9617700	8243000
>WP_013239544.1 MULTISPECIES: phosphoglucomutase	K	QNGTDIRGIAIENPEKKVNLT AVEVKAIAGG	_GIAIENPEK(ac)K_	9	-0.63927	97.456	16864000	15014000
>WP_013239550.1 MULTISPECIES: shikimate dehydrogenase	K	SEEAKNIGAINTIEFKKNSLK GYNTDYYGFG	_NIGAINTIEFK(ac)K_	11	-0.07395	155.48	252180000	151810000
>WP_013239551.1 MULTISPECIES: prephenate dehydratase	K	KNWKLNPYFNTARSAKYISE QNVKSKAAIAS	_SAK(ac)YISEQNVK_	3	0.43894	146.71	39531000	29858000
>WP_013239551.1 MULTISPECIES: prephenate dehydratase	K	FNTARSAKYISEQNVKSKAAI ASKNAAKLYG	_YISEQNVK(ac)SK_	8	-0.15803	123.79	128730000	114070000
>WP_013239552.1 MULTISPECIES: chorismate synthase	K	GRLTAPIVFAGALCRKILEEK GIFIGSHIKS	_K(ac)ILEEKGIFIGSHI K_	1	-0.15371	42.338	10194000	5438500
>WP_013239552.1 MULTISPECIES: chorismate synthase	K	GTIEDPKSFKFTSIEKSSLEK LSESKFPVLD	_FTSIEK(ac)SSLEK_	6	0.67663	57.836	13943000	0
>WP_013239552.1 MULTISPECIES: chorismate synthase	K	FTSIEKSSLEKLSESKFPVLD ENCGEKMQQY	_LSESK(ac)FPVLDEN CGEK_	5	-0.26348	74.772	18518000	3326400
>WP_013239552.1 MULTISPECIES: chorismate synthase	K	PKSFKFTSIEKSSLEKLSESK FPVLDENCGE	_SSLEK(ac)LSESK_	5	-0.1074	72.652	23830000	7998300
>WP_013239552.1 MULTISPECIES: chorismate synthase	K	PIVFAGALCRKILEEKGIFIGS HIKSIGTIE	_ILEEK(ac)GIFIGSHIK _	5	0.52007	92.935	35376000	4177400
>WP_013239552.1 MULTISPECIES: chorismate synthase	K	KTASLLRPGHADFTGKIKYRG FNDYRGGGHF	_TASLLRPGHADFTG K(ac)IK_	15	-0.33525	154.82	72078000	73725000
>WP_013239552.1 MULTISPECIES: chorismate synthase	K	SHIKSIGTIEDPKSFKFTSIEK SSLEKLSES	_SFK(ac)FTSIEK_	3	1.0015	99.815	744480000	565650000
>WP_013239553.1 MULTISPECIES: 3-phosphoshikimate 1-carboxyvinyltransferase	K	AVIRILKDMGVEIEEKGDTLK VNPSETHGTY	_DMGVEIEEK(ac)GDT LK_	9	-1.0333	83.499	7549100	6329100
>WP_013239553.1 MULTISPECIES: 3-phosphoshikimate 1-carboxyvinyltransferase	K	FYRNFQGKLPLTINGKLKPGE YRVKGNISSQ	_LPLTINGK(ac)LKPG EYR_	8	0.2761	73.91	7628100	8715500
>WP_013239553.1 MULTISPECIES: 3-phosphoshikimate 1-carboxyvinyltransferase	K	IKESDRLKAISTELNKIGADV KEREDGLIIN	_AISTELNK(ac)IGADV K_	8	-0.26964	56.482	9298800	7993300
>WP_013239553.1 MULTISPECIES: 3-phosphoshikimate 1-carboxyvinyltransferase	K	MKYVSIKPTKLKGQIQVPPS KSVCH	_YVSIKPTK(ac)LK_	8	0.01107	81.338	12385000	8675500
>WP_013239554.1 MULTISPECIES: 3-dehydroquinate synthase	K	ILKKYGLPVYIQGVDKNVLVD TIALDKKSSD	_YGLPVYIQGVDK(ac) NVLVDTIALDKK_	12	-0.15342	43.888	0	21961000
>WP_013239554.1 MULTISPECIES: 3-dehydroquinate synthase	K	YKCCSIKKQLVEEDEKDLGN RMLLNFGHTLG	_QLVEEDEK(ac)DLG NR_	8	-0.71075	59.898	5098400	0
>WP_013239554.1 MULTISPECIES: 3-dehydroquinate synthase	K	SLDMLKKIYENLCELKIKRKD VIVSLGGGVV	_IYENLCELK(ac)IK_	9	1.14	78.548	11358000	24312000
>WP_013239554.1 MULTISPECIES: 3-dehydroquinate synthase	K	YKIYMERGILTSIGKKLRQVY EGRTLVVITD	_GILTSIGK(ac)K(ac)L R_	9	-0.27028	120.09	24389000	20744000
>WP_013239554.1 MULTISPECIES: 3-dehydroquinate synthase	K	GFIKDKTILEDLNSYKSKEEA LNNIENIIYK	_TILEDLNSYK(ac)SK _	10	0.25763	68.809	33613000	23792000
>WP_013239554.1 MULTISPECIES: 3-dehydroquinate synthase	K	QGVDKNVLVDTIALDKKSSD KNEINLIMVEE	_NVLVDTIALDK(ac)K _	11	-0.2324	116.01	43649000	25663000
>WP_013239554.1 MULTISPECIES: 3-dehydroquinate synthase	K	DNGFKVNVISIEPGEKSKSLD MLKKIYENLC	_VNVISIEPGEK(ac)S K_	11	0.24556	117.92	46519000	56060000
>WP_013239554.1 MULTISPECIES: 3-dehydroquinate synthase	K	ISIEPGEKSKSLDMLKKIYEN LCELKIKRKD	_SLDMLK(ac)K_	6	-0.0838	81.784	51556000	2072200

>WP_013239554.1 MULTISPECIES: 3-dehydroquinate synthase	K	LYMKELKQSFLDNGFKVNVISIEPGEKSKSL	_QSFLDNGFK(ac)VNVISIEPGEK_	9	0.2259	141.31	210720000	207140000
>WP_013239554.1 MULTISPECIES: 3-dehydroquinate synthase	K	QYKIYMERGILTSIGKKLRQVYEGRTLVVIT	_GILTSIGK(ac)K(ac)LR_	8	-0.27028	120.09	251160000	242950000
>WP_013239555.1 MULTISPECIES: prephenate dehydrogenase/arogenate dehydrogenase family protein	K	AIISKDVDSIKNIFEKASSSRKELVKG	_NIFEK(ac)ASSSR_	5	-0.08093	61.353	4896200	3126700
>WP_013239555.1 MULTISPECIES: prephenate dehydrogenase/arogenate dehydrogenase family protein	K	TPTAKNTSRGINLIGKMARKIGCSNVTCISP	_GINLIGK(ac)MAR_	7	-0.52687	87.913	7315100	20917000
>WP_013239555.1 MULTISPECIES: prephenate dehydrogenase/arogenate dehydrogenase family protein	K	LVEEIEKFEESINLLKKAIISKDVDSIKNIF	_FEESINLLK(ac)K_	9	0.30702	44.425	8973100	0
>WP_013239555.1 MULTISPECIES: prephenate dehydrogenase/arogenate dehydrogenase family protein	K	ALYPKDTVKFVKDNFKYLKSGAIITDTSGIK	_DNFK(ac)YLK_	4	0.24402	92.467	26678000	9293700
>WP_013239555.1 MULTISPECIES: prephenate dehydrogenase/arogenate dehydrogenase family protein	K	EIDLIIIALYPKDTVKFVKDNFKYLKSGAII	_DTVK(ac)FVK_	4	0.08111	105.39	26976000	12827000
>WP_013239562.1 MULTISPECIES: ACT domain-containing protein	K	MLVDLSKMKTDFIELKNHLENKAKEIGVVIK	_TDFIELK(ac)NHLENK_	7	1.4671	128.76	15252000	18387000
>WP_013239562.1 MULTISPECIES: ACT domain-containing protein	K	KNHLENKAKEIGVVIKLQREDIFTSMHQI	_EIGVVIK(ac)LQR_	7	0.8863	89.047	21291000	15731000
>WP_013239563.1 PFL family protein	K	FKITRMGQLVAGEASKRLSVPFGIVDLSLAP	_MGQLVAGEASK(ac)R_	11	-1.0898	48.659	5020700	4567400
>WP_013239563.1 PFL family protein	K	FIARGGRIPAPIHSFKN	_IPAPIHSFK(ac)N_	9	-0.31861	157.34	3.617E+09	2.751E+09
>WP_013239565.1 MULTISPECIES: chemotaxis protein	K	DRYVDASNKVYTVSEKSVRAAAEVLFIVANA	_VYTVSEK(ac)SVR_	7	0.6582	61.353	6284100	13477000
>WP_013239568.1 MULTISPECIES: N-acetyltransferase	K	RQSHWAKNRPAEVIAKTIKHSLCFAIYHNDI	_NRPAEVIAK(ac)TIK_	9	0.28303	78.324	4239700	5820900
>WP_013239568.1 MULTISPECIES: N-acetyltransferase	K	YRRRGLGKKLIEFINKYPAIKDTSKFLWTKY	_LIEFINK(ac)YPAIK_	7	0.56821	87.363	9478700	5662700
>WP_013239578.1 MULTISPECIES: 4Fe-4S dicluster domain-containing protein	K	DLRKLIIESLNQVGEKILKDTNEKNILYQRY	_LIIESLNQVGEK(ac)ILK_	12	0.69883	38.028	4497000	5275900
>WP_013239582.1 MULTISPECIES: aspartate/glutamate racemase family protein	K	VGLLGTKFTMEEDFYKKVLKDKFNINVSIPD	_FTMEEDFYK(ac)K_	9	-0.06038	107.24	14237000	3023900
>WP_013239582.1 MULTISPECIES: aspartate/glutamate racemase family protein	K	VIHQIIYNELCKGIIKDPSREKYKKIINKLS	_EVIHQIIYNELCKGIIK(ac)DPSR_	17	-0.41073	56.081	15290000	0
>WP_013239582.1 MULTISPECIES: aspartate/glutamate racemase family protein	K	ICTNTMHKLAPDIESKVGIKVLHIAEAAGKK	_LAPDIESK(ac)VGIK_	8	0.58599	67.456	15677000	16555000
>WP_013239582.1 MULTISPECIES: aspartate/glutamate racemase family protein	K	KFTMEEDFYKKVLKDKFNINVSIPDENDREV	_DK(ac)FNINVSIPDENDR_	2	1.6642	183.6	41020000	22315000
>WP_013239582.1 MULTISPECIES: aspartate/glutamate racemase family protein	K	SKVGIKVLHIAEAAGKKIIEKNIKTVGLLGT	_VLHIAEAAGK(ac)K_	10	-0.04614	131.82	43758000	36341000
>WP_013239582.1 MULTISPECIES: aspartate/glutamate racemase family protein	K	WEELSNIMVNAAQSLKKGGADFIIICTNTMH	_WEELSNIMVNAAQSLK(ac)K_	16	0.54869	93.754	46344000	47283000
>WP_013239582.1 MULTISPECIES: aspartate/glutamate racemase family protein	K	NDREVIHQIIYNELCKGIIKDPSREKYKKII	_EVIHQIIYNELCK(ac)GIIKDPSR_	13	0.05088	139.11	57287000	44965000
>WP_013239582.1 MULTISPECIES: aspartate/glutamate racemase family protein	K	FESTLEYYRIINETVKKTLGGLHSAECILYS	_IINETVK(ac)K_	7	-0.2566	71.379	79098000	76813000
>WP_013239582.1 MULTISPECIES: aspartate/glutamate racemase family protein	K	HIAEAAGKKIIEKNIKTVGLLGTKFTMEEDF	_NIK(ac)TVGLLGTK_	3	-0.28325	129	665100000	643620000

>WP_013239594.1 ArsR family transcriptional regulator	K	MNQQEVSVRIFKALGHPIRY KIVKFLY	_IFK(ac)ALGHPIR_	3	-0.04074	55.567	5314000	10920000
>WP_013239603.1 esterase	K	ILKNIDMMVEICEKNKFVPIIL TPLPVKDDI	_NK(ac)FVPIILTPLPV K_	2	0.08688	33.685	5305200	12008000
>WP_013239606.1 MULTISPECIES: site-2 protease family protein	K	FAHAFVADRLGDKTAKFQGR LTLNPFAHIDI	_TAK(ac)FQGR_	3	-0.68435	81.297	20207000	0
>WP_013239622.1 FAD-dependent oxidoreductase	K	LENRLRFAREIVEEIKNRCGE DFPVVLRYSP	_EIVEEIK(ac)NR_	7	0.44697	78.934	4527000	4179000
>WP_013239622.1 FAD-dependent oxidoreductase	K	KDLRDGALPGEEFVEKGRDL DEGIEAAKLLV	_DGALPGEEFVEK(ac)GR_	12	-1.5551	65.092	5435600	3981300
>WP_013239622.1 FAD-dependent oxidoreductase	K	PQACKEKDNALLPILKKKKV LIVGGGVAGCE	_EKDNALLPILK(ac)K _	11	-0.28639	94.191	14083000	9271200
>WP_013239622.1 FAD-dependent oxidoreductase	K	RWLDKICRELTIDEIKSIVKKF GDGAYNAKR	_ELTIDEIK(ac)SIVK_	8	-0.03205	54.524	16343000	11692000
>WP_013239622.1 FAD-dependent oxidoreductase	K	IKSIVKKFGDGAYNAKRAGFD GIEIHAVHEG	_FGDGAYNAK(ac)R_	9	-0.85659	140.75	23876000	20927000
>WP_013239622.1 FAD-dependent oxidoreductase	K	LGDDDKVFTAADVLTKKKDC GGTTVVVGGGL	_VFTAADVLTK(ac)K_	10	0.07056	147.37	31260000	22442000
>WP_013239642.1 MULTISPECIES: response regulator	K	ASGYIRKPFHTETLLKTLEEY K	_KPFHTETLLK(ac)TL EEYK_	10	-0.60929	83.54	9239400	15235000
>WP_013239650.1 MULTISPECIES: D-2-hydroxyacid dehydrogenase	K	EYFKNQQPLKNIVDFKTGYR VNKKKV	_NIVDFK(ac)TGYR_	6	-0.39247	60.434	15308000	12264000
>WP_013239660.1 flavodoxin family protein	K	EEIKETQGGFFSDSEKRAVV NKKLEKYEKIQ	_ETQGGFFSDSEK(ac) R_	12	-0.26255	40.552	4570400	0
>WP_013239660.1 flavodoxin family protein	K	KIGDNIMKAVNTPEEKRTWL GDEDMGWCPNC	_AVNTPEEK(ac)R_	8	0.08457	126.07	29705000	24356000
>WP_013239663.1 MULTISPECIES: FAD-dependent oxidoreductase	K	PGIIIAVDEDVPKEVKFGTKL VDFLDTAKVK	_EVK(ac)FGTK_	3	-0.1556	72.138	3872200	0
>WP_013239663.1 MULTISPECIES: FAD-dependent oxidoreductase	K	GYESYFEARTVEELAKKLNI DAEKLQDTIDE	_TVEELAK(ac)K_	7	0.9145	61.962	16052000	12885000
>WP_013239688.1 MULTISPECIES: GntR family transcriptional regulator	K	ELIEAFEKKDEKLAEKIGRKH TINAKKFIEK	_LAEK(ac)IGR_	4	0.77319	90.986	7256100	0
>WP_013239698.1 MULTISPECIES: 3-phosphoglycerate dehydrogenase	K	INWTKSLKGKGEDVVKLVEK SKSQFQGPEIK	_GKGEDVVK(ac)LVE K_	8	-0.05647	113.72	12192000	12437000
>WP_013239712.1 FAD-binding protein	K	VSLGTASNKELVEEIKTAFDK VVLLGDAAKA	_ELVEEIK(ac)TAFDK _	7	0.5277	60.628	3806000	0
>WP_013239712.1 FAD-binding protein	K	PVIGVSVIREPEFAEKVLEEE KVDFIGSARQ	_EPEFAEK(ac)VLEE EK_	7	-0.23425	76.262	6502500	5776900
>WP_013239712.1 FAD-binding protein	K	QNLIDVMGRIAEHEIKLYPKH KLVKIDGTTV	_IAEHEIK(ac)LYPK_	7	-0.21023	88.441	10963000	9387700
>WP_013239804.1 ImmA/IrrE family metallo-endopeptidase	K	EELGHHHKTSGEILGKKNINK IKQEKMARNW	_TSGEILGK(ac)K_	8	-0.35431	56.432	12410000	10080000
>WP_013239812.1 MULTISPECIES: cob(I)yrinic acid a,c-diamide adenosyltransferase	K	VFFGQFVKGMEYSELKAKEY LPNFTLEQFGR	_GMEYSELK(ac)AK_	8	-0.09774	96.89	14285000	10830000
>WP_013239812.1 MULTISPECIES: cob(I)yrinic acid a,c-diamide adenosyltransferase	K	ADKRIIERADLVTEMKEIKHY YTEGVPARVG	_ADLVTEMK(ac)EIK_	8	-0.529	116.74	110860000	87114000
>WP_013239814.1 cobalt-precorrin-6A reductase	K	NCAFIKDYEAEAMLLKDSGR EGGTYEKIRAC	_DYEAEAMLLK(ac)D SGR_	10	0.60565	112.72	7622100	0
>WP_013239814.1 cobalt-precorrin-6A reductase	K	IEYIRYERPSCIEEFKSEDKV VEVKDYDELK	_YERPSCIEEFK(ac)S EDK_	11	-0.10469	96.993	25059000	22567000
>WP_013239815.1 sirohydrochlorin cobaltochelatase	K	KEYAVRRAFTSGIIIKKLKNQ KKIYIDTVPE	_RAFTSGIIIK(ac)K_	10	-0.6376	76.332	43305000	40959000
>WP_013239817.1 cobalt-precorrin 5A hydrolase	K	PDVFAKENGFIINDLKDAKYI AALLVEGKKV	_ENGFIINDLK(ac)DAK _	10	-2.2953	45.864	1087600	3852200
>WP_013239818.1 MULTISPECIES: precorrin-4 C(11)-methyltransferase	K	LKKGYGRNVPIAVIQKATWE DEKCVIGTLDD	_NVPIAVIQK(ac)ATW EDEK_	9	-1.7556	43.791	10148000	7890700
>WP_013239818.1 MULTISPECIES: precorrin-4 C(11)-methyltransferase	K	YFIGAGPGNADLITVKGRDILK TADVVIYAG	_VYFIGAGPGNADLIT VK(ac)GR_	17	0.04823	82.007	10516000	9780900
>WP_013239818.1 MULTISPECIES: precorrin-4 C(11)-methyltransferase	K	TIILTRVEGRTPVPEKENLER LASVGASMAL	_TPVPEK(ac)ENLER_	6	0.08033	122.7	69507000	62641000

>WP_013239818.1 MULTISPECIES: precorrin-4 C(11)-methyltransferase	K	LNEVIDVIKKYHGEGKKIVRLHTGDPTIYGA	_YHGEGK(ac)K_	6	0.41329	116.88	90777000	140920000
>WP_013239819.1 cobalt-factor II C(20)-methyltransferase	K	SQVDKITDEKFVVIMKVYRKEENVLNVLEEK	_FVVIM(ox)K(ac)VYR_	6	-0.66609	71.223	3855600	2048700
>WP_013239819.1 cobalt-factor II C(20)-methyltransferase	K	KVYRKEENVLNVLEEKGFKYICVKRAYREGQ	_KEENVLNVLEEK(ac)GFK_	12	0.19181	94.487	14501000	12214000
>WP_013239819.1 cobalt-factor II C(20)-methyltransferase	K	YICVKRAYREGQQEVLKNREDILKSRDYMSLI	_EGQEVLK(ac)NR_	7	0.00197	72.23	23126000	16476000
>WP_013239819.1 cobalt-factor II C(20)-methyltransferase	K	PMGGKEQEGKIFEAFKTIEERLNEGKNVAFL	_IFEAFK(ac)TIEER_	6	0.09164	132.76	32336000	27372000
>WP_013239819.1 cobalt-factor II C(20)-methyltransferase	K	DTAKNFVDENKEVIIKHFPMGGKEQEGKIFE	_EVIIK(ac)HFPMGGK_	5	-0.35217	125.97	49351000	33879000
>WP_013239819.1 cobalt-factor II C(20)-methyltransferase	K	YREGQQEVLKNREDILKSRDYMSLIIAQRD	_NREDILK(ac)SR_	7	-0.51572	141.1	76927000	57993000
>WP_013239819.1 cobalt-factor II C(20)-methyltransferase	K	PGDEELLTLKAVNVIKKCEVIVAPSAKDGDR	_AVNVIK(ac)K_	6	0.01425	86.086	150740000	71664000
>WP_013239820.1 precorrin-6Y C5,15-methyltransferase (decarboxylating) subunit CbiT	K	NFITLNNLYKALDTLKSMKCEVECIQVSINK	_ALDTLK(ac)SMK_	6	-0.1147	86.624	18575000	12270000
>WP_013239820.1 precorrin-6Y C5,15-methyltransferase (decarboxylating) subunit CbiT	K	EKNEEALSVIEKNIDKFNVNNLTVVKGEALE	_NIDK(ac)FNVNNLTVVK_	4	0.81796	53.491	25817000	0
>WP_013239821.1 precorrin-6y C5,15-methyltransferase (decarboxylating) subunit CbiE	K	LGIVKENRISIWFTDKKHSPDYLCSILEENA	_ISIWFTDK(ac)K_	8	0.68433	60.518	3744400	1187300
>WP_013239821.1 precorrin-6y C5,15-methyltransferase (decarboxylating) subunit CbiE	K	PGNKDYILPKAAVVMKRSQAILGFERAVDSL	_AAVVM(ox)K(ac)R_	6	0.36183	88.591	8525600	6798100
>WP_013239822.1 cobalamin biosynthesis protein CbiD	K	KIDTPKGIELVIPIEKVRHGKDFVECCVLKD	_GIELVIPIEK(ac)VR_	10	-0.20017	43.991	0	3680600
>WP_013239823.1 MULTISPECIES: precorrin-8X methylmutase	K	LYKLKELTLAGKAKPKFIVGAPVGFVGAADS	_AKPK(ac)FIVGAPVGFVGAADSK_	4	0.37556	58.49	2444700	22197000
>WP_013239823.1 MULTISPECIES: precorrin-8X methylmutase	K	PVGFVGAADSKEEIEKLDVPMITIRGRKKGGS	_EEIEK(ac)LDVPMITIR_	5	-2.3281	65.092	7060900	4180900
>WP_013239823.1 MULTISPECIES: precorrin-8X methylmutase	K	MDYIKSPMGIEKRSFEIIGEEMGECNF	_SPMGIEK(ac)R_	7	-0.45296	126.41	37113000	20075000
>WP_013239823.1 MULTISPECIES: precorrin-8X methylmutase	K	EMGECNFPERELSIVKRVIHTTGDFQYKDIL	_ELSIVK(ac)R_	6	-0.10813	110.63	98704000	72430000
>WP_013239823.1 MULTISPECIES: precorrin-8X methylmutase	K	TALYKLKELTLAGKAKPKFIVGAPVGFVGAA	_AK(ac)PKFIVGAPVGFVGAADSK_	2	0.60281	91.637	120770000	75734000
>WP_013239824.1 nicotinate-nucleotide--dimethylbenzimidazole phosphoribosyltransferase	K	IKAIEAGIESVDDLVKKGYDLFGTGEMGVGN	_AIEAGIESVDDLVK(ac)K_	14	0.30647	74.678	4732900	0
>WP_013239824.1 nicotinate-nucleotide--dimethylbenzimidazole phosphoribosyltransferase	K	DLTVGKGSGITDEQLKSKKKAVQAAIDVNKP	_GSGITDEQLK(ac)SK_	10	0.19182	134.99	49925000	45986000
>WP_013239824.1 nicotinate-nucleotide--dimethylbenzimidazole phosphoribosyltransferase	K	DNMATFEEADVKNSDKFIDIREK	_NSDK(ac)FIDIR_	4	0.35998	112.75	76709000	79249000
>WP_013239825.1 cobyrinate a,c-diamide synthase	K	DVEMLLRCFKENKNFKDDYHIENRKIRTAVA	_NFK(ac)DDYHIENR_	3	-0.05764	66.262	4833000	1989100
>WP_013239825.1 cobyrinate a,c-diamide synthase	K	LSSNISMMESIKTELKSGLRCYAECGGLMYL	_TELK(ac)SGLR_	4	-0.32325	107.66	16335000	17543000
>WP_013239826.1 porphobilinogen synthase	K	LIDEKAIVKEMLTCIKRAGAGMIITYYALDI	_EMLTCIK(ac)R_	7	0.39084	87.616	2283100	3779600
>WP_013239826.1 porphobilinogen synthase	K	FAMVKAAAKAGLIDEKAIVKEMLTCIKRAGA	_AGLIDEK(ac)AIVK_	7	-0.04935	115.78	22631000	16377000
>WP_013239826.1 porphobilinogen synthase	K	SAFYGPFRDAADSAPKFGDRKGYQMDPANVR	_DAADSAPK(ac)FGDRK_	8	-0.53184	213.16	743900000	676930000
>WP_013239827.1 uroporphyrinogen-III C-methyltransferase	K	YKNQDEINNMLVELAKKGHVVGRIKGGDPYV	_NQDEINNMLVELAK(ac)K_	14	1.1929	91.207	11980000	5838900

>WP_013239827.1 uroporphyrinogen-III C-methyltransferase	K	LIFLMGFRNLEVICSKLKENGMDENTPCAVV	_NLEVICSK(ac)LK_	8	0.27195	80.763	19823000	13895000
>WP_013239827.1 uroporphyrinogen-III C-methyltransferase	K	VTHRKVARSFHIFTGKTAEKLDINWEAAAKI	_SFHIFTGK(ac)TAEK_	8	0.34288	155.48	51013000	73588000
>WP_013239827.1 uroporphyrinogen-III C-methyltransferase	K	ERLLTENYDIRNIKGKFAAIGPATERAIKSK	_GK(ac)FAAIGPATER_	2	0.68874	116.54	125500000	100550000
>WP_013239828.1 hydroxymethylbilane synthase	K	MNFKIATRKSKLAQTQTEIVMDILQK	_SK(ac)LAQTQTEIVMDILQK_	2	-0.11322	33.836	4924200	0
>WP_013239828.1 hydroxymethylbilane synthase	K	GIETKKDSKNKSFFSKLDDENTRICVEAERS	_SFFSK(ac)LDDENTR_	5	0.31132	136.7	19275000	8569100
>WP_013239828.1 hydroxymethylbilane synthase	K	KDVTGNKEDYMVLGEKLAKMILKA	_EDYMVLGEK(ac)LAK_	9	1.2141	83.758	130390000	64800000
>WP_013239830.1 aspartate aminotransferase	K	ESQKYKCVFLIRALTKFFALPGIRMGYGLCK	_ALTK(ac)FFALPGIR_	4	0.1271	55.452	4470100	3935900
>WP_013239830.1 aspartate aminotransferase	K	QIIHSHLKENMEYDYKDIGQKITKSEALIIG	_ENMEYDYK(ac)DIGQK_	8	0.16431	43.382	5042100	7555200
>WP_013239830.1 aspartate aminotransferase	K	RCLKEDVLIRRCNNFKELDSKFIRLAVKDRW	_RCNNFK(ac)ELDSK_	6	-0.30207	88.441	8782100	8902000
>WP_013239830.1 aspartate aminotransferase	K	LSFKENIEKALKDVEKYPDVEYRQLKNSIKE	_DVEK(ac)YPDVEYR_	4	-0.31129	86.313	9130600	10616000
>WP_013239830.1 aspartate aminotransferase	K	GIRMGYGLCKNENLIKSIKSKQNPWNINSFA	_NENLIK(ac)SIK_	6	-0.31044	58.592	9445700	10003000
>WP_013239830.1 aspartate aminotransferase	K	AVKDRWRNERILQVLKKISKDVTT	_ILQVLK(ac)K_	6	0.52599	86.866	18119000	16419000
>WP_013239832.1 MULTISPECIES: cobyric acid synthase	K	RKNLDIDKIYNIMGIKR	_IYNIMGIK(ac)R_	8	-0.03166	111.04	5375800	7175600
>WP_013239832.1 MULTISPECIES: cobyric acid synthase	K	KDILEPGLKMLEDIVKVPCLGVIPHFRLALE	_MLEDIVK(ac)VPCLGVIPHFR_	7	0.7163	87.148	8930000	11399000
>WP_013239832.1 MULTISPECIES: cobyric acid synthase	K	IKLPHISNFTDLDALKSEEDVSVRFITSVEE	_LPHISNFTDLDALK(ac)SEEDVSVR_	14	-0.2741	69.398	15590000	13457000
>WP_013239832.1 MULTISPECIES: cobyric acid synthase	K	HGVFDGIDFREFIVNKIRENKGLGYKKSKTY	_EFIVNK(ac)IR_	6	-0.46007	127.84	44367000	48293000
>WP_013239832.1 MULTISPECIES: cobyric acid synthase	K	NMKLEFRDMLKEHFEKMEKKFDIIVMEGAGS	_EHFEK(ac)MEK_	5	-0.61658	123.35	70063000	50490000
>WP_013239832.1 MULTISPECIES: cobyric acid synthase	K	FTEEEKKRVKGTIINKFRGDKDILEPGLKML	_GTIINK(ac)FR_	6	0.39615	128.57	106160000	118640000
>WP_013239832.1 MULTISPECIES: cobyric acid synthase	K	LVIIPGSKNTIEDLLKIRQCGLEDRIKEYSK	_NTIEDLLK(ac)IR_	8	0.17088	150.61	396970000	381260000
>WP_013239832.1 MULTISPECIES: cobyric acid synthase	K	EFNKRVTAPIDIAVIKLPHISNFTDLDALKS	_VTAPIDIAVIK(ac)LPHISNFTDLDALK_	11	-5.5438	165.4	495510000	751140000
>WP_013239833.1 MULTISPECIES: ABC transporter ATP-binding protein	K	AFGPRQMGMDEDEVNKRVNDCLELLNVKDLE	_QMGMDEDEVNK(ac)R_	11	-0.1841	53.967	4851700	5216400
>WP_013239835.1 MULTISPECIES: NUDIX hydrolase	K	QLQEITYTGKNNEELKWERIKRTNTTNTVVI	_NNEELK(ac)WER_	6	0.30076	51.927	3230600	2427600
>WP_013239835.1 MULTISPECIES: NUDIX hydrolase	K	MKRISEKTLYKGKWIQLQEITY	_ISEK(ac)TLYK_	4	0.10953	58.433	9410100	14231000
>WP_013239840.1 MULTISPECIES: MetQ/NlpA family ABC transporter substrate-binding protein	K	AHEGIIKVKSGDLISKLDITENKKNLKFQEL	_SGDLISK(ac)LDITENKK_	7	0.2538	45.33	5537700	15901000
>WP_013239840.1 MULTISPECIES: MetQ/NlpA family ABC transporter substrate-binding protein	K	DLISKLDITENKKNLKFQELDAPQLPRTLTE	_NLK(ac)FQELDAPQLPR_	3	-0.02531	86.291	9396900	19085000
>WP_013239840.1 MULTISPECIES: MetQ/NlpA family ABC transporter substrate-binding protein	K	IKVGASPQPHAEILEKVKPILKKQGYDLQIV	_VGASPQPHAEILEK(ac)VKPILK_	14	-0.41029	38.017	9995200	5687600
>WP_013239840.1 MULTISPECIES: MetQ/NlpA family ABC transporter substrate-binding protein	K	EIAIPNDPTNGARALKLLAHEGIIKVKSGDL	_ALK(ac)LLAHEGIIK_	3	-0.52173	126.95	77229000	29385000
>WP_013239841.1 MULTISPECIES: D-alanine--D-alanine ligase	K	GSDEIVVELEKSLQEKVEKICVQCWEVLKCK	_SLQEK(ac)VEK_	5	0.90885	83.005	8332400	0
>WP_013239842.1 MULTISPECIES: alanine racemase	K	TSDEKDKTYSQEQFEKYKTFYNELINRKIKI	_TYSQEQFEK(ac)YK_	9	-0.25906	52.693	9629100	5578700

>WP_013239848.1 MULTISPECIES: nitroreductase	K	AVEKGFGGCMLGNVNKKELKSNLNLDEKYEI	_GFGGCMLGNVNK(ac)K_	12	-0.74696	45.915	8277400	11329000
>WP_013239848.1 MULTISPECIES: nitroreductase	K	LVLKNRSCRRFYQNEKITVETLKYLVNLARL	_RFYQNEK(ac)ITVETLK_	7	1.2809	132.47	19034000	15765000
>WP_013239848.1 MULTISPECIES: nitroreductase	K	MIKELVLKNRSCRRFYQNEKITV	_ELVLK(ac)NR_	5	0.11926	144.29	190530000	133830000
>WP_013239849.1 MULTISPECIES: lactate dehydrogenase	K	KVLAYSHRQDETEYFKKFSKKYDVEVVLCDD	_QDETEYFK(ac)K_	8	-0.19874	60.518	12478000	5598100
>WP_013239849.1 MULTISPECIES: lactate dehydrogenase	K	CISIITTKISDKLVEKFHEIGVKFISTRTIG	_LVEK(ac)FHEIGVK_	4	0.31229	123.95	19532000	21908000
>WP_013239849.1 MULTISPECIES: lactate dehydrogenase	K	KFISTRTIGYDHIDIKKAKELGVHIGNVNYS	_TIGYDHIDIK(ac)K_	10	0.48515	164.9	57857000	64666000
>WP_013239873.1 ATP-binding protein	K	DRPCLDSPSKFDIIFKTEGIKYAYGFSVTSE	_FDIIFK(ac)TEGIK_	6	3.2644	76.655	2855300	0
>WP_013239873.1 ATP-binding protein	K	YKFTNDIEKQTAIKDKFNSPNKLFIAVASLW	_DK(ac)FNSPNK_	2	0.30085	71.379	7394500	1804700
>WP_013239873.1 ATP-binding protein	K	QELTMDLMKSNESINKRVKSLLKTVVSDIED	_SNESINK(ac)R_	7	0.10634	93.429	14468000	11487000
>WP_013239895.1 MULTISPECIES: type II toxin-antitoxin system Phd/YefM family antitoxin	K	KVAKLVDESKSVVIMKNNKPKYVLLDFDEFS	_SVVIMK(ac)NNKPK_	6	0.15927	85.377	23487000	11565000
>WP_013239899.1 BREX-1 system adenine-specific DNA-methyltransferase PglX	K	YWLSKDMKEAFKRGIKLANIAEVKQGLATSD	_GIK(ac)LANIAEVK_	3	-0.08387	64.64	6419400	0
>WP_013239899.1 BREX-1 system adenine-specific DNA-methyltransferase PglX	K	YDIYKSAGYSEREIPKLILEKNIYGLDIDDR	_EIPK(ac)LILEK_	4	-0.71219	53.08	11828000	0
>WP_013239900.1 BREX system P-loop protein BrxC	K	FYEEDSIVEALSKTTKMSEETARDWYNKAEE	_TTK(ac)MSEETAR_	3	-0.02936	57.019	7729300	6899100
>WP_013239900.1 BREX system P-loop protein BrxC	K	DANIKTVIIHAKNNSKLNEEDVEVLKLLFMI	_NNSK(ac)LNEEDVEVLK_	4	0.15363	126.71	16072000	18506000
>WP_013239932.1 MULTISPECIES: universal stress protein	K	PEKYCVFGRVADEILKKANCDEFDMIIMTKS	_VADEILK(ac)K_	7	0.3511	82.75	14616000	10577000
>WP_013239936.1 MULTISPECIES: NUDIX domain-containing protein	K	AVGSIVKAVQVAVEKKPLIIGKPHSYMFKKA	_K(ac)PLIIGK_	1	0.05107	84.244	9296900	10306000
>WP_013239947.1 MULTISPECIES: threonine synthase	K	EKLMKSEVIHSTVCEKQQMKDEIRKFLV	_SEVIHSTVCEK(ac)QQMK_	11	-0.35014	35.891	0	4704700
>WP_013239947.1 MULTISPECIES: threonine synthase	K	DAQTEVKKIFNDEELKKELDEKSYMFSSANS	_IFNDEELK(ac)K_	8	-2.2505	54.608	3559900	16385000
>WP_013239947.1 MULTISPECIES: threonine synthase	K	LAYDNKFESDEIVPLKKVGDVFYLELFHGPT	_FESDEIVPLK(ac)K_	10	-0.06917	59.542	11181000	13230000
>WP_013239947.1 MULTISPECIES: threonine synthase	K	GVSDIQKRQMVTQVGKNTKVIGIKGNFDDAQ	_QMVTQVGK(ac)NTK_	8	-0.10973	80.245	15611000	7326500
>WP_013239947.1 MULTISPECIES: threonine synthase	K	VIGIKGNFDDAQTEVKKIFNDEELKKELDEK	_GNFDDAQTEVK(ac)K_	11	-0.19598	84.289	16990000	17781000
>WP_013239947.1 MULTISPECIES: threonine synthase	K	DIQKRQMVTQVGKNTKVIGIKGNFDDAQTEV	_NTK(ac)VIGIK_	3	0.83254	71.692	19122000	17072000
>WP_013239947.1 MULTISPECIES: threonine synthase	K	CIIGKFFTDFTEDELKKCIDLAYDNKFESDE	_FFTDFTEDELK(ac)K_	11	0.1571	72.089	35586000	44471000
>WP_013239947.1 MULTISPECIES: threonine synthase	K	FVPDSIPSVNEEELQKFKNMTYKELALCIIG	_GISEDGGLFVPDSIPSVNEEELQK(ac)FK_	24	-0.4619	35.32	41220000	11363000
>WP_013239947.1 MULTISPECIES: threonine synthase	K	VMSKTASLEIPKPIEKLMKSEVIHSTVCEKQ	_TASLEIPKPIEK(ac)LMK_	12	0.09576	127.36	45469000	54016000
>WP_013239947.1 MULTISPECIES: threonine synthase	K	RIVVFFPEKGVSDIQKRQMVTQVGKNTKVIG	_GVSDIQK(ac)R_	7	-0.07448	130.27	58271000	44736000
>WP_013239947.1 MULTISPECIES: threonine synthase	K	YYFHGYMELLRKGQIKLGEKINFVVPTGNFG	_GQIK(ac)LGEK_	4	0.41325	120.09	188160000	46057000
>WP_013239947.1 MULTISPECIES: threonine synthase	K	FADVEDTRIVVFFPEKGVSDIQKRQMVTQVG	_IVVFFPEK(ac)GVSDIQK_	8	0.59024	100.45	293060000	232980000
>WP_013239948.1 MULTISPECIES: aminotransferase class V-fold PLP-dependent enzyme	K	GLGEGIRYIYDLGVQKIRDKENEIIEYALKR	_YIYDLGVQK(ac)IR_	9	0.22913	50.284	10491000	9588300

>WP_013239950.1 MULTISPECIES: lipoyl synthase	K	EVRPMAVYKRSLELLKRVKIMDNSILTKSGF	_SLELLK(ac)R_	6	-0.31428	97.071	5543200	6797300
>WP_013239953.1 MULTISPECIES: cold-shock protein	K	DVEEGPKGLQAANVVKL	_GLQAANVVK(ac)L_	9	0.15924	85.731	104410000	32140000
>WP_013239963.1 MULTISPECIES: hypothetical protein	K	NNLSKLMELVPNIVSKLKKKNEKKENI	_LM(ox)ELVPNIVSK(ac)LK_	11	1.7172	53.252	12908000	10748000
>WP_013239967.1 MULTISPECIES: GTP 3',8-cyclase MoaA	K	PVDIRFIELMPIGEGKKLYENDNLSSEVVLK	_FIELMPIGEGK(ac)K_	11	-5.6098	73.848	2720500	7498400
>WP_013239967.1 MULTISPECIES: GTP 3',8-cyclase MoaA	K	VKRPYGDLLRFEDILKIIKAAATLGINKIRY	_FEDILK(ac)IIK_	6	-0.46071	56.432	2958800	0
>WP_013239967.1 MULTISPECIES: GTP 3',8-cyclase MoaA	K	LKHCLNDEKLLLEELKRGILGKPEEHNMSSK	_LLLEELK(ac)R_	7	0.33511	94.114	9561000	5791700
>WP_013239976.1 MULTISPECIES: amidohydrolase	K	EKNAEKIINFSKSVEKEPELGFKEQKTSKKV	_SVEK(ac)EPELGFK_	4	0.14083	48.741	0	3880400
>WP_013239976.1 MULTISPECIES: amidohydrolase	K	KTSKKVKGIFDEIGLKYRDGLALTGVKAKLK	_GIFDEIGLK(ac)YR_	9	-5.2928	69.01	4612000	3735300
>WP_013239980.1 MULTISPECIES: phosphatase	K	HTGNPKFPIHAEEVVKKAKEKSILIEINNSS	_FPIHAEEVVK(ac)K_	10	0.05972	99.522	7016700	12786000
>WP_013239989.1 MULTISPECIES: DNA polymerase I	K	EGFKVDKDKLVEQEGKFKIEIDKVQKEIYSL	_LVEQEGK(ac)FK_	7	1.5627	56.563	2294600	1978100
>WP_013239994.1 MULTISPECIES: aspartate--tRNA ligase	K	ERMPYKIAMEKYGSDKPDLRFGMEINDISEV	_YGSDK(ac)PDLR_	5	0.13373	53.683	5102400	1839400
>WP_013239994.1 MULTISPECIES: aspartate--tRNA ligase	K	DLRDRTGILQIVFGEKINKEAFEKSDNVKSE	_TGILQIVFGEK(ac)INK_	11	-0.75556	50.155	12042000	11137000
>WP_013239994.1 MULTISPECIES: aspartate--tRNA ligase	K	RFIQRVFKKILNVDVKLPIERMPYKIAMEKY	_ILNVDVK(ac)LPIER_	7	-0.26621	85.845	21113000	16647000
>WP_013239994.1 MULTISPECIES: aspartate--tRNA ligase	K	IKVPNSAKMGRKQIDKLVEFVKDYGAKGLVW	_QIDK(ac)LVEFVK_	4	-0.02406	48.741	25500000	23208000
>WP_013239994.1 MULTISPECIES: aspartate--tRNA ligase	K	VWIAYKEDGIKSSISKFLTEEDTKNILNKLE	_SSISK(ac)FLTEEDTK_	5	0.11141	150.21	33019000	19051000
>WP_013239995.1 MULTISPECIES: histidine--tRNA ligase	K	KLFYFTPVLRYENVQKGRLREHHQFGIEAFG	_YENVQK(ac)GR_	6	0.1014	73.665	12250000	12695000
>WP_013239995.1 MULTISPECIES: histidine--tRNA ligase	K	SIGCATCRKKYNEALKKFLKENYDALCDTCK	_YNEALK(ac)K_	6	1.022	97.798	17095000	16526000
>WP_013239997.1 MULTISPECIES: MBL fold hydrolase	K	HGHADHTSAAEEIRNKYNAPIAISREDYNMM	_NK(ac)YNAPIAISR_	2	3.9525	84.507	5918000	5917000
>WP_013239997.1 MULTISPECIES: MBL fold hydrolase	K	YNAPIAISREDYNMMKSRELMYGKLIDEVDI	_EDYNMMK(ac)SR_	7	-0.04207	86.882	19561000	15139000
>WP_013239997.1 MULTISPECIES: MBL fold hydrolase	K	DEMVVFPGHGSKTSIKEEKMYNPFL	_TSIK(ac)EEK_	4	0.19234	95.352	80985000	56977000
>WP_013239999.1 MULTISPECIES: bifunctional (p)ppGpp synthetase/guanosine-3',5'-bis(diphosphate) 3'-pyrophosphohydrolase	K	ILIKLADRLHNMRTLKYMPVKKQKEKAKETF	_TLK(ac)YMPVK_	3	-0.1018	73.665	8963200	17659000
>WP_013240000.1 MULTISPECIES: adenine phosphoribosyltransferase	K	SFKDITTLLQDKTAFKHTVNKIARYLKNKHI	_TAFK(ac)HTVNK_	4	0.5597	66.568	5849500	5355400
>WP_013240000.1 MULTISPECIES: adenine phosphoribosyltransferase	K	TTLLQDKTAFKHTVNKIARYLKNKHIDVVVG	_HTVNK(ac)IAR_	5	0.17261	107.9	98466000	77403000
>WP_013240015.1 MULTISPECIES: YebC/PmpR family DNA-binding transcriptional regulator	K	AIAAKEGGSNPDSNSKLRDVIAKAKANNMPQ	_EGGSNPDSNSK(ac)LR_	11	-0.52402	59.185	13200000	11465000
>WP_013240015.1 MULTISPECIES: YebC/PmpR family DNA-binding transcriptional regulator	K	MSGHSKWHNIQAKKGKADAKRGKIFTKI	_WHNIQAK(ac)K_	7	0.25482	138.83	59388000	66999000
>WP_013240016.1 MULTISPECIES: YigZ family protein	K	SYFTVKDEASCQFEEKKSIFIGSIKRVYTEN	_DEASCQFEEK(ac)K_	10	-0.09891	91.855	5499000	3242600
>WP_013240016.1 MULTISPECIES: YigZ family protein	K	IFIGSIKRVYTENEAKKFISKVKEKNIKATH	_VYTENEAK(ac)K_	8	-0.38146	122.13	11627000	14384000
>WP_013240016.1 MULTISPECIES: YigZ family protein	K	SCQFEEKKSIFIGSIKRVYTENEAKKFISKV	_SIFIGSIK(ac)R_	8	0.40125	157.91	15774000	17432000
>WP_013240020.1 MULTISPECIES: hypoxanthine phosphoribosyltransferase	K	RIGKRIEEVGKIITTKYKDKNLYVLSLLRGS	_IITTK(ac)YK_	5	0.3764	84.244	439580000	316550000

>WP_013240035.1 MULTISPECIES: UDP-N-acetylglucosamine 2-epimerase (non-hydrolyzing)	K	SNLLFVPTESAVENLKKEGITKGVYNVGDVM	_VLTDHLSNLLFVPTESAVENLK(ac)K_	22	0.31228	32.391	0	4242700
>WP_013240035.1 MULTISPECIES: UDP-N-acetylglucosamine 2-epimerase (non-hydrolyzing)	K	MKVLTVVGARPQFIKAAAVSNVIRKHHTEI	_VLTVVGARPQFIK(ac)AAAVSNVIR_	13	0.02449	40.012	3706200	10548000
>WP_013240036.1 MULTISPECIES: UDP-3-O-(3-hydroxymyristoyl)glucosamine N-acyltransferase	K	GKEPMRSVNSIFKDEKKFDPALIKDGCLIGA	_SVNSIFKDEK(ac)K_	10	0.29217	131.43	0	7208400
>WP_013240037.1 gfo/Idh/MocA family oxidoreductase	K	KDADDMIECSKKNNVKLCVSHQNRFNKPVQQ	_NNVK(ac)LCVSHQNR_	4	-0.05265	36.847	922380	6084900
>WP_013240037.1 gfo/Idh/MocA family oxidoreductase	K	MALSIKDADDMIECSKKNNVKLCVSHQNRFN	_DADDMIECSK(ac)K_	10	-0.12091	48.741	2320700	15052000
>WP_013240037.1 gfo/Idh/MocA family oxidoreductase	K	LVEEKAEQRKNEYIEKVGDAKVAVYTDYKKM	_KNEYIEK(ac)VGDAK_	7	-0.19414	122.64	14851000	11842000
>WP_013240038.1 MULTISPECIES: nucleotide sugar dehydrogenase	K	KAKAGYEVIGFDVQDKKVESVNEGHNYIGDI	_AGYEVIGFDVQDK(ac)K_	13	-0.01266	65.805	4823500	8329200
>WP_013240038.1 MULTISPECIES: nucleotide sugar dehydrogenase	K	MSQLRDELIQKLEDRKAKLGVVGLGY	_DELIQK(ac)LEDR_	6	0.61241	96.015	6746700	4970400
>WP_013240038.1 MULTISPECIES: nucleotide sugar dehydrogenase	K	EGHNYIGDIVDSDLEKLVKEGRLKATTDFSF	_KVESVNEGHNYIGDIVDSDLEK(ac)LVK_	22	0.39272	99.064	49842000	26289000
>WP_013240038.1 MULTISPECIES: nucleotide sugar dehydrogenase	K	NGAEVIVSDPFIPEFKRNGKVYHTVKWEDVI	_NGAEVIVSDPFIPEFK(ac)R_	16	-0.02492	174.95	99194000	83277000
>WP_013240046.1 MULTISPECIES: polysaccharide biosynthesis protein	K	EVALTSTEHDKIFVEKPAKIDMTFVEESIKQ	_IFVEK(ac)PAK_	5	0.26103	56.721	3816100	13638000
>WP_013240046.1 MULTISPECIES: polysaccharide biosynthesis protein	K	FGNVLGSNGSVIPLFKKQIAHGGPVTVTHPE	_FGNVLGSNGSVIPLFK(ac)K_	16	-0.73788	51.695	7976100	7264900
>WP_013240047.1 MULTISPECIES: UTP--glucose-1-phosphate uridylyltransferase	K	RDELKKPFMKYLLDLKEQKIFKEVYDDITSN	_YLLDLK(ac)EQK_	6	-1.12	62.823	12387000	11933000
>WP_013240049.1 MULTISPECIES: phospho-sugar mutase	K	GNSLKDAEEKMNVFEKRVMKIIDDVNC	_MNVFEK(ac)R_	6	-2.0962	91.065	4140900	3532600
>WP_013240049.1 MULTISPECIES: phospho-sugar mutase	K	DAKIVKKLDYKLSVEKDLININENIIHLPKS	_LSVEK(ac)DLININENIIHLPK_	5	1.3756	54.103	14518000	29660000
>WP_013240049.1 MULTISPECIES: phospho-sugar mutase	K	KRTLGDAKIVKKLDYKLSVEKDLININENII	_LDYK(ac)LSVEK_	4	1.1585	91.584	33529000	37447000
>WP_013240049.1 MULTISPECIES: phospho-sugar mutase	K	VKNLAIRKDLIEAHAKDLKIIYTPLHGTGNI	_DLIEAHAK(ac)DLK_	8	-0.7599	63.408	102430000	111140000
>WP_013240064.1 MULTISPECIES: alpha-hydroxy-acid oxidizing protein	K	ILKGIMTPDEAELAVKAGVSAIVVSNHGGRV	_GIMTPDEAELAVK(ac)AGVSAIVVSNHGGR_	13	1.7096	169.6	0	143400000
>WP_013240064.1 MULTISPECIES: alpha-hydroxy-acid oxidizing protein	K	FGGQREGVKVYVENLKSELKSAMVLTGCKSV	_VYVENLK(ac)SELK_	7	0.26272	56.225	8030900	6830700
>WP_013240066.1 MULTISPECIES: endonuclease MutS2	K	SEAAKIKEKYEEKASKLQNIRDKAIIGAQRE	_ASK(ac)LQNIR_	3	0.3527	85.064	8964200	11491000
>WP_013240067.1 MULTISPECIES: peptidase	K	IKVPNIVKNEFNYIEKFIDKNLDKIAGIVTA	_NEFNYIEK(ac)FIDK_	8	-0.72004	71.548	13444000	2918800
>WP_013240068.1 MULTISPECIES: cell division protein ZapA	K	MVAQNKELKFQVQSGKYKIIDLQHKLEENQI	_FQVQSGK(ac)YK_	7	0.24206	64.04	5992500	4147800
>WP_013240068.1 MULTISPECIES: cell division protein ZapA	K	YMNENTKMVAQNKELKFQVQSGKYKIIDLQH	_ELK(ac)FQVQSGK_	3	-0.9248	75.509	9028600	15495000
>WP_013240068.1 MULTISPECIES: cell division protein ZapA	K	SSSSEALKEKDEKISKLEEEIKIIKEEAQKY	_ISK(ac)LEEEIK_	3	-1.0952	114.5	13870000	13898000
>WP_013240069.1 MULTISPECIES: phenylalanine--tRNA ligase subunit beta	K	VSRKVFDKILLPEDSKLRNAIAIKNPLGEDY	_ILLPEDSK(ac)LR_	8	0.05453	70.977	3339800	5047100
>WP_013240069.1 MULTISPECIES: phenylalanine--tRNA ligase subunit beta	K	IKSGTIVVDTAKDGEKFTTLDEEERQLNQDM	_DGEK(ac)FTTLDEEER_	4	0.15457	77.078	6790300	16558000
>WP_013240072.1 MULTISPECIES: 50S ribosomal protein L20	K	AINDPKGFTELVEVAKKQLNA	_GFTELVEVAK(ac)K_	10	0.96191	115.18	31004000	20161000
>WP_013240074.1 MULTISPECIES: translation initiation factor IF-3	K	KIINKSFLINQDIVEKEVRVISDDGSQMGII	_SFLINQDIVEK(ac)EVR_	11	0.08749	92.063	4526400	4677400
>WP_013240074.1 MULTISPECIES: translation initiation factor IF-3	K	FHSKLQDVCVIERQPKLEGRNMIMFLSAKKA	_QPK(ac)LEGR_	3	0.85003	96.19	15119000	13875000

>WP_013240075.1 MULTISPECIES: threonine--tRNA ligase	K	MPERFDLTYVGADGEKHRPVMVHRVVFGSIE	_FDLTYVGADGEK(ac)HRPVMVHR_	12	-0.41272	38.017	0	2974800
>WP_013240075.1 MULTISPECIES: threonine--tRNA ligase	K	FMTKDNMKDEIVNVIKLIDSFYKVFKFEYFV	_DEIVNVIK(ac)LIDSFYK_	8	-0.94762	106.19	0	5498000
>WP_013240075.1 MULTISPECIES: threonine--tRNA ligase	K	NGKVEELNKEINEDCKLEILTFEDEDGKKIL	_EINEDCK(ac)LEILTFEDEDGKK_	7	-0.32251	89.859	7704800	8596400
>WP_013240075.1 MULTISPECIES: threonine--tRNA ligase	K	MIKIVLKDGKELEVEKGLKVAD	_IVLK(ac)DGK_	4	-1.1631	73.616	8573100	11189000
>WP_013240075.1 MULTISPECIES: threonine--tRNA ligase	K	EDCKLEILTFEDEDGKKILRHTGSHILAQAV	_LEILTFEDEDGK(ac)K_	12	0.0859	98.531	9859600	0
>WP_013240075.1 MULTISPECIES: threonine--tRNA ligase	K	RFELTRDEAVKLMEEKNEPYKVQLIKDLPED	_LMEEK(ac)NEPYK_	5	0.33554	90.108	11634000	8073000
>WP_013240075.1 MULTISPECIES: threonine--tRNA ligase	K	TEGLKDALKAVGLDYKVNEGDGAFYGPKIDF	_AVGLDYK(ac)VNEGDGAFYGPK_	7	0.10226	74.786	15335000	14179000
>WP_013240075.1 MULTISPECIES: threonine--tRNA ligase	K	MIKIVLKDGKELEVEKGLKVADVAAKLSTSL	_ELEVEK(ac)GLK_	6	0.38493	95.358	19593000	18344000
>WP_013240075.1 MULTISPECIES: threonine--tRNA ligase	K	KILRHTGSHILAQAVKRLYPEVKLAIGPSID	_HTGSHILAQAVK(ac)R_	12	-0.60045	129.82	23636000	23836000
>WP_013240075.1 MULTISPECIES: threonine--tRNA ligase	K	KKNDLKDYLTMMEEAKKRDHRKLGKELDLFS	_DYLTMMEEAK(ac)K_	10	-0.36918	89.403	25989000	33605000
>WP_013240075.1 MULTISPECIES: threonine--tRNA ligase	K	LKVADVAAKLSTSLGKKALGAKINGKVEELN	_LSTSLGK(ac)K_	7	0.46793	94.114	45129000	55167000
>WP_013240075.1 MULTISPECIES: threonine--tRNA ligase	K	LPIRYGEMGIVHRHEKSGALHGLMRVRCFTQ	_HEK(ac)SGALHGLMR_	3	-0.12364	180.18	54972000	60921000
>WP_013240075.1 MULTISPECIES: threonine--tRNA ligase	K	EKIEGEMNKIIKENEKLERFELTRDEAVKLM	_ENEK(ac)LER_	4	0.54384	108.09	85645000	52095000
>WP_013240075.1 MULTISPECIES: threonine--tRNA ligase	K	EKNKMLQRIYGTAFEKKNDLKDYLTMMEEAK	_IYGTAFEK(ac)K_	8	0.32621	120.12	115120000	82388000
>WP_013240075.1 MULTISPECIES: threonine--tRNA ligase	K	LKENDIRVESDMRNEKIGYKIREAQMQKVPY	_NEK(ac)IGYK_	3	-0.60111	94.662	653220000	644090000
>WP_013240078.1 aspartate aminotransferase	K	ERRDYVFNSLKSLGFKVNLPKGAFYIFPSIK	_SLGFK(ac)VNLPK_	5	-0.37578	82.749	18829000	15604000
>WP_013240079.1 4-hydroxy-tetrahydrodipicolinate reductase	K	VSALLYKNFDIEIIEKHHNQKVDAPSGTALL	_NFDIEIIEK(ac)HHNQK_	9	0.44719	189.3	157860000	127010000
>WP_013240080.1 MULTISPECIES: 4-hydroxy-tetrahydrodipicolinate synthase	K	KTLKELCKLDNVVAVKEASGNISQIAKMKAL	_LDNVVAVK(ac)EASGNISQIAK(ac)MK_	8	1.0787	82.36	15876000	15018000
>WP_013240080.1 MULTISPECIES: 4-hydroxy-tetrahydrodipicolinate synthase	K	RTGLNIQPKTLKELCKLDNVVAVKEASGNIS	_ELCK(ac)LDNVVAVK_	4	-0.26043	62.924	18175000	14372000
>WP_013240080.1 MULTISPECIES: 4-hydroxy-tetrahydrodipicolinate synthase	K	DMCKLFLEGNVKEALKMQLKALTLMNTMFIE	_EALK(ac)MQLK_	4	-0.82537	80.229	26747000	17488000
>WP_013240080.1 MULTISPECIES: 4-hydroxy-tetrahydrodipicolinate synthase	K	KERKDTIKFTVDTVNKRIPVIAGTGSNCTES	_FTVDTVNK(ac)R_	8	-2.3732	157.91	53897000	51407000
>WP_013240080.1 MULTISPECIES: 4-hydroxy-tetrahydrodipicolinate synthase	K	RLPLCDMSKENLDVLKSELKNYGLLK	_ENLDVLK(ac)SELK_	7	0.34028	60.301	123550000	95164000
>WP_013240080.1 MULTISPECIES: 4-hydroxy-tetrahydrodipicolinate synthase	K	VVAVKEASGNISQIAKMKALCGDDIDLYSGN	_LDNVVAVK(ac)EASGNISQIAK(ac)MK_	19	1.0787	134.98	245170000	190250000
>WP_013240081.1 aspartate-semialdehyde dehydrogenase	K	HIDVFLENGYTKEEMKMVNETRKILHDDTLR	_EEMK(ac)MVNETR_	4	0.06704	48.741	4171200	3401600
>WP_013240081.1 aspartate-semialdehyde dehydrogenase	K	FASAKSSGKILKFKDKDYTVEELKEDNIKNK	_DK(ac)DYTVEELK_	2	0.13721	47.288	5672100	4642400
>WP_013240081.1 aspartate-semialdehyde dehydrogenase	K	KGAATNAVQIAECIIKDK	_KGAATNAVQIAECIIK(ac)DK_	16	-0.07229	64.705	18855000	0
>WP_013240081.1 aspartate-semialdehyde dehydrogenase	K	KEVPLVVPEVNPEDIKWNKGIIANPNCSTIQ	_EVPLVVPEVNPEDIK(ac)WNK_	15	0.59374	58.246	22438000	13930000
>WP_013240081.1 aspartate-semialdehyde dehydrogenase	K	KILKFKDKDYTVEELKEDNIKNKKIDIALFS	_DYTVEELK(ac)EDNIK_	8	-0.01457	173.03	40463000	49420000
>WP_013240081.1 aspartate-semialdehyde dehydrogenase	K	PVTYSHSESINVELKKDFKIEDIFELYKNAT	_K(ac)DFKIEDIFELYK_	1	0.09247	78.814	46884000	2539400
>WP_013240081.1 aspartate-semialdehyde dehydrogenase	K	YSHSESINVELKKDFKIEDIFELYKNATGVV	_KDFK(ac)IEDIFELYK_	4	0.50528	172.82	62957000	91604000
>WP_013240089.1 DUF4364 domain-containing protein	K	ESNSTLMNLKLNVDSKKTAQKLCEKWEKNFS	_LNVDSK(ac)K_	6	0.16068	77.058	5416900	4541800

>WP_013240093.1 valine--tRNA ligase	K	IERLNKEKEKLEAEIKRVENKLSNEKFVSKA	_LEAEIK(ac)R_	6	0.22493	105.95	6243100	6847200
>WP_013240093.1 valine--tRNA ligase	K	ESITISKWPEYDESLKDLKAEKDMEYIIEAI	_WPEYDESLK(ac)DLK_	9	0.83493	39.73	6830400	4792700
>WP_013240093.1 valine--tRNA ligase	K	VKITPAHDPNDYHVGKRHNLPEIIIMNEDGT	_ITPAHDPNDYHVGK(ac)R_	14	-0.06041	93.823	30600000	32262000
>WP_013240093.1 valine--tRNA ligase	K	RKLVVEDLDKQGYLVKIKEHAHNVGCHDRCG	_QGYLVK(ac)IK_	6	0.45149	67.169	32440000	24282000
>WP_013240093.1 valine--tRNA ligase	K	LEAEIKRVENKLSNEKFVSKAPEAVVNAERE	_LSNEK(ac)FVSK_	5	0.12768	96.342	43217000	35737000
>WP_013240100.1 MULTISPECIES: C-terminal binding protein	K	IHAPLTPETRHIFDYKAFKQMKNTSMIINVS	_HIFDYK(ac)AFK_	6	0.30117	55.567	12370000	12146000
>WP_013240101.1 MULTISPECIES: RraA family protein	K	MSNIGCKIISDFKRPDKSLVEGFRNIPV	_IISDFK(ac)RPDK_	6	0.50154	107.24	11302000	13286000
>WP_013240103.1 MULTISPECIES: amidohydrolase	K	RFNLDYKVAKQSISNKFLPDIISTDTTKVTL	_QSISNK(ac)FLPDIISTDTTK_	6	1.0386	41.513	5912200	0
>WP_013240118.1 3-oxoacyl-[acyl-carrier-protein] reductase	K	SSEESTKEVVKSVIAKFKKIDILINNAGVTS	_SVIAK(ac)FK_	5	-0.10084	81.958	3924600	4920900
>WP_013240146.1 MULTISPECIES: carboxymuconolactone decarboxylase	K	NVLCKAGVEAINAAEKRFEEINSIKEDEK	_AGVEAINAAEK(ac)R_	11	-0.33727	53.967	1731300	0
>WP_013240148.1 MULTISPECIES: nitroreductase	K	VRAYTDEVISKETITKLIELGTKASTGSGLE	_ETITK(ac)LIELGTK_	5	-5.8766	137.16	132410000	162710000
>WP_013240166.1 MULTISPECIES: ATP-dependent helicase	K	FTFINVPERVSSIILKKTNGKKLQGRRVSVE	_VSSIILK(ac)K_	7	0.65721	60.436	82948000	52741000
>WP_013240169.1 MULTISPECIES: transcription elongation factor GreA	K	EVTLVSTVEADVKNMKISIESPLGKALYKLK	_NMK(ac)ISIESPLGK_	3	-0.35756	71.241	3201900	4729000
>WP_013240174.1 DUF1858 domain-containing protein	K	ITKNMTIAEIIKMNPKNAERLMTFGMECVTC	_MNPK(ac)NAER_	4	-0.26612	56.729	5509100	3976700
>WP_013240174.1 DUF1858 domain-containing protein	K	MSEITKNMTIAEIIKMNPKNAERLMTFGME	_NMTIAEIIK(ac)MNPK_	9	0.72561	43.382	7254200	4194900
>WP_013240177.1 MULTISPECIES: adenosylcobinamide-GDP ribazoletransferase	K	GFFAFKGNDKIIEIMKDSRIGTYACATIVMD	_IIEIMK(ac)DSR_	6	0.43731	85.377	6058600	5933700
>WP_013240179.1 transcriptional repressor	K	MDNLTSIFREKKLKLTPQRIAVYKYLQST	_LK(ac)LTPQR_	2	0.48621	104.45	25539000	25337000
>WP_013240186.1 MULTISPECIES: endopeptidase La	K	MDKNLKVLPLIPLRGITVFPY	_NLK(ac)VLPLIPLR_	3	0.31154	62.303	8439800	9236200
>WP_013240187.1 MULTISPECIES: ATP-dependent Clp protease ATP-binding subunit ClpX	K	AVDTKKPELIEAEDGKRTPIKLKKSRTRKGP	_KPELIEAEDGK(ac)R_	11	-0.24852	110.38	24745000	25611000
>WP_013240187.1 MULTISPECIES: ATP-dependent Clp protease ATP-binding subunit ClpX	K	EFKEDALEAISDEALKRNTGARGLRAIIEET	_EDALEAISDEALK(ac)R_	13	0.03205	182.02	25530000	35459000
>WP_013240188.1 MULTISPECIES: ATP-dependent Clp endopeptidase, proteolytic subunit ClpP	K	ATDIGIHAKRILDIKKKLNTIISERTGQPLE	_K(ac)KLNTIISER_	1	-0.11585	73.885	2444500	18655000
>WP_013240188.1 MULTISPECIES: ATP-dependent Clp endopeptidase, proteolytic subunit ClpP	K	TDIGIHAKRILDIKKKLNTIISERTGQPLEK	_KK(ac)LNTIISER_	2	0.16326	74.944	10510000	9061700
>WP_013240188.1 MULTISPECIES: ATP-dependent Clp endopeptidase, proteolytic subunit ClpP	K	KLNTIISERTGQPLEKVEKDTERDNFMTAEE	_TGQPLEK(ac)VEK_	7	-0.54217	71.03	11700000	10863000
>WP_013240188.1 MULTISPECIES: ATP-dependent Clp endopeptidase, proteolytic subunit ClpP	K	EEAKEYGLIDEVITKKK	_EYGLIDEVITKK(ac)K_	12	0.72628	62.14	20827000	14254000
>WP_013240188.1 MULTISPECIES: ATP-dependent Clp endopeptidase, proteolytic subunit ClpP	K	AEEAKEYGLIDEVITKKK	_EYGLIDEVITK(ac)K_	11	0.20501	116.24	281540000	200170000
>WP_013240189.1 MULTISPECIES: trigger factor	K	KNTYEVKDEEVEAELKTMQQKNARIETKESG	_DEEVEAELK(ac)TMQQK_	9	-0.04334	43.03	2952900	7231000
>WP_013240189.1 MULTISPECIES: trigger factor	K	KVCSNAEINIPEVMIKKEIDTMLKDLELRLK	_VCSNAEINIPEVMIK(ac)K_	15	0.48659	30.133	3940600	0

>WP_013240189.1 MULTISPECIES: trigger factor	K	QKSYLETDVVNEKVVKMLVDSSKATA	_VVK(ac)MLVDSSK_	3	-1.1707	83.397	5998800	11498000
>WP_013240189.1 MULTISPECIES: trigger factor	K	NIPEVMIKKEIDTMLKDLELRLKYQGLDLKT	_KEIDTMLK(ac)DLELR_	8	-0.11229	105.14	10031000	5553900
>WP_013240189.1 MULTISPECIES: trigger factor	K	RVRTDLVIGEIAKVEKIEATDDELMERAKEM	_VEK(ac)IEATDDELMER_	3	0.15878	101.62	13460000	9739900
>WP_013240189.1 MULTISPECIES: trigger factor	K	DDEFAKDISEFDTLDKVKEDIKNKKKEANEL	_DISEFDTLDK(ac)VK_	10	-0.77198	62.14	14464000	8832400
>WP_013240189.1 MULTISPECIES: trigger factor	K	KFNESMKKAFAKNAKKFNIPGFRKGKAPMNI	_K(ac)FNIPGFR_	1	0.27841	139.68	17942000	18182000
>WP_013240189.1 MULTISPECIES: trigger factor	K	KKEIDTMLKDLELRLKYQGLDLKTYYQYTNN	_LK(ac)YQGLDLK_	2	-0.14329	95.523	23769000	18091000
>WP_013240189.1 MULTISPECIES: trigger factor	K	KEFIYTAKVVVLPEVKLGEYKGLEVKKNTYE	_VVVLPEVK(ac)LGEYK_	8	0.27437	80.522	31982000	29881000
>WP_013240189.1 MULTISPECIES: trigger factor	K	KLEITVEAEKFNESMKKAFAKNAKKFNIPGF	_FNESM(ox)K(ac)K_	6	0.35758	81.119	119160000	76797000
>WP_013240194.1 MULTISPECIES: dihydroorotate dehydrogenase electron transfer subunit	K	KNELSYTTEKVYKNEKTAEGIYKLEIQGKFK	_NEK(ac)TAEGIYK_	3	-0.16886	57.348	5752000	1100400
>WP_013240194.1 MULTISPECIES: dihydroorotate dehydrogenase electron transfer subunit	K	NEKTAEGIYKLEIQGKFKGKPGQFYMLRCWK	_LEIQGK(ac)FK_	6	-0.02575	73.931	10176000	10052000
>WP_013240195.1 MULTISPECIES: orotidine-5'-phosphate decarboxylase	K	MIIDKLYDDVLKKGNVCVGLDTALEYI	_LYDDVLK(ac)K_	7	0.17694	59.8	14229000	15053000
>WP_013240196.1 MULTISPECIES: aspartate carbamoyltransferase regulatory subunit	K	RAGYGMKIFNYLELNKAKYSVALIMNAVSEK	_IFNYLELNK(ac)AK_	9	-0.01805	93.258	25029000	29024000
>WP_013240198.1 MULTISPECIES: CoA activase	K	FGRYAGAIGAALLASKLK	_YAGAIGAALLASK(ac)LK_	13	0.98478	39.509	0	9873800
>WP_013240200.1 2-hydroxyacyl-CoA dehydratase	K	LNDYVPVHVMELPQMKREKNCKAWEEEIKEF	_AWEILNDYVPVHVMELPQMK(ac)R_	20	-0.47733	64.83	0	16150000
>WP_013240200.1 2-hydroxyacyl-CoA dehydratase	K	ISQIAFYDDPDRFVEKLNELCDELDERVKKG	_FVEK(ac)LNELCDELDER_	4	-0.71634	63.037	10518000	13302000
>WP_013240224.1 MULTISPECIES: 4-amino-4-deoxychorismate lyase	K	TLFNTRKNNYTEEQYKRGFKVKISSVRRNEF	_KNNYTEEQYK(ac)R_	10	1.0133	73.885	11221000	4160100
>WP_013240232.1 MULTISPECIES: DUF3786 domain-containing protein	K	MLSYLYRATNSSLTNKWVPLRELEGVGHAYQ	_ATNSSLTNK(ac)WVPLR_	9	0.48728	77.912	9955700	10225000
>WP_013240232.1 MULTISPECIES: DUF3786 domain-containing protein	K	NMCKEFVKYDPEEVAKRSRAYYDSEKKQFTL	_YDPEEVAK(ac)R_	8	-0.41465	117.89	17648000	18092000
>WP_013240233.1 MULTISPECIES: hemerythrin	K	LEYTKYHFEYEENMLKKYNYDHIHDQEEEHG	_YHFEYEENMLK(ac)K_	11	0.1692	48.004	4065300	10045000
>WP_013240242.1 MULTISPECIES: hypothetical protein	K	IVTKNMWFNGTELTVKAQRAIRNEQITIISH	_NMWFNGTELTVK(ac)AQR_	12	-4.4696	82.102	3244900	0
>WP_013240242.1 MULTISPECIES: hypothetical protein	K	PIQNRICLNNFDEFFKKHKVDFNDEKDIISV	_ICLNNFDEFFK(ac)K_	11	2.5843	74.76	5907800	0
>WP_013240242.1 MULTISPECIES: hypothetical protein	K	NFLRQESHLQKFFQIKHEYHGGHRYIPAEKA	_FFQIK(ac)HEYHGGHR_	5	-2.0524	43.382	8376700	9677700
>WP_013240263.1 hypothetical protein	K	GKCGNKYPVYNQDKLKFTGIVQDSCMDKAAK	_LK(ac)FTGIVQDSCMDK_	2	-0.31068	66.533	8785000	7973800
>WP_013240273.1 hypothetical protein	K	SPEKYRALELFYLKEKTYEEVAERLSCGVVT	_EK(ac)TYEEVAER_	2	0.27926	46.592	6157600	3443200
>WP_013240275.1 HNH endonuclease	K	YPKIHGRHAHRVIAEKVLGRKLRKGEIVHHK	_VIAEK(ac)VLGR_	5	-0.05515	78.934	1353300	13579000
>WP_013240275.1 HNH endonuclease	K	SEDNLEVLPSQKEHAKLHQKNGRFK	_EHAK(ac)LHQK_	4	-0.15555	62.2	1643700	0
>WP_013240280.1 DUF2815 domain-containing protein	K	YSVSVIIPKTDTDTLKAIKEAVAEAKEQGKS	_TDTDTLK(ac)AIK_	7	-1.2798	101.38	20116000	15921000
>WP_013240280.1 DUF2815 domain-containing protein	K	EQGKSKWNGKIPSNLKTPLRDGDSERPDDEA	_IPSNLK(ac)TPLR_	6	-0.11092	140.16	54600000	1639300
>WP_013240284.1 hypothetical protein	K	MRKIKVALVKFKGYQEFMEYSYFTD	_VALVK(ac)FK_	5	0.6634	86.113	3548800	19131000

>WP_013240291.1 helix-turn-helix domain-containing protein	K	DKIKDLRKLKKITQQKLAKSIGLSQSSIGMI	_ITQQK(ac)LAK_	5	-0.66088	64.265	2122000	2421200
>WP_013240294.1 MULTISPECIES: XTP/dITP diphosphatase	K	DKKNNEKLLKLLEGMKPQDREAKFVCALVLI	_LLEGMK(ac)PQDR_	6	-0.10889	84.17	0	6437500
>WP_013240294.1 MULTISPECIES: XTP/dITP diphosphatase	K	GEVNGIICDEERGNNKFGYDPLFYVPEYHKT	_GNNK(ac)FGYDPLFYVPEYHK_	4	-0.90132	33.836	10184000	0
>WP_013240295.1 MULTISPECIES: ribonuclease PH	K	PFTRDDLQELLRLGEKGINNMIHAQKESLKM	_LGEK(ac)GINNMIHAQK_	4	0.06917	82.543	10299000	9303400
>WP_013240296.1 MULTISPECIES: AIR synthase	K	DYEDKLKNILTTEEIKEAKGYIKYISVVKEG	_NILTTEEIK(ac)EAK_	9	0.0672	51.875	5210900	7786600
>WP_013240296.1 MULTISPECIES: AIR synthase	K	MKAGKLNWEDLKTIIDENRKVVREDVR	_LNWEDLK(ac)TIIDENRK_	7	-0.26318	44.807	9083000	5859800
>WP_013240296.1 MULTISPECIES: AIR synthase	K	MKAGKLNWEDLKTIIDENRK	_AGK(ac)LNWEDLK_	3	0.45798	95.573	22412000	22260000
>WP_013240306.1 MULTISPECIES: NAD-dependent DNA ligase LigA	K	NLAYRTELGSTSKVPKWAIAYKYPPEEKETK	_VPK(ac)WAIAYK_	3	-0.48127	57.598	2538300	7630200
>WP_013240307.1 MULTISPECIES: DNA helicase PcrA	K	LNISDKDITDREIINKISGQKDNLISPHQFK	_EIINK(ac)ISGQK_	5	0.87694	57.348	0	2996900
>WP_013240307.1 MULTISPECIES: DNA helicase PcrA	K	LKLENIKEGTKVKHGKFGLGTIVSISKSGSD	_HGK(ac)FGLGTIVSISK_	3	-1.2715	67.115	2975200	7774100
>WP_013240308.1 MULTISPECIES: hypothetical protein	K	TINEIKALEQRLQVAKMLKNKKTYLDIAAAT	_LQVAK(ac)MLK_	5	-0.32624	69.198	8821600	9615100
>WP_013240321.1 glutamine-hydrolyzing GMP synthase	K	AKTNGCPVAAIENTDKKVYGVQFHPEVEHTP	_TNGCPVAAIENTDK(ac)K_	14	0.88259	61.097	4041300	1861100
>WP_013240321.1 glutamine-hydrolyzing GMP synthase	K	KDRFLGKLEGVSDPEKKRKIIGEEFIRVFEE	_LEGVSDPEK(ac)K_	9	-0.39891	118.4	54283000	27505000
>WP_013240321.1 glutamine-hydrolyzing GMP synthase	K	GLLRKDEGDQVEHVFKKQFDMNFIRVNAKDR	_KDEGDQVEHVFK(ac)K_	12	0.78549	133.16	71622000	30123000
>WP_013240321.1 glutamine-hydrolyzing GMP synthase	K	KSHHNVGGLPEDMDFKLIEPLRELFKDEVRA	_SHHNVGGLPEDM(ox)DFK(ac)LIEPLR_	15	-0.01851	141.08	200650000	188520000
>WP_013240321.1 glutamine-hydrolyzing GMP synthase	K	EDMDFKLIEPLRELFKDEVRAVGEELGIPHK	_ELFK(ac)DEVR_	4	-0.10551	126.52	233180000	257080000
>WP_013240322.1 IMP dehydrogenase	K	VDTAHGHSKGVLDAVKTIKEKYPNVQIIAGN	_GVLDAVK(ac)TIK_	7	-0.38506	59.542	2562100	2012600
>WP_013240322.1 IMP dehydrogenase	K	ETNYDKKISEVMTREKLITAPEDTTIEEAKE	_EK(ac)LITAPEDTTIEEAK_	2	0.24916	37.092	5126400	6325300
>WP_013240322.1 IMP dehydrogenase	K	PVIADGGIKYSGDIVKALAAGAKVVMMGSMF	_YSGDIVK(ac)ALAAGAK_	7	-0.62374	66.533	7555500	8401800
>WP_013240322.1 IMP dehydrogenase	K	EILKTSKIEKLPLVDKDNNLRGLITIKDIEK	_LPLVDK(ac)DNNLR_	6	0.3207	67.153	9965000	10024000
>WP_013240322.1 IMP dehydrogenase	K	LPREVSLATNLTKTIKLNIPLMSAGMDTVTD	_TIK(ac)LNIPLMSAGMDTVTDSK_	3	1.4269	149.94	206680000	175260000
>WP_013240323.1 chaperonin GroEL	K	AIPEVEKLTSDVPDIKVGIDIIRKALEEPIR	_LTSDVPDIK(ac)VGIDIIR_	9	-0.68785	52.823	3887800	15598000
>WP_013240323.1 chaperonin GroEL	K	LFGEDARKSMQEGVNKLANAVKVTLGPKGRN	_SMQEGVNK(ac)LANAVK_	8	0.17204	53.754	4560400	13649000
>WP_013240323.1 chaperonin GroEL	K	VDKAVEEIKKISTTVKGKEDIARIAAISASD	_ISTTVK(ac)GK_	6	0.09514	63.419	10473000	12899000
>WP_013240323.1 chaperonin GroEL	K	KMEAAIEDPYILITDKKISNIQDILPLLEKI	_MEAAIEDPYILITDK(ac)K_	15	-0.78721	68.919	15496000	0
>WP_013240323.1 chaperonin GroEL	K	IELEDPYENMGAQLVKEVATKTNDVAGDGTT	_EIELEDPYENMGAQLVK(ac)EVATK_	17	-0.42559	66.644	17962000	9509300
>WP_013240323.1 chaperonin GroEL	K	LVVNKLRGTFTCVAVKAPGFGDRRKEMLQDI	_GTFTCVAVK(ac)APGFGDRR_	9	0.70274	128.06	54790000	25679000
>WP_013240323.1 chaperonin GroEL	K	IQDILPLLEKIVQQGKKLLIIAEDVEGEALA	_IVQQGK(ac)K_	6	0.18089	76.847	67220000	10382000
>WP_013240323.1 chaperonin GroEL	K	IKVQIEETTSDFDKEKLQERLAKLAGGVAVV	_VQIEETTSDFDKEK(ac)LQER_	14	0.21822	208.26	80741000	68953000
>WP_013240323.1 chaperonin GroEL	K	TTSDFDKEKLQERLAKLAGGVAVVKVGAATE	_LAK(ac)LAGGVAVVK_	3	0.33325	83.182	88719000	34175000
>WP_013240323.1 chaperonin GroEL	K	ANPMLIRQGIKIAVDKAVEEIKKISTTVKGK	_IAVDK(ac)AVEEIK(ac)K_	5	-0.57297	105.13	91263000	71923000

>WP_013240323.1 chaperonin GroEL	K	QISSNAGVEGSVIIEKVKNSEIGVGYDALKG	_QISSNAGVEGSVIIEK(ac)VK_	16	1.217	100.35	101340000	107400000
>WP_013240323.1 chaperonin GroEL	K	GVAVVKVGAATETELKEKKLRIEDALAATKA	_VGAATETELK(ac)EK_	10	1.3266	149.38	104130000	74788000
>WP_013240323.1 chaperonin GroEL	K	LQERLAKLAGGVAVVKVGAATETELKEKKLR	_LAGGVAVVK(ac)VGAATETELK_	9	-0.42249	82.482	121170000	245620000
>WP_013240323.1 chaperonin GroEL	K	DKKISNIQDILPLLEKIVQQGKKLLIIAEDV	_KISNIQDILPLLEK(ac)IVQQGK_	14	-5.2991	178.52	164620000	585820000
>WP_013240323.1 chaperonin GroEL	K	VKVTLGPKGRNVVLDKKFGSPLITNDGVTIA	_NVVLDK(ac)K_	6	-0.89521	79.116	173750000	115810000
>WP_013240323.1 chaperonin GroEL	K	KVTLGPKGRNVVLDKKFGSPLITNDGVTIAK	_K(ac)FGSPLITNDGVTIAK_	1	0.38951	125.52	212150000	155280000
>WP_013240323.1 chaperonin GroEL	K	IARIAAISASDEEIGKLIADAMEKVGNEGVI	_IAAISASDEEIGK(ac)LIADAMEK_	13	-3.1883	162.09	427190000	19743000
>WP_013240323.1 chaperonin GroEL	K	RQGIKIAVDKAVEEIKKISTTVKGKEDIARI	_IAVDK(ac)AVEEIK(ac)K_	11	-0.57297	103.91	708970000	507170000
>WP_013240323.1 chaperonin GroEL	K	VGYDALKGEYVNMVEKGIVDPTKVTRSALQN	_GEYVNMVEK(ac)GIVDPTK_	9	-0.65001	132.59	734400000	604330000
>WP_013240324.1 MULTISPECIES: co-chaperone GroES	K	GIGGTVDGKEVKMEVKVGDKVLFSKYAGNEV	_MEVK(ac)VGDK_	4	0.07039	67.563	6154100	0
>WP_013240324.1 MULTISPECIES: co-chaperone GroES	K	VVAVGIGGTVDGKEVKMEVKVGDKVLFSKYA	_EVK(ac)MEVK_	3	-0.47844	84.498	18022000	15917000
>WP_013240324.1 MULTISPECIES: co-chaperone GroES	K	ETTKSGIVLPGSAKEKPQEAEVVAVGIGGTV	_EK(ac)PQEAEVVAVGIGGTVDGK_	2	1.634	85.231	41077000	0
>WP_013240327.1 MULTISPECIES: DNA-3-methyladenine glycosylase 2 family protein	K	NRIPMIQRAIKNISEKWGEPLEYKNKIYYSF	_NISEK(ac)WGEPLEYK_	5	-0.77513	51.211	5309700	2755800
>WP_013240329.1 MULTISPECIES: redox-sensing transcriptional repressor Rex	K	CVPNNNAQKVCDILVKNKVKGIWNFAPVDLV	_VCDILVK(ac)NK_	7	0.00381	67.563	4125400	3064800
>WP_013240329.1 MULTISPECIES: redox-sensing transcriptional repressor Rex	K	MDKKKNISMAVIKRLPKYHRYLMDLMKN	_NISMAVIK(ac)R_	8	0.18032	85.67	29300000	27836000
>WP_013240329.1 MULTISPECIES: redox-sensing transcriptional repressor Rex	K	AGNIGQAIANYTRFEKIGFRLEAIFDINPKL	_FEK(ac)IGFR_	3	0.2404	124.37	29711000	26402000
>WP_013240329.1 MULTISPECIES: redox-sensing transcriptional repressor Rex	K	DKKKNISMAVIKRLPKYHRYLMDLMKNDVDR	_RLPK(ac)YHR_	4	0.32002	154.11	42113000	57642000
>WP_013240334.1 3-hydroxybutyryl-CoA dehydrogenase	K	SNTSGLSPTAIAKNTKYPERVAIAHFWNPPQ	_NTK(ac)YPER_	3	0.4406	101.6	42387000	39818000
>WP_013240335.1 MULTISPECIES: thiamine-binding protein	K	TTMEGELSELLKIVEKAQSICTEAGAERVAS	_IVEK(ac)AQSICTEAGAER_	4	-0.4418	115.5	20848000	12039000
>WP_013240336.1 DNA-binding response regulator	K	LNEIVSKIDKESNLVKRIPCERQGKIILVNV	_IDKESNLVK(ac)R_	9	0.92904	107.83	3251300	10477000
>WP_013240340.1 peptidase M16	K	ILKNNHKSMVIVKPEKGLEEKKEKALKEKLG	_SMVIVKPEK(ac)GLEEK_	9	0.05962	41.644	0	3417500
>WP_013240340.1 peptidase M16	K	ESEKDKFKQVVETTLKDMVNKGIDKKLVEAS	_QVVETTLK(ac)DMVNK_	8	0.48975	77.912	4362500	2972000
>WP_013240340.1 peptidase M16	K	AQSFGENGNSHKFYPKFVVRSKVLVDNLPKL	_FYPK(ac)FVVR_	4	0.09882	65.252	12309000	10947000
>WP_013240340.1 peptidase M16	K	ILNQAAFSSERQMTIKYPISSSEREEDKTFL	_QMTIK(ac)YPISSSER_	5	1.1197	141.38	18327000	19757000
>WP_013240340.1 peptidase M16	K	KLSDSELEKIIQDTQKLKKRQESGDSKEDLM	_IIQDTQK(ac)LK_	7	-0.25694	110.87	19699000	18922000
>WP_013240340.1 peptidase M16	K	LPKLISILNDVINHTKFEEKKRLREIIQETK	_LISILNDVINHTK(ac)FEEK_	13	-0.23485	111.22	23272000	32322000
>WP_013240340.1 peptidase M16	K	SVMNKNNVCVLGNEQKIKENKDIFNNMVSLF	_NNVCVLGNEQK(ac)IK_	11	1.0491	156.36	60850000	47202000
>WP_013240342.1 pyrroline-5-carboxylate reductase	K	SLEKNNFRGTVIEAVKKCTEKSIEMSKRV	_GTVIEAVK(ac)K_	8	-0.03439	77.677	19098000	16579000

>WP_013240343.1 MULTISPECIES: pyruvate carboxylase	K	IHPGYGFLSENPELAKKCKEAGIEFIGPTSD	_NVDAIHPGYGFLSENPELAK(ac)K_	20	-0.15937	49.973	0	2046300
>WP_013240343.1 MULTISPECIES: pyruvate carboxylase	K	DDEGYRFLVFEVNGIKRDIRIKDNLAFSGSG	_FLVFEVNGIK(ac)R_	10	1.4204	87.298	3622200	12096000
>WP_013240343.1 MULTISPECIES: pyruvate carboxylase	K	KNTNRDTKLDADNLQKISNYWEDVRPIYSQF	_LDADNLQK(ac)ISNYWEDVRPIYSQFESGLK_	8	3.1423	192.18	4287800	8812800
>WP_013240343.1 MULTISPECIES: pyruvate carboxylase	K	KYTLQYYINLAKEIEKTGAQILGIKDMSALL	_EIEK(ac)TGAQILGIK_	4	-0.28304	45.28	6217400	0
>WP_013240343.1 MULTISPECIES: pyruvate carboxylase	K	FIGNKVVNETRGKKKKFNIPIVPKVEEDIKL	_K(ac)FNIPIVPK_	1	-0.27058	68.735	8429900	0
>WP_013240343.1 MULTISPECIES: pyruvate carboxylase	K	ALSEEKRQQICEDALKIARTVGYTSAGTLEF	_RQQICEDALK(ac)IAR_	10	-0.67273	67.456	9993900	1762500
>WP_013240343.1 MULTISPECIES: pyruvate carboxylase	K	VKTNADFIIKVLSHEKFIKGECDTNFIEDNP	_VLSHEK(ac)FIK_	6	0.37512	102.06	14963000	12596000
>WP_013240343.1 MULTISPECIES: pyruvate carboxylase	K	QKVIEFTPSLALSEEKRQQICEDALKIARTV	_VIEFTPSLALSEEK(ac)R_	14	1.3925	82.102	15739000	12061000
>WP_013240343.1 MULTISPECIES: pyruvate carboxylase	K	IPGVQEAIKTEEEALKFAKFCGYPVMIKAAD	_TEEEALK(ac)FAK_	7	-0.3825	42.395	18276000	0
>WP_013240343.1 MULTISPECIES: pyruvate carboxylase	K	IEDNPDLFDIKPKLDKEMSVLKFIGNKVVNE	_LDK(ac)EMSVLK_	3	0.16734	107.79	22632000	19941000
>WP_013240343.1 MULTISPECIES: pyruvate carboxylase	K	YNSAKNESRKAFGSEKIYIEKYIESPKHIEV	_AFGSEK(ac)IYIEK_	6	0.86687	115.12	30191000	22102000
>WP_013240343.1 MULTISPECIES: pyruvate carboxylase	K	MEMLGDKIKSKIVAQKAGVPTIPGVQEAIKT	_IVAQK(ac)AGVPTIPGVQEAIK_	5	0.78363	106.67	66457000	67462000
>WP_013240343.1 MULTISPECIES: pyruvate carboxylase	K	FKVRPGELLPPEDFAKIKEYLTKKYKREFNN	_VRPGELLPPEDFAK(ac)IK_	14	-0.12312	126.63	134850000	99158000
>WP_013240344.1 tRNA (adenosine(37)-N6)-threonylcarbamoyltransferase complex transferase subunit TsaD	K	PKIDKLAKEGNPDAIKFPRANFHDESSLDFS	_EGNPDAIK(ac)FPR_	8	-0.1118	58.676	4490400	6195700
>WP_013240354.1 MULTISPECIES: DUF3786 domain-containing protein	K	RCLLRLSKTFGNNINKFKEVFESLGAQEVSM	_TFGNNINK(ac)FK_	8	-0.17912	107.15	162810000	142930000
>WP_013240356.1 MULTISPECIES: RNA-binding protein	K	EIIEGMLKEVRLLEHKIVIEKAM	_LLEHK(ac)IVIEK_	5	0.74611	72.006	13196000	11929000
>WP_013240357.1 DUF4445 domain-containing protein	K	KREYEIFERAKNQILKCGMEFKTYVRKDYLE	_NQILK(ac)CGMEFK_	5	0.05264	98.353	0	11876000
>WP_013240357.1 DUF4445 domain-containing protein	K	PILRKIPQLLRESDFKVTITHIPRGVGKTTI	_ESDFK(ac)VTITHIPR_	5	0.56999	79.633	21839000	29646000
>WP_013240357.1 DUF4445 domain-containing protein	K	GEISCGMRASGGAIEKVKIGSDYEPIISVIG	_ASGGAIEK(ac)VK_	8	-0.52061	95.483	44343000	1451100
>WP_013240357.1 DUF4445 domain-containing protein	K	ASSSMHGMKIEDFSSKREYEIFERAKNQILK	_IEDFSSK(ac)R_	7	-0.57829	182.39	68158000	68833000
>WP_013240357.1 DUF4445 domain-containing protein	K	TAGNVISEDSRHITEKEKKEGYVLACQCEVK	_HITEK(ac)EK_	5	-0.02006	131.11	155020000	167670000
>WP_013240359.1 CO dehydrogenase/CO-methylating acetyl-CoA synthase complex subunit beta	K	RIVWMPKELKDFVAEKLNKTAKELYDIDNFA	_DFVAEK(ac)LNK_	6	0.46432	85.731	0	242920000
>WP_013240359.1 CO dehydrogenase/CO-methylating acetyl-CoA synthase complex subunit beta	K	TIDKAPGRMPLGMLIKVSGTNMQKDFEPVLE	_MPLGMLIK(ac)VSGTNMQK_	8	0.09917	53.403	5712700	13160000
>WP_013240359.1 CO dehydrogenase/CO-methylating acetyl-CoA synthase complex subunit beta	K	EDVVKFLEEKGHPALKMDPIM	_GHPALK(ac)MDPIM_	6	-0.78471	95.573	69849000	52307000
>WP_013240359.1 CO dehydrogenase/CO-methylating acetyl-CoA synthase complex subunit beta	K	VPKEGVVDENLGIWEKVNETVSKISQGAVTS	_EGVVDENLGIWEK(ac)VNETVSK_	13	0.81344	140.32	86366000	72910000
>WP_013240359.1 CO dehydrogenase/CO-methylating acetyl-CoA synthase complex subunit beta	K	DHKIEVIGPDIDTIDKAPGRMPLGMLIKVSG	_IEVIGPDIDTIDK(ac)APGR_	13	-1.5335	114.59	95167000	81085000
>WP_013240359.1 CO dehydrogenase/CO-methylating acetyl-CoA synthase complex subunit beta	K	ALAAAEGIVKQAVDEKGKDYKVAFPDTAYSL	_QAVDEK(ac)GK_	6	0.53509	88.294	96574000	40581000

>WP_013240359.1 CO dehydrogenase/CO-methylating acetyl-CoA synthase complex subunit beta	K	FTGSKQALAAAEGIVKQAVDEKGKDYKVAFP	_QALAAAEGIVK(ac)QAVDEK_	11	-0.94416	131.48	137050000	111480000
>WP_013240359.1 CO dehydrogenase/CO-methylating acetyl-CoA synthase complex subunit beta	K	QRNLTWVRIGKEAFEKGFRLKHFGEVIYAKM	_IGK(ac)EAFEK(ac)GFR_	8	0.37755	119.17	467790000	424770000
>WP_013240359.1 CO dehydrogenase/CO-methylating acetyl-CoA synthase complex subunit beta	K	TIATESEDVVKFLEEKGHPALKMDPIM	_FLEEK(ac)GHPALK_	5	0.57274	110.84	1.237E+09	1.151E+09
>WP_013240360.1 MULTISPECIES: carbon monoxide dehydrogenase	K	LLFDPLFLVIKGMQDKQKEVLEAIKLISDMG	_GMQDK(ac)QK_	5	-0.79207	74.191	5880000	10251000
>WP_013240360.1 MULTISPECIES: carbon monoxide dehydrogenase	K	SIRKAIEERNPEPIFKRAKEQLDAGANYLDF	_NPEPIFK(ac)R_	7	-0.29878	124.08	8.035E+09	7.704E+09
>WP_013240361.1 acetyl-CoA synthase subunit gamma	K	LAEATAPIMKTITIGKGDNEYKLGGETVLFR	_TITIGK(ac)GDNEYK_	6	-0.78815	38.021	2083000	2149700
>WP_013240361.1 acetyl-CoA synthase subunit gamma	K	IQKLGYKNLVLDPGGKSIKEAFENTVQIRRI	_NLVLDPGGK(ac)SIK_	9	-0.00833	45.257	2928800	3628600
>WP_013240361.1 acetyl-CoA synthase subunit gamma	K	KDENPLVYGANKDNFKEMVELVKGDKLALGV	_DNFK(ac)EMVELVK_	4	-0.65561	73.848	19101000	15929000
>WP_013240361.1 acetyl-CoA synthase subunit gamma	K	NEYKLGGETVLFRHEKTFVNRNRFAVAFSDS	_HEK(ac)TFVNR_	3	-0.93824	106.6	52032000	39129000
>WP_013240361.1 acetyl-CoA synthase subunit gamma	K	GISEKTKNRTLLIPGKVAVLKGELEELLPDW	_TLLIPGK(ac)VAVLK_	7	0.12558	75.819	77444000	147150000
>WP_013240361.1 acetyl-CoA synthase subunit gamma	K	VEIGKCPHMSDEAMEKLAEATAPIMKTITIG	_CPHMSDEAM(ox)EK(ac)LAEATAPIMK_	11	0.60769	74.953	114360000	42104000
>WP_013240361.1 acetyl-CoA synthase subunit gamma	K	PIMKTITIGKGDNEYKLGGETVLFRHEKTFV	_GDNEYK(ac)LGGETVLFR_	6	-0.07304	139.58	149120000	107260000
>WP_013240361.1 acetyl-CoA synthase subunit gamma	K	GAAIADEIKKCGISEKTKNRTLLIPGKVAVL	_KCGISEK(ac)TK_	7	0.53858	137.98	158480000	93321000
>WP_013240361.1 acetyl-CoA synthase subunit gamma	K	IKYAGNKDKYLALINKIKASGVKVAYALVCE	_YLALINK(ac)IK_	7	-1.2404	105.57	217730000	221920000
>WP_013240361.1 acetyl-CoA synthase subunit gamma	K	WAAGKFTGAAIADEIKKCGISEKTKNRTLLI	_FTGAAIADEIK(ac)K_	11	-0.04092	134.99	277610000	243160000
>WP_013240361.1 acetyl-CoA synthase subunit gamma	K	NKDNFKEMVELVKGDKLALGVKADGLEALYG	_GDK(ac)LALGVK_	3	-0.68667	84.615	501270000	395860000
>WP_013240361.1 acetyl-CoA synthase subunit gamma	K	RTFGYPSIIFLDELTKADKFMEVALSTLFTL	_TFGYPSIIFLDELTK(ac)ADK_	15	-1.083	135.27	525130000	650940000
>WP_013240361.1 acetyl-CoA synthase subunit gamma	K	DEAMEKLAEATAPIMKTITIGKGDNEYKLGG	_LAEATAPIMK(ac)TITIGK_	10	1.7318	104.86	1.386E+09	1.44E+09
>WP_013240362.1 MULTISPECIES: acetyl-CoA synthase subunit delta	K	KFSGKIGEVEIGTGEKALKLGGESVLPFYTF	_IGEVEIGTGEK(ac)ALK_	11	0.96855	124.3	2948700	4614300
>WP_013240362.1 MULTISPECIES: acetyl-CoA synthase subunit delta	K	EDWIDPLKDIYKDVAKDPVKWAQFVEEKYSP	_DIYK(ac)DVAK(ac)DPVK_	8	1.169	71.241	11158000	7949600
>WP_013240362.1 MULTISPECIES: acetyl-CoA synthase subunit delta	K	AIKTPLVVAGTGNHEKDAKLFEKVAQETEGH	_TPLVVAGTGNHEK(ac)DAK_	13	0.24222	193.47	29620000	26289000
>WP_013240362.1 MULTISPECIES: acetyl-CoA synthase subunit delta	K	GKIGEVEIGTGEKALKLGGESVLPFYTFDGD	_ALK(ac)LGGESVLPFYTFDGDTGNTPK_	3	0.11091	89.721	38441000	20131000
>WP_013240362.1 MULTISPECIES: acetyl-CoA synthase subunit delta	K	TLQVPIISPVSFEACKVKEAMDSEEDSPQWG	_TLQVPIISPVSFEACK(ac)VK_	16	0.45868	89.982	49827000	28371000
>WP_013240362.1 MULTISPECIES: acetyl-CoA synthase subunit delta	K	VAGTGNHEKDAKLFEKVAQETEGHNILLMSA	_LFEK(ac)VAQETEGHNILLMSAVEDNYK_	4	-0.0759	65.833	57422000	31932000
>WP_013240362.1 MULTISPECIES: acetyl-CoA synthase subunit delta	K	DVYPEDWIDPLKDIYKDVAKDPVKWAQFVEE	_DIYK(ac)DVAK(ac)DPVK_	4	1.169	107.99	97257000	88694000
>WP_013240362.1 MULTISPECIES: acetyl-CoA synthase subunit delta	K	DVAKDPVKWAQFVEEKYSPDFICLRLISADP	_WAQFVEEK(ac)YSPDFICLR_	8	-0.45828	137.19	154080000	126450000
>WP_013240362.1 MULTISPECIES: acetyl-CoA synthase subunit delta	K	PEDCAKTAKAVVEAIKTPLVVAGTGNHEKDA	_AVVEAIK(ac)TPLVVAGTGNHEK_	7	0.53692	176.42	225140000	168320000
>WP_013240362.1 MULTISPECIES: acetyl-CoA synthase subunit delta	K	MFKKPTQKFSGKIGEVEIGTGEK	_KPTQK(ac)FSGK_	5	-0.05012	141.95	2.109E+09	1.519E+09
>WP_013240363.1 carbon monoxide dehydrogenase	K	PEDSSCRKALNEILSKIQFEN	_KALNEILSK(ac)IQFEN_	9	1.4897	77.192	0	55749000

>WP_013240363.1 carbon monoxide dehydrogenase	K	RAPEGKLNSGIKEEIKKQELDLVGVVPMDQM	_LNSGIKEEIK(ac)K_	10	0.25959	82.417	6438400	6582000
>WP_013240363.1 carbon monoxide dehydrogenase	K	TGKTTLTGLLIDYLIKKGSGPILAVDADANA	_TTLTGLLIDYLIK(ac)K_	13	-0.85179	120.53	74331000	8719300
>WP_013240364.1 dihydrolipoyl dehydrogenase	K	PTKALLASTDVLSVIKGASKFGINVEGEAKP	_ALLASTDVLSVIK(ac)GASK_	13	0.23335	73.464	0	4459400
>WP_013240364.1 dihydrolipoyl dehydrogenase	K	KEKDAEKAGIQYKVGKFDFRGLGKAQAMGKL	_VGK(ac)FDFR_	3	-0.1737	79.469	6342100	9381100
>WP_013240364.1 dihydrolipoyl dehydrogenase	K	YMFEHRGVKLIRGTGKLISNKEVEVTKQDGS	_GTGK(ac)LISNK_	4	0.23114	67.881	16387000	901310
>WP_013240364.1 dihydrolipoyl dehydrogenase	K	GKFDFRGLGKAQAMGKLQGFVKIITDEKDVI	_AQAMGK(ac)LQGFVK_	6	-0.11142	78.653	16657000	13164000
>WP_013240364.1 dihydrolipoyl dehydrogenase	K	RAYAEGLGLDDIGIEKDQKGRIIVNEYLETN	_RAYAEGLGLDDIGIEK(ac)DQK_	16	1.2876	72.819	98660000	48345000
>WP_013240364.1 dihydrolipoyl dehydrogenase	K	AEREGIVAVQNAVLDKKKKMSYKAVPGCTFV	_EGIVAVQNAVLDK(ac)K_	13	0.56412	169.04	113270000	103680000
>WP_013240364.1 dihydrolipoyl dehydrogenase	K	EPEIASVGMKEKDAEKAGIQYKVGKFDFRGL	_EKDAEK(ac)AGIQYK_	6	0.75724	190.46	143790000	108710000
>WP_013240364.1 dihydrolipoyl dehydrogenase	K	DGKKVITSDEVLNLEKLPKSMILVGGGPIGC	_VITSDEVLNLEK(ac)LPK_	12	0.33095	169.28	210830000	278740000
>WP_013240364.1 dihydrolipoyl dehydrogenase	K	EALPHLAPLEDEDVAKQLQRIFKQHKIKYFV	_VVEALPHLAPLEDEDVAK(ac)QLQR_	18	0.23335	206.7	441470000	409390000
>WP_013240364.1 dihydrolipoyl dehydrogenase	K	INVEGEAKPDFDAIMKRKDKVVDQLVKGIEY	_PDFDAIM(ox)K(ac)R_	8	0.44653	239.18	1.247E+09	1.123E+09
>WP_013240365.1 5,10-methylenetetrahydrofolate reductase	K	AQGVDAKVMVGIIPLKSAGMAKYMNKNVPGI	_VMVGIIPLK(ac)SAGMAK_	9	0.29082	59.607	0	4850600
>WP_013240365.1 5,10-methylenetetrahydrofolate reductase	K	APPKGTDLSHLIECAKKIKGRVQGVNVTDFQ	_GTDLSHLIECAK(ac)K_	12	-0.3737	187.11	233220000	190900000
>WP_013240366.1 MULTISPECIES: hypothetical protein	K	LLKTRKDLKSLKAELKEADAVVSLACGDGTQ	_SLK(ac)AELK(ac)EADAVVSLACGDGTQTVAK_	7	-0.58332	84.933	13191000	17092000
>WP_013240366.1 MULTISPECIES: hypothetical protein	K	IISEKKSFDELLDYLKDSEKVIITGCSLCAT	_SFDELLDYLK(ac)DSEK_	10	0.89092	59.607	19947000	5990200
>WP_013240366.1 MULTISPECIES: hypothetical protein	K	PSCNLLKTRKDLKSLKAELKEADAVVSLACG	_SLK(ac)AELK(ac)EADAVVSLACGDGTQTVAK_	3	-0.58332	121.9	32363000	32104000
>WP_013240366.1 MULTISPECIES: hypothetical protein	K	GYKILDPSCNLLKTRKDLKSLKAELKEADAV	_K(ac)DLK(ac)SLK_	1	0.41003	163.9	64431000	13570000
>WP_013240366.1 MULTISPECIES: hypothetical protein	K	SLACGDGTQTVAKLVKIPVYPGNNTMFIGEV	_LVK(ac)IPVYPGNNTMFIGEVER_	3	-0.40568	51.566	65190000	38231000
>WP_013240366.1 MULTISPECIES: hypothetical protein	K	ATTCKVGGEEEVLAMKAKLEEQGKKVLGYKI	_VGGEEEVLAMK(ac)AK_	11	-0.12651	155.02	88297000	51243000
>WP_013240366.1 MULTISPECIES: hypothetical protein	K	KVLGYKILDPSCNLLKTRKDLKSLKAELKEA	_ILDPSCNLLK(ac)TR_	10	0.22242	195.25	138160000	88392000
>WP_013240366.1 MULTISPECIES: hypothetical protein	K	ILDPSCNLLKTRKDLKSLKAELKEADAVVSL	_K(ac)DLK(ac)SLK_	4	0.41003	163.9	869490000	861890000
>WP_013240367.1 bifunctional 5,10-methylene-tetrahydrofolate dehydrogenase/5,10-methylene-tetrahydrofolate cyclohydrolase	K	GANGSDLAYEKGALKKCEKIGIEAVVKELPA	_K(ac)CEKIGIEAVVK_	1	0.49348	65.043	4105100	15289000
>WP_013240367.1 bifunctional 5,10-methylene-tetrahydrofolate dehydrogenase/5,10-methylene-tetrahydrofolate cyclohydrolase	K	GSDLAYEKGALKKCEKIGIEAVVKELPADIS	_KCEK(ac)IGIEAVVK_	4	0.22647	121.45	14191000	24983000
>WP_013240367.1 bifunctional 5,10-methylene-tetrahydrofolate dehydrogenase/5,10-methylene-tetrahydrofolate cyclohydrolase	K	VGANGSDLAYEKGALKKCEKIGIEAVVKELP	_VGANGSDLAYEK(ac)GALK(ac)K_	16	0.10803	78.285	14834000	14536000
>WP_013240367.1 bifunctional 5,10-methylene-tetrahydrofolate dehydrogenase/5,10-methylene-tetrahydrofolate cyclohydrolase	K	PSAVIEILKHYKVPMKGKNAVIVGRSMVVGK	_HYK(ac)VPMK(ac)GK_	7	0.33407	118.26	35081000	27692000

>WP_013240367.1 bifunctional 5,10-methylene-tetrahydrofolate dehydrogenase/5,10-methylene-tetrahydrofolate cyclohydrolase	K	GVGSVTSSILAQHIVKACKLQNNL	_ASMITPVPGGVGSVTSSILAQHIVK(ac)ACK_	25	0.9558	32.985	36938000	11590000
>WP_013240367.1 bifunctional 5,10-methylene-tetrahydrofolate dehydrogenase/5,10-methylene-tetrahydrofolate cyclohydrolase	K	ADAISEALTKEVNDLKVKGITPKLTLVRVGA	_EVNDLK(ac)VK_	6	-0.33409	63.419	59731000	49182000
>WP_013240367.1 bifunctional 5,10-methylene-tetrahydrofolate dehydrogenase/5,10-methylene-tetrahydrofolate cyclohydrolase	K	TLVRVGANGSDLAYEKGALKKCEKIGIEAVV	_VGANGSDLAYEK(ac)GALK(ac)K_	12	0.10803	104.41	71470000	63735000
>WP_013240367.1 bifunctional 5,10-methylene-tetrahydrofolate dehydrogenase/5,10-methylene-tetrahydrofolate cyclohydrolase	K	IEAVVKELPADISQDKFIEELKKINADKTVN	_ELPADISQDK(ac)FIEELK_	10	-0.02568	123.02	89074000	60205000
>WP_013240367.1 bifunctional 5,10-methylene-tetrahydrofolate dehydrogenase/5,10-methylene-tetrahydrofolate cyclohydrolase	K	LTKEVNDLKVKGITPKLTLVRVGANGSDLAY	_GITPK(ac)LTLVR_	5	0.12006	114.4	186460000	160780000
>WP_013240367.1 bifunctional 5,10-methylene-tetrahydrofolate dehydrogenase/5,10-methylene-tetrahydrofolate cyclohydrolase	K	APCTPSAVIEILKHYKVPMKGKNAVIVGRSM	_HYK(ac)VPMK(ac)GK_	3	0.33407	119.89	206570000	149780000
>WP_013240367.1 bifunctional 5,10-methylene-tetrahydrofolate dehydrogenase/5,10-methylene-tetrahydrofolate cyclohydrolase	K	ELPADISQDKFIEELKKINADKTVNAIMVFR	_FIEELK(ac)K_	6	-0.34921	80.18	818070000	616900000
>WP_013240368.1 MULTISPECIES: sugar ABC transporter substrate-binding protein	K	KDTEEQKKIKEETLEKCLKQACSVPVSIVKQ	_IKEETLEK(ac)CLK_	8	0.68351	98.105	13531000	11828000
>WP_013240368.1 MULTISPECIES: sugar ABC transporter substrate-binding protein	K	CSVPVSIVKQAYEAIKLHEALVDNCSKLAIS	_QAYEAIK(ac)LHEALVDNCSK_	7	0.04376	76.768	14338000	0
>WP_013240368.1 MULTISPECIES: sugar ABC transporter substrate-binding protein	K	PLVEEGIKIADKVYEKVVSALSK	_VYEK(ac)VVSALSK_	4	0.37876	90.434	17592000	16814000
>WP_013240368.1 MULTISPECIES: sugar ABC transporter substrate-binding protein	K	PLSKAYGMPKDTEEQKKIKEETLEKCLKQAC	_DTEEQK(ac)K_	6	-0.10235	105.12	33745000	7658700
>WP_013240368.1 MULTISPECIES: sugar ABC transporter substrate-binding protein	K	MKLADKSCTDFIEVLASKAAT	_LADK(ac)SCTDFIEVLASK_	4	-0.29251	64.507	39110000	24130000
>WP_013240368.1 MULTISPECIES: sugar ABC transporter substrate-binding protein	K	MALGGMVCNLTIGKKKYAQYDEKVKGILKRS	_KK(ac)YAQYDEK_	2	0.00852	156.48	94915000	91401000
>WP_013240368.1 MULTISPECIES: sugar ABC transporter substrate-binding protein	K	KKVKAETEPLVEEGIKIADKVYEKVVSALSK	_AETEPLVEEGIK(ac)IADK_	12	-0.23569	112.5	178010000	149710000
>WP_013240368.1 MULTISPECIES: sugar ABC transporter substrate-binding protein	K	CNLTIGKKKYAQYDEKVKGILKRSDELQAEL	_YAQYDEK(ac)VK_	7	-0.09941	132.17	358240000	218930000
>WP_013240368.1 MULTISPECIES: sugar ABC transporter substrate-binding protein	K	AETEPLVEEGIKIADKVYEKVVSALSK	_IADK(ac)VYEK_	4	0.08836	78.334	364550000	222390000
>WP_013240369.1 formate--tetrahydrofolate ligase	K	GIEVAKEVLRLIKEEKNDFQFAYDEKLPIRD	_EEK(ac)NDFQFAYDEK(ac)LPIR_	3	-0.50633	31.876	3456300	0
>WP_013240369.1 formate--tetrahydrofolate ligase	K	TTTAIGVADALNRMGKSVVVALREPSMGPVF	_MGK(ac)SVVVALR_	3	-1.6505	41.283	12723000	6599800
>WP_013240369.1 formate--tetrahydrofolate ligase	K	VVAVTGSIMKMPGLGKVPSAEKIDVDENGVI	_MPGLGK(ac)VPSAEK_	6	0.07897	73.36	12905000	10205000
>WP_013240369.1 formate--tetrahydrofolate ligase	K	EKGLPNLLKHVENITKVYKLPAVVALNAFPT	_HVENITK(ac)VYK_	7	0.84238	100.72	19849000	21780000

>WP_013240369.1 formate--tetrahydrofolate ligase	K	GAEKFLDIKCRMAGLKPDAVIIVATVRALKY	_M(ox)AGLK(ac)PDAVIIVATVR_	5	2.305	36.638	24967000	2413100
>WP_013240369.1 formate--tetrahydrofolate ligase	K	SEVWAKGGEGGIEVAKEVLRLIKEEKNDFQF	_GGEGGIEVAK(ac)EVLR_	10	1.9035	144.27	64274000	72118000
>WP_013240369.1 formate--tetrahydrofolate ligase	K	LKPDAVIIVATVRALKYNGGVPKADLNNENL	_ALK(ac)YNGGVPK_	3	0.32263	71.614	67001000	48903000
>WP_013240369.1 formate--tetrahydrofolate ligase	K	ELYGKYKAKVNYNLLKTTPGKNGKLILCTAI	_VNYNLLK(ac)TTPGK_	7	0.3268	126.34	89273000	51774000
>WP_013240369.1 formate--tetrahydrofolate ligase	K	KIYGADDVTFTNQADKEIDELEKLGFGKTPV	_IYGADDVTFTNQADK(ac)EIDELEK_	15	-1.1431	95.206	98547000	42168000
>WP_013240369.1 formate--tetrahydrofolate ligase	K	MTYKSDIEIAQECTMKDIKEIAKKLNISEDD	_SDIEIAQECTM(ox)K(ac)DIK_	12	-0.36894	157.38	125050000	68546000
>WP_013240369.1 formate--tetrahydrofolate ligase	K	VGYTRDGKPVTAHDLKAEGAMAALLKDALKP	_DGKPVTAHDLK(ac)AEGAMAALLK_	11	-0.26827	70.453	141410000	115600000
>WP_013240369.1 formate--tetrahydrofolate ligase	K	KVNYNLLKTTPGKNGKLILCTAINPTPAGEG	_NGK(ac)LILCTAINPTPAGEGK_	3	-0.53567	192.55	155840000	140990000
>WP_013240369.1 formate--tetrahydrofolate ligase	K	VTFTNQADKEIDELEKLGFGKTPVCIAKTQY	_EIDELEK(ac)LGFGK_	7	0.03335	176.48	318710000	312850000
>WP_013240369.1 formate--tetrahydrofolate ligase	K	VPKADLNNENLGALEKGLPNLLKHVENITKV	_ADLNNENLGALEK(ac)GLPNLLK_	13	0.47079	177.12	385300000	324670000
>WP_013240369.1 formate--tetrahydrofolate ligase	K	QADKEIDELEKLGFGKTPVCIAKTQYSLTDD	_LGFGK(ac)TPVCIAK_	5	-0.58561	120.57	475620000	444770000
>WP_013240369.1 formate--tetrahydrofolate ligase	K	LIKEEKNDFQFAYDEKLPIRDKIRAVAQKIY	_NDFQFAYDEK(ac)LPIRDK_	10	0.103	218.63	2.389E+09	2.195E+09
>WP_013240370.1 MULTISPECIES: carbon monoxide dehydrogenase	K	FPKEIADEIVPISEMKKLVAERTNSTPGSFG	_EIADEIVPISEM(ox)K(ac)K_	13	1.6003	137.68	84519000	75286000
>WP_013240371.1 carbon-monoxide dehydrogenase catalytic subunit	K	CIMPALAKLSKSYHTKFITTSPKAHITDSIY	_SYHTK(ac)FITTSPK_	5	-0.0102	74.46	3531300	4592200
>WP_013240371.1 carbon-monoxide dehydrogenase catalytic subunit	K	LDSAKKILKEAILNFKNRDQSKVMIPELKSK	_EAILNFK(ac)NR_	7	0.17488	68.735	8318800	5757000
>WP_013240371.1 carbon-monoxide dehydrogenase catalytic subunit	K	NNPKVVQDSAHIETIKGLIKNDVIVVVTGCA	_VVQDSAHIETIK(ac)GLIK_	12	-0.61492	43.592	23911000	1933700
>WP_013240371.1 carbon-monoxide dehydrogenase catalytic subunit	K	NFKNRDQSKVMIPELKSKAILGYSVEEIINK	_VMIPELK(ac)SK_	7	-0.32872	74.162	26040000	24130000
>WP_013240371.1 carbon-monoxide dehydrogenase catalytic subunit	K	GKQIGEVNLPPSLPEKRKELWRKLGVFPRAV	_QIGEVNLPPSLPEK(ac)R_	14	-0.09929	155.98	53480000	74472000
>WP_013240371.1 carbon-monoxide dehydrogenase catalytic subunit	K	MEDWVGAKFFIETDPKKAVEQIVNRMNEKRK	_FFIETDPK(ac)K_	8	0.29942	96.331	113010000	140870000
>WP_013240372.1 universal stress protein	K	PLDGTDRSMHSLDLLKKMFKKDEVEVTLMNI	_SMHSLDLLK(ac)K_	9	0.16862	67.334	4219100	3794800
>WP_013240372.1 universal stress protein	K	VNKFFDFGYAGDEILKKASQDNFDIIIMTKS	_FFDFGYAGDEILK(ac)K_	13	-0.8635	58.487	13113000	10680000
>WP_013240377.1 (4Fe-4S)-binding protein	K	GFIGGKKASINESMCKKCGKCETVCKFQAII	_ASINESMCK(ac)K_	9	0.04767	74.162	0	6670700
>WP_013240377.1 (4Fe-4S)-binding protein	K	FITELDKGIISRSEMKIGADGSGKLVNYLRK	_SEMK(ac)IGADGSGK_	4	0.16195	68.809	9949000	6333500
>WP_013240377.1 (4Fe-4S)-binding protein	K	TMVCINKYDINEEIAKKIENFVFLENLELVG	_YDINEEIAK(ac)K_	9	-1.9744	62.303	16846000	11526000
>WP_013240378.1 (4Fe-4S)-binding protein	K	YNAVTYGKREIGKIEKGTSRDIKCSRGILNI	_IEK(ac)GTSR_	3	-0.50356	72.789	11521000	15375000
>WP_013240378.1 (4Fe-4S)-binding protein	K	EPNGFLFLKPKVDVVKSVKVEYPIIDACKCT	_VDVVK(ac)SVK_	5	-0.0529	76.679	58227000	46933000
>WP_013240379.1 diguanylate cyclase	K	NSVLQKYKNNELEEIKTSGPAHH	_NNELEEIK(ac)TSGPAHH_	8	-0.67333	66.285	10483000	8309000
>WP_013240379.1 diguanylate cyclase	K	KAYKCSSIDINSVLQKYKNNELEEIKTSGPA	_CSSIDINSVLQK(ac)YK_	12	-0.3691	139.89	142380000	102190000
>WP_013240382.1 ATP-binding protein	K	DCSKKIKLFNGESTTKFLEEMNLKLLGEIPV	_LFNGESTTK(ac)FLEEMNLK_	9	0.07805	132.84	125910000	99062000
>WP_013240382.1 ATP-binding protein	K	IPVLNSVGNLSDPTYKNEDEDLEKVFYPIVD	_LLGEIPVLNSVGNLSDPTYK(ac)NEDEDLEK_	20	0.13576	124.28	364320000	269630000

>WP_013240382.1 ATP-binding protein	K	EDLEKVFYPIVDNVMKSLDK	_VFYPIVDNVM(ox)K(ac)SLDK_	11	1.88	109.83	472030000	68259000
>WP_013240383.1 hypothetical protein	K	SATVGKYEAEIEQINKRINELEELLRKKRCD	_YEAEIEQINK(ac)R_	10	0.38292	203.94	70996000	36321000
>WP_013240386.1 flavin reductase family protein	K	SHEMFAGKIEYVHADKEMIKDNGDIDFSKIQ	_IEYVHADK(ac)EMIK_	8	0.48205	89.629	22708000	15499000
>WP_013240386.1 flavin reductase family protein	K	SHSGRNEDKFSKLNIKVDEGIKVNAPLLADC	_LNIK(ac)VDEGIK_	4	0.20921	80.706	26692000	15742000
>WP_013240386.1 flavin reductase family protein	K	EYLGSHSGRNEDKFSKLNIKVDEGIKVNAPL	_FSK(ac)LNIK_	3	-0.32863	94.006	98757000	79539000
>WP_013240396.1 sulfite reductase subunit alpha	K	YSGLTTRLQIEVPWIKDEDAEKVIQEAKALG	_LQIEVPWIK(ac)DEDAEK_	9	2.6236	30.133	0	1807600
>WP_013240396.1 sulfite reductase subunit alpha	K	LCVTNCRQKALQLVDKKIVHNKNLCVECGEC	_ALQLVDK(ac)K_	7	-0.99797	95.909	30264000	23639000
>WP_013240396.1 sulfite reductase subunit alpha	K	NFTVEELNNINKIAEKYGRGYSGLTTRLQIE	_IAEK(ac)YGR_	4	0.14572	83.614	32110000	0
>WP_013240396.1 sulfite reductase subunit alpha	K	CRELDRKYFGTDTPHKCKIGIVGCANNCGKA	_YFGTDTPHK(ac)CK_	9	-0.50842	129.16	40065000	37195000
>WP_013240398.1 ABC transporter ATP-binding protein	K	LCLEIENDNGKINILKDINLKFKKNKIYVIT	_INILK(ac)DINLK_	5	0.18686	48.004	0	3155600
>WP_013240398.1 ABC transporter ATP-binding protein	K	ENDNGKINILKDINLKFKKNKIYVITGPNGG	_DINLK(ac)FK_	5	0.18278	105.95	81582000	70612000
>WP_013240398.1 ABC transporter ATP-binding protein ;>WP_013239339.1 MULTISPECIES: ABC transporter ATP-binding protein	K	YINRDVDSSLSGGELKRIEIATILARDLEVA	_DVDSSLSGGELK(ac)R_	12	-0.17224	41.644	1621500	0
>WP_013240400.1 membrane protein	K	FPIIKGKEKLLQADFKGAKGQAFTDMPNNFH	_LLQADFK(ac)GAK_	7	-0.15919	73.885	4150000	1458200
>WP_013240405.1 sulfurase	K	NWDRQISILPIEALEKVPSDKIEEVKKGGFT	_QISILPIEALEK(ac)VPSDK_	12	1.1528	37.083	3899900	9201000
>WP_013240405.1 sulfurase	K	RPYIVSREGRFGIVVKGGKINVGDPVEVI	_FGIVVK(ac)GGK_	6	1.4041	69.954	6888800	2078400
>WP_013240405.1 sulfurase	K	MGKVVSLNISEKRGTEKIQVPSVNVIE	_VVSLNISEK(ac)R_	9	1.0223	61.353	26186000	1336600
>WP_013240405.1 sulfurase	K	IEALEKVPSDKIEEVKKGGFTENITISGIPL	_VPSDKIEEVK(ac)K_	10	1.1941	158.42	26816000	24079000
>WP_013240409.1 class I SAM-dependent methyltransferase	K	VDSNWSKNQPVDQFYKKCLLNFGRKKPLTEE	_NQPVDQFYK(ac)K_	9	-0.41627	63.283	4971600	4960800
>WP_013240410.1 formylmethanofuran dehydrogenase subunit E	K	NTVICDKCGEGVPEHKIRMSEGKKLCLDCFE	_CGEGVPEHK(ac)IR_	9	-1.0739	40.589	0	5699800
>WP_013240410.1 formylmethanofuran dehydrogenase subunit E	K	ILESPVDEVFEIKKPKFEVPKTARLFNTVIC	_KPK(ac)FEVPK_	3	-0.5336	125.82	97587000	92154000
>WP_013240420.1 HD domain-containing protein	K	EAISKIFANIIDNKSKFTATHSKGIASLAYL	_SK(ac)FTATHSK_	2	0.6946	75.509	33826000	35406000
>WP_013240422.1 tRNA (adenosine(37)-N6)-threonylcarbamoyltransferase complex dimerization subunit type 1 TsaB	K	NLNLARASSLGELGLKLLKSGIQDDLYSFAP	_ASSLGELGLK(ac)LLK_	10	1.04	42.209	5174600	0
>WP_013240424.1 tyrosine--tRNA ligase	K	NAECFKKQFSRLVDFKDDKAILANNADWLLN	_LVDFK(ac)DDK_	5	0.22036	99.139	21161000	19438000
>WP_013240427.1 MULTISPECIES: cytochrome c551	K	MADKTIVCKDCGKEFVFTEGEQEF	_TIVCK(ac)DCGK_	5	-0.38441	54.608	0	28176000
>WP_013240427.1 MULTISPECIES: cytochrome c551	K	KEFVFTEGEQEFYKEKGFENDPVRCPECRRK	_EK(ac)GFENDPVR_	2	-0.6386	125.75	15821000	12569000
>WP_013240431.1 peptide chain release factor 3	K	SSSTKVEDLILTSDTKKVKDLKGRDLLIFQS	_VEDLILTSDTK(ac)K_	11	-0.012	73.466	5210000	5970500
>WP_013240432.1 tryptophan--tRNA ligase	K	VNAFLQDKKYLEDIYKKGAEKAYYVSSKVLR	_YLEDIYK(ac)K_	7	1.0133	94.302	10235000	3258700
>WP_013240432.1 tryptophan--tRNA ligase	K	ELKPIQDKVNAFLQDKKYLEDIYKKGAEKAY	_VNAFLQDK(ac)K_	8	-1.0044	77.192	12718000	11442000
>WP_013240435.1 aspartate kinase	K	IIVQKYGGSSVGTPEKIKNVAKTVVSRVENG	_YGGSSVGTPEK(ac)IK_	11	0.01881	90.926	19213000	10721000

>WP_013240435.1 aspartate kinase	K	SVVGIGMKTTSGVVAKMLKLFKENNIGVKMI	_TTSGVVAK(ac)MLK_	8	-0.86974	106.29	21939000	11659000
>WP_013240438.1 MULTISPECIES: ATP-dependent zinc metalloprotease FtsH	K	VIVDRPDLKGRESILKVHTKGVKVSEDVDLN	_ESILK(ac)VHTK_	5	-0.16844	65.347	0	2678500
>WP_013240491.1 MULTISPECIES: NAD-dependent alcohol dehydrogenase	K	KPEIYTFDTCKEFINKFKIGKEDLIITSEHT	_EFINK(ac)FK_	5	0.52256	86.512	49162000	44215000
>WP_013240507.1 ribonuclease R	K	FKKVLDDLEKEGLVVKTRRKRYGIPDRMGLV	_EGLVVK(ac)TR_	6	-0.02642	99.802	8206400	5926900
>WP_013240509.1 MULTISPECIES: phosphopyruvate hydratase	K	KSNEEAIQVIVEAIEKAGYTPGKDIFIALDP	_SNEEAIQVIVEAIEK(ac)AGYTPGK_	15	-0.69912	276.68	556080000	523830000
>WP_013240511.1 triose-phosphate isomerase	K	TLEERESDVTEEVLGKQIKIDLAGLEKSQVG	_ESDVTEEVLGK(ac)QIK_	11	-0.6785	33.204	4567600	24967000
>WP_013240511.1 triose-phosphate isomerase	K	GALVGGASLKSEDFAKIVNY	_SEDFAK(ac)IVNY_	6	0.16634	56.432	9998900	12544000
>WP_013240511.1 triose-phosphate isomerase	K	MNKTVTEALKLVEELKPLVSDAKCDVVVCPP	_LVEELK(ac)PLVSDAK_	6	0.43722	128.31	15650000	13222000
>WP_013240511.1 triose-phosphate isomerase	K	AQSDIDGALVGGASLKSEDFAKIVNY	_AQMAQSDIDGALVGGASLK(ac)SEDFAK_	19	-0.02586	58.418	17267000	38069000
>WP_013240511.1 triose-phosphate isomerase	K	QIKIDLAGLEKSQVGKLVVAYEPIWAIGTGK	_SQVGK(ac)LVVAYEPIWAIGTGK_	5	0.82284	57.989	25733000	13896000
>WP_013240511.1 triose-phosphate isomerase	K	SERRQYFNETDESINKKVKKAFEYDINPIVC	_RQYFNETDESINK(ac)K_	13	1.2993	122.39	56071000	42109000
>WP_013240511.1 triose-phosphate isomerase	K	EVLGKQIKIDLAGLEKSQVGKLVVAYEPIWA	_IDLAGLEK(ac)SQVGK_	8	0.30748	156.81	118160000	64481000
>WP_013240511.1 triose-phosphate isomerase	K	IRSVIAKMYGRETAEKTRIQYGGSVKPATIK	_ETAEK(ac)TR_	5	0.17703	91.9	186250000	72801000
>WP_013240511.1 triose-phosphate isomerase	K	IAGNWKMNKTVTEALKLVEELKPLVSDAKCD	_TVTEALK(ac)LVEELKPLVSDAK_	7	-4.1219	310.4	259270000	0
>WP_013240512.1 MULTISPECIES: phosphoglycerate kinase	K	LLEKNAKVILCSHLGKPKGEPKPEMSLLPVS	_VILCSHLGK(ac)PK_	9	0.32598	104.82	8572800	10229000
>WP_013240512.1 MULTISPECIES: phosphoglycerate kinase	K	MAFNKKTIEDVDVSGKRVLVRCDFNVPLKDG	_TIEDVDVSGK(ac)R_	10	-0.24831	152.07	29887000	28388000
>WP_013240512.1 MULTISPECIES: phosphoglycerate kinase	K	LDIGPKTSKLYEEAIKDAKTVVWNGPMGVFE	_LYEEAIK(ac)DAK_	7	-0.2368	91.549	34614000	32129000
>WP_013240512.1 MULTISPECIES: phosphoglycerate kinase	K	DVVVGKNARAAVKNMKNGDVVLLENTRYRKE	_NM(ox)K(ac)NGDVVLLENTR_	3	-2.3729	169.04	70361000	50612000
>WP_013240512.1 MULTISPECIES: phosphoglycerate kinase	K	PKGEPKPEMSLLPVSKRLSELLKKEVVFAAD	_GEPKPEMSLLPVSK(ac)R_	14	-0.62408	108.89	108970000	69788000
>WP_013240512.1 MULTISPECIES: phosphoglycerate kinase	K	IKDGYMGLDIGPKTSKLYEEAIKDAKTVVWN	_TSK(ac)LYEEAIK_	3	-0.01148	115.57	118360000	102240000
>WP_013240512.1 MULTISPECIES: phosphoglycerate kinase	K	KGVKLLLPIDTVIADKFDANAKPIVTDGVDI	_LLLPIDTVIADK(ac)FDANAKPIVTDGVDIK_	12	-1.2279	122.5	132310000	12859000
>WP_013240512.1 MULTISPECIES: phosphoglycerate kinase	K	EMSLLPVSKRLSELLKKEVVFAADDVVVGKN	_RLSELLK(ac)K_	7	0.01464	117.93	176860000	144870000
>WP_013240513.1 MULTISPECIES: type I glyceraldehyde-3-phosphate dehydrogenase	K	TTGAAKAVALVLPQLKGKLNGFSLRVPTPTV	_AVALVLPQLK(ac)GK_	10	0.03204	104.52	27573000	21192000
>WP_013240513.1 MULTISPECIES: type I glyceraldehyde-3-phosphate dehydrogenase	K	ELSRDVTVEEVNEAFKKAAESDMKGILGYSD	_DVTVEEVNEAFK(ac)K_	12	-2.3676	210.79	164720000	125740000
>WP_013240514.1 MULTISPECIES: transcriptional regulator	K	EDVLKFEQRIVPELVKLLEKRYNILRTIYYN	_IVPELVK(ac)LLEK_	7	0.90133	56.225	1888600	9082900
>WP_013240517.1 MULTISPECIES: tRNA (uridine(34)/cytosine(34)/5-carboxymethylaminomethyluridine(34)-2'-O)-methyltransferase TrmL	K	FYFSTTHGKNFYHEAKFKDGDFIVFGRETCG	_NFYHEAK(ac)FK_	7	-0.10315	123.63	47146000	54962000
>WP_013240521.1 MULTISPECIES: hypothetical protein	K	EGTDDKLIQVGRFYQKLNDIIGSNLPLQKIY	_FYQK(ac)LNDIIGSNLPLQK_	4	-1.5003	64.507	8476500	0
>WP_013240521.1 MULTISPECIES: hypothetical protein	K	YQKLNDIIGSNLPLQKIYRSKGLPAHLIKEK	_LNDIIGSNLPLQK(ac)IYR_	13	-0.5585	70.529	19088000	0
>WP_013240523.1 MULTISPECIES: competence/damage-inducible protein A	K	NTEEEAVELIKPIESKIREKLGINIYGENDD	_ANTEEEAVELIKPIESK(ac)IR_	17	0.56393	87.16	12248000	26596000

>WP_013240524.1 MULTISPECIES: endonuclease III	K	MDKQNIDNILKVLKETYPEAKCALNF	_QNIDNILK(ac)VLK_	8	2.2656	45.829	3395100	16124000
>WP_013240525.1 MULTISPECIES: 1-acyl-sn-glycerol-3-phosphate acyltransferase	K	TASLAKGKRGVVLIQKLTKAAILPIGISGTE	_GVVLIQK(ac)LTK_	7	0.04299	58.676	4748600	2488500
>WP_013240528.1 MULTISPECIES: cysteine synthase A	K	NFKNEDAADIYVKLEKFNPGGSIKDRAALGM	_LEK(ac)FNPGGSIK_	3	-0.53508	49.423	6298800	0
>WP_013240531.1 NADH peroxidase	K	EGEISWADEHKVGVAKGVDEKVLEELRANFT	_VGVAK(ac)GVDEK(ac)VLEELR_	5	-0.11495	83.106	2422200	0
>WP_013240531.1 NADH peroxidase	K	ENPPEKCPVCGAPSDKFVKKAEGEISWADEH	_CPVCGAPSDK(ac)FVK_	10	-0.86535	104.24	12793000	18749000
>WP_013240531.1 NADH peroxidase	K	FVKKAEGEISWADEHKVGVAKGVDEKVLEEL	_KAEGEISWADEHK(ac)VGVAK_	13	0.19402	180.78	134670000	122580000
>WP_013240531.1 NADH peroxidase	K	ETGACKGKKDLATLAKKLNYDAIHDTVHEMC	_DLATLAK(ac)K_	7	0.55056	86.011	148920000	107360000
>WP_013240531.1 NADH peroxidase	K	AKFAELLGEVVLPDTKKNLQMRVDAETGACK	_FAELLGEVVLPDTK(ac)K_	14	1.2763	133.14	308390000	190120000
>WP_013240531.1 NADH peroxidase	K	WADEHKVGVAKGVDEKVLEELRANFTGECSE	_VGVAK(ac)GVDEK(ac)VLEELR_	10	-0.11495	183.99	767870000	600880000
>WP_013240541.1 MULTISPECIES: acyl-CoA dehydrogenase	K	EGPFEEIQKEVEVTNKFPQKFFDLSIENNLY	_EVEVTNK(ac)FPQK_	7	0.00931	38.875	4309700	12662000
>WP_013240541.1 MULTISPECIES: acyl-CoA dehydrogenase	K	MSKLNEKEFQDYLKQIRALAEG	_LNEK(ac)EFQDYLK_	4	0.8425	65.395	8897200	7089600
>WP_013240555.1 MULTISPECIES: pyridoxal 5'-phosphate synthase lyase subunit PdxS	K	IIKEIQESVSIPVMAKVRIGHFVEAQVLQQL	_EIQESVSIPVMAK(ac)VR_	13	-1.1334	137.8	20361000	13270000
>WP_013240555.1 MULTISPECIES: pyridoxal 5'-phosphate synthase lyase subunit PdxS	K	GVFVGSGIFKSENPQKRATAIVLATTYYNDP	_SENPQK(ac)R_	6	-0.43244	100.82	104960000	66075000
>WP_013240556.1 MULTISPECIES: pyridoxal 5'-phosphate synthase glutaminase subunit PdxT	K	TVKNLCSIGKHVVAAKYKNILVTSFHPELTE	_HVVAAK(ac)YK_	6	-0.08441	117.02	414840000	5450300
>WP_013240561.1 MULTISPECIES: fructose-6-phosphate aldolase	K	HKNIIIKIPMTVEGLKAVKILSKENIKTNVT	_IPMTVEGLK(ac)AVK_	9	-3.3358	45.257	1965300	0
>WP_013240561.1 MULTISPECIES: fructose-6-phosphate aldolase	K	SDIATVPYKILIQMTKHPLTDIGIQRFLDDW	_ILIQMTK(ac)HPLTDIGIQR_	7	0.12476	107.98	13699000	17250000
>WP_013240561.1 MULTISPECIES: fructose-6-phosphate aldolase	K	KEAKELAKIHKNIIIKIPMTVEGLKAVKILS	_NIIIK(ac)IPMTVEGLK_	5	0.00339	40.025	15865000	4343300
>WP_013240561.1 MULTISPECIES: fructose-6-phosphate aldolase	K	ELVEQISDIFKIHDIKTEIIAASIRNPLHVI	_IHDIK(ac)TEIIAASIR_	5	0.14048	109.83	19061000	14074000
>WP_013240561.1 MULTISPECIES: fructose-6-phosphate aldolase	K	HPLTDIGIQRFLDDWKTVPNK	_FLDDWK(ac)TVPNK_	6	-0.29047	73.848	24390000	31624000
>WP_013240562.1 MULTISPECIES: hypothetical protein	K	IMNKVTEKYGTFECSKLKQKGISCDEIIDYT	_YGTFECSK(ac)LK_	8	-0.22971	93.058	17386000	9847100
>WP_013240563.1 MULTISPECIES: choline kinase	K	MCGGLFMKMEELVQKKLCLAFNDDSIMFDK	_MEELVQK(ac)K_	7	-0.97615	133.55	17772000	16337000
>WP_013240565.1 MULTISPECIES: histidine kinase	K	QQCKNKSGLGFQSTDKRLKRYYGEQYGLKIL	_SGLGFQSTDK(ac)R_	10	-0.77785	100.55	9267900	0
>WP_013240565.1 MULTISPECIES: histidine kinase	K	SLDLSESTKLSHQDVKRLSIEKEILEKKIID	_LSHQDVK(ac)R_	7	-0.18981	121.09	74479000	79897000
>WP_013240572.1 MULTISPECIES: choline trimethylamine-lyase	K	MDIREFSNKFAEATKNMSAEEQAA	_EFSNK(ac)FAEATK_	5	1.324	38.021	1872500	5210800
>WP_013240583.1 MULTISPECIES: TetR/AcrR family transcriptional regulator	K	ADTFLDYNFLMDNKMKFSEAIQEVLKIILYG	_MK(ac)FSEAIQEVLK_	2	0.09687	83.617	43891000	26688000
>WP_013240584.1 MULTISPECIES: NADPH-dependent butanol dehydrogenase	K	LPRDIYFGEDALGALKTLKGKKAVVVVGGGS	_DIYFGEDALGALK(ac)TLK_	13	-0.03473	60.474	12207000	21914000
>WP_013240584.1 MULTISPECIES: NADPH-dependent butanol dehydrogenase	K	DFDQMKKILQCIYDGKKVTF	_ILQCIYDGK(ac)K_	9	0.23598	96.89	16604000	18429000
>WP_013240584.1 MULTISPECIES: NADPH-dependent butanol dehydrogenase	K	LIEGVEPDPSVETVMKGAKIMTEFGPDWIVA	_LIEGVEPDPSVETVMK(ac)GAK_	16	-0.39312	72.819	28843000	37188000

>WP_013240631.1 MULTISPECIES: glutamine--fructose-6-phosphate transaminase (isomerizing)	K	EKVLSNKDIIQEAAEKIYKEQDVFFLGRGLD	_DIIQEAAEK(ac)IYK_	9	1.2931	65.395	6567400	3014200
>WP_013240633.1 MULTISPECIES: phosphoglucosamine mutase	K	DELEDRIQKVIESNFKDVSMPIGAEIGRKVV	_VIESNFK(ac)DVSMPIGAEIGR_	7	-0.12125	30.767	5085500	0
>WP_013240633.1 MULTISPECIES: phosphoglucosamine mutase	K	KDYVEFTKSTIDIDLKGLKVVLDCANGASYV	_STIDIDLK(ac)GLK_	8	-0.97652	67.153	5187400	4222100
>WP_013240633.1 MULTISPECIES: phosphoglucosamine mutase	K	KKNIYAEDEEIISEIKKIEEKLDGCGRVLIR	_NIYAEDEEIISEIK(ac)K_	14	-1.4314	82.102	11934000	12011000
>WP_013240633.1 MULTISPECIES: phosphoglucosamine mutase	K	TVKTKVGDRYVLEEMKKQGYKLGGEQSGHII	_YVLEEMK(ac)K_	7	-0.75459	98.392	14110000	11583000
>WP_013240633.1 MULTISPECIES: phosphoglucosamine mutase	K	MLEGQNQSEIDKIAHKLAELIESKVN	_IAHK(ac)LAELIESK_	4	-0.28638	155.08	130610000	98041000
>WP_013240634.1 MULTISPECIES: hypothetical protein	K	DILFDSKLDNLIIEHKKLKAVLVNGNEIGCD	_LDNLIIEHK(ac)K_	9	0.77462	48.44	2479000	0
>WP_013240638.1 MULTISPECIES: NAD(P)/FAD-dependent oxidoreductase	K	NKEAEAEANYVNELSKKTYYIPMYKESLNLS	_EAEAEANYVNELSK(ac)K_	14	0.6687	41.378	7460200	7721400
>WP_013240639.1 MULTISPECIES: thioredoxin	K	KFVGFRPKDAIKDALKKHI	_DAIK(ac)DALK(ac)K_	8	-0.78855	119.68	9701200	4454300
>WP_013240639.1 MULTISPECIES: thioredoxin	K	SIPTVMVFKNGNMVDKFVGFRPKDAIKDALK	_NGNM(ox)VDK(ac)FVGFRPK_	7	-0.25113	61.212	80032000	25931000
>WP_013240639.1 MULTISPECIES: thioredoxin	K	MLSPIIEEVSDELGEKAKFFKLNVDENPVTA	_MLSPIIEEVSDELGEK(ac)AK_	16	-0.18655	157.11	97709000	75203000
>WP_013240639.1 MULTISPECIES: thioredoxin	K	IEEVSDELGEKAKFFKLNVDENPVTAAQFKI	_FFK(ac)LNVDENPVTAAQFK_	3	-0.28876	189.37	149370000	92168000
>WP_013240642.1 MULTISPECIES: phosphoenolpyruvate--protein phosphotransferase	K	IGKVVVKMKTEISIEKRHIDDVIQEKERFQK	_TEISIEK(ac)R_	7	-0.85235	58.981	1654100	10400000
>WP_013240642.1 MULTISPECIES: phosphoenolpyruvate--protein phosphotransferase	K	EISIEKRHIDDVIQEKERFQKALELSKSQLE	_HIDDVIQEK(ac)ER_	9	-1.0287	134.13	3180400	6620100
>WP_013240642.1 MULTISPECIES: phosphoenolpyruvate--protein phosphotransferase	K	VFGNIGKPEDVDQVLKNGGEGIGLFRTEFLY	_VEVFGNIGKPEDVDQVLK(ac)NGGEGIGLFR_	18	0.84512	83.881	43747000	32898000
>WP_013240645.1 MULTISPECIES: citrate lyase subunit alpha	K	TAAGAKLAIVVTNLIKGRLPIIKDKVTTVTT	_LAIVVTNLIK(ac)GR_	10	0.0932	110.86	23329000	12597000
>WP_013240646.1 MULTISPECIES: citrate lyase	K	AIDTPFTDTNDYEGLKKDTTTAKKLGFTGKA	_VDAIDTPFTDTNDYEGLK(ac)K_	18	1.601	39.237	10516000	11136000
>WP_013240648.1 MULTISPECIES: fumarate hydratase	K	MEKKITTPLTEEKVKTLKAGDSVLISGT	_ITTPLTEEK(ac)VK_	9	-0.36793	48.004	4159500	3812600
>WP_013240648.1 MULTISPECIES: fumarate hydratase	K	MIGKGLRSKEVIESMKKNKAVYFAAIGGAAA	_EVIESMK(ac)K_	7	-0.61276	79.906	5854800	4173700
>WP_013240649.1 MULTISPECIES: fumarate hydratase	K	AKKALVRPLSERNKNKFYSDLENELLDKINL	_NK(ac)FYSDLENELLDK_	2	0.55679	82.102	143840000	51012000
>WP_013240650.1 MULTISPECIES: methylaspartate ammonia-lyase	K	AIRYGITQAILDAVAKSKKITMAEVIRNEYN	_YGITQAILDAVAK(ac)SK_	13	0.50042	43.297	3390500	0
>WP_013240650.1 MULTISPECIES: methylaspartate ammonia-lyase	K	ALINNVEEKLGMHGEKLLEYVKWLRNRVIEL	_LGMHGEK(ac)LLEYVK_	7	0.22186	55.899	22986000	10163000
>WP_013240651.1 MULTISPECIES: methylaspartate mutase subunit E	K	RLPMSKDLEDEISVIKAEVKCILDKVFELGK	_DLEDEISVIK(ac)AEVK_	10	-1.9166	57.804	7932200	0
>WP_013240651.1 MULTISPECIES: methylaspartate mutase subunit E	K	VAAMKALEQQIDEYLKKYGYNDVYITTVFHQ	_ALEQQIDEYLK(ac)K_	11	1.3431	79.82	8057200	8225500
>WP_013240651.1 MULTISPECIES: methylaspartate mutase subunit E	K	SYTRQNRYDECEVGIKESKSQGRSLLNGFPG	_YDECEVGIK(ac)ESK_	9	-0.45627	72.958	9189100	1917400
>WP_013240651.1 MULTISPECIES: methylaspartate mutase subunit E	K	LAQPRAGVALIDDHIKLLKHLQDEGGADLLP	_AGVALIDDHIK(ac)LLK_	11	-0.23267	69.716	80090000	59047000
>WP_013240652.1 MULTISPECIES: hypothetical protein	K	DIMIGFNKAYAELQSKLKDKKVDFVKKLACS	_AYAELQSK(ac)LK_	8	0.01956	59.426	3167200	2939300
>WP_013240653.1 MULTISPECIES: methylaspartate mutase subunit S	K	IYPPGTLPEVTINDLKKDLNV	_IYPPGTLPEVTINDLK(ac)K_	16	0.00041	42.338	6019900	27631000
>WP_013240653.1 MULTISPECIES: methylaspartate mutase subunit S	K	GNLVVGKQKWEDVYNKFKSMGFDRIYPPGTL	_WEDVYNK(ac)FK_	7	0.02077	57.836	34201000	23392000

>WP_013240659.1 MULTISPECIES: 30S ribosomal protein S9	K	SRALLKADENLRPELKKAGFLTRDPRMKERK	_ADENLRPELK(ac)K_	10	0.08539	109.24	18631000	11755000
>WP_013240659.1 MULTISPECIES: 30S ribosomal protein S9	K	KKYGLKKARRAPQFSKR	_RAPQFSK(ac)R_	7	0.37989	149.57	23420000	9265100
>WP_013240659.1 MULTISPECIES: 30S ribosomal protein S9	K	KVIVNQPLELTSTKGKFNVLVNVHGGGFTGQ	_GK(ac)FNVLVNVHGGGFTGQAGAIR_	2	-0.1199	157.97	338580000	281100000
>WP_013240660.1 MULTISPECIES: 50S ribosomal protein L13	K	AEHNHEAQKPEVLELKY	_GAEHNHEAQKPEVLELK(ac)Y_	17	0.23077	67.605	0	4136000
>WP_013240663.1 MULTISPECIES: energy-coupling factor transporter ATPase	K	AFGPKNLGLSDDDINKRVKRAMNMVGLKYED	_NLGLSDDDINK(ac)R_	11	1.6011	83.314	5928800	0
>WP_013240664.1 MULTISPECIES: energy-coupling factor transporter ATPase	K	IIMEGSPRNIFSQVSKMKKIGLDVPQMTELA	_NIFSQVSK(ac)MK_	8	-0.81162	69.979	9884700	5941800
>WP_013240665.1 MULTISPECIES: 50S ribosomal protein L17	K	TEEEVVKNLFTDIAPKYAERNGGYTRMYKMG	_NLFTDIAPK(ac)YAER_	9	-0.05	151.54	201800000	181220000
>WP_013240666.1 MULTISPECIES: DNA-directed RNA polymerase subunit alpha	K	MPIGTIPVDSIYTPIKRVNFAVENTRVGQIT	_SEDMPIGTIPVDSIYTPIK(ac)R_	19	-1.1975	43.534	11331000	9762400
>WP_013240666.1 MULTISPECIES: DNA-directed RNA polymerase subunit alpha	K	TIEELDLSVRSYNCLKRAGINTVQELTERTM	_SYNCLK(ac)R_	6	-0.35508	86.113	23155000	15047000
>WP_013240669.1 MULTISPECIES: 30S ribosomal protein S13	K	VTIEGDLRREIALNIKRLIEIGSYRGIRHRR	_REIALNIK(ac)R_	8	-0.40597	154.01	85548000	72157000
>WP_013240670.1 MULTISPECIES: translation initiation factor IF-1	K	VELESGHKILAHISGKLRMNFIRILPGDKVT	_ILAHISGK(ac)LR_	8	0.56755	96.89	8370600	14866000
>WP_013240671.1 MULTISPECIES: adenylate kinase	K	FRKNISEKTPLGVEAKKYLDNGQLVPDEVTI	_TPLGVEAK(ac)K_	8	0.22667	87.149	19740000	29815000
>WP_013240671.1 MULTISPECIES: adenylate kinase	K	PHISTGDIFRKNISEKTPLGVEAKKYLDNGQ	_NISEK(ac)TPLGVEAK_	5	0.43341	116.98	52409000	32088000
>WP_013240673.1 MULTISPECIES: 50S ribosomal protein L15	K	KSKDGLKILGNGELQKKLTVKAAKFTKTAVE	_ILGNGELQK(ac)K_	9	0.6646	79.974	15120000	18313000
>WP_013240673.1 MULTISPECIES: 50S ribosomal protein L15	K	AFEDGTEITPELLLEKKVIKKSKDGLKILGN	_LNAFEDGTEITPELLLEK(ac)K_	18	0.07909	149.47	447900000	179650000
>WP_013240675.1 MULTISPECIES: 30S ribosomal protein S5	K	IAGGPSRAVLELAGLKDVRAKSVGSNNPRNM	_AVLELAGLK(ac)DVR_	9	0.76316	52.555	5847900	2767200
>WP_013240675.1 MULTISPECIES: 30S ribosomal protein S5	K	SIEIPEAIRKGIEDAKKNLIEVPIIGTTVPH	_GIEDAK(ac)K_	6	1.1038	88.029	21375000	2394800
>WP_013240676.1 MULTISPECIES: 50S ribosomal protein L18	K	AAKVVGGQTIAKKAIEKGIKEVVFDRGGYIYH	_AIEK(ac)GIK_	4	0.05765	78.616	26569000	17199000
>WP_013240676.1 MULTISPECIES: 50S ribosomal protein L18	K	GSNKEAAKVVGGQTIAKKAIEKGIKEVVFDRG	_VVGGQTIAK(ac)K_	8	0.2625	104.17	345510000	228220000
>WP_013240677.1 MULTISPECIES: 50S ribosomal protein L6	K	RKDMNIAVEDNSIVVKRNNDEKESRALHGLT	_KDMNIAVEDNSIVVK(ac)R_	15	-0.19846	68.919	24287000	13906000
>WP_013240677.1 MULTISPECIES: 50S ribosomal protein L6	K	IRNWRRPEPYKGKGIKYENEVIRRKEGKTGK	_GIK(ac)YENEVIR_	3	-0.34583	152.28	112480000	100580000
>WP_013240678.1 MULTISPECIES: 30S ribosomal protein S8	K	SMKYGKDKERVITGLKRISKPGLRVYCRKEE	_VITGLK(ac)R_	6	0.15389	132.72	88339000	63310000
>WP_013240680.1 MULTISPECIES: 50S ribosomal protein L5	K	IGIKEQLIFPEIEYDKIDKVRGMDVIFVTTA	_EQLIFPEIEYDK(ac)IDK_	12	-1.1023	60.474	5965400	6290500
>WP_013240680.1 MULTISPECIES: 50S ribosomal protein L5	K	KEQLIFPEIEYDKIDKVRGMDVIFVTTANTD	_EQLIFPEIEYDKIDK(ac)VR_	15	1.4937	104.44	41796000	15876000
>WP_013240680.1 MULTISPECIES: 50S ribosomal protein L5	K	NIALPRVRDFSGVPDKSFDGRGNYSIGIKEQ	_DFSGVPDK(ac)SFDGR_	8	-0.06983	126.95	67262000	41557000
>WP_013240680.1 MULTISPECIES: 50S ribosomal protein L5	K	MSSRLQEKYEKEVIPALMDKFKY	_LQEK(ac)YEK_	4	0.16722	101.64	322390000	126540000
>WP_013240680.1 MULTISPECIES: 50S ribosomal protein L5	K	LQEKYEKEVIPALMDKFKYKNIMQAPKLDKI	_EVIPALM(ox)DK(ac)FK_	9	0.17114	93.561	516830000	285100000
>WP_013240681.1 MULTISPECIES: 50S ribosomal protein L24	K	MVISGKDKGKTGEVLKVMPKTGKVIVKDVNV	_TGEVLK(ac)VMPK_	6	1.1587	83	16478000	1816700
>WP_013240681.1 MULTISPECIES: 50S ribosomal protein L24	K	KHQKPNKQNMQGGIIKKEAPIYSSKVMLYCD	_QNMQGGIIK(ac)K_	9	0.17745	84.213	29552000	17075000

>WP_013240682.1 MULTISPECIES: 50S ribosomal protein L14	K	TIVASVKSATPGGVVKKGEVVKAVIVRSVRG	_SATPGGVVK(ac)K_	9	0.27939	99.653	40860000	21819000
>WP_013240683.1 MULTISPECIES: 30S ribosomal protein S17	K	PLSKDKRWRLVEIVEKAK	_LVEIVEK(ac)AK_	7	0.75072	81.338	45590000	1964400
>WP_013240684.1 MULTISPECIES: 50S ribosomal protein L29	K	NPMRIKEVKKSIAQIKTILREEELRSFEQ	_SIAQIK(ac)TILR_	6	0.32677	142	53379000	31449000
>WP_013240685.1 MULTISPECIES: 50S ribosomal protein L16	K	VPEPVAREAMRLASHKLPIKTKFVTRKDFEE	_LASHK(ac)LPIK_	5	0.0471	111.46	17355000	15997000
>WP_013240686.1 MULTISPECIES: 30S ribosomal protein S3	K	IRKFVKEKTAIAGVSKIQIERAAKRVKLNVF	_TAIAGVSK(ac)IQIER_	8	0.15513	57.136	3268000	31483000
>WP_013240686.1 MULTISPECIES: 30S ribosomal protein S3	K	ADAQLMAENVALQLEKRISFRRAMKQTIQRA	_QAEADAQLMAENVALQLEK(ac)R_	19	-0.12157	63.578	4729600	2693500
>WP_013240686.1 MULTISPECIES: 30S ribosomal protein S3	K	IGVKVWVYRGEVLPAKKPVENKEEVKA	_GEVLPAK(ac)KPVENK_	7	0.27093	57.804	10568000	8220500
>WP_013240686.1 MULTISPECIES: 30S ribosomal protein S3	K	PGMIIGKGGQGINALKAEIQKMVTEKTVLIN	_GGQGINALK(ac)AEIQK_	9	0.1404	116.9	51070000	28794000
>WP_013240686.1 MULTISPECIES: 30S ribosomal protein S3	K	YADKKNFADNIVEDNKIRKFVKEKTAIAGVS	_NFADNIVEDNK(ac)IR_	11	-0.405	217.09	123970000	70762000
>WP_013240688.1 MULTISPECIES: 30S ribosomal protein S19	K	ALTRTYRGHVADKTEKTTRVASQK	_GHVADKTEK(ac)TTR_	9	0.60742	75.695	4460100	5136500
>WP_013240688.1 MULTISPECIES: 30S ribosomal protein S19	K	GEFALTRTYRGHVADKTEKTTRVASQK	_GHVADK(ac)TEK_	6	0.59941	96.342	16965000	13205000
>WP_013240688.1 MULTISPECIES: 30S ribosomal protein S19	K	SRSVKKGPYVQEALLKKINELNKNGEKKVVK	_KGPYVQEALLK(ac)K_	11	-0.23735	124.51	51871000	20880000
>WP_013240689.1 MULTISPECIES: 50S ribosomal protein L2	K	EITTDVPEKSLLAALKRSGGRNAHGKITVRH	_SLLAALK(ac)R_	7	-0.45203	87.323	5342400	2896200
>WP_013240689.1 MULTISPECIES: 50S ribosomal protein L2	K	VRSAGASAQLMAKEGKYATLRLPSGEMRHVR	_EGK(ac)YATLR_	3	-0.34482	92.439	7116500	5154400
>WP_013240690.1 MULTISPECIES: 50S ribosomal protein L23	K	ARYIGKKKRVGVHIGKRADFKKAIVKLKEDS	_VGVHIGK(ac)R_	7	-0.50969	63.306	16530000	772110
>WP_013240690.1 MULTISPECIES: 50S ribosomal protein L23	K	RRPVVTEKSMAGMNEKKYTFIVDIHANKSMI	_SMAGMNEK(ac)K_	8	-0.506	84.213	17570000	6487000
>WP_013240691.1 MULTISPECIES: 50S ribosomal protein L4	K	DYRISLTKSVKRVAMKSALSSKVADNEMVVL	_VAMK(ac)SALSSK_	4	1.135	79.693	1081700	4984100
>WP_013240691.1 MULTISPECIES: 50S ribosomal protein L4	K	VVALLANKRQGTQSAKTRAEVSGGGIKPWRQ	_QGTQSAK(ac)TR_	7	0.49229	104.05	6748200	4270800
>WP_013240691.1 MULTISPECIES: 50S ribosomal protein L4	K	IPVNNLNVYDLLKYEKLIITKDAVSKIEEVY	_YEK(ac)LIITK_	3	-0.41204	75.294	8868400	8666800
>WP_013240691.1 MULTISPECIES: 50S ribosomal protein L4	K	PKTKEIVKMLDAFNAKKALIVTAESNKNVYV	_MLDAFNAK(ac)K_	8	-0.97895	55.567	10439000	1386200
>WP_013240691.1 MULTISPECIES: 50S ribosomal protein L4	K	LENLELETPKTKEIVKMLDAFNAKKALIVTA	_EIVK(ac)MLDAFNAK_	4	0.31803	99.539	25307000	16200000
>WP_013240692.1 MULTISPECIES: 50S ribosomal protein L3	K	IREKLVNKPMKGHFEKAGVTLKRFIKEFKLE	_GHFEK(ac)AGVTLK_	5	-0.5243	56.225	13492000	10097000
>WP_013240692.1 MULTISPECIES: 50S ribosomal protein L3	K	IPVTVIEAGPCAVIQKKTVEKDGYEAVQVGF	_VIPVTVIEAGPCAVIQK(ac)K_	17	-0.61084	85.619	15460000	21041000
>WP_013240692.1 MULTISPECIES: 50S ribosomal protein L3	K	NKPMKGHFEKAGVTLKRFIKEFKLENASEYK	_AGVTLK(ac)R_	6	0.41423	151.17	40135000	25052000
>WP_013240692.1 MULTISPECIES: 50S ribosomal protein L3	K	SMGACSDPSRTFKNMKMAGHMGNRNTTVLNL	_NMK(ac)MAGHMGNR_	3	-0.16806	112.75	57559000	33510000
>WP_013240692.1 MULTISPECIES: 50S ribosomal protein L3	K	LEVAKVIPEKNIILIKGGVPGPNKGLVAIRN	_NIILIK(ac)GGVPGPNK_	6	0.15467	123.64	79595000	54519000
>WP_013240692.1 MULTISPECIES: 50S ribosomal protein L3	K	KRFIKEFKLENASEYKEGGQEIKADVFTAGDK	_LENASEYK(ac)EGGQEIK_	8	0.11382	157.38	100630000	75735000
>WP_013240692.1 MULTISPECIES: 50S ribosomal protein L3	K	NTTVLNLEVAKVIPEKNIILIKGGVPGPNKG	_VIPEK(ac)NIILIK_	5	0.16088	73.499	152630000	139470000
>WP_013240693.1 MULTISPECIES: elongation factor Tu	K	SKEGKAAATKYDEIDKAPEEKERGITINTAH	_YDEIDK(ac)APEEK_	6	0.90622	75.479	10584000	9364900
>WP_013240693.1 MULTISPECIES: elongation factor Tu	K	ERGQVLAKPGTVHPHKKFVGQVYVLKKEEGG	_PGTVHPHK(ac)K_	8	0.85697	72.928	25838000	72400000
>WP_013240693.1 MULTISPECIES: elongation factor Tu	K	AAATKYDEIDKAPEEKERGITINTAHVEYET	_YDEIDKAPEEK(ac)ER_	11	0.20251	107.37	63468000	39916000

>WP_013240693.1 MULTISPECIES: elongation factor Tu	K	RGITINTAHVEYETDKRHYAHVDCPGHADYV	_GITINTAHVEYETDK(ac)R_	15	-0.00831	210.9	150740000	159190000
>WP_013240693.1 MULTISPECIES: elongation factor Tu	K	SGVLKIGDELEIVGLKEEKKKTTCTGVEMFR	_IGDELEIVGLK(ac)EEK_	11	-0.9803	136.17	172270000	175030000
>WP_013240693.1 MULTISPECIES: elongation factor Tu	K	GRGTVATGRVESGVLKIGDELEIVGLKEEKK	_VESGVLK(ac)IGDELEIVGLK_	7	0.19709	139.67	187850000	89053000
>WP_013240693.1 MULTISPECIES: elongation factor Tu	K	LKIGDELEIVGLKEEKKKTTCTGVEMFRKLL	_IGDELEIVGLKEEK(ac)K_	14	-0.03934	189.33	595080000	459670000
>WP_013240693.1 MULTISPECIES: elongation factor Tu	K	AITMVLSKEGKAAATKYDEIDKAPEEKERGI	_AAATK(ac)YDEIDKAPEEK_	5	0.19461	185.02	1.069E+09	1.117E+09
>WP_013240694.1 MULTISPECIES: elongation factor G	K	PKTKAGQEKMGIALAKLAEEDPTFKTYTDQE	_MGIALAK(ac)LAEEDPTFK_	7	1.0401	89.541	13158000	10098000
>WP_013240694.1 MULTISPECIES: elongation factor G	K	RSLRVLDGAVTVLDAKSGVEPQTETVWRQAD	_VLDGAVTVLDAK(ac)SGVEPQTETVWR_	12	0.11915	37.896	13705000	9626300
>WP_013240694.1 MULTISPECIES: elongation factor G	K	VIRVAIEPKTKAGQEKMGIALAKLAEEDPTF	_AGQEK(ac)MGIALAK_	5	0.77875	102.66	30833000	22399000
>WP_013240694.1 MULTISPECIES: elongation factor G	K	MTFDHYEEVPKSIQEKIVGERNS	_SIQEK(ac)IVGER_	5	-0.1698	109.42	36037000	30903000
>WP_013240694.1 MULTISPECIES: elongation factor G	K	VAYKETIRKTVKAQGKFIRQSGGHGQYGDCS	_AQGK(ac)FIR_	4	0.59952	114.54	41925000	24457000
>WP_013240694.1 MULTISPECIES: elongation factor G	K	MEREYPLEKFRNIGIMAHIDAGKT	_EYPLEK(ac)FR_	6	0.06758	122.13	114960000	70660000
>WP_013240694.1 MULTISPECIES: elongation factor G	K	GTVMDEVKIPDELADKAEEYRTALIEAVADV	_IPDELADK(ac)AEEYR_	8	-1.4531	196.7	147570000	102030000
>WP_013240695.1 MULTISPECIES: 30S ribosomal protein S7	K	RDVLPDPVYNSKVVTKLVNSIMEDGKKGVAQ	_VVTK(ac)LVNSIMEDGKK_	4	-2.4109	38.028	3130400	0
>WP_013240695.1 MULTISPECIES: 30S ribosomal protein S7	K	SKVVTKLVNSIMEDGKKGVAQKICYGAFDII	_LVNSIMEDGK(ac)K_	10	0.18374	123.8	14044000	12039000
>WP_013240695.1 MULTISPECIES: 30S ribosomal protein S7	K	VAQKICYGAFDIIHEKTNKEAMEVFEEAMNN	_ICYGAFDIIHEK(ac)TNK_	12	0.8668	100.54	37151000	32867000
>WP_013240696.1 MULTISPECIES: 30S ribosomal protein S12	K	ALKECPQKRGVCTVVKTTTPKKPNSALRKIA	_GVCTVVK(ac)TTTPK_	7	0.34436	88.021	17387000	9368100
>WP_013240697.1 MULTISPECIES: 50S ribosomal protein L7ae-like protein	K	MVNRLKGNKVVGLKQTVKAIKNSTAKTVY	_VVGLK(ac)QTVK(ac)AIK_	5	-1.8878	70.908	10328000	5171000
>WP_013240698.1 MULTISPECIES: DNA-directed RNA polymerase subunit beta	K	ASFQETTRVLTDAAIKGKIDPLLGLKENVII	_VLTDAAIK(ac)GK_	8	0.36396	44.309	6115300	7717500
>WP_013240698.1 MULTISPECIES: DNA-directed RNA polymerase subunit beta	K	QEDCGTHEGYDVSEIKEGNEVIESLAERLTG	_QEDCGTHEGYDVSEIK(ac)EGNEVIESLAER_	16	-0.52454	33.265	6775600	0
>WP_013240698.1 MULTISPECIES: DNA-directed RNA polymerase subunit beta	K	IIIPKDVYMDPKIAEKIEASGIKKVKIRSVF	_IAEK(ac)IEASGIK_	4	-0.37829	89.805	11869000	12723000
>WP_013240698.1 MULTISPECIES: DNA-directed RNA polymerase subunit beta	K	KRLLGEADAAVDKIEKMYRRGFISEEERYQR	_LLGEADAAVDKIEK(ac)MYR_	14	1.0096	96.993	15090000	12952000
>WP_013240698.1 MULTISPECIES: DNA-directed RNA polymerase subunit beta	K	CYMKHGATKTSIMLDKIKARGYHYSTISGIT	_TSIMLDK(ac)IK_	7	0.90988	74.162	17198000	3402900
>WP_013240698.1 MULTISPECIES: DNA-directed RNA polymerase subunit beta	K	FVMKKLVESGLAHNIKSAKRMVERVQTQVWD	_LVESGLAHNIK(ac)SAK_	11	-0.45277	68.034	18134000	16786000
>WP_013240698.1 MULTISPECIES: DNA-directed RNA polymerase subunit beta	K	ISTHGARKGNADTALKTADSGYLTRRLVDVS	_KGNADTALK(ac)TADSGYLTR_	9	-0.52353	149.09	38959000	37875000
>WP_013240698.1 MULTISPECIES: DNA-directed RNA polymerase subunit beta	K	DPKETQLLKKQLLTEKEYREAADKYGEQNFV	_QLLTEK(ac)EYR_	6	0.44363	103.7	81182000	43673000
>WP_013240699.1 MULTISPECIES: DNA-directed RNA polymerase subunit beta	K	VGYMYILKLAHLVDDKIHARSTGPYSLVTQQ	_LAHLVDDK(ac)IHAR_	8	-1.8953	37.426	2425700	1414000
>WP_013240699.1 MULTISPECIES: DNA-directed RNA polymerase subunit beta	K	RANEPIDENGNFIDNKITVRDKEDVIVVPGQ	_ANEPIDENGNFIDNK(ac)ITVR_	15	0.35667	59.512	4979400	4575800

>WP_013240699.1 MULTISPECIES: DNA-directed RNA polymerase subunit beta	K	EEITRDIPNVGEDALKDIDERGIIRIGAEVR	_DIPNVGEDALK(ac)DIDER_	11	0.07928	82.259	13087000	10580000
>WP_013240700.1 MULTISPECIES: 50S ribosomal protein L7/L12	K	IKAVREATGLGLKEAKALVDGAPKTLKEAVS	_EATGLGLK(ac)EAK(ac)ALVDGAPK_	11	0.12758	51.819	25053000	17406000
>WP_013240700.1 MULTISPECIES: 50S ribosomal protein L7/L12	K	EKTEFDVVLKAAGGEKIKVIKAVREATGLGL	_AAGGEK(ac)IK_	6	0.53345	63.419	78930000	33901000
>WP_013240700.1 MULTISPECIES: 50S ribosomal protein L7/L12	K	KTLKEAVSKEDAEAMKAKFEEIGAEIELK	_EDAEAMK(ac)AK_	7	-0.05584	71.614	152390000	83942000
>WP_013240700.1 MULTISPECIES: 50S ribosomal protein L7/L12	K	AAAGAEEKTEFDVVLKAAGGEKIKVIKAVRE	_TEFDVVLK(ac)AAGGEK_	8	-0.18878	141.46	238200000	238340000
>WP_013240700.1 MULTISPECIES: 50S ribosomal protein L7/L12	K	LKEAVSKEDAEAMKAKFEEIGAEIELK	_AK(ac)FEEIGAEIELK_	2	-0.15138	113.76	306900000	295590000
>WP_013240700.1 MULTISPECIES: 50S ribosomal protein L7/L12	K	IKVIKAVREATGLGLKEAKALVDGAPKTLKE	_EATGLGLK(ac)EAK(ac)ALVDGAPK_	8	0.12758	73.632	1.009E+09	754100000
>WP_013240701.1 MULTISPECIES: 50S ribosomal protein L10	K	EVKEARVQEIKEKMEKAQGVILAKYQGLTVE	_MEK(ac)AQGVILAK_	3	-0.17064	39.625	6006000	1987700
>WP_013240701.1 MULTISPECIES: 50S ribosomal protein L10	K	VILAKYQGLTVEEDTKLRKELRDAGVEYKVY	_YQGLTVEEDTK(ac)LR_	11	-4.7427	79.633	11464000	3427000
>WP_013240701.1 MULTISPECIES: 50S ribosomal protein L10	K	RILNDFSKDHKVLELKAGIVQGEIFDENKVK	_VLELK(ac)AGIVQGEIFDENK_	5	0.16858	65.716	18341000	5662300
>WP_013240702.1 MULTISPECIES: 50S ribosomal protein L1	K	SGTVTFDVAKAIEEIKAGKVEYRVDKTSIVH	_AIEEIK(ac)AGK_	6	-0.82156	67.897	0	7456100
>WP_013240702.1 MULTISPECIES: 50S ribosomal protein L1	K	MGKKYVESAKLIDKGTLYEPSEAIE	_YVESAK(ac)LIDK_	6	1.003	78.653	19937000	14161000
>WP_013240702.1 MULTISPECIES: 50S ribosomal protein L1	K	EPSEAIELVLKTSKAKFDETIELALKLGVDP	_AK(ac)FDETIELALK_	2	0.61258	95.401	27537000	18134000
>WP_013240702.1 MULTISPECIES: 50S ribosomal protein L1	K	AVVKAKPSASKGQYLKSVVISSTMGPGIKIN	_GQYLK(ac)SVVISSTMGPGIK_	5	-0.0389	79.97	39395000	39289000
>WP_013240702.1 MULTISPECIES: 50S ribosomal protein L1	K	QKLLDNFHTLMEAVVKAKPSASKGQYLKSVV	_LLDNFHTLMEAVVK(ac)AK_	14	-0.27026	207.37	541740000	294630000
>WP_013240702.1 MULTISPECIES: 50S ribosomal protein L1	K	SIVHVPIGKKSFESQKLLDNFHTLMEAVVKA	_SFESQK(ac)LLDNFHTLMEAVVK_	6	-1.3446	302.52	1.747E+09	1.876E+09
>WP_013240704.1 MULTISPECIES: transcription termination/antitermination protein NusG	K	PLENFLAVIQEINTEKRKIKALVNMFGRETP	_SGPLENFLAVIQEINTEK(ac)R_	18	1.6421	268.55	0	26119000
>WP_013240706.1 MULTISPECIES: 50S ribosomal protein L33	K	NTMKNKKNDPDRLEMKKYCPFCHKHTVHKET	_NDPDRLEMK(ac)K_	9	-0.43163	57.348	7554900	6524600
>WP_013240706.1 MULTISPECIES: 50S ribosomal protein L33	K	EMKKYCPFCHKHTVHKETR	_HTVHK(ac)ETR_	5	-0.57728	137.9	122130000	85003000
>WP_013240709.1 MULTISPECIES: 23S rRNA (guanosine(2251)-2'-O)-methyltransferase RlmB	K	YMKICKVTNLNTTIEKLKKDGIWVYGADMSG	_VTNLNTTIEK(ac)LKK_	10	-0.08179	54.524	2594300	6825900
>WP_013240711.1 MULTISPECIES: cysteine--tRNA ligase	K	KFIKEYYKDADALHIKRATVNPRATLYITKI	_DADALHIK(ac)R_	8	0.13935	75.378	1100900	4332600
>WP_013240711.1 MULTISPECIES: cysteine--tRNA ligase	K	SKRGSIEKEIEALIEKRQIARSEKNWALSDK	_EIEALIEK(ac)R_	8	-0.57379	81.338	6119600	5918500
>WP_013240711.1 MULTISPECIES: cysteine--tRNA ligase	K	NLLDNKEDIPISENEKEYRNKLGKYKQRYIE	_EDIPISENEK(ac)EYR_	10	-0.07446	92.856	16817000	15978000
>WP_013240713.1 MULTISPECIES: PIN/TRAM domain-containing protein	K	VLAELRHIADSSDGLKRTRGRRGLDILNKIQ	_HIADSSDGLK(ac)R_	10	0.5494	212.72	40830000	34952000
>WP_013240713.1 MULTISPECIES: PIN/TRAM domain-containing protein	K	EKDFPNIPEVDAKLLKLSQVLDGKVVTNDYN	_LLK(ac)LSQVLDGK_	3	-0.06517	134.2	56978000	61775000
>WP_013240714.1 MULTISPECIES: hypothetical protein	K	SESLLRHKSILDIITKLQESNARINRAVAKS	_SILDIITK(ac)LQESNAR_	8	1.3665	182.89	547000000	1.302E+09
>WP_013240715.1 MULTISPECIES: DNA integrity scanning protein DisA	K	NKIPRIPTNVIENLVKNFKELKGVMEASYEQ	_IPTNVIENLVK(ac)NFK_	11	1.341	192.4	0	33641000
>WP_013240715.1 MULTISPECIES: DNA integrity scanning protein DisA	K	MRLEKDKELKSILKLLAPGTQLREGLRNI	_SILK(ac)LLAPGTQLR_	4	0.24763	59.898	18707000	15138000
>WP_013240717.1 MULTISPECIES: ATP-dependent Clp protease ATP-binding subunit	K	CIGATTIDEYRKYIEKDAALERRFQPIIVGE	_YIEK(ac)DAALER_	4	0.20994	68.069	8676800	5933800

>WP_013240717.1 MULTISPECIES: ATP-dependent Clp protease ATP-binding subunit	K	QLEEEDLKQIVKLMLKDVSSRLKDQEIEIGF	_LMLK(ac)DVSSR_	4	-0.08105	51.092	10375000	18972000
>WP_013240717.1 MULTISPECIES: ATP-dependent Clp protease ATP-binding subunit	K	EEILHKRVVGQDEAVKSISKAVRRARVGLKD	_VVGQDEAVK(ac)SISK_	9	-0.46521	78.763	12758000	10220000
>WP_013240717.1 MULTISPECIES: ATP-dependent Clp protease ATP-binding subunit	K	VVSQWTNIPVEKLTEKESERLLKLEEILHKR	_LTEK(ac)ESER_	4	0.4207	111.95	47101000	25197000
>WP_013240717.1 MULTISPECIES: ATP-dependent Clp protease ATP-binding subunit	K	IPVEKLTEKESERLLKLEEILHKRVVGQDEA	_LLK(ac)LEEILHK_	3	0.11224	111.65	84795000	105070000
>WP_013240717.1 MULTISPECIES: ATP-dependent Clp protease ATP-binding subunit	K	EKESERLLKLEEILHKRVVGQDEAVKSISKA	_LEEILHK(ac)R_	7	0.36735	213.35	198380000	166520000
>WP_013240719.1 MULTISPECIES: excinuclease Uvr	K	LKSDLQKAVSLEEYEKAATIRDAIKEIEKQV	_AVSLEEYEK(ac)AATIR_	9	-1.3473	73.91	2596600	17954000
>WP_013240719.1 MULTISPECIES: excinuclease Uvr	K	SECYKNFSNILGPVIKRVQANLGHTGKIPKN	_NFSNILGPVIK(ac)R_	11	0.45138	149.94	19006000	39189000
>WP_013240723.1 MULTISPECIES: UDP-N-acetylmuramoyl-L-alanine--D-glutamate ligase	K	IFKTPSMRIDSPALVKAKQNGAYVTSEMEEF	_IDSPALVK(ac)AK_	8	1.5283	74.162	7225600	7054800
>WP_013240724.1 MULTISPECIES: glycine--tRNA ligase	K	RKDFNVEYDDAGSIGKRYRREDEIGTPYCIT	_KDFNVEYDDAGSIGK(ac)R_	15	0.31462	89.911	19544000	15998000
>WP_013240724.1 MULTISPECIES: glycine--tRNA ligase	K	AQGVEKASADGWSNEKLEKYIDDNNIVCPSC	_ASADGWSNEK(ac)LEK_	10	0.29282	168.61	53602000	40844000
>WP_013240725.1 MULTISPECIES: lysine--tRNA ligase	K	LNDPIVQRERFMQQLKERELGDDEAYMMDED	_FMQQLK(ac)ER_	6	1.2283	69.423	2842100	10368000
>WP_013240725.1 MULTISPECIES: lysine--tRNA ligase	K	NSDVRDTFLKRTAIIKSIREFLDNKDYIEVE	_TAIIK(ac)SIR_	5	0.22508	57.859	3450200	17750000
>WP_013240725.1 MULTISPECIES: lysine--tRNA ligase	K	VAKSLKPLPEKWHGLKDPDLRYRQRYVDLII	_WHGLK(ac)DPDLR_	5	-0.44159	81.338	10230000	17501000
>WP_013240725.1 MULTISPECIES: lysine--tRNA ligase	K	VEAVKKFANVDFDEVKDDEEAREIAKGKHIE	_FANVDFDEVK(ac)DDEEAR_	10	2.0656	94.544	15165000	30259000
>WP_013240725.1 MULTISPECIES: lysine--tRNA ligase	K	MSKDEKKLHALEEKSNELIRERLQKFEDL	_LHALEEK(ac)SNELIR_	7	0.91234	140.82	154350000	105520000
>WP_013240725.1 MULTISPECIES: lysine--tRNA ligase	K	PVEISPLTKKKRGNPKFTERFEGFIFGREIC	_RGNPK(ac)FTER_	5	0.1668	175.93	494930000	368860000
>WP_013240729.1 MULTISPECIES: type III pantothenate kinase	K	VPIIVGPGVKTGINVKYDNPREVGADRIVNA	_TGINVK(ac)YDNPR_	6	-0.13601	56.951	8029500	8896400
>WP_013240730.1 MULTISPECIES: ATP-dependent metallopeptidase FtsH/Yme1/Tma family protein	K	LEDEIVGLLGGRVAEKLIIGDISTGAKNDIE	_VAEK(ac)LIIGDISTGAK_	4	-0.77141	78.285	0	5775500
>WP_013240730.1 MULTISPECIES: ATP-dependent metallopeptidase FtsH/Yme1/Tma family protein	K	KEIRKLIDESYDKAEKLLSDNLNKLHAVAKQ	_AEK(ac)LLSDNLNK_	3	0.21778	62.408	5517500	5264500
>WP_013240730.1 MULTISPECIES: ATP-dependent metallopeptidase FtsH/Yme1/Tma family protein	K	ILVGAPDVKGREEILKVHSKNKHLSDEVKLD	_GREEILK(ac)VHSK_	7	0.30588	66.498	5526200	5967600
>WP_013240730.1 MULTISPECIES: ATP-dependent metallopeptidase FtsH/Yme1/Tma family protein	K	KNKHLSDEVKLDVLAKRTPGFTGADLENLMN	_LDVLAK(ac)R_	6	0.31366	135.35	21016000	22335000
>WP_013240739.1 MULTISPECIES: HU family DNA-binding protein	K	MNKSELIAGIAEKSKLTKKDVEAALKGF	_SELIAGIAEK(ac)SK_	10	0.36688	100.55	15532000	9889300
>WP_013240739.1 MULTISPECIES: HU family DNA-binding protein	K	RAERIGRNPRTKEEIKIPESTVPVFKAGKEF	_TKEEIK(ac)IPESTVPVFK_	6	0.08841	91.789	86359000	70349000
>WP_013240739.1 MULTISPECIES: HU family DNA-binding protein	K	AALKGFIESVEETLEKGDKVQLVGFGTFETR	_GFIESVEETLEK(ac)GDK_	12	-0.84793	170.52	95071000	57649000
>WP_013240739.1 MULTISPECIES: HU family DNA-binding protein	K	TKEEIKIPESTVPVFKAGKEFKDRVNK	_IPESTVPVFK(ac)AGK_	10	-0.28532	122.39	603890000	563010000
>WP_013240740.1 MULTISPECIES: nucleoside triphosphate pyrophosphohydrolase	K	GINFETYDHLYEKKQKFEDVYRSIAEDLILK	_QK(ac)FEDVYR_	2	-0.3719	82.749	9374600	4228500

>WP_013240748.1 MULTISPECIES: DNA-binding response regulator	K	GYDYPGDSRTVDVHVKRLR EKLQGGPNWQIE	_TVDVHVK(ac)R_	7	0.07231	159.04	88974000	66428000
>WP_013240749.1 MULTISPECIES: ribose-phosphate pyrophosphokinase	K	RKFADKLNASIAIIDKRRPKA NVSEVMNIIG	_LNASIAIIDK(ac)R_	10	-4.8023	65.395	3910600	0
>WP_013240749.1 MULTISPECIES: ribose-phosphate pyrophosphokinase	K	SPDLGSVTRARKFADKLNAS IAIIDKRRPKA	_FADK(ac)LNASIAIID KR_	4	-0.00255	63.894	4805400	0
>WP_013240749.1 MULTISPECIES: ribose-phosphate pyrophosphokinase	K	MITHGKNIKIFAGNSHPELAK QIA	_NIK(ac)IFAGNSHPEL AK_	3	-0.00428	106.52	55071000	39928000
>WP_013240749.1 MULTISPECIES: ribose-phosphate pyrophosphokinase	K	KFKILSIAPVFAEAIKRIYEDV SVSKLFED_	_ILSIAPVFAEAIK(ac) R_	13	-0.12636	182.07	554860000	389520000
>WP_013240750.1 bifunctional UDP-N-acetylglucosamine diphosphorylase/glucosamine-1-phosphate N-acetyltransferase GlmU	K	STIGKSVRIGDFVEVKKSTIG DKTKVSHLTY	_IGDFVEVK(ac)K_	8	0.39161	86.624	228650000	188090000
>WP_013240751.1 MULTISPECIES: septation protein SpoVG	K	VIEGQNGLFIAMPSRKTPDGE FKDIAHPINT	_K(ac)TPDGEFK(ac)DI AHPINTQTR_	1	-0.03648	31.462	0	4681000
>WP_013240751.1 MULTISPECIES: septation protein SpoVG	K	DIAHPINTQTREKIQKAILDEY EKVKSEETV	_EKIQK(ac)AILDEYE K_	5	0.42476	85.457	11994000	9381000
>WP_013240751.1 MULTISPECIES: septation protein SpoVG	K	DEYEKVKSEETVTEEKVKE	_SEETVTEEK(ac)VK_	9	0.05345	167.3	83771000	84822000
>WP_013240751.1 MULTISPECIES: septation protein SpoVG	K	QTREKIQKAILDEYEKVKSEE TVTEEKVKE_	_AILDEYEK(ac)VK_	8	-0.11046	98.048	88542000	39313000
>WP_013240751.1 MULTISPECIES: septation protein SpoVG	K	LFIAMPSRKTPDGEFKDIAHP INTQTREKIQ	_TPDGEFK(ac)DIAHPI NTQTR_	7	0.14164	250.1	1.127E+09	913380000
>WP_013240753.1 MULTISPECIES: UDP-N-acetylmuramate--L-alanine ligase	K	VIYTAAIQKNNEEFLKAKTLNI PLMSRAELL	_NNEEFLK(ac)AK_	7	0.37094	85.731	18075000	18487000
>WP_013240753.1 MULTISPECIES: UDP-N-acetylmuramate--L-alanine ligase	K	EIAKRGIKSTNISDFKSIVDYL KSNLKDGDL	_STNISDFK(ac)SIVDY LK_	8	-0.26003	61.732	19103000	11924000
>WP_013240753.1 MULTISPECIES: UDP-N-acetylmuramate--L-alanine ligase	K	TEIKATLNAAINYPHKRIICVF QPHTFSRTI	_ATLNAAINYPHK(ac) R_	12	-0.48572	69.602	23007000	21492000
>WP_013240764.1 MULTISPECIES: helix-turn-helix domain-containing protein	K	KLGVSEKFINEAESGKRIVNQ NIIDKLSKIL	_FINEAESGK(ac)R_	9	1.0979	73.067	0	9923900
>WP_013240764.1 MULTISPECIES: helix-turn-helix domain-containing protein	K	GQKIKLARTEMGISQKQLAKK LGVSEKFINE	_TEMGISQK(ac)QLAK _	8	0.11058	34.96	1943200	0
>WP_013240764.1 MULTISPECIES: helix-turn-helix domain-containing protein	K	IRTETIEIKSLKIIAKLDRLEIKL	_IIAK(ac)LDR_	4	-0.14535	104.45	26679000	18232000
>WP_013240764.1 MULTISPECIES: helix-turn-helix domain-containing protein	K	LISSRSGIRTETIEIKSLKIIAKL DRLEIKL	_TETIEIK(ac)SLK_	7	-0.82215	93.096	48098000	19619000
>WP_013240764.1 MULTISPECIES: helix-turn-helix domain-containing protein	K	GISQKQLAKKLGVSEKFINEA ESGKRIVNQN	_LGVSEK(ac)FINEAE SGKR_	6	-0.58303	176.4	77827000	42243000
>WP_013240765.1 MULTISPECIES: glutamate racemase	K	DSKMAPYGVKTAEEVKRLTF NAVELLLTKNI	_TAEEVK(ac)R_	6	0.49429	78.496	7227300	0
>WP_013240765.1 MULTISPECIES: glutamate racemase	K	DIPIIDGSKGTVEQLKRQLVK YHIKNNKNKA	_GTVEQLK(ac)R_	7	-0.36903	110.81	45368000	36000000
>WP_013240765.1 MULTISPECIES: glutamate racemase	K	SMDSEFIIQLSYKLLKEGSI	_LLK(ac)EGSI_	3	0.68304	83.801	1.042E+09	1.213E+09
>WP_013240766.1 MULTISPECIES: glutamine synthetase	K	YLAMMDGIKYAVTNEKTEDD LLKELSKKAED	_YAVTNEK(ac)TEDDL LK_	7	-0.12625	32.734	2826400	0
>WP_013240766.1 MULTISPECIES: glutamine synthetase	K	KELSKKAEDDADYLEKGRA YRSEENVFESFT	_KAEDDADYLEK(ac) GR_	11	0.1889	98.943	3389500	0
>WP_013240766.1 MULTISPECIES: glutamine synthetase	K	IKYAVTNEKTEDDLLKELSKK AEDDADYLEK	_TEDDLLK(ac)ELSK_	7	-0.2054	69.864	30837000	20077000
>WP_013240766.1 MULTISPECIES: glutamine synthetase	K	LVYVVPKETHSEENLKKMLT AHSEIKFVSLI	_ETHSEENLK(ac)K_	9	0.58891	59.153	54812000	37902000
>WP_013240766.1 MULTISPECIES: glutamine synthetase	K	KNCVEHFKSNLLDLFKKYPS TISSFGIKYED	_SNLLDLFK(ac)K_	8	-0.01709	79.974	96471000	73451000

>WP_013240769.1 MULTISPECIES: UDP-N-acetylglucosamine 1-carboxyvinyltransferase	K	NESIWESRFKHVDELKKMGANIKVEGRTAII	_HVDELK(ac)K_	6	0.30798	94.262	6183300	5219700
>WP_013240772.1 MULTISPECIES: hypothetical protein	K	SNQKRREIHKQWVSSKTIVNKTKVGRNDPCP	_QWVSSK(ac)TIVNK_	6	0.5016	56.225	12059000	10224000
>WP_013240774.1 MULTISPECIES: asparagine--tRNA ligase	K	DLQTEHERYLTEKVFKKPIFVTDYPKAIKAF	_VFK(ac)KPIFVTDYPK_	3	0.39639	60.628	0	5045600
>WP_013240774.1 MULTISPECIES: asparagine--tRNA ligase	K	LQTEHERYLTEKVFKKPIFVTDYPKAIKAFY	_VFK(ac)KPIFVTDYPK_	4	0.5275	33.685	0	5045600
>WP_013240774.1 MULTISPECIES: asparagine--tRNA ligase	K	NDGSFFKNIQIVFDEKLPNFKELTKLAISSS	_NIQIVFDEK(ac)LPNFK_	9	0.87382	58.487	3677000	0
>WP_013240774.1 MULTISPECIES: asparagine--tRNA ligase	K	FKNIQIVFDEKLPNFKELTKLAISSSLSIEG	_LPNFK(ac)ELTK_	5	-0.31997	86.898	4285900	9115000
>WP_013240774.1 MULTISPECIES: asparagine--tRNA ligase	K	SEFGRITYTEAVDILKKSGASFEYPVEWGID	_ITYTEAVDILK(ac)K_	11	0.18082	95.018	32645000	26661000
>WP_013240774.1 MULTISPECIES: asparagine--tRNA ligase	K	EITLEGNSSSDYPLQKKRHTLEYLRTIAHLR	_EITLEGNSSSDYPLQK(ac)K_	16	-0.44718	151.9	44748000	31391000
>WP_013240774.1 MULTISPECIES: asparagine--tRNA ligase	K	WGIDLQTEHERYLTEKVFKKPIFVTDYPKAI	_YLTEK(ac)VFK_	5	0.06933	75.738	62341000	60133000
>WP_013240775.1 MULTISPECIES: CopG family transcriptional regulator	K	DGTSDEISSMTGKLGKLDGITVKTAITKK	_LGK(ac)LDGITVK_	3	0.42902	55.401	6680700	5418900
>WP_013240775.1 MULTISPECIES: CopG family transcriptional regulator	K	SSMTGKLGKLDGITVKTAITKK	_LDGITVK(ac)TAITK_	7	0.37095	68.809	18780000	16644000
>WP_013240780.1 MULTISPECIES: dihydrofolate reductase	K	DQVDIRRFNMIYDNLKRYASGDKLVNIVDIN	_FNMIYDNLK(ac)R_	9	0.906	65.627	11508000	6359700
>WP_013240780.1 MULTISPECIES: dihydrofolate reductase	K	GVYIVNIARGSILDEKSLLENIKNGKIAGAA	_GSILDEK(ac)SLLENIK_	7	0.77469	75.669	14704000	10165000
>WP_013240780.1 MULTISPECIES: dihydrofolate reductase	K	EWIVLKILEMLKHSQKFYEKQKNKIWKMDMG	_HSQK(ac)FYEK_	4	-0.09769	161.76	75908000	80659000
>WP_013240784.1 MULTISPECIES: 3-hydroxyacyl-[acyl-carrier-protein] dehydratase FabZ	K	KGPAGIGKAVAYVEGKKAAEGELLFMIGDKN	_AVAYVEGK(ac)K_	8	0.5019	92.062	21411000	15412000
>WP_013240784.1 MULTISPECIES: 3-hydroxyacyl-[acyl-carrier-protein] dehydratase FabZ	K	KVVPGDTLKLEAEIVKIKGPAGIGKAVAYVE	_LEAEIVK(ac)IK_	7	0.2147	115.71	51328000	39955000
>WP_013240786.1 MULTISPECIES: beta-ketoacyl-[acyl-carrier-protein] synthase II	K	GGIGTIEKQHSVLEEKGPNRITPLFIPMIIS	_QHSVLEEK(ac)GPNR_	8	0.56547	208.56	98041000	73365000
>WP_013240787.1 MULTISPECIES: 3-oxoacyl-[acyl-carrier-protein] reductase	K	LSDKVKDTTLNNIPLKKFGNAEDVANVVAFL	_DTTLNNIPLK(ac)K_	10	0.01855	57.348	6164700	5533700
>WP_013240787.1 MULTISPECIES: 3-oxoacyl-[acyl-carrier-protein] reductase	K	KGAFNCIRHVSPIMVKQRSGKIISISSVVGV	_HVSPIMVK(ac)QR_	8	-0.29525	134.25	61666000	61547000
>WP_013240788.1 MULTISPECIES: [acyl-carrier-protein] S-malonyltransferase	K	NVQDIKSLNSTVEELKNR	_SLNSTVEELK(ac)NR_	10	1.8676	85.288	7365300	16011000
>WP_013240790.1 MULTISPECIES: ketoacyl-ACP synthase III	K	IIPHQANYRIIECAAKKLGIGMDRFYINLDA	_IIECAAK(ac)K_	7	0.4263	73.931	23139000	14609000
>WP_013240792.1 MULTISPECIES: nitronate monooxygenase	K	AGGGIYTGADIAKFFKLGVDAVQMGTRFVAT	_FFK(ac)LGVDAVQMGTR_	3	1.1119	83.729	16499000	17295000
>WP_013240792.1 MULTISPECIES: nitronate monooxygenase	K	MKLPPLIIGDLKAKIPIIQGGMGVGIS	_LPPLIIGDLK(ac)AK_	10	0.15637	130.01	21861000	28734000
>WP_013240797.1 MULTISPECIES: NAD(P)/FAD-dependent oxidoreductase	K	FGYRSIFDSFIGVINKKIIPILLKESSVEDI	_SIFDSFIGVINK(ac)K_	12	0.97427	76.655	6606000	10653000
>WP_013240799.1 MULTISPECIES: adenylosuccinate synthase	K	FQGGNNAGHTVEVGDKQYKLHLVPSGILYKD	_FQGGNNAGHTVEVGDK(ac)QYK_	16	-0.16267	88.133	26153000	26616000
>WP_013240800.1 MULTISPECIES: FAD-dependent oxidoreductase	K	TEPFNQPITYGKYIAKLGNMLTGNGIMVQRL	_YIAK(ac)LGNMLTGNGIMVQR_	4	0.74579	51.147	11453000	7513700
>WP_013240800.1 MULTISPECIES: FAD-dependent oxidoreductase	K	KNTLLYGVEVKFYSSKFDTNDKFETPIKNLY	_FYSSK(ac)FDTNDK_	5	-1.2243	58.09	56307000	47788000
>WP_013240802.1 MULTISPECIES: [FeFe] hydrogenase H-cluster maturation GTPase HydF	K	ADIKDLDVEHMKKELKIPIVKVSALKRKGIL	_ELK(ac)IPIVK_	3	0.3021	58.815	0	3421100

>WP_013240802.1 MULTISPECIES: [FeFe] hydrogenase H-cluster maturation GTPase HydF	K	LDVEHMKKELKIPIVKVSALK RKGILELKNQ	_IPIVK(ac)VSALK_	5	-0.47352	51.092	1309400	31015000
>WP_013240805.1 MULTISPECIES: M18 family aminopeptidase	K	LKKFGFVEIKEEEALKIKKCG KYFVTKNQSA	_EEEALK(ac)IK_	6	0.34342	62.2	10743000	777240
>WP_013240805.1 MULTISPECIES: M18 family aminopeptidase	K	SPTSFHAVKNAISELKKFGFV EIKEEEALKI	_NAISELK(ac)K_	7	0.19044	81.594	20144000	18811000
>WP_013240812.1 MULTISPECIES: methyl-accepting chemotaxis protein	K	AGESGKGFAVVADEIKKLAD SSLDETRKIGK	_GFAVVADEIK(ac)K_	10	0.02666	62.303	391920000	377240000
>WP_013240829.1 MULTISPECIES: nitrogen regulatory PII	K	MGNKLKKVDIITHKAKLEELR EALNDIGV	_VDIITHK(ac)AK_	7	1.6764	110.39	23553000	16856000
>WP_013240835.1 MULTISPECIES: ATP-dependent protease, Lon family	K	KTTAARIALEEAKKLKFTPFR KDAKFVEVDG	_LK(ac)FTPFR_	2	0.12725	94.262	14153000	15784000
>WP_013240835.1 MULTISPECIES: ATP-dependent protease, Lon family	K	ISDLNDKVKISVEDLKKVISIS RFVPFEEVP	_ISVEDLK(ac)K_	7	0.02036	93.551	15865000	12758000
>WP_013240836.1 MULTISPECIES: 50S ribosomal protein L9	K	EAERKEKLAEIEAAQKLAAS LKGKEIKLTVK	_LAEIEAAQK(ac)LAA SLK_	9	0.83863	98.694	6308400	4265100
>WP_013240836.1 MULTISPECIES: 50S ribosomal protein L9	K	LFGSITGKDISDELNKKFHINV DKKKIAVNN	_DISDELNK(ac)K_	8	0.03479	56.916	7922500	4351000
>WP_013240836.1 MULTISPECIES: 50S ribosomal protein L9	K	DISDELNKKFHINVDKKKIAV NNIRQLGTYD	_KFHINVDK(ac)K_	8	0.45697	96.342	15035000	5345400
>WP_013240836.1 MULTISPECIES: 50S ribosomal protein L9	K	MKVILLKDVKTLGKKGQVINA S	_VILLK(ac)DVK_	5	-1.3881	79.474	19050000	10967000
>WP_013240836.1 MULTISPECIES: 50S ribosomal protein L9	K	GTYDVEVKLYPEISTKIKVVI AEK	_LYPEISTK(ac)IK_	8	0.12661	90.108	21156000	21354000
>WP_013240837.1 MULTISPECIES: delta-lactam-biosynthetic de-N-acetylase	K	FSSILEGKDILGQNIKNVVRDF NVKQVANGK	_DILGQNIK(ac)NVVR DFNVK_	8	-0.80627	40.801	22728000	32369000
>WP_013240840.1 MULTISPECIES: 30S ribosomal protein S18	K	SGNCAKHQRELTVAIKRARNI ALLPFTTE	_ELTVAIK(ac)R_	7	-0.02462	100.19	8773400	7115600
>WP_013240842.1 MULTISPECIES: 30S ribosomal protein S6	K	LQPSLDEEACKANIEKFKGVI ENGGGVIENV	_ANIEK(ac)FK_	5	-0.08232	84.097	444010000	174800000
>WP_013240843.1 MULTISPECIES: DUF951 domain-containing protein	K	CGSNEWEVIRLGADIKIKCCG CGRIVMLPRN	_LGADIK(ac)IK_	6	0.25092	68.224	7148000	7031100
>WP_013240843.1 MULTISPECIES: DUF951 domain-containing protein	K	MKKVYYIGDVVEMKKGHPC GSNEWEVIRL	_VYYIGDVVEMK(ac) K_	11	-0.54902	87.363	34490000	20011000
>WP_013240850.1 MULTISPECIES: chromosome partitioning protein ParB	K	DAYSIVAGERRWRAAKLANI KEVPAIIMNLS	_AAK(ac)LANIK_	3	0.32615	68.787	7783700	8566300
>WP_013240851.1 MULTISPECIES: ParA family protein	K	AEVELINKENRENILKNNLKK LKAKFDFIFI	_ENILK(ac)NNLK_	5	-0.13508	66.27	4598100	12506000
>WP_013240851.1 MULTISPECIES: ParA family protein	K	CDGRTNLSTQVISEVKKYFK DKVYKSTIPRN	_TNLSTQVISEVK(ac) K_	12	-0.19672	106.13	27967000	23657000
>WP_013240853.1 MULTISPECIES: 16S rRNA (guanine(527)-N(7))-methyltransferase RsmG	K	IKIENDDLNHNLVVIKKIRRTP NMYPRKAGI	_IENDDLNHNLVVIK(a c)K_	14	0.44368	54.898	14282000	18220000
>WP_013240853.1 MULTISPECIES: 16S rRNA (guanine(527)-N(7))-methyltransferase RsmG	K	IVNPQIKVVLLDSLNKRINFLN QVISDIELN	_VVLLDSLNK(ac)R_	9	0.69642	171.56	31272000	12772000
>WP_013240854.1 MULTISPECIES: tRNA uridine-5-carboxymethylaminomethyl(34) synthesis enzyme MnmG	K	KEVVDFLNSIGSSELKKTTSL YELIKRPELD	_KEVVDFLNSIGSSEL K(ac)K_	16	0.0257	56.851	0	4431000
>WP_013240854.1 MULTISPECIES: tRNA uridine-5-carboxymethylaminomethyl(34) synthesis enzyme MnmG	K	INAALKSQGNPPLILKRSDGY IGVLVDDLVT	_SQGNPPLILK(ac)R_	10	0.00761	49.5	6316400	9516300
>WP_013240854.1 MULTISPECIES: tRNA uridine-5-carboxymethylaminomethyl(34) synthesis enzyme MnmG	K	INYDKINGLRIEATQKLKKIRP MSVGQASRI	_IEATQK(ac)LK_	6	0.31082	93.374	24348000	21194000
>WP_013240856.1 MULTISPECIES: protein jag	K	ARRVAEKVKRTGRIIKLEPM NPYERRIIHSA	_IIK(ac)LEPMNPYER _	3	-0.17698	54.7	3690100	0

>WP_013240856.1 MULTISPECIES: protein jag	K	MDFIETTGKTVEDAFKTALSELKVEEDKVEM	_TVEDAFK(ac)TALSELK_	7	0.39307	40.025	8501200	10874000
>WP_023161661.1 MULTISPECIES: serine dehydratase subunit alpha family protein	K	AKHGLVFPFGEGLVEKDYEKTIQNIGRVGHQ	_HGLVFPFGEGLVEK(ac)DYEK_	14	0.31253	96.163	42047000	37084000
>WP_023161684.1 MULTISPECIES: ketopantoate reductase family protein	K	TEVDIFAGTVCKLGEKYGVDTPVNRTLLNII	_LGEK(ac)YGVDTPVNR_	4	0.22634	114.72	21830000	17634000
>WP_023161837.1 MULTISPECIES: nitrate reductase	K	FINTERRLSAVTPILKKEKDELTDYDILLGI	_LSAVTPILK(ac)K_	9	0.07029	93.096	9437700	12272000
>WP_023161837.1 MULTISPECIES: nitrate reductase	K	GFKEHVKKYTLENVEKRTGISSKRVMELAEI	_YTLENVEK(ac)R_	8	-0.49843	113.22	10251000	34021000
>WP_023161837.1 MULTISPECIES: nitrate reductase	K	DREYIEKYSEGFEGFKEHVKKYTLENVEKRT	_YSEGFEGFK(ac)EHVK_	9	0.21552	74.475	21236000	27071000
>WP_023162006.1 MULTISPECIES: 3-deoxy-7-phosphoheptulonate synthase	K	SVESEEQIVKIAQDVKKSGAKFLRGGAFKPR	_IAQDVK(ac)K_	6	0.76153	88.029	0	56406000
>WP_023162006.1 MULTISPECIES: 3-deoxy-7-phosphoheptulonate synthase	K	VIQVGARNMQNFELLKQLGKTNKPILLKRGL	_NMQNFELLK(ac)QLGK_	9	-0.22764	103.75	8008000	3894600
>WP_023162006.1 MULTISPECIES: 3-deoxy-7-phosphoheptulonate synthase	K	EQIVKIAQDVKKSGAKFLRGGAFKPRTSPYS	_SGAK(ac)FLR_	4	0.46567	101.69	27823000	11593000
>WP_023162006.1 MULTISPECIES: 3-deoxy-7-phosphoheptulonate synthase	K	TYTRNTLDLSAIPAIKRLSHLPVIVDPSHAA	_NTLDLSAIPAIK(ac)R_	12	0.86597	113.24	35985000	34820000
>WP_023162006.1 MULTISPECIES: 3-deoxy-7-phosphoheptulonate synthase	K	DVKKSGAKFLRGGAFKPRTSPYSFQGLRTRG	_GGAFK(ac)PR_	5	0.53044	74.335	36908000	40001000
>WP_023162006.1 MULTISPECIES: 3-deoxy-7-phosphoheptulonate synthase	K	SIKPKVFEQLMNDISKIPGKTIHKAYDTVNV	_VFEQLMNDISK(ac)IPGK_	11	-0.34559	73.887	37580000	33855000
>WP_023162006.1 MULTISPECIES: 3-deoxy-7-phosphoheptulonate synthase	K	PYSFQGLRTRGLDLLKIARQETGLPIVTEIM	_GLDLLK(ac)IAR_	6	0.14718	126.41	56958000	43050000
>WP_023162006.1 MULTISPECIES: 3-deoxy-7-phosphoheptulonate synthase	K	DADKIRANEFVENVEKVQQPYKLSNRLFHPD	_ANEFVENVEK(ac)VQQPYK_	10	0.83285	179.17	358630000	162990000
>WP_023162006.1 MULTISPECIES: 3-deoxy-7-phosphoheptulonate synthase	K	YKLSNRLFHPDDTVIKVKNASIGGDELAVIA	_LFHPDDTVIK(ac)VK_	10	0.01173	135.6	580260000	577680000
>WP_023162298.1 MULTISPECIES: rRNA pseudouridine synthase	K	SDEDGAKVEVTIQEGKFHQVKRMFNALGKNV	_VEVTIQEGK(ac)FHQVK_	9	-0.03569	62.14	15051000	7472000
>WP_023162486.1 MULTISPECIES: 50S ribosomal protein L30	K	MAKLKITLEKSLIGRKKDHIATVNA	_ITLEK(ac)SLIGR_	5	0.77184	81.865	7247300	0
>WP_023162488.1 MULTISPECIES: transcription elongation factor GreA	K	TYEGITKLEDELEYLKTAKRREIMEKIKVAL	_LEDELEYLK(ac)TAK_	9	-1.6331	80.469	17012000	12195000
>WP_023163007.1 MULTISPECIES: homoserine dehydrogenase	K	EGITKIKSIDIRYAKKFNMVIKLLAIAKENE	_K(ac)FNMVIK_	1	0.38897	97.602	30443000	30002000
>WP_023163064.1 MULTISPECIES: hypothetical protein	K	IYNEIKKDSQVKFSDKFLENTLKNIKGNSTY	_FSDK(ac)FLENTLK_	4	0.00358	161.99	29320000	34218000
>WP_029169889.1 MULTISPECIES: helicase-exonuclease AddAB subunit AddA	K	LFHEKLGLGPDYVNVKRHICYPTVMKQVLRK	_LGLGPDYVNVK(ac)R_	11	-0.10346	79.82	9877600	11396000
>WP_029170000.1 MULTISPECIES: MerR family transcriptional regulator	K	TFERSKIEDNILHLEKKINTYKKMIAKTRVL	_SKIEDNILHLEK(ac)K_	12	0.10735	77.527	7061800	6453000
>WP_029170021.1 MULTISPECIES: RNA polymerase sigma factor RpoD	K	GKVPLLLPEEEISLAKRIEEGDQIAKKKLAE	_VPLLLPEEEISLAK(ac)R_	14	-0.2264	80.316	34575000	28323000
>WP_029170021.1 MULTISPECIES: RNA polymerase sigma factor RpoD	K	ARTIRIPVHMVETINKLVRVSRQLLQELGRE	_IPVHMVETINK(ac)LVR_	11	-0.2465	91.62	56900000	34718000
>WP_029170021.1 MULTISPECIES: RNA polymerase sigma factor RpoD	K	MAKGGNKMKSKSAKLQLVKKIIEKGKKNG	_SAK(ac)LQLVK_	3	0.30187	119.21	57595000	26165000
>WP_029170054.1 MULTISPECIES: cell division protein FtsA	K	MAAINGIEKFGENVLKKLVRIGIPKYIGAAS	_FGENVLK(ac)K_	7	0.49173	122.18	21351000	24722000
>WP_029170094.1 MULTISPECIES: phospho-N-acetylmuramoyl-pentapeptide-transferase	K	AILEGPILIPLLHKFKFGQNIREDGPKSHLK	_FK(ac)FGQNIR_	2	-0.23518	66.073	5979400	19735000
>WP_029170100.1 MULTISPECIES: NAD-dependent dehydratase	K	KARDLINWEPEHTLEKGIGETERWIEGTKLL	_DLINWEPEHTLEK(ac)GIGETER_	13	-0.4266	76.768	9438300	7575300
>WP_029170100.1 MULTISPECIES: NAD-dependent dehydratase	K	FDNLSNGRIENLQEFKNNSGFKFIGGDVRDE	_IENLQEFK(ac)NNSGFK_	8	-4.1613	85.457	9924200	2415800
>WP_029170128.1 MULTISPECIES: elongation factor P	K	FEQIPLEYEKVEDAIKFLKENMFAIIKFYKG	_VEDAIK(ac)FLK_	6	-0.45103	48.091	6874400	1953100

>WP_029170128.1 MULTISPECIES: elongation factor P	K	VITGSVTDTTFNPTAKLQEAVIERKEMQYLY	_NVITGSVTDTTFNPTAK(ac)LQEAVIER_	17	0.46659	72.532	97469000	37535000
>WP_029170202.1 MULTISPECIES: GTP cyclohydrolase I FolE	K	KPGSKTVTAVARGLFKSEPRLRDEVYRMINM	_GLFK(ac)SEPR_	4	-0.73974	72.547	7120100	0
>WP_029170202.1 MULTISPECIES: GTP cyclohydrolase I FolE	K	AHIAYIPDGKVVGLSKLARTVEVFAKRLQLQ	_VVGLSK(ac)LAR_	6	0.41437	98.033	29708000	26772000
>WP_029170221.1 MULTISPECIES: Fe-S cluster assembly scaffold protein NifU	K	GDIMKIYIKVEDDVIKDIKFKTFGCGSAIAS	_VEDDVIK(ac)DIK_	7	0.19259	94.616	46326000	31178000
>WP_029170221.1 MULTISPECIES: Fe-S cluster assembly scaffold protein NifU	K	PVKMHCSVLAEEAIHKAINDYRESQGLEPWA	_MHCSVLAEEAIHK(ac)AINDYR_	13	0.19132	123.41	166450000	75408000
>WP_029702388.1 MULTISPECIES: sensor histidine kinase	K	LSQSLLKIAGQVHEIKKDNQRIYSGLSKMIL	_IAGQVHEIK(ac)K_	9	-0.02737	97.813	16214000	12543000
>WP_041705024.1 MULTISPECIES: transcriptional regulator	K	QQLLLSANVVFIKNNKYEEIIKTFISLGKIT	_NNK(ac)YEEIIK_	3	-2.1643	54.066	4477300	13465000
>WP_041705024.1 MULTISPECIES: transcriptional regulator	K	MNYTKEAADISIKLGKFYEDSKMDKEAAKYL	_LGK(ac)FYEDSK_	3	0.09488	119.62	19604000	26005000
>WP_041705054.1 bifunctional 2-keto-4-hydroxyglutarate aldolase/2-keto-3-deoxy-6-phosphogluconate aldolase	K	IIKIFPGSVFGPQIIKAFKGPIPQGNFMPTG	_IFPGSVFGPQIIK(ac)AFK_	13	2.7287	84.842	30766000	13964000
>WP_041705054.1 bifunctional 2-keto-4-hydroxyglutarate aldolase/2-keto-3-deoxy-6-phosphogluconate aldolase	K	KGAKTGDYELVTETAKKFVQAVKEAKEGKYN	_TGDYELVTETAK(ac)K_	12	-0.40811	132.79	41535000	19780000
>WP_041705054.1 bifunctional 2-keto-4-hydroxyglutarate aldolase/2-keto-3-deoxy-6-phosphogluconate aldolase	K	MNRVRTIMKLAQQGVVAVIRADSK	_TIMK(ac)LAQQGVVAVIR_	4	0.47403	186.13	88518000	41969000
>WP_041705054.1 bifunctional 2-keto-4-hydroxyglutarate aldolase/2-keto-3-deoxy-6-phosphogluconate aldolase	K	VKEAVEGLEAGAEIIKIFPGSVFGPQIIKAF	_EAVEGLEAGAEIIK(ac)IFPGSVFGPQIIK_	14	-0.55799	152.96	95365000	145110000
>WP_041705109.1 FprA family A-type flavoprotein	K	VIMGPFKKFVLMGLDKIKNLDFDMVGPSHGP	_FVLMGLDK(ac)IK_	8	-0.58085	63.283	6618800	5193400
>WP_041705109.1 FprA family A-type flavoprotein	K	DMVGPSHGPVHIDDIKKSIESYRKWALTESK	_NLDFDM(ox)VGPSHGPVHIDDIK(ac)K_	20	-2.7525	98.269	93615000	75643000
>WP_041705189.1 single-stranded DNA-binding protein	K	VGTDVIIEGQLRSYNKFVDGANRLILTVFAR	_SYNK(ac)FVDGANR_	4	0.20372	117.25	30891000	27152000
>WP_041705601.1 MULTISPECIES: glutamate synthase (NADPH), homotetrameric	K	SNLMKAYRDDYATPIKAGKKVAVVGGGNVAM	_DDYATPIK(ac)AGK_	8	0.01848	75.229	10270000	14597000
>WP_041705601.1 MULTISPECIES: glutamate synthase (NADPH), homotetrameric	K	ILGDEKGWVKGIRCIKMELGEPDASGRRKPV	_CIK(ac)MELGEPDASGR_	3	0.27366	101.62	10970000	2.768E+09
>WP_041705601.1 MULTISPECIES: glutamate synthase (NADPH), homotetrameric	K	GKCVLGKKGDAVAIGKLERFVADWSRKNNID	_KGDAVAIGK(ac)LER_	9	-0.33143	113.24	13125000	14291000
>WP_041705601.1 MULTISPECIES: glutamate synthase (NADPH), homotetrameric	K	MGAGKKAAKAIDEYLKK	_AIDEYLK(ac)K_	7	0.75287	89.296	18970000	6545300
>WP_041705601.1 MULTISPECIES: glutamate synthase (NADPH), homotetrameric	K	RFVADWSRKNNIDLSKTLPKNGKKVAVIGSG	_NNIDLSK(ac)TLPK_	7	0.32142	153.39	32428000	32238000
>WP_041705601.1 MULTISPECIES: glutamate synthase (NADPH), homotetrameric	K	DVIIGRTVTIDELIEKEKFDAVFIGSGAGLP	_TVTIDELIEK(ac)EK_	10	0.46142	117.23	41550000	33906000
>WP_041705601.1 MULTISPECIES: glutamate synthase (NADPH), homotetrameric	K	RLPKDSVVKHEVENVKKLGVKIETDVIIGRT	_HEVENVK(ac)K_	7	0.22891	159.28	121260000	126600000
>WP_041705601.1 MULTISPECIES: glutamate synthase (NADPH), homotetrameric	K	IIGRTVTIDELIEKEKFDAVFIGSGAGLPRF	_EK(ac)FDAVFIGSGAGLPR_	2	2.0223	160.81	141190000	130510000
>WP_041705601.1 MULTISPECIES: glutamate synthase (NADPH), homotetrameric	K	PNPLISTTTKGLEMNKRKCLIAEEETGLTTR	_GLEM(ox)NK(ac)R_	6	1.3677	113.25	202560000	180380000

>WP_041705614.1 MULTISPECIES: proline--tRNA ligase	K	KGVPIRLEVGPKDIEKNQVVLVRRDSGEKII	_DIEK(ac)NQVVLVR_	4	1.142	70.977	1566000	0
>WP_041705614.1 MULTISPECIES: proline--tRNA ligase	K	ETLFCEHYAKIVQSYKDLPKLYNQWCSVVRW	_IVQSYK(ac)DLPK_	6	-1.0747	51.927	2763900	2881400
>WP_041705614.1 MULTISPECIES: proline--tRNA ligase	K	VSMDNLEKDIPDLLDKIHDGMFEKAKNLIEN	_DIPDLLDK(ac)IHDGMFEK_	8	-0.03908	37.555	2805300	0
>WP_041705614.1 MULTISPECIES: proline--tRNA ligase	K	LIENNTNNAVNMEEFKDIMENKIGFVKAMWC	_NLIENNTNNAVNMEEFK(ac)DIMENK_	17	-0.25331	39.086	14598000	13028000
>WP_041705614.1 MULTISPECIES: proline--tRNA ligase	K	TTEFLWQEGHTIHETKKEAQEETTRMLNVYA	_TTEFLWQEGHTIHETK(ac)K_	16	-0.52014	129.38	25434000	22136000
>WP_049781919.1 diaminopropionate ammonia-lyase	K	NLCEVKTFHESFEQYKKTPLVKLDGLANFLG	_TFHESFEQYK(ac)K_	10	0.22125	59.542	0	4221400
>WP_049781921.1 sugar-binding transcriptional regulator	K	HVVEITINKPHSRVPKLENILKENFDLKDAL	_VPK(ac)LENILK_	3	1.0137	79.659	24433000	29721000
>WP_049781921.1 sugar-binding transcriptional regulator	K	SAIRQANHVIGIAAGKFKAKAILGAIRGKYI	_QANHVIGIAAGK(ac)FK_	12	0.38401	164.48	177860000	177830000
>WP_063562606.1 hypothetical protein	K	IDFMKQADNMLEEAEKHME	_QADNMLEEAEK(ac)HME_	11	-0.50147	43.813	18204000	8309400
>WP_063562606.1 hypothetical protein	K	EHNKSHEEGFAEWVEKAKNMGKVETSQFIEK	_SHEEGFAEWVEK(ac)AK_	12	0.29741	135.37	21887000	14303000
>WP_081442034.1 hypothetical protein	K	NDIDLLKKQLKGNIQKLIDSGDLKDARQLID	_GNIQK(ac)LIDSGDLK_	5	-0.47576	40.025	6241500	5965600
>WP_081442057.1 MULTISPECIES: acetolactate synthase small subunit	K	DMNEKSMIIEITGNEKKISAFIELMKPYGIK	_SMIIEITGNEK(ac)K_	11	0.88672	35.204	3420200	0
>WP_081442060.1 MULTISPECIES: DUF2292 domain-containing protein	K	SITLVIQDGKVVQIEKSEKVRLV	_VVQIEK(ac)SEK_	6	-0.75057	65.347	6612900	30750000
>WP_081442103.1 hydrogenase	K	DFAGIDKNKAEEFIEKEERKYYEYLEDFSDF	_AEEFIEK(ac)EER_	7	0.28571	79.693	1113000	5567400
>WP_081442103.1 hydrogenase	K	LVLSPWLGLKTAEHLKKKYGQPYLHIPTIPI	_TAEHLK(ac)K_	6	0.27437	94.262	4545900	9895700
>WP_087943146.1 selenide, water dikinase SelD	K	EVKSSVIGEVIPIQVKKYIEVE	_SSVIGEVIPIQVK(ac)K_	13	0.26562	46.704	7344300	22030000
>WP_087943148.1 2-isopropylmalate synthase	K	AFINAMNKIHYENSLKKVEEC	_IHYENSLK(ac)K_	8	-0.20764	58.676	0	3922300
>WP_087943148.1 2-isopropylmalate synthase	K	TPNVSLNINDKLTIAKQLQKLSVDVIEAGFP	_LTIAK(ac)QLQK_	5	0.78123	58.981	2413100	17282000
>WP_087943148.1 2-isopropylmalate synthase	K	LGYKNLGKEKIDEIFKEFKDLADKKKHVSDE	_IDEIFK(ac)EFK_	6	0.39398	91.584	8384900	6121900
