# Supplementary material for: Interactive Regulation of Formate Dehydrogenase during CO2 Fixation in Gas-Fermenting Bacteria
Source: mBio. 2020 Aug 18;11(4):e00650-20. doi: 10.1128/mBio.00650-20 (PMC7439476; doi:10.1128/mBio.00650-20)
Supplement: TABLE S2 [file mBio.00650-20-st002.pdf]

Table S2 Strains and plasmids used in this study.

Strains or plasmids	Description of genotype	Source or reference
Bacterial strains		
C. ljungdahlii		
WT	DSM 13528, wild-type strain	DSMZ
Δat2	DSM 13528 Δ*at2*	This study
Δdat1	DSM 13528 Δ*dat1*	This study
ΔFDH1	DSM 13528 Δ*fdh1*	This study
ΔFDH2	DSM 13528 Δ*fdh2*	This study
ΔFDH3	DSM 13528 Δ*fdh3*	This study
ΔCcpA	DSM 13528 Δ*ccpA*	This study
$FDH1^{WT}$	ΔFDH1, carrying the pMTL83151-P_{fdh1}-*fdh1* plasmid	This study
$FDH1^{K29Q}$	ΔFDH1, carrying the pMTL83151-P_{fdh1}-$fdh1_{K29Q}$ plasmid	This study
$FDH1^{K29R}$	ΔFDH1, carrying the pMTL83151-P_{fdh1}-$fdh1_{K29R}$ plasmid	This study
$CcpA^{WT}$	ΔCcpA, carrying the pMTL83151-P_{ccpA}-*ccpA* plasmid	This study
$CcpA^{K56Q}$	ΔCcpA, carrying the pMTL83151-P_{ccpA}-$ccpA_{K56Q}$ plasmid	This study
$CcpA^{K56R}$	ΔCcpA, carrying the pMTL83151-P_{ccpA}-$ccpA_{K56R}$ plasmid	This study
E. coli		
DH5α	General cloning host strain	Invitrogen
BL21 (DE3)	Strain used for protein overexpression	Novagen
Plasmids		
pMTLcas-*at2*	*pCB102 ori, catP, ColE1,tra, Pthl-Cas9,* P*araE*-sgRNA, *at2* homologous arm	This study
pMTLcas-*dat1*	*pCB102 ori, catP, ColE1,tra, Pthl-Cas9,* P*araE*-sgRNA, *dat* homologous arm	This study
pMTLcas-*fdh1*	*pCB102 ori, catP, ColE1,tra, Pthl-Cas9,* P*araE*-sgRNA, *fdh1* homologous arm	This study
pMTLcas-*fdh2*	*pCB102 ori, catP, ColE1,tra, Pthl-Cas9,* P*araE*-sgRNA, *fdh2* homologous arm	This study
pMTLcas-*fdh3*	*pCB102 ori, catP, ColE1,tra, Pthl-Cas9,* P*araE*-sgRNA, *fdh3* homologous arm	This study
pMTLcas-*ccpA*	*pCB102 ori, catP, ColE1,tra, Pthl-Cas9,* P*araE*-sgRNA, *ccpA* homologous arm	This study
pET-28a	Vector used for protein purification	Invitrogen
pET-28a-At2	pET-28a carrying *at2* gene	This study
pET-28a-Dat1	pET-28a carrying *dat1* gene	This study
pET-28a-FDH1	pET-28a carrying *fdh1* gene	This study

pET-28a-FDH1^{K29Q}	pET-28a carrying *fdh1*$_{K29Q}$ gene	This study
pET-28a-FDH1^{K29R}	pET-28a carrying *fdh1*$_{K29R}$ gene	This study
pET-28a-FDH1^{K64Q}	pET-28a carrying *fdh1*$_{K64Q}$ gene	This study
pET-28a-FDH1^{K96Q}	pET-28a carrying *fdh1*$_{K96Q}$ gene	This study
pET-28a-FDH1^{K196Q}	pET-28a carrying *fdh1*$_{K196Q}$ gene	This study
pET-28a-FDH1^{K367Q}	pET-28a carrying *fdh1*$_{K367Q}$ gene	This study
pET-28a-FDH1^{K393Q}	pET-28a carrying *fdh1*$_{K393Q}$ gene	This study
pET-28a-FDH1^{K466Q}	pET-28a carrying *fdh1*$_{K466Q}$ gene	This study
pET-28a-FDH1^{K517Q}	pET-28a carrying *fdh1*$_{K517Q}$ gene	This study
pET-28a-FDH1^{K544Q}	pET-28a carrying *fdh1*$_{K544Q}$ gene	This study
pET-28a-FDH1^{K616Q}	pET-28a carrying *fdh1*$_{K616Q}$ gene	This study
pET-28a-FDH1^{K677Q}	pET-28a carrying *fdh1*$_{K677Q}$ gene	This study
pET-28a-FDH1^{K682Q}	pET-28a carrying *fdh1*$_{K682Q}$ gene	This study
pET-28a-FDH1^{K687Q}	pET-28a carrying *fdh1*$_{K687Q}$ gene	This study
pET-28a-FDH1^{K690Q}	pET-28a carrying *fdh1*$_{K690Q}$ gene	This study
pET-28a-FDH1^{K694Q}	pET-28a carrying *fdh1*$_{K694Q}$ gene	This study
pET-28a-FDH1^{K697Q}	pET-28a carrying *fdh1*$_{K697Q}$ gene	This study
pET-28a-FDH1^{K704Q}	pET-28a carrying *fdh1*$_{K704Q}$ gene	This study
pET-28a-FDH2	pET-28a carrying *fdh2* gene	This study
pET-28a-FDH2^{K46Q}	pET-28a carrying *fdh2*$_{K46Q}$ gene	This study
pET-28a-FDH2^{K57Q}	pET-28a carrying *fdh2*$_{K57Q}$ gene	This study
pET-28a-FDH2^{K92Q}	pET-28a carrying *fdh2*$_{K92Q}$ gene	This study
pET-28a-FDH2^{K259Q}	pET-28a carrying *fdh2*$_{K259Q}$ gene	This study
pET-28a-FDH2^{K270Q}	pET-28a carrying *fdh2*$_{K270Q}$ gene	This study
pET-28a-FDH2^{K275Q}	pET-28a carrying *fdh2*$_{K275Q}$ gene	This study
pET-28a-FDH2^{K363Q}	pET-28a carrying *fdh2*$_{K363Q}$ gene	This study
pET-28a-FDH2^{K678Q}	pET-28a carrying *fdh2*$_{K678Q}$ gene	This study
pET-28a-CcpA	pET-28a carrying *ccpA* gene	This study
pET-28a-CcpA^{K56Q}	pET-28a carrying *ccpA*$_{K56Q}$ gene	This study
pET-28a-CcpA^{K56R}	pET-28a carrying *ccpA*$_{K56R}$ gene	This study
pET-28a-CcpA^{K202Q}	pET-28a carrying *ccpA*$_{K202Q}$ gene	This study
pET-28a-CcpA^{K210Q}	pET-28a carrying *ccpA*$_{K210Q}$ gene	This study
pET-28a-CcpA^{K215Q}	pET-28a carrying *ccpA*$_{K215Q}$ gene	This study
pET-28a-CcpA^{K228Q}	pET-28a carrying *ccpA*$_{K228Q}$ gene	This study
pET-28a-CcpA^{K236Q}	pET-28a carrying *ccpA*$_{K236Q}$ gene	This study
pET-28a-CcpA^{K306Q}	pET-28a carrying *ccpA*$_{K306Q}$ gene	This study
pET-28a-CcpA^{K310Q}	pET-28a carrying *ccpA*$_{K310Q}$ gene	This study
pET-28a-CcpA^{K315Q}	pET-28a carrying *ccpA*$_{K315Q}$ gene	This study
pET-28a-CcpA^{K323Q}	pET-28a carrying *ccpA*$_{K323Q}$ gene	This study
pMTL83151	*ColE1, catP, pCB102 ori*	(*1*)
pMTL83151-P$_{fdh1}$-*fdh1*	P$_{fdh1}$-*fdh1* overexpression vector, derived from pMTL83151	This study
pMTL83151-P$_{fdh1}$-*fdh1*$_{K29Q}$	P$_{fdh1}$-*fdh1*$_{K29Q}$ overexpression vector, derived from pMTL83151	This study

pMTL83151-P_{fdh1}-*fdh1*$_{K29R}$	P_{fdh1}-*fdh1*$_{K29R}$ overexpression vector, derived from pMTL83151	This study
pMTL83151-$P_{fdh1\text{-}m}$-*fdh1*	$P_{fdh1\text{-}m}$-*fdh1* overexpression vector, derived from pMTL83151	This study
pMTL83151-$P_{fdh1\text{-}m}$-*fdh1*$_{K29R}$	$P_{fdh1\text{-}m}$-*fdh1*$_{K29R}$ overexpression vector, derived from pMTL83151	This study
pMTL83151-P_{ccpA}-*ccpA*	P_{ccpA}-*ccpA* overexpression vector, derived from pMTL83151	This study
pMTL83151-P_{ccpA}-*ccpA*$_{K56Q}$	P_{ccpA}-*ccpA*$_{K56Q}$ overexpression vector, derived from pMTL83151	This study
pMTL83151-P_{ccpA}-*ccpA*$_{K56R}$	P_{ccpA}-*ccpA*$_{K56R}$ overexpression vector, derived from pMTL83151	This study
pMTL83151-P_{1339}-*fdh1*	P_{1339}-*fdh1* overexpression vector, derived from pMTL83151	This study
pMTL83151-P_{1339}-*fdh1*$_{K56R}$	P_{1339}-*fdh1*$_{K56R}$ overexpression vector, derived from pMTL83151	This study
