# Supplementary material for: Interactive Regulation of Formate Dehydrogenase during CO2 Fixation in Gas-Fermenting Bacteria
Source: mBio. 2020 Aug 18;11(4):e00650-20. doi: 10.1128/mBio.00650-20 (PMC7439476; doi:10.1128/mBio.00650-20)
Supplement: TABLE S3 [file mBio.00650-20-st003.pdf]

Table S3 Primers used in this study.

Primer name	Sequence (5'-3')	Description
*dat1*gRNA-for	ACGCGTCGACGATTATGTACTAAATACCCC GTTTTAGAGCTAGAAATAGCAA	forward primer used for gene knock out
*dat1*gRNA-rev	AATTGATGAAAAAGATGAGAATAAAAATA AGAAGCCTGCA	reverse primer used for gene knock out
*dat1*UpArm-for	TGCAGGCTTCTTATTTTTATTCTCATCTTTT TCATCAATT	forward primer used for gene knock out
*dat1*UpArm-rev	TTAAAAAGAATAAAATCCATAAAATCACC TCTGATTAATA	reverse primer used for gene knock out
*dat1*DownArm-for	TATTAATCAGAGGTGATTTTATGGATTTTAT TCTTTTTAA	forward primer used for gene knock out
*dat1*DownArm-rev	CCGCTCGAGGGTTTGGCACCTCGATGTCG GCTCGTCGCAT	reverse primer used for gene knock out
*at2*gRNA-for	ACGCGTCGACATTGGAGAGGCGGAGATTA TGTTTTAGAGCTAGAAATAGCAA	forward primer used for gene knock out
*at2*gRNA-rev	AGAAGATAACCCTGATTTTTATAAAAATAA GAAGCCTGCAAAT	reverse primer used for gene knock out
*at2*UpArm-for	ATTTGCAGGCTTCTTATTTTTATAAAAATC AGGGTTATCTTCT	forward primer used for gene knock out
*at2*UpArm-rev	CCATCTCCCAACAACAACATTACAACACC TCCTAATATCATAA	reverse primer used for gene knock out
*at2*DownArm-for	TTATGATATTAGGAGGTGTTGTAATGTTGT TGTTGGGAGATGG	forward primer used for gene knock out
*at2*DownArm-rev	CCGCTCGAGCTATATTCCAAGCATGGCTTA TCCATCCATCT	reverse primer used for gene knock out
*ccpA*gRNA-for	ACGCGTCGACTGGCAAATGCTAGTGCCGT AGTTTTAGAGCTAGAAATAGCAA	forward primer used for gene knock out
*ccpA*gRNA-rev	TTTCTTCTGTCATAAATATAAAAATAAGAA GCCTGCAAAT	reverse primer used for gene knock out
*ccpA*UpArm-for	ATTTGCAGGCTTCTTATTTTTATATTTATGA CAGAAGAAA	forward primer used for gene knock out
*ccpA*UpArm-rev	TGTCCTAAATAAAACATCTTATCACCTACC AATTAC	reverse primer used for gene knock out
*ccpA*DownArm-for	GTAATTGGTAGGTGATAAGATGTTTTATTT AGGACA	forward primer used for gene knock out
*ccpA*DownArm-rev	CCGCTCGAGCATTTAGTTTTTTAATCTTA GCAAAT	reverse primer used for gene knock out
*fdh1*gRNA-for	CTTAAGGAGGAGTTTTCGTCGACAATTTTACCTTC CACGTCTTGTTTTAGAGCTAGAAA	forward primer used for gene knock out
*fdh1*gRNA-rev	CTCAGAGTCACTTATATTTCCATAAAAATA AGAAGCCTGCAAATGCAGG	reverse primer used for gene knock out
*fdh1*UpArm-for	CCTGCATTTGCAGGCTTCTTATTTTTATGG AAATATAAGTGACTCTGAG	forward primer used for gene knock out

*fdh1*UpArm-rev	GCCATCTTTATATTAAAATATGTTTTACCCC CCAATAACTTT	reverse primer used for gene knock out
*fdh1*DownArm-for	GTTATTGGGGGGTAAAACATATTTTAATAT AAAGATGGCTA	forward primer used for gene knock out
*fdh1*DownArm-rev	GCATGTCTGCAGGCCTCGAGCATATAGCA GTCTCTTTCTTT	reverse primer used for gene knock out
*fdh2*gRNA-for	TTAAGGAGGAGTTTTCGTCGACGTTTGGC TGCGGCATAAACCGTTTTAGAGCTAGAAA	forward primer used for gene knock out
*fdh2*gRNA-rev	GGAAGCAAAGCTTAGAATGGAATAAAAAT AAGAAGCCTGCAAATGCAGG	reverse primer used for gene knock out
*fdh2*UpArm-for	CCTGCATTTGCAGGCTTCTTATTTTTATTCC ATTCTAAGCTTTGCTTCC	forward primer used for gene knock out
*fdh2*UpArm-rev	GTATTCAATATAATTACCTCCATTCTTACAC TCCTTTCTTTAAG	reverse primer used for gene knock out
*fdh2*DownArm-for	AAAGAAAGGAGTGTAAGAATGGAGGTAA TTATATTGAATACC	forward primer used for gene knock out
*fdh2*DownArm-rev	GCATGTCTGCAGGCCTCGAGCTTGAATAG TTGTTACTTTTAG	reverse primer used for gene knock out
*fdh3g*RNA-for	TTAAGGAGGAGTTTTCGTCGACAATTTTG CCAGCAACTGCCTGTTTTAGAGCTAGAAA	forward primer used for gene knock out
*fdh3*gRNA-rev	CCTACACCTGCTACAGCTGCAATAAAAAT AAGAAGCCTGCAAATGCAGG	reverse primer used for gene knock out
*fdh3*UpArm-for	CCTGCATTTGCAGGCTTCTTATTTTTATTGC AGCTGTAGCAGGTGTAGG	forward primer used for gene knock out
*fdh3*UpArm-rev	CTAAGAATCACTTGATAAAAGAATAACTA CCCCATTATTTTTA	reverse primer used for gene knock out
*fdh3*DownArm-for	AAAAATAATGGGGTAGTTATTCTTTTATCA AGTGATTCTTAGG	forward primer used for gene knock out
*fdh3*DownArm-rev	GCATGTCTGCAGGCCTCGAGCTTCTGCCA CCTTTTGTACC	reverse primer used for gene knock out
pET28a-Dat1-for	CGGATCTCAGTGGTGGTGGTGGTGGTGCT CGAGTTACAATATACTTTTTAG	forward primer used for Dat purification
pET28a-Dat1-rev	CAGCAGCGGCCTGGTGCCGCGCGGCAGC CATATGATGAGTTATGAGAATT	reverse primer used for Dat purification
pET28a-Aat2-for	CGGATCTCAGTGGTGGTGGTGGTGGTGCT CGAGTTATTTTTTAATTTCATA	forward primer used for At2 purification
pET28a-At2-rev	CAGCAGCGGCCTGGTGCCGCGCGGCAGC CATATGATGATAAGAAGAGGA	reverse primer used for At2 purification
pET28a-CcpA-for	CGGATCTCAGTGGTGGTGGTGGTGGTGCT CGAGTTATTTTTTGCAGGAAT	forward primer used for CcpA purification
pET28a-CcpA-rev	CAGCAGCGGCCTGGTGCCGCGCGGCAGC CATATGGTGGCAGCATCAATTA	reverse primer used for CcpA purification
pET28a-FDH1-for	CGGATCTCAGTGGTGGTGGTGGTGGTGCT CGAGTTAAACATTCATCTTTTTCTTT	forward primer used for FDH1 purification

pET28a-FDH1-rev	CAGCAGCGGCCTGGTGCCGCGCGGCAGC CATATGATGGATAAAAAAGTTTTAACT	reverse primer used for FDH1 purification
pET28a-FDH2-for	CGGATCTCAGTGGTGGTGGTGGTGGTGCT CGAGTTAAGCGTCTTTACGCATACTC	forward primer used for FDH2 purification
pET28a-FDH2-rev	CAGCAGCGGCCTGGTGCCGCGCGGCAGC CATATGATGAAAAGTATACTAACTACT	reverse primer used for FDH2 purification
P_{1339}-for	CGAATTCGAGCTCGGTACCCGGGGATCCT TTATATTTAGTCCCTTGCCTt	forward primer used for overexpression
P_{1339}-*fdh1*-rev	GTTAAAACTTTTTTATCCATGGATCCAACT CCTCCTTAAG	reverse primer used for overexpression
fdh1-P_{1339}-for	CTTAAGGAGGAGTTGGATCCATGGATAAA AAAGTTTTAAC	forward primer used for overexpression
fdh1-rev	CATGTCTGCAGGCCTCGAGTTAGTGATGA TGATGATGATGAACATTCATCTTTTTCTTTA	reverse primer used for overexpression
qRT-*rho*-for	ACTGTAAGGCATAGTGAG	forward primer used for qRT-PCR
qRT-*rho*-rev	TCAGGTCCTGTTAAGTAG	reverse primer used for qRT-PCR
qRT-*at2*-for	AGCTAAGGAAACAGAAAATCTG	forward primer used for qRT-PCR
qRT-*at2*-rev	ATATTCCTTGTCCTCTATAATC	reverse primer used for qRT-PCR
qRT-*fdh1*-for	GATGAACCAGGATATGTACA	forward primer used for qRT-PCR
qRT-*fdh1*-rev	TGCACAATACTTTAATTCAG	reverse primer used for qRT-PCR
qRT-*fdh2*-for	GTACATCATAAAGATCGTTT	forward primer used for qRT-PCR
qRT-*fdh2*-rev	CATTAGTACATCGTGCTGAA	reverse primer used for qRT-PCR
qRT-*fdh3*-for	ACGATAAGGAAGCCTAAGAT	forward primer used for qRT-PCR
qRT-*fdh3*-rev	TAGCACAGTTGTCCACATTA	reverse primer used for qRT-PCR
EMSA-*at2*-for	AGCCAGTGGCGATAAGAAAAGGACGAC ATGGTAGTGGGAC	forward primer used for EMSA
EMSA-*at2*-rev	AGCCAGTGGCGATAAGTTATTTTTTAATT TCATACATT	reverse primer used for EMSA
EMSA-*at2*mu-for	TTCCTTGTCCTCTATAATCTTTGCCCCCCTA TACACTCTGTAT	forward primer used for EMSA
EMSA-*at2*mu-rev	GATTATAGAGGACAAGGAATAGGGGGGCA ATTATTTGATCATG	reverse primer used for EMSA
EMSA-*fdh1*-for	AGCCAGTGGCGATAAGTATTTATAAAAGAT CTGAGG	forward primer used for EMSA

EMSA-*fdh1*-rev	AGCCAGTGGCGATAAGCGCCACAGTAAGGACAAACA	reverse primer used for EMSA
EMSA-20590-for	AGCCAGTGGCGATAAGCATAAACGGAGTTCTTGGTT	forward primer used for EMSA
EMSA-20590-rev	AGCCAGTGGCGATAAGAATTTACCTCCATTGTAAA	reverse primer used for EMSA
FDH1-29Q-for	TCTGCTTGAATTATTTTGCCATTCTTTAC	forward primer used for FDH1 purification
FDH1-29Q-rev	GCAAAATAATTCAAGCAGAGCCTGCTAAT	reverse primer used for FDH1 purification
FDH1-29R-for	CTCTGCTCTAATTATTTTGCCATTCTTTAC	forward primer used for FDH1 purification
FDH1-29R-rev	GCAAAATAATTAGAGCAGAGCCTGCTAAT	reverse primer used for FDH1 purification
FDH1-64Q-for	ATCATCGGATGTTGAATTCTAGATGTCAAT	forward primer used for FDH1 purification
FDH1-64Q-rev	ATTGACATCTAGAATTCAACATCCGATGAT	reverse primer used for FDH1 purification
FDH1-96Q-for	GGAATCAGGTCCATATTGCTCTTTTATTTG	forward primer used for FDH1 purification
FDH1-96Q-rev	CAAATAAAAGAGCAATATGGACCTGATTC	reverse primer used for FDH1 purification
FDH1-196Q-for	ATCTACAACTATTACTTGTGCACCCTTTTG	forward primer used for FDH1 purification
FDH1-196Q-rev	CAAAAGGGTGCACAAGTAATAGTTGTAGA	reverse primer used for FDH1 purification
FDH1-367Q-for	AACACCCCAAGCTTGTTCAAATTTTTCCCT	forward primer used for FDH1 purification
FDH1-367Q-rev	AGGGAAAAATTTGAACAAGCTTGGGGTG	reverse primer used for FDH1 purification
FDH1-393Q-for	TAATATGCCTTTATTTGATCCTCTTTAAGC	forward primer used for FDH1 purification
FDH1-393Q-rev	GCTTAAAGAGGATCAAATAAAGGCATATT	reverse primer used for FDH1 purification
FDH1-466Q-for	CTTAGGTTCTACAGCTTGTCTAAATCTCTG	forward primer used for FDH1 purification
FDH1-466Q-rev	CAGAGATTTAGACAAGCTGTAGAACCTAA	reverse primer used for FDH1 purification
FDH1-517Q-for	TTCTTCGAGTCTTTGATAAGTTGCTCCTTT	forward primer used for FDH1 purification
FDH1-517Q-rev	AAAGGAGCAACTTATCAAAGACTCGAAG	reverse primer used for FDH1 purification
FDH1-545Q-for	TAGGTGTATTAAATTGATTACCTTTGTAGA	forward primer used for FDH1 purification

FDH1-545Q-rev	TCTACAAAGGTAATCAATTTAATACACCTA	reverse primer used for FDH1 purification
FDH1-616Q-for	ACCATCTATTATTCCTTGAGCCTTTGCATC	forward primer used for FDH1 purification
FDH1-616Q-rev	GATGCAAAGGCTCAAGGAATAATAGATGG	reverse primer used for FDH1 purification
FDH1-677Q-for	TACTGCACAATATTGTAATTCAGGAGTTTT	forward primer used for FDH1 purification
FDH1-677Q-rev	AAAACTCCTGAATTACAATATTGTGCAGTA	reverse primer used for FDH1 purification
FDH1-682Q-for	TCTTTTATAGCTTCTATTTGTACTGCACAAT ACTTTAATT	forward primer used for FDH1 purification
FDH1-682Q-rev	AATTAAAGTATTGTGCAGTACAAATAGAA GCTATAAAAGA	reverse primer used for FDH1 purification
FDH1-687Q-for	TTCTTTCTGATCTTGTATAGCTTCTATTTT	forward primer used for FDH1 purification
FDH1-687Q-rev	AAAATAGAAGCTATACAAGATCAGAAAGA	reverse primer used for FDH1 purification
FDH1-690Q-for	ACTTTTCAGCTTCTTGCTGATCTTTTATAG	forward primer used for FDH1 purification
FDH1-690Q-rev	CTATAAAAGATCAGCAAGAAGCTGAAAAG	reverse primer used for FDH1 purification
FDH1-694Q-for	TCTTTTATAAATTGTTCAGCTTCTTTCTGA	forward primer used for FDH1 purification
FDH1-694Q-rev	TCAGAAAGAAGCTGAACAATTTATAAAAG	reverse primer used for FDH1 purification
FDH1-697Q-for	ATCATATTGATCTTGTATAAACTTTTCAGC	forward primer used for FDH1 purification
FDH1-697Q-rev	GCTGAAAAGTTTATACAAGATCAATATGAT	reverse primer used for FDH1 purification
FDH1-704Q-for	TTCATCTTTTTTTGTAAAAGATCATATTGA	forward primer used for FDH1 purification
FDH1-704Q-rev	TCAATATGATCTTTTACAAAAAAAGATGAA	reverse primer used for FDH1 purification
FDH2-46Q-for	TAAACCAAGGGAAGCTCTGCTCACAAGG CCGTTTTGGTTTTGACTTTGT	forward primer used for FDH2 purification
FDH2-46Q-rev	ACAAAGTCAAAACCAAAACGGCCTTGTG AGCAGAGCTTCCCTTGGTTTA	reverse primer used for FDH2 purification
FDH2-57Q-for	TTGGTTTTGACTTTGTACATCATCAAGATC GTTTAACTAGTCCTTTGAT	forward primer used for FDH2 purification
FDH2-57Q-rev	ATCAAAGGACTAGTTAAACGATCTTGATG ATGTACAAAGTCAAAACCAA	reverse primer used for FDH2 purification
FDH2-92Q-for	TTTCTAAAATCAAAAAAACTGTACAAGAA TATGGTTCTGACAGTGTTGC	forward primer used for FDH2 purification

FDH2-92Q-rev	GCAACACTGTCAGAACCATATTCTTGTACAGTTTTTTTGATTTTAGAAA	reverse primer used for FDH2 purification
FDH2-259Q-for	GAGGATTTCAAAGAAGTTGTAATGCAATTCACGCCAAAGTATACATCAAG	forward primer used for FDH2 purification
FDH2-259Q-rev	CTTGATGTATACTTTGGCGTGAATTGCATTACAACTTCTTTGAAATCCTC	reverse primer used for FDH2 purification
FDH2-270Q-for	CCAAAGTATACATCAAGTATAACTCAAGTTCCAGCAGACAAGATAATAGA	forward primer used for FDH2 purification
FDH2-270Q-rev	TCTATTATCTTGTCTGCTGGAACTTGAGTTATACTTGATGTATACTTTGG	reverse primer used for FDH2 purification
FDH2-275Q-for	GTATAACTAAGGTTCCAGCAGACCAAATAATAGAAGCTGCAAGAATTAT	forward primer used for FDH2 purification
FDH2-275Q-rev	ATAATTCTTGCAGCTTCTATTATTTGGTCTGCTGGAACCTTAGTTATAC	reverse primer used for FDH2 purification
FDH2-363Q-for	TAACTAATCCTGATGTGAAAGCTCAATTTGAAAAAGCATGGAATGCTCA	forward primer used for FDH2 purification
FDH2-363Q-rev	TGAGCATTCCATGCTTTTTCAAATTGAGCTTTCACATCAGGATTAGTTA	reverse primer used for FDH2 purification
FDH2-678Q-for	AGTTTGCTCGGTAAAGATAAAACAATTAGATTTTTCAGATTTTGGATGTC	forward primer used for FDH2 purification
FDH2-678Q-rev	GACATCCAAAATCTGAAAAATCTAATTGTTTTATCTTTACCGAGCAAACT	reverse primer used for FDH2 purification
CcpA-56Q-for	CTTGTTCTTTGAGTTTGAAGACTTCTTGCT	forward primer used for CcpA purification
CcpA-56Q-rev	AGCAAGAAGTCTTCAAACTCAAAGAACA	reverse primer used for CcpA purification
CcpA-56R-for	CTTGTTCTTTGAGTTCTAAGACTTCTTGCT	forward primer used for CcpA purification
CcpA-56R-rev	AGCAAGAAGTCTTAGAACTCAAAGAACA	reverse primer used for CcpA purification
CcpA-202Q-for	TATTTTCCTCAAGAACTTGTCTATACCCTT	forward primer used for CcpA purification
CcpA-202Q-rev	AAGGGTATAGACAAGTTCTTGAGGAAAAT	reverse primer used for CcpA purification
CcpA-210Q-for	ACTTTTGTCCAATTGCAGGTTATTTTCCTC	forward primer used for CcpA purification
CcpA-210Q-rev	GAGGAAAATAACCTGCAATTGGACAAAA	reverse primer used for CcpA purification
CcpA-215Q-for	ACATTTCCAAAATAAACTTGACTTTTGTCC	forward primer used for CcpA purification
CcpA-215Q-rev	GGACAAAAGTCAAGTTTATTTTGGAAATG	reverse primer used for CcpA purification
CcpA-228Q-for	TTTCACTCATTCCTTGGTAACCATCTTTTG	forward primer used for CcpA purification

CcpA-228Q-rev	CAAAAGATGGTTACCAAGGAATGAGTGA	reverse primer used for CcpA purification
CcpA-236Q-for	GAATCTACTTTAGTTTGACTGAGTATTTCA	forward primer used for CcpA purification
CcpA-236Q-rev	TGAAATACTCAGTCAAACTAAAGTAGATT	reverse primer used for CcpA purification
CcpA-306Q-for	TGCTTATTTATTAATTGTATGAGCATCCTC	forward primer used for CcpA purification
CcpA-306Q-rev	GAGGATGCTCATACAATTAATAAATAAGCA	reverse primer used for CcpA purification
CcpA-310Q-for	TTCAAGTTCTTGTTGATTTATTAATTTTAT	forward primer used for CcpA purification
CcpA-310Q-rev	ATAAAATTAATAAATCAACAAGAACTTGA	reverse primer used for CcpA purification
CcpA-315Q-for	ATACATAGTTCTTTTGTTCAAGTTCTTGCT	forward primer used for CcpA purification
CcpA-315Q-rev	AGCAAGAACTTGAACAAAAGAACTATGTA	reverse primer used for CcpA purification
CcpA-323Q-for	TTTTTTGCAGGAATCCCTCTCAATCAATTG ATGTGACAATACAT	forward primer used for CcpA purification
CcpA-323Q-rev	CAGCAGCGGCCTGGTGCCGCGCGGCAGC CATATGGTGGCAGCATCAATTAAAGACGT	reverse primer used for CcpA purification
CcpA-56R-for	CTTGTTCTTTGAGTTCTAAGACTTCTTGCT	forward primer used for CcpA purification
CcpA-56R-rev	AGCAAGAAGTCTTAGAACTCAAAGAACA AG	reverse primer used for CcpA purification
